# A Stable N‐Heterocyclic Silylene with a 1,1′‐Ferrocenediyl Backbone

**DOI:** 10.1002/anie.202011691

**Published:** 2020-12-01

**Authors:** Nadine Weyer, Myron Heinz, Julia I. Schweizer, Clemens Bruhn, Max C. Holthausen, Ulrich Siemeling

**Affiliations:** ^1^ Institut für Chemie Universität Kassel Heinrich-Plett-Straße 40 34132 Kassel Germany; ^2^ Institut für Anorganische und Analytische Chemie Goethe-Universität Max-von-Laue-Straße 7 60438 Frankfurt am Main Germany

**Keywords:** carbene homologues, insertion, metallocenes, silicon, subvalent compounds

## Abstract

The N‐heterocyclic silylene [{Fe(η^5^‐C_5_H_4_‐NDipp)_2_}Si] (**1DippSi**, Dipp=2,6‐diisopropylphenyl) shows an excellent combination of pronounced thermal stability and high reactivity towards small molecules. It reacts readily with CO_2_ and N_2_O, respectively affording (**1DippSi**O_2_)_2_C and (**1DippSi**O)_2_ as follow‐up products of the silanone **1DippSi**O. Its reactions with H_2_O, NH_3_, and FcPH_2_ (Fc=ferrocenyl) furnish the respective oxidative addition products **1DippSi**(H)X (X=OH, NH_2_, PHFc). Its reaction with H_3_BNH_3_ unexpectedly results in B−H, instead of N−H, bond activation, affording **1DippSi**(H)(BH_2_NH_3_). DFT results suggest that dramatically different mechanisms are operative for these H−X insertions.

## Graphical abstract

A stable cyclic diaminosilylene featuring a ferrocene‐based backbone is reported. The dicoordinate Si^II^ atom is part of a six‐membered FeC_2_N_2_Si ring and shows a comparatively large bond angle of ca. 107°. This N‐heterocyclic silylene can activate strong bonds (C=O, N=O, B−H, N−H, P−H, O−H) under ambient conditions.

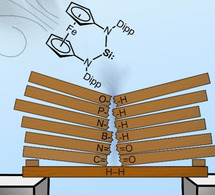

The N‐heterocyclic silylene (NHSi) **A** ^[1]^ (Figure 1) is a heavier NHC^[2]^ analogue and represents the first stable compound containing divalent and dicoordinate silicon.^[3]^ Backbone‐saturated congeners are significantly more reactive. For example, whereas 1,3‐bis(2,6‐diisopropylphenyl)imidazolin‐2‐ylidene (IPr) is inert towards PH_3_, the backbone‐saturated congener SIPr readily inserts into a P−H bond,^[4]^ and silylene **B** undergoes self‐insertion into an Si−N bond during its tetramerisation.^[5]^ The first isolable dialkylsilylene **C** ^[6]^ exhibits a more pronounced ambiphilicity than diaminosilylenes and rearranges to a Si^IV^ compound.^[7]^

**Figure 1:**
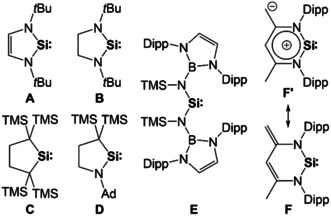
Silylenes **A**–**F** (Ad=1‐adamantyl, Dipp=2,6‐diisopropylphenyl, TMS=trimethylsilyl).

The rapid development of carbene chemistry has led to acyclic diaminocarbenes (ADACs),^[8]^ ring‐expanded NHCs (reNHCs) with ring sizes >5^[9]^ and cyclic (alkyl)(amino)carbenes (CAACs),^[10]^ which are all closely related to standard NHCs, but exhibit a more pronounced ambiphilicity, and hence higher reactivity.^[11]^ While more than a dozen silicon analogues of standard NHCs have been isolated,[^3^, ^12^] only a single example each has been reported for stable silicon analogues of CAACs,^[13]^ ADACs^[14]^ and reNHCs,^[15]^ viz. silylenes **D**–**F** (Figure 1). The ambivalent reactivity of reNHSi **F** was rationalised by a significant contribution of N‐ylidic canonical structures summarised as **F′**. We here report on the reNHSi [{Fe(η^5^‐C_5_H_4_‐NDipp)_2_}Si] (**1DippSi**), which contains a six‐membered FeC_2_N_2_Si ring. **1DippSi** is an analogue of our stable ferrocene‐based NHCs, whose ambiphilicity allowed for small‐molecule activation reactions unprecedented for diaminocarbenes.[^16^, ^17^]

Our attempts to obtain reNHSis of the type **1RSi** by reduction of corresponding Si^IV^ dihalides **1RSi**X_2_ (X=Cl, Br) or by α‐elimination of HCl from **1RSi**(H)Cl were unsuccessful.^[18]^ An alternative approach, which was introduced for the acyclic diaminosilylene (ADASi) **E**, is the reaction of [SiCl_2_(IPr)]^[19]^ with the corresponding lithium amide.^[14]^ This Si^II^ precursor turned out to be the key to success. Its reaction with **1Mes**Li_2_ in C_6_D_6_ afforded the silylene **1MesSi** together with IPr (Scheme 1). Although too unstable for isolation, **1MesSi** was sufficiently persistent at room temperature for detecting its ^29^Si NMR signal (*δ*=121.5 ppm), which is significantly downfield‐shifted with respect to reNHSi **F** (*δ*=88.4 ppm)^[15]^ and NHSi **A** (78.3 ppm).^[1]^ The signal of the Si^II^ atom in ADASi **E** was observed at even lower field (*δ*=204.6 ppm).^[14]^ Trapping of **1MesSi** with (PhSe)_2_ at room temperature in benzene solution afforded **1MesSi**(SePh)_2_; details are provided in the Supporting Information (SI). The bulkier homologue **1DippSi**, obtained from [SiCl_2_(IPr)] and **1Dipp**Li_2_ in toluene at room temperature, is sufficiently stable for isolation (Scheme 1). IPr and **1DippSi** could not be separated by crystallisation or sublimation. It was possible to remove IPr from ADASi **E** by stirring a hexane solution of the mixture at room temperature under an atmosphere of CO_2_, which led to the precipitation of IPr(CO_2_).^[14]^ This method was not successful in our case, because, in contrast to **E**, **1DippSi** reacts swiftly with CO_2_ under the same mild conditions, affording the orthocarbonate (**1DippSi**O_2_)_2_C (Scheme 2; see the SI). The primary products are most likely CO and the silanone **1DippSi**O,^[20]^ which subsequently undergoes a cycloaddition with CO_2_ in a 2:1 ratio. When generated by reaction of **1DippSi** with N_2_O in benzene at room temperature, this silanone forms the expected dimer (**1DippSi**O)_2_ (Scheme 2; see the SI).^[21]^ An analogous stepwise reaction with CO_2_ was first reported for decamethylsilicocene (Cp*_2_Si).^[22]^ Dialkylsilylene **C** ^[23]^ as well as IPr=N‐Si‐OSi*t*Bu_3_ and IPr=N‐Si‐Si(SiMe_3_)_3_, an acyclic (imino)(siloxy)‐ and (imino)(silyl)silylene,^[24]^ are the only examples containing dicoordinate Si^II^ in this context to date.^[25]^ We found that IPr is easily removed by complexation with ZnCl_2_, which is inert towards **1DippSi**. In contrast to **1DippSi**, [ZnCl_2_(IPr)]^[26]^ is insoluble in hexane.

**Scheme 1:**
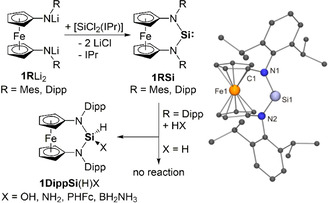
Synthesis of **1MesSi** (persistent, Mes=mesityl) and **1DippSi** (stable) and reactions of the latter with H_2_O, NH_3_, FcPH_2_ (Fc=ferrocenyl), and H_3_BNH_3_ under ambient conditions in benzene or toluene. Selected bond lengths [Å] and angles [°] for **1DippSi**: Si1‐N1 1.7327(12), Si1‐N2 1.7344(12), N1‐Si1‐N2 106.58(6); sum of angles (Σ∡) at N1 359.9, at N2 360.0.

**Scheme 2:**
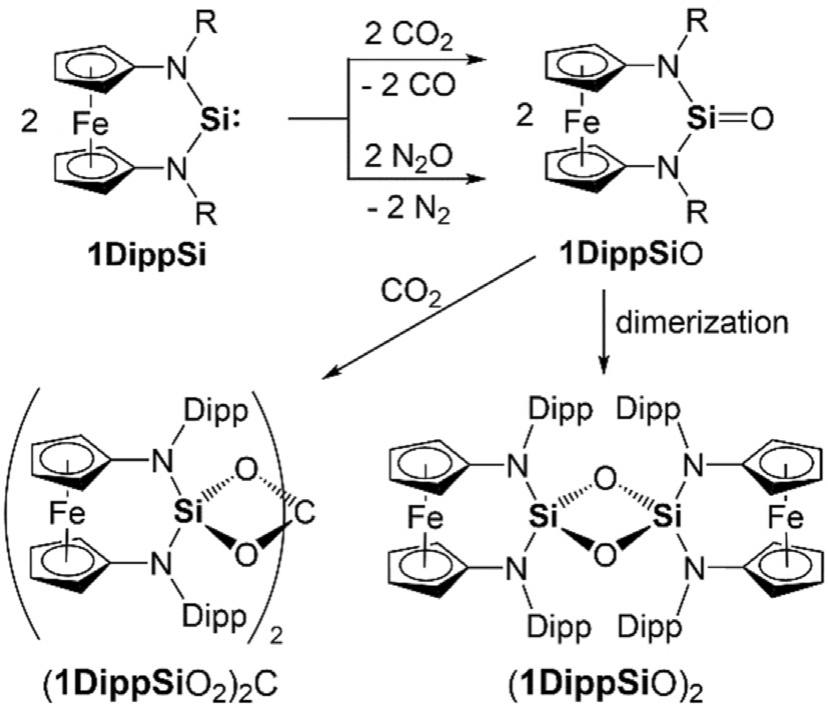
Reactions of **1DippSi** with CO_2_ and N_2_O under ambient conditions in benzene, respectively affording (**1DippSi**O_2_)_2_C and (**1DippSi**O)_2_ via the silanone **1DippSi**O as assumed intermediate.

The ^29^Si NMR signal of **1DippSi** is located at *δ*=115.7 ppm, upfield‐shifted by 6 ppm with respect to **1MesSi. 1DippSi** was structurally characterised by X‐ray diffraction (Scheme 1). The Si bond angle (106.6°) lies in between the values determined for reNHSi **F** (99.3°)^[15]^ and ADASi **E** (110.9°)^[14]^ and is close to that reported for a heterocyclic silylene with a six‐membered ring containing an NSi^II^BP unit.^[27]^ Silylenes whose dicoordinate Si^II^ atom is part of a five‐membered ring exhibit more acute Si bond angles close to 90°.[^3^, ^6^, ^12^, ^13^, ^28^]

Similar to **1MesSi**, **1DippSi** undergoes an oxidative addition with (PhSe)_2_ in benzene solution at room temperature to afford **1DippSi**(SePh)_2_ (see the SI). We next addressed the oxidative addition of strong H−X bonds of different polarities, which is of fundamental importance for chemical synthesis and catalysis.^[29]^ While **1DippSi** is inert towards H_2_ under ambient conditions, it reacted readily with H_2_O, NH_3_, and FcPH_2_, affording the corresponding derivatives of the type **1DippSi**(H)X (Scheme 1, Figure 2; X=OH, NH_2_, PHFc; see the SI). The reaction of H_2_O with stable dicoordinate Si^II^ compounds to the corresponding hydroxysilane has been reported only for the metallasilylene [Cp*(CO)_3_Cr‐Si‐SIPr]^+[30]^ as well as for **A** ^[31]^ and **F**.^[32]^ The hydroxysilanes **A**(H)OH and **F**(H)OH were not observed, but their intermediacy was merely inferred from the products isolated. In contrast, the analogous NH_3_ addition product **F**(H)NH_2_ was obtained in high yield from the reaction of **F** with NH_3_.^[33]^ **F** is the exception to the rule that five‐ and six‐membered NHSis cannot be employed for NH_3_ activation, although they are more Lewis acidic and have a smaller singlet‐triplet gap compared to the corresponding NHCs.^[34]^ Apart from [Cp*(CO)_3_Cr‐Si‐SIPr]^+^,^[30]^ Dipp(Me_3_Si)N‐Si‐Si(SiMe_3_)_3_ ^[35]^ and IPr=N‐Si‐OSi*t*Bu_3_ ^[24a]^ we are not aware of any other stable silylene to undergo an oxidative addition of NH_3_. The reaction of NH_3_ with **1DippSi** is remarkable because NH_3_ activation is a challenging target even for transition metal complexes^[36]^—the potential of low‐valent main‐group element compounds in this context was uncovered only recently.[^25^, ^37^] The reaction of **1DippSi** with FcPH_2_ afforded the oxidative addition product **1DippSi**(H)(PHFc). In view of the ability of **1DippSi** for N‐H activation, the activation of a P−H bond, which is weaker than an N−H bond by ca. 100 kJ mol^−1^, is not unexpected;^[38]^ reNHSi **F** is also capable of P‐H activation.^[39]^ We next addressed the reaction of **1DippSi** with H_3_BNH_3_ (Scheme 1),^[40]^ expecting the formation of **1DippSi**H_2_, most likely by transfer of a protic and a hydridic H atom^[41]^ to the divalent atom, as was observed for 1,3‐di‐*tert*‐butylimidazolin‐2‐ylidene^[33]^ and reNHSi **F**.^[42]^ Instead, the reaction furnished **1DippSi**(H)(BH_2_NH_3_) (Figure 2), although the B−H bond is stronger than the N−H bond of H_3_BNH_3_.^[43]^ First B−H bond activation reactions with Si^II^ compounds were reported only recently,^[44]^ and the reaction of the (silyl)(vinyl)silylene ^Me^IPr=CH‐Si‐Si(SiMe_3_)_3_ (^Me^IPr=1,3‐bis(2,6‐diisopropylphenyl)‐4,5‐dimethylimidazolin‐2‐ylidene) with pinacolborane (HBPin), which affords ^Me^IPr=CH‐Si(H)(Bpin)‐Si(SiMe_3_)_3_, is the only example involving dicoordinate Si^II^.^[45]^

**Figure 2:**
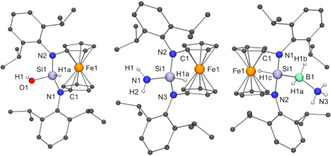
Molecular structures of **1DippSi**(H)OH (left; selected bond lengths [Å] and angles [°]: Si1‐N1 1.7197(16), Si1‐N2 1.7199(16), Si1‐O1 1.6071(16), N1‐Si1‐N2 111.49(8); Σ∡ at N1 359.9, at N2 359.4), **1DippSi**(H)NH_2_ (middle; selected bond lengths [Å] and angles [°]: Si1‐N1 1.691(4), Si1‐N2 1.721(3), Si1‐N3 1.730(3), N2‐Si1‐N3 111.72(14); Σ∡ at N2 358.5, at N3 358.0) and **1DippSi**(H)(BH_2_NH_3_) (right; selected bond lengths [Å] and angles [°]: Si1‐N1 1.7571(14), Si1‐N2 1.7694(14), Si1‐B1 2.008(2), N3‐B1 1.600(3), N1‐Si‐N2 107.84(7), Si1‐B1‐N1 116.41(13); Σ∡ at N1 358.3, at N2 356.6).

We performed a DFT study on the electronic characteristics and the reactivity of **1**, the full molecular model of **1DippSi**.^[46]^ At the PBEh‐3c level of DFT employed, the HOMO comprises the expected silylene lone pair together with significant contributions of the ferrocene moiety and the LUMO is dominated by the silylene p‐orbital, with a substantial HOMO–LUMO energy separation of Δ*E*_H/L_=6.4 eV. The unexpectedly low computed singlet‐triplet energy difference of Δ*E*_S/*T*_=0.4 eV does not correlate with this value because the lowest triplet state arises from a local excitation within the ferrocene moiety and does not involve the silylene p‐orbital (Figure 3).^[47]^

**Figure 3:**
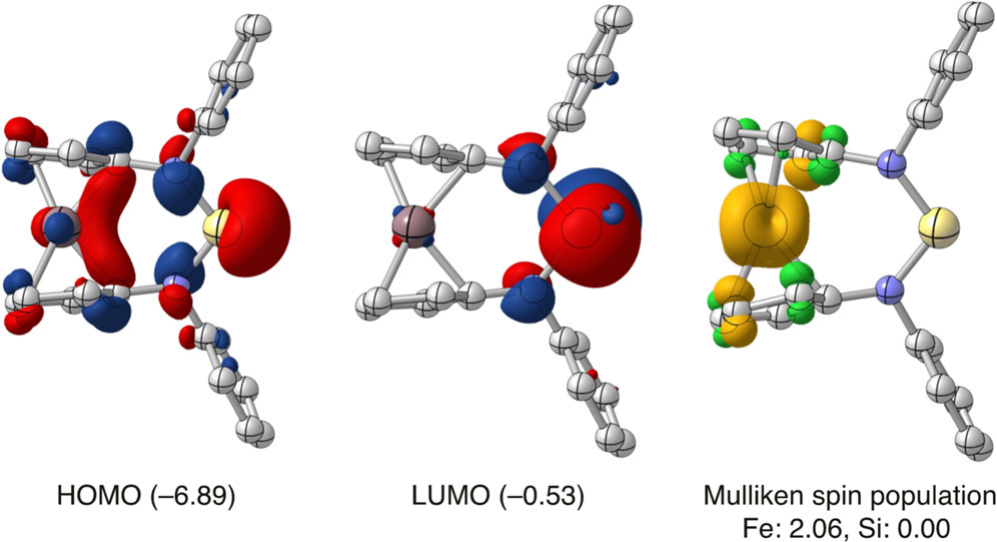
Frontier molecular orbitals and triplet spin‐density distribution computed for **1** (i. e. the full molecular model of **1DippSi**; orbital energies in eV, isocontour surfaces at ±0.05 a_0_^−3/2^ for orbitals and ±0.005 a_0_^−3^ for the spin density, α spin: yellow, β spin: green; *i*Pr groups and H atoms not shown).

Surprisingly, direct oxidative addition of H_2_O and NH_3_ to **1** is precluded by high kinetic barriers for both substrates (35 and 42 kcal mol^−1^, respectively), and two distinct alternative pathways were identified instead. The lowest‐energy pathway for NH_3_ activation commences with the formation of adduct **2** (Scheme 3, top). Proton transfer is facilitated by a second NH_3_ molecule acting as a proton shuttle and the experimentally observed product **3** is formed in a strongly exergonic step with a moderate overall barrier of 20 kcal mol^−1^. H_2_O, in turn, does not form a datively bonded adduct with **1**, but directly adds across an Si−N bond via **TS2** to form hydroxysilylene **4** in an exergonic step (Scheme 3, bottom). From there on out, silanone **5** is formed through a water‐assisted proton transfer;^[48]^ the experimentally observed product **6** results in a strongly exergonic step after passage of a minute barrier in **TS4**.

**Scheme 3:**
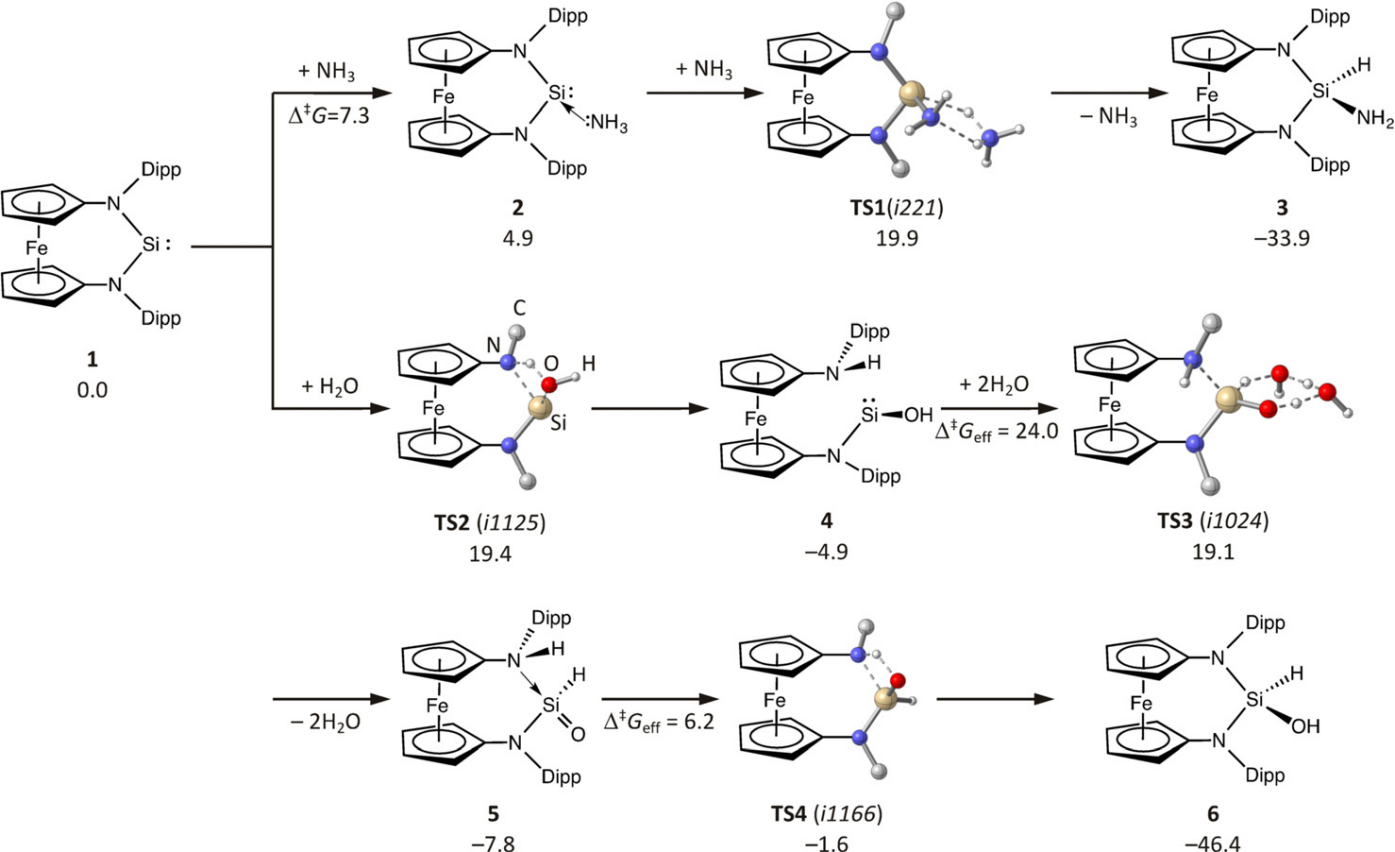
Computed lowest‐energy reaction pathway for the formal H‐X oxidative addition to **1** with NH_3_ (top) and H_2_O (bottom, Δ*G* ^298^ in kcal mol^−1^). Bonds formed or broken in transition states are dashed, unreactive H atoms are omitted and the orientation of the Dipp substituents in the transition state is indicated by showing the respective C_ipso_ atom only.

The quantum‐chemical evaluation discloses a concerted dehydrogenation of H_3_BNH_3_ by **1** as initial step along the lowest‐energy pathway for ammonia‐borane activation (Scheme 4). Alternative direct insertion of **1** into an N−H or B−H bond is precluded by high kinetic barriers (53 and 35 kcal mol^−1^, respectively; see the SI). Whereas the resulting silane **7** forms as an unreactive side product,^[49]^ H_2_BNH_2_ is a highly reactive species that has been thoroughly studied in the thermal and catalytic dehydrogenation of H_3_BNH_3_ and is known to polymerize below −150 °C.^[50]^

**Scheme 4:**
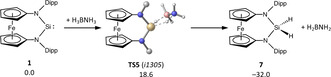
Computed reaction path for the formation of **7** from **1** and H_3_BNH_3_; Δ*G* ^298^ in kcal mol^−1^.

Obviously, B‐H insertion of **1** in H_2_BNH_2_ competes efficiently with the polymerization of the latter, leading to the formation of **8** (Scheme 5). With a low barrier of 12 kcal mol^−1^ **8** can dehydrogenate a second equivalent of H_3_BNH_3_ through intermediate **9** yielding the experimentally observed product **10** while regenerating H_2_BNH_2_. After initial formation of H_2_BNH_2_ from **1** and H_3_BNH_3_ the follow‐up reaction cascade involving B‐H insertion by **1** and subsequent dehydrogenation of H_3_BNH_3_ is kinetically favoured over the formation of **7**.

**Scheme 5:**
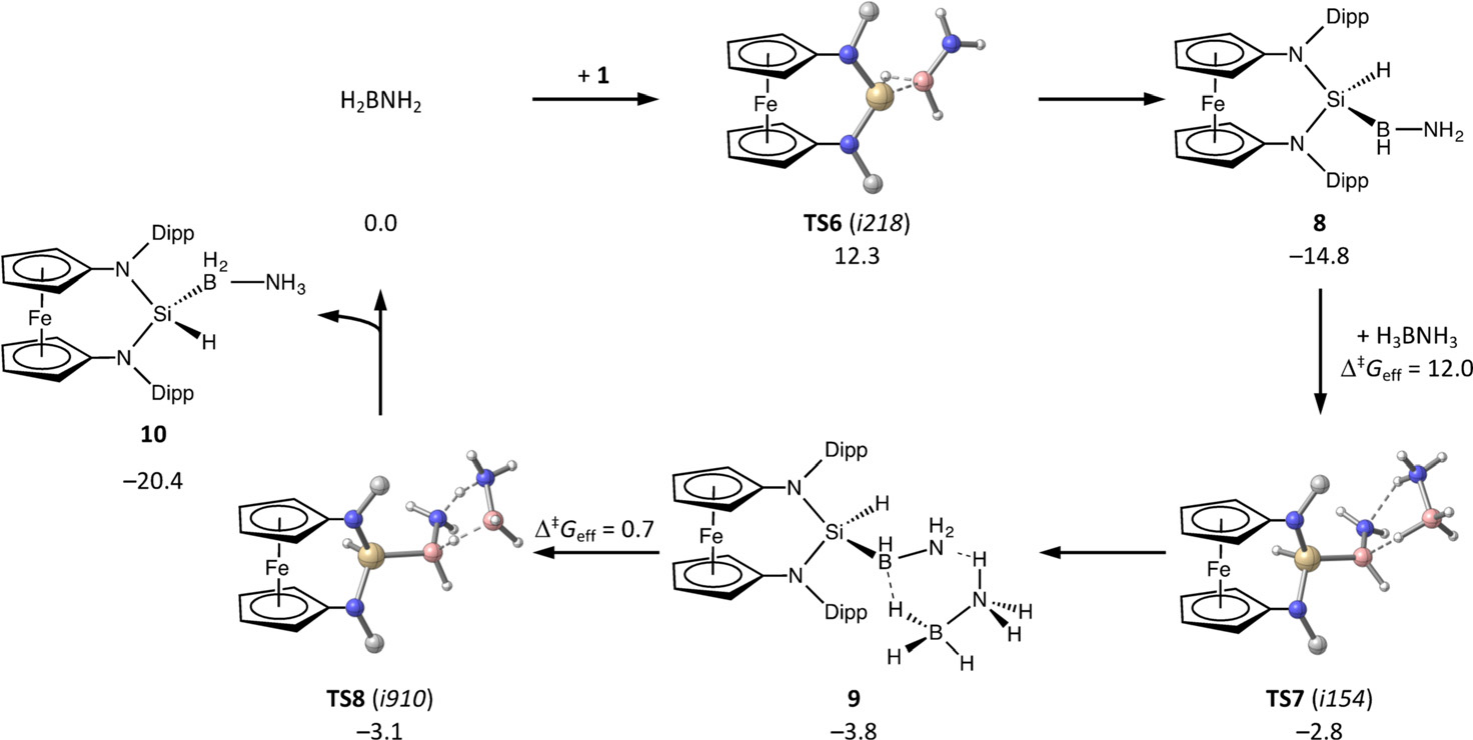
Computed reaction path for the formation of **10** from **1**, H_2_BNH_2_and H_3_BNH_3_ (Δ*G* ^298^ in kcal mol^−1^ relative to the separated reactants, activation barriers relative to the preceding minimum).

In conclusion, we have described the synthesis and reactivity of the new stable reNHSi **1DippSi. 1DippSi** reacts readily with N_2_O and CO_2_, which is in contrast to the inertness of **F**, the only other stable cyclic diaminosilylene featuring a ring‐expanded structure known to date.^[25a]^ Studies on the reactivity of **1DippSi** towards H‐X bonds of different strengths and polarities show parallels to previous reactivity studies on other silylenes. The reactions with NH_3_ and H_2_O both give the H‐X insertion products. Mechanistically, however, they differ significantly. More particularly, the lowest‐energy path of the reaction with H_2_O involves the N‐Si cooperative activation of an O−H bond. For H_3_BNH_3_ the reaction mechanism consists of two key elementary steps, the first one being the dehydrogenation of H_3_BNH_3_ to H_2_BNH_2_, which subsequently catalyses the conversion of **1DippSi** to **1DippSi**(H)(BH_2_NH_3_) with H_3_BNH_3_. In contrast to H_3_BNH_3_, H_2_BNH_2_ has a vacant p‐orbital, which enables insertion of the silylene in a B−H bond in the second step. This silylborane can in turn dehydrogenate a second equivalent of H_3_BNH_3_ to give the final product **1DippSi**(H)(BH_2_NH_3_).

## Conflict of interest

The authors declare no conflict of interest.

## Supporting information

As a service to our authors and readers, this journal provides supporting information supplied by the authors. Such materials are peer reviewed and may be re‐organized for online delivery, but are not copy‐edited or typeset. Technical support issues arising from supporting information (other than missing files) should be addressed to the authors.

Supplementary
