# Supplementary material for: A Stable N‐Heterocyclic Silylene with a 1,1′‐Ferrocenediyl Backbone
Source: Angew Chem Int Ed Engl. 2020 Dec 1;60(5):2624–8. doi: 10.1002/anie.202011691 (PMC7898919; doi:10.1002/anie.202011691)
Supplement: Supplementary file 1 — Supplementary [file ANIE-60-2624-s001.pdf]

Angewandte Chemie
Eine Zeitschrift der Gesellschaft Deutscher Chemiker

Supporting Information

A Stable N-Heterocyclic Silylene with a 1,1′-Ferrocenediyl Backbone

Nadine Weyer, Myron Heinz, Julia I. Schweizer, Clemens Bruhn, Max C. Holthausen, and Ulrich Siemeling**

anie_202011691_sm_miscellaneous_information.pdf

Table of Contents

**I**	**Experimental Section**		S2
	A	Compound Synthesis	S2
	B	X-Ray Crystallography	S8
	C	Plots of NMR Spectra	S15
**II**	**Computational Details**		S33
	**References**		S69

I Experimental Section

A Compound Synthesis

General considerations

All reactions involving air-sensitive compounds were performed in an inert atmosphere (argon or dinitrogen) by using standard Schlenk techniques or a conventional glovebox. Starting materials were procured from standard commercial sources and used as received. A sample of high-purity ammonia-borane was kindly provided by Prof. Umit B. Demirci (University of Montpellier, France). [$SiCl_2$(IPr)],[S1] **1Mes**Li_2,[S2] and **1Dipp**Li_2,[S3] were synthesised by adapted versions of the published procedures. NMR spectra were recorded at ambient temperature with Varian NMRS-500 and MR-400 spectrometers operating at 500 and 400 MHz, respectively, for ^{1}H. Elemental analyses were carried out with a HEKAtech Euro EA-CHNS elemental analyser at the Institute of Chemistry, University of Kassel, Germany.

Synthesis of 1MesSi (NMR experiment)

[$SiCl_2$(IPr)] (10 mg, 0.02 mmol) and **1Mes**Li_2 (9 mg, 0.02 mmol) were each dissolved in C_6D_6 (0.4 mL). Both solutions were frozen, then warmed carefully to a slushy state and combined in an NMR tube. After reaching room temperature, the solution was subjected to immediate NMR spectroscopic analysis.

^{1}H NMR (500 MHz, C_6D_6): δ= 6.79 (s, 4H, C_6H_2), 4.33, 3.88 (2 s, 2 × 4H, C_5H_4), 2.54 (s, 12H, *o*-Me), 2.10 ppm (s, 6H, *p*-Me). ^{13}C{^{1}H} NMR (101 MHz, C_6D_6): δ= 146.2, 137.4, 130.0, 123.8 (4 × $C_6H_2Me_3$), 105.4 (C_{ipso} C_5H_4), 68.1, 67.6 (2 × CH C_5H_4); 20.9, 20.3 ppm (2 × Me). ^{29}Si{^{1}H} NMR (99 MHz, C_6D_6): δ= 121.5 ppm.

Synthesis of 1DippSi

A suspension of [$SiCl_2$(IPr)] (176 mg, 0.36 mmol) in toluene (4 mL) was cooled to −20 °C and slowly added to a stirred solution of **1Dipp**Li_2 (197 mg, 0.36 mmol) in a mixture of toluene (15 mL) and *n*-hexane (10 mL) cooled to −20 °C. The cooling bath was removed and stirring continued for 4 h. Diethyl ether (3 mL) and zinc chloride (49 mg, 0.36 mmol) were added and stirring was continued for 12 h. Volatile components were removed under reduced pressure. The residue was extracted with *n*-hexane (3 × 15 mL). The extracts were combined and their volume reduced to ca. 5 mL under reduced pressure, which afforded the product as a

yellow crystalline solid. A second crop was obtained by reducing the volume of the mother liquor to ca. 2 mL under reduced pressure. Yield 87 mg (43 %).

$C_{34}H_{42}N_2FeSi$ (562.64): calcd. C 72.58, H 7.52, N 4.98 %; found C 71.96, H 7.62, N 4.73 %.

^{1}H NMR (500 MHz, C_6D_6): δ= 7.14 – 7.09 (m, 6H, C_6H_3), 4.48 (s, 4H, C_5H_4), 4.04 (sept, $^{3}J_{HH}$ = 6.9 Hz, 4H, C*H*Me$_2$), 3.90 (s, 4H, C_5H_4), 1.41, 1.32 ppm (2 d, $^{3}J_{HH}$ = 6.9 Hz, 2 × 12H, CH*Me*$_2$). ^{13}C{^{1}H} NMR (101 MHz, C_6D_6): δ= 148.6 (*C*CHMe$_2$), 140.8 (C_{ipso} Dipp), 128.0, 124.5 (2 × CH Dipp), 106.6 (C_{ipso} C_5H_4), 67.9, 67.8 (2 × CH C_5H_4), 28.8 (*C*HMe$_2$), 25.9, 24.3 ppm (2 × CH*Me*$_2$). ^{29}Si{^{1}H} NMR (99 MHz, C_6D_6): δ= 115.7 ppm.

Synthesis of 1MesSi(SePh)$_2$

[$SiCl_2$(IPr)] (24 mg, 0.05 mmol) and **1Mes**Li_2 (23 mg, 0.05 mmol) were each dissolved in benzene (5 mL). Both solutions were frozen, then warmed carefully to a slushy state and combined. After reaching room temperature, a solution of diphenyl diselenide (16 mg, 0.05 mmol) in benzene (2 mL) was added to the stirred mixture. After 1 h volatile components were removed under reduced pressure. The residue was extracted with *n*-hexane (3 × 5 mL) and the combined extract filtered through a short Celite pad. The filtrate was reduced to dryness under reduced pressure. IPr and trace amounts of **1Mes**H_2 were removed from the dark yellow residue by sublimation (dynamic vacuum, final bath temperature 110 °C). Yield 27 mg (68 %).

$C_{40}H_{40}N_2FeSe_2Si$ (790.61): calcd. C 60.77, H 5.10, N 3.54 %; found C 60.99, H 5.14, N 3.43 %.

^{1}H NMR (500 MHz, C_6D_6): δ= 7.35 (m, 4H, Ph), 6.86 (m, 2H, Ph), 6.67 (m, 4H, Ph), 6.70 (s, 4H, C_6H_2), 4.62, 3.88 (2 s, 2 × 4H, C_5H_4), 2.60 (s, 12H, *o*-Me); 2.09 ppm (s, 6H, *p*-Me). ^{13}C{^{1}H} NMR (101 MHz, C_6D_6): δ= 142.6, 138.4, 135.9 (3 × C_{ipso} Mes), 134.7 (CH Ph), 130.3 (CH Mes), 128.6 (C_{ipso} Ph), 128.4, 126.8 (2 × CH Ph), 99.5 (C_{ipso} C_5H_4), 69.4, 68.0 (2 × CH C_5H_4), 21.7 (*o*-Me), 20.8 ppm (*p*-Me). ^{29}Si{^{1}H} NMR (99 MHz, C_6D_6): δ= –27.5 ppm. ^{77}Se{^{1}H} NMR (95 MHz, C_6D_6): δ= 143.0 ppm.

Synthesis of 1DippSi(SePh)$_2$

C_6D_6 (0.7 mL) was added to **1DippSi** (34 mg, 0.06 mmol) and diphenyl diselenide (20 mg, 0.06 mmol) in an NMR tube. After 12 h the solution was subjected to NMR spectroscopic analysis, which revealed essentially quantitative reaction. Slow evaporation of the solvent afforded the product as a yellow crystalline solid. Trace amounts of residual diphenyl

diselenide were removed by sublimation (dynamic vacuum, final bath temperature 110 °C). Yield 26 mg (50 %).

$C_{46}H_{52}N_2FeSe_2Si$ (874.77): calcd. C 63.16, H 5.99, N 3.20 %; found C 63.20, H 6.14, N 2.99 %.

^{1}H NMR (500 MHz, C_6D_6): δ = 7.19 (s, 6H, C_6H_3), 6.95 (m, 4H, Ph), 6.81 (m, 2H, Ph), 6.72 (m, 4H, Ph), 4.73 (s, 4H, C_5H_4), 4.18 (sept, $^3J_{HH}$ = 6.6 Hz, 4H, C*H*Me$_2$), 3.89 (s, 4H, C_5H_4), 1.48, 1.26 ppm (2 d, $^3J_{HH}$ = 6.6 Hz, 2 × 12H, CH*Me*$_2$). ^{13}C{^{1}H} NMR (101 MHz, C_6D_6): δ = 149.1 (*C*CHMe$_2$), 142.6 (C$_{ipso}$ Dipp), 135.6 (CH Ph), 131.7 (C$_{ipso}$ Ph), 128.7 (CH Ph), 127.9 (CH Dipp), 126.8 (CH Ph), 125.7 (CH Dipp), 101.3 (C$_{ipso}$ C_5H_4), 69.5, 68.2 (2 × CH C_5H_4), 29.1 (*C*HMe$_2$), 27.4, 25.5 ppm (2 × CH*Me*$_2$). ^{29}Si{^{1}H} NMR (99 MHz, C_6D_6): δ = –29.0 ppm. ^{77}Se{^{1}H} NMR (95 MHz, C_6D_6): δ = 149.3 ppm.

Synthesis of (1DippSiO$_2$)$_2$C

The reaction was performed in an NMR tube sealed to a ground-glass joint. A solution of **1DippSi** (11 mg, 0.02 mmol) in C_6D_6 (0.8 mL) was frozen with liquid dinitrogen and the NMR tube evacuated. Carbon dioxide (1.7 mL, 0.07 mmol) was introduced and the solution warmed to ambient temperature. Slow evaporation of the solvent afforded the product as a pale yellow crystalline solid. Yield 6 mg (51 %).

$C_{69}H_{84}N_4Fe_2O_4Si_2$ (1201.29): calcd. C 68.99, H 7.05, N 4.66 %; found C 68.37, H 7.06, N 4.23 %.

^{1}H NMR (500 MHz, C_6D_6): δ = 7.11 (m, 4H, C_6H_3), 7.05 (m, 8H, C_6H_3), 4.33, 3.70 (2 s, 2 × 8H, C_5H_4), 3.70 (sept, $^3J_{HH}$ = 6.7 Hz, 8H, C*H*Me$_2$), 1.25, 1.12 ppm (2 d, $^3J_{HH}$ = 6.7 Hz, 2 × 24H, CH*Me*$_2$). ^{13}C{^{1}H} NMR (101 MHz, C_6D_6): δ = 147.8 (*C*CHMe$_2$), 139.5 (C$_{ipso}$ Dipp), 128.4 (CH Dipp), 127.6 (CO_4), 125.1 (CH Dipp), 100.4 (C$_{ipso}$ C_5H_4), 68.2, 68.1 (2 × CH C_5H_4), 28.7 (*C*HMe$_2$), 26.0, 25.3 ppm (2 × CH*Me*$_2$). ^{29}Si{^{1}H} NMR (99 MHz, C_6D_6): δ = –57.9 ppm.

Synthesis of (1DippSiO)$_2$

The reaction was performed in an NMR tube sealed to a ground-glass joint. A solution of **1DippSi** (11 mg, 0.02 mmol) in C_6D_6 (0.8 mL) was frozen with liquid dinitrogen and the NMR tube evacuated. Nitrous oxide (1.7 mL, 0.07 mmol) was introduced and the solution warmed to ambient temperature. Slow evaporation of the solvent afforded the product as a pale yellow crystalline solid. Yield 6 mg (53 %).

$C_{68}H_{84}N_4Fe_2O_2Si_2$ (1157.28): calcd. C 70.57, H 7.31, N 4.84 %; found C 70.67, H 7.32, N 4.61 %.

^{1}H NMR (500 MHz, C_6D_6): δ = 7.14 (m, 4H, C_6H_3), 7.01 (m, 4H, C_6H_3), 6.84 (m, 4H, C_6H_3), 4.32 (s, 4H, C_5H_4), 4.23 (sept, $^3J_{HH}$ = 6.6 Hz, 4H, C*H*Me2), 3.77, 3.65, 3.53 (3 s, 3 × 4H, C_5H_4), 3.16 (sept, $^3J_{HH}$ = 6.7 Hz, 4H, C*H*Me2), 1.80, (d, $^3J_{HH}$ = 6.6 Hz, 12H, CH*Me*2), 1.55 (d, $^3J_{HH}$ = 6.7 Hz, 12H, CH*Me*2), 0.64 (d, $^3J_{HH}$ = 6.6 Hz, 12H, CH*Me*2), 0.34 ppm (d, $^3J_{HH}$ = 6.7 Hz, 12H, CH*Me*2). ^{13}C{^{1}H} NMR (101 MHz, C_6D_6): δ = 148.4, 145.8 (2 × *C*CHMe2), 145.2 (C_{ipso} Dipp), 128.4, 127.2, 126.8, 125.5 (4 × CH Dipp), 108.0 (C_{ipso} C_5H_4), 71.3, 68.0, 67.6, 64.5 (4 × CH C_5H_4), 29.2, 29.0 (2 × *C*HMe2), 27.2 (two closely spaced signals), 26.2, 25.4 ppm (4 × CH*Me*2). ^{29}Si{^{1}H} NMR (99 MHz, C_6D_6): δ = −58.6 ppm.

Synthesis of 1DippSi(H)OH

The product is formed when benzene solutions of **1DippSi** are exposed to an inert gas atmosphere containing trace amounts of water, which may happen simply by serendipity. The following procedure, which was originally intended to be used for the reaction of the silylene with carbon suboxide, was found to afford the water addition product conveniently in reproducible yields. A 100 mL flask was charged with a mixture of malonic acid (150 mg, 1.44 mmol) and phosphorus pentoxide (1.50 g, 5.3 mmol) and connected with a glass elbow to an empty 100 mL Schlenk flask. The apparatus was connected to the Schlenk line and evacuated for ca. 20 min. The reaction flask was subsequently immersed in a heating bath kept at 140 °C for ca. 10 min under static vacuum. The resulting gaseous products were collected by condensation into the Schlenk flask cooled with liquid dinitrogen. This flask was disconnected from the reaction flask and the liquid dinitrogen bath replaced by an ethanol/liquid dinitrogen cooling bath (−110 °C), which was allowed to warm up to ambient temperature over the course of 4 h under ambient pressure (i. e. stopcock valve open to the Schlenk line). It was subsequently connected with a glass elbow to a 100 mL Schlenk flask containing a frozen solution of **1DippSi** (28 mg, 0.05 mmol) in toluene (7 mL), which was immersed in a liquid dinitrogen bath after closing the stopcock valve. The bath was removed and the apparatus allowed to warm up to ambient temperature under ambient pressure (i. e. stopcock valve open to the Schlenk line). The two flasks were disconnected. The solvent was removed under reduced pressure and the residue taken up in benzene. Slow evaporation of the solvent afforded the product as a yellow crystalline solid. Yield 18 mg (62 %).

$C_{34}H_{44}N_2FeOSi$ (580.66): calcd. C 70.33, H 7.64, N 4.82 %; found C 70.22, H 7.44, N 4.40 %.

^{1}H NMR (500 MHz, C_6D_6): δ = 7.10 (m, 6H, C_6H_3), 5.27 (s, 1H, SiH); 4.58, 4.52 (2 s, 2 × 2H, C_5H_4), 4.13, 4.03 (2 sept, $^{3}J_{HH}$ = 6.8 Hz, 2 × 2H, C*H*Me$_2$), 3.90 (s, 4H, C_5H_4), 1.56 (s, 1H, OH), 1.39, 1.30 ppm (2 m, 2 × 12H, m, CHMe$_2$). ^{13}C{^{1}H} NMR (101 MHz, C_6D_6): δ = 149.8, 148.7 (2 × *C*CHMe$_2$), 140.7 (C$_{ipso}$ Dipp), 127.5, 124.9, 124.7 (3 × CH Dipp), 101.4 (C$_{ipso}$ C_5H_4), 69.9, 68.0, 67.8, 67.5 (4 × CH C_5H_4), 28.6, 28.4, 25.9, 25.8 (4 × CH*Me*$_2$), 25.4 ppm (two closely spaced signals, 2 × *C*HMe$_2$). ^{29}Si{^{1}H} NMR (99 MHz, C_6D_6): δ = –45.7 ppm.

Synthesis of 1DippSi(H)NH$_2$

The reaction was performed in an NMR tube sealed to a ground-glass joint. A solution of **1DippSi** (17 mg, 0.03 mmol) in C_6D_6 (0.8 mL) was frozen with liquid dinitrogen and the NMR tube evacuated. Ammonia (1.0 mL, 0.04 mmol) was introduced and the solution warmed to ambient temperature. Slow evaporation of the solvent afforded the product as a yellow crystalline solid. Yield 12 mg (69 %).

$C_{34}H_{45}N_3FeSi$ (579.67): calcd. C 70.45, H 7.83, N 7.25 %; found C 70.33, H 7.95, N 7.01 %.

^{1}H NMR (500 MHz, C_6D_6): δ = 7.13 (m, 6H, C_6H_3), 5.76 (t, $^{3}J_{HH}$ = 3.5 Hz, 1H, SiH), 4.58, 4.49 (2 s, 2 × 2H, C_5H_4), 4.12, 4.05 (2 sept, $^{3}J_{HH}$ = 6.9 Hz, 2 × 2H, C*H*Me$_2$), 3.92, 3.90 (2 s, 2 × 2H, C_5H_4), 1.38, 1.35, 1.34, 1.27 (4 d, $^{3}J_{HH}$ = 6.9 Hz, 4 × 6H, CH*Me*$_2$), 0.37 ppm (br. s, 2H, NH$_2$). ^{13}C{^{1}H} NMR (101 MHz, C_6D_6): δ = 148.9, 148.7 (2 × *C*CHMe$_2$), 142.6 (C$_{ipso}$ Dipp), 128.6, 127.1, 125.0, 124.5 (4 × CH Dipp), 101.0 (C$_{ipso}$ C_5H_4), 71.0, 67.8, 67.4, 66.3 (4 × CH C_5H_4), 28.8, 28.1 (2 × *C*HMe$_2$), 26.1, 26.0, 25.9, 25.0 ppm (4 × CH*Me*$_2$). ^{29}Si{^{1}H} NMR (99 MHz, C_6D_6): δ = –42.4 ppm.

Synthesis of 1DippSi(H)(PHFc)

A suspension of ferrocenylphosphine (11 mg, 0.05 mmol) in *n*-hexane (4 mL) was added to a stirred solution of **1DippSi** (28 mg, 0.05 mmol) in toluene (4 mL). After 12 h volatile components were removed under reduced pressure. The residue was taken up in a minimal amount of benzene. Slow evaporation of the solvent afforded the product as a yellow crystalline solid. Yield 27 mg (69 %).

$C_{44}H_{53}N_2Fe_2PSi$ (780.66): calcd. C 67.70, H 6.84, N 3.59 %; found C 67.14, H 6.94, N 3.66 %.

^{1}H NMR (500 MHz, C_6D_6): δ = 7.24 (m, 1H, C_6H_3), 7.15, 7.11 (2 m, 2 × 2H, C_6H_3), 6.98 (m, 1H, C_6H_3), 6.49 (d, $^{2}J_{PH}$ = 11.9 Hz, 1H, SiH); 4.65 (s, 2H, C_5H_4), 4.60, 4.57 (2 m, 2 × 1H, C_5H_4), 4.28, 4.20, 4.04 (3 sept, $^{3}J_{HH}$ = 6.7 Hz, 3 × 1H, C*H*Me$_2$), 3.90 (m, 6H, C_5H_4), 3.80 (s,

1H, C_5H_4), 3.74 (sept, $^3J_{HH}$ = 6.7 Hz, 1H, C*H*Me$_2$), 3.70 (s, 5H, Cp), 3.08 (s, 1H, C_5H_4), 2.80 (d, $^1J_{PH}$ = 210.0 Hz, 1H, PH), 1.81, 1.70, 1.49, 1.44, 1.42, 1.22, 1.08, 0.94 ppm (8 d, $^3J_{HH}$ = 6.7 Hz, 8 × 3H, CH*Me*$_2$). ^{13}C{^{1}H} NMR (101 MHz, C_6D_6): δ = 149.8, 149.3, 148.9, 148.6 (4 × *C*CHMe$_2$), 142.6, 141.7 (2 × C_{ipso} Dipp), 127.6, 127.3, 125.4, 125.1, 124.9, 124.6 (6 × CH Dipp), 102.5, 102.4 (2 × C_{ipso} C_5H_4), 75.7 (d, J_{PC} = 34.7 Hz, CH C_5H_4), 74.0 (d, J_{PC} = 9.4 Hz, CH C_5H_4), 72.0, 71.4 (2 × CH C_5H_4), 70.7 (d, J_{PC} = 8.2 Hz, CH C_5H_4), 70.0 (CH, C_5H_4), 69.6 (Cp), 67.8 (d, J_{PC} = 13.5 Hz, CH C_5H_4), 67.3 (d, J_{PC} = 8.2 Hz, CH C_5H_4), 66.5 (d, J_{PC} = 10.8 Hz, C_{ipso} Fc), 65.7, 65.1 (2 × CH C_5H_4), 28.9 (two closely spaced signals), 28.3 (3 × *C*HMe$_2$), 28.5 (d, J_{PC} = 3.1 Hz, *C*HMe$_2$), 27.1, 26.6, 26.2, 25.5 (two closely spaced signals), 24.7 (6 × CH*Me*$_2$), 26.4 ppm (d, J_{PC} = 9.4 Hz, CH*Me*$_2$), 25.6 (d, J_{PC} = 10.9 Hz, CH*Me*$_2$). ^{29}Si NMR (99 MHz, C_6D_6): δ = –18.6 ppm (ddd, $^1J_{SiH}$ = 235 Hz, $^1J_{PSi}$ = 11 Hz, $^2J_{SiH}$ = 5 Hz). ^{31}P{^{1}H} NMR (202 MHz, C_6D_6): δ = –159.2 ppm.

Synthesis of 1DippSi(H)(BH_2NH_3)

Ammonia-borane (1.2 mg, 0.04 mmol) was added to a solution of **1DippSi** (21.9 mg, 0.039 mmol) in toluene (5 mL). The mixture was stirred for 12 h. Volatile components were removed under reduced pressure. The residue was taken up in benzene (1 mL). Slow evaporation of the solvent afforded the product as a pale yellow crystalline solid. Yield 12.7 mg (55 %).

$C_{34}H_{48}N_3BFeSi$ (593.51): calcd. C 68.81, H 8.15, N 7.08 %; found C 68.86, H 8.33, N 6.24 %.

^{1}H NMR (500 MHz, C_6D_6): δ = 7.12, 7.06, 7.00 (3 m, 3 × 2H, C_6H_3), 6.28 (br. s, 1H, SiH), 4.83, 4.63 (2 s, 2 × 2H, C_5H_4), 4.37, 4.24 (2 sept, $^3J_{HH}$ = 6.8 Hz, 2 × 2H, C*H*Me$_2$), 4.03, 3.95 (2 m, 2 × 2H, C_5H_4), 1.55, 1.45, 1.32, 1.23 (4 d, $^3J_{HH}$ = 6.8 Hz, 4 × 6H, CH*Me*$_2$), 0.89 ppm (t, $^3J_{HH}$ = 7.0 Hz, 3H, NH_3). ^{13}C{^{1}H} NMR (101 MHz, C_6D_6): δ = 149.5, 148.9 (2 × *C*CHMe$_2$), 144.7 (C_{ipso} Dipp), 125.9, 124.1, 123.9 (3 × CH Dipp), 105.2 (C_{ipso} C_5H_4), 71.5, 66.9, 65.9, 64.8 (4 × CH C_5H_4), 28.2, 27.6 (2 × *C*HMe$_2$), 26.4, 25.6, 25.3, 25.2 ppm (4 × CH*Me*$_2$). ^{11}B{^{1}H} NMR (160 MHz): δ = –23.2 ppm. Signals due to boron-bonded Si and H atoms could not be detected, as has been noted before for related compounds such as, for example, $(Me_3Si)_3SiBH_2NEt_3$.[S4]

B X-Ray Crystallography

For each data collection a single crystal was mounted on a micro-mount and all geometric and intensity data were taken from this sample. Data collections were carried out at 100(2) K using MoK_α radiation (λ = 0.71073 Å) either on a Stoe IPDS2 diffractometer equipped with a 2-circle goniometer and an area detector, or on a Stoe StadiVari diffractometer equipped with a 4-circle goniometer and a DECTRIS Pilatus 200K detector. The data sets were corrected for absorption, Lorentz and polarisation effects. The structures were solved by direct methods (SHELXT) and refined using alternating cycles of least-squares refinements against F^2 (SHELXL2014/7).[S5] C-bonded H atoms were included to the models in calculated positions, heteroatom-bonded H atoms have been found in the difference Fourier lists. All H atoms were treated with the 1.2 fold or 1.5 fold isotropic displacement parameter of their bonding partner. Experimental details for each diffraction experiment are given in Table S1. CCDC 2025512 – 2025520 contain the supplementary crystallographic data for this paper. These data are provided free of charge by The Cambridge Crystallographic Data Centre.

Table S1. X-ray crystallographic details.

	**1DippSi**·C_6H_6	**1DippSi**(H)OH	**1DippSi**(H)NH_2·C_6H_6	**1DippSi**(H)PHFc	**1DippSi**(H)(BH_2NH_3) C_6H_6	**1MesSi**($SePh)_2$	**1DippSi**($SePh)_2$·½C_6H_6	(**1DippSi**O$)_2$·3C_6H_6	(**1DippSi**$O_2)_2$C·2C_6H_6
Chemical formula	$C_{40}H_{48}FeN_2Si$	$C_{34}H_{44}FeN_2OSi$	$C_{40}H_{51}FeN_3Si$	$C_{44}H_{53}Fe_2N_2PSi$	$C_{40}H_{54}BFeN_3Si$	$C_{40}H_{40}FeN_2Se_2Si$	$C_{49}H_{55}FeN_2Se_2Si$	$C_{86}H_{102}Fe_2N_4O_2Si_2$	$C_{81}H_{96}Fe_2N_4O_4Si_2$
Formula mass	640.74	580.65	657.77	780.64	671.61	790.60	913.81	1391.59	1357.49
Crystal system	triclinic	monoclinic	triclinic	orthorhombic	monoclinic	monoclinic	monoclinic	monoclinic	monoclinic
Space group	*P*-1	$P2_1/n$	*P*-1	$P2_12_12_1$	$P2_1/n$	$P2_1/c$	$P2_1/n$	$C2/c$	$P2_1/n$
a/Å	9.6664(3)	14.7950(5)	9.9324(7)	21.6987(6)	9.8394(2)	11.0243(4)	17.9177(10)	18.6690(9)	15.3561(7)
b/Å	9.8648(3)	9.9505(4)	11.8878(8)	10.6299(3)	29.5924(7)	8.4164(2)	11.7160(5)	20.9176(8)	19.8360(12)
c/Å	19.0474(6)	20.9936(7)	15.0050(8)	16.7015(6)	12.7890(3)	37.7848(14)	21.8288(13)	18.4822(9)	23.5810(12)
α/°	97.704(2)	90	93.880(5)	90	90	90	90	90	90
β/°	103.986(2)	106.856(2)	96.851(5)	90	103.655(2)	97.041(3)	114.131(4)	96.024(4)	101.838(4)
γ/°	99.817(2)	90	99.294(5)	90	90	90	90	90	90
U/Å^3	1707.23(9)	2957.8(2)	1729.1(2)	3852.3(2)	3618.54(14)	3479.4(2)	4181.9(4)	7177.6(6)	7030.1(6)
Z	2	4	2	4	4	4	4	4	4
μ/mm^{-1}	4.094	0.580	0.503	0.860	0.482	2.592	2.167	0.490	4.051
F(000)	684	1240	704	1648	1440	1608	1884	2968	2888
Crystal size/mm^3	0.29 × 0.22 × 0.15	0.18 × 0.15 × 0.01	0.53 × 0.32 × 0.21	0.13 × 0.06 × 0.05	0.26 × 0.22 × 0.21	0.28 × 0.16 × 0.05	0.22 × 0.21 × 0.06	0.17 × 0.15 × 0.04	0.17 × 0.08 × 0.06
Absorption correction	multi-scan	integration	integration	multi-scan	multi-scan	integration	integration	integration	integration
T_{min}/T_{max}	0.2341/0.5229	0.9224/0.9927	0.7670/0.9100	0.8034/0.9583	0.5818/0.8771	0.6331/0.8916	0.6049/0.6882	0.9167/0.9814	0.6193/0.8100
θ range/°	4.628 – 70.974	1.500 – 25.661	1.743 – 25.993	1.539 – 25.989	1.777 – 26.000	1.861 – 25.499	1.247 – 25.500	1.767 – 26.999	2.938 – 68.496
Reflections measured	13805	13419	11494	12751	27029	15636	28744	15666	29515
Independent reflections	6303	5557	6740	7550	7107	6462	7778	7722	12559
[R_{int}]	[0.0151]	[0.0201]	[0.0488]	[0.0550]	[0.0175]	[0.0450]	[0.0272]	[0.0468]	[0.0702]
Parameters	405	366	417	465	427	431	504	443	854
Final R_1 (wR_2) [$I > 2\sigma(I)$]	0.0295 (0.0785)	0.0330 (0.0788)	0.0625 (0.1600)	0.0538 (0.1152)	0.0338 (0.0850)	0.0516 (0.1328)	0.0282 (0.0730)	0.0423 (0.0967)	0.0797 (0.2080)
Final R_1 (wR_2) [all data]	0.0309 (0.0796)	0.0404 (0.0876)	0.0800 (0.1725)	0.0843 (0.1561)	0.0404 (0.0919)	0.0620 (0.1408)	0.0345 (0.0782)	0.0676 (0.1093)	0.1142 (0.2475)
Goodness of fit	1.052	1.046	1.074	1.116	1.061	1.042	0.818	1.010	1.064
Residual electron density/eÅ^{-3}	−0.530 – 0.260	−0.303 – 0.801	−0.826 – 1.506	−1.347 – 1.140	−0.313 – 0.497	−0.668 – 1.959	−0.335 – 0.492	−0.497 – 0.433	−0.927-– 0.877
CCDC No.	2025512	2025513	2025514	2025515	2025516	2025517	2025518	2025519	2025520

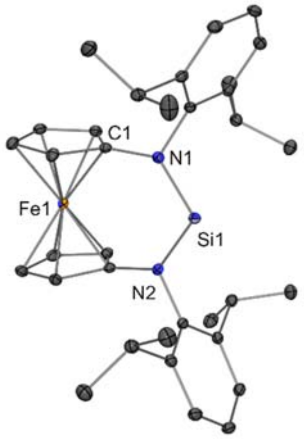

Figure S1. Molecular structure of **1DippSi** (ORTEP plot, 30 % ellipsoids) in the crystal. Hydrogen atoms have been omitted for clarity. Selected bond lengths [Å] and angles [°]: Si1–N1 1.7327(12), Si1–N2 1.7344(12), N1–Si1–N2 106.58(6); sum of angles (Σ∡) at N1 359.9, at N2 360.0

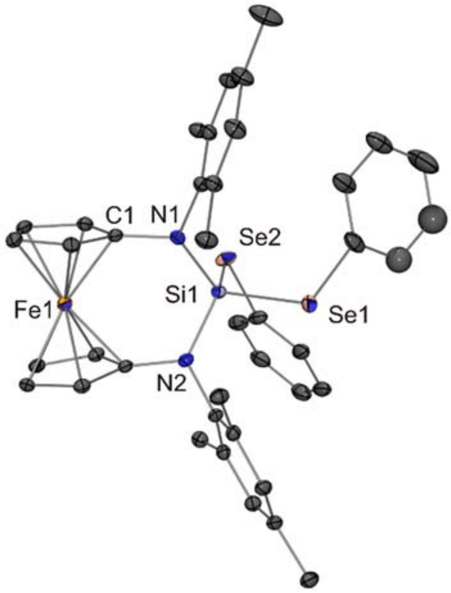

Figure S2. Molecular structure of **1MesSi**(SePh)$_2$ (ORTEP plot, 30 % ellipsoids) in the crystal. Hydrogen atoms have been omitted for clarity. Selected bond lengths [Å] and angles [°]: Si1–N1 1.736(3), Si1–N2 1.739(3), Si1–Se1 2.2915(10), Si1–Se2 2.2773(10), N1–Si1–N2 112.46(14), Se1–Si1–Se2 111.61(4); Σ∡ at N1 359.4, at N2 360.0.

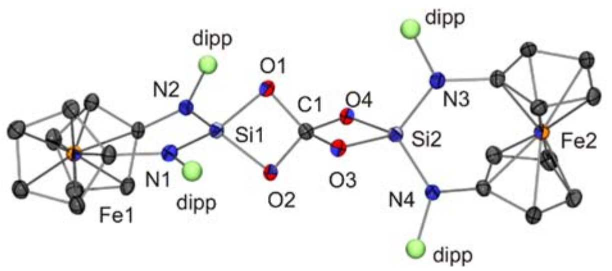

Figure S3. Molecular structure of (**1DippSi**O_2)$_2$C·2C_6H_6 (ORTEP plot, 30 % ellipsoids) in the crystal. Hydrogen atoms and the atoms of the Dipp groups, except the N-bonded C_{ipso} atoms (dipp, shown as circles of arbitrary size in pale green), as well as the solvent molecules have been omitted for clarity. Selected bond lengths [Å] and angles [°]: Si1–N1 1.694(4), Si1–N2 1.700(4), Si1–O1 1.677(3), Si1–O2 1.667(3), Si2–N3 1.713(4), Si2–N4 1.704(4), Si2–O3 1.670(3), Si2–O4 1.681(3), C1–O1 1.402(6), C1–O2 1.420(6), C1–O3 1.403(5), C1–O4 1.402(6), N1–Si1–N2 112.7(2), N3–Si2–N4 112.9(2), O1–Si1–O2 80.7(2), O3–Si2–O4 80.7(2); Σ∡ at N1 359.9, at N2 359.9, at N3 360.0, at N4 360.0.

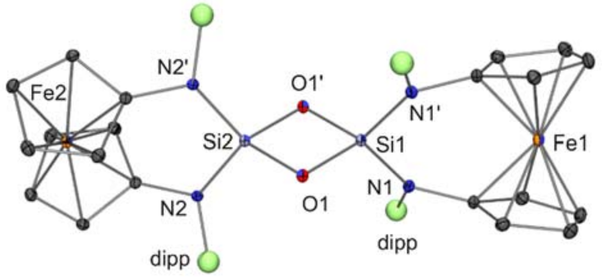

Figure S4. Molecular structure of (**1DippSi**O)$_2$·3C_6H_6 (ORTEP plot, 30 % ellipsoids) in the crystal. Hydrogen atoms and the atoms of the Dipp groups, except for the N-bonded C_{ipso} atoms (dipp, shown as circles of arbitrary size in pale green), as well as solvent molecules have been omitted for clarity. Selected bond lengths [Å] and angles [°]: Si1–N1 1.733(2), Si2–N2 1.740(2), Si1–O1 1.6819(15), Si2–O1 1.6791(15), N1–Si1–N1´ 107.24(12), N2–Si2–N2´ 106.50(12), O1–Si1–O1´ 86.86(11), O1–Si2–O1´ 87.04(10); Σ∡ at N1 359.8, at N2 359.9.

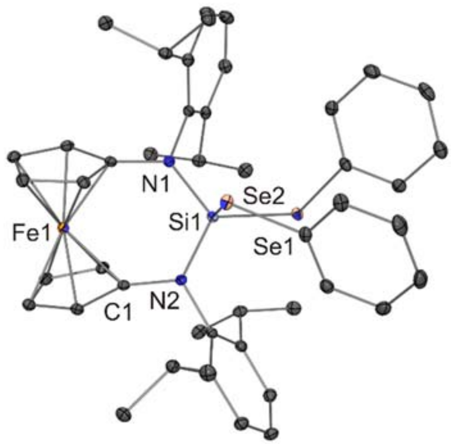

Figure S5. Molecular structure of **1DippSi**(SePh)$_2$·½C_6H_6 (ORTEP plot, 30 % ellipsoids) in the crystal. Hydrogen atoms and the solvent molecule have been omitted for clarity. Selected bond lengths [Å] and angles [°]: Si1–N1 1.745(2), Si1–N2 1.741(2), Si1–Se1 2.2717(6), Si1–Se2 2.2959(6), N1–Si1–N2 109.49(9), Se1–Si1–Se2 107.56(3); Σ∡ at N1 359.4, at N2 360.0.

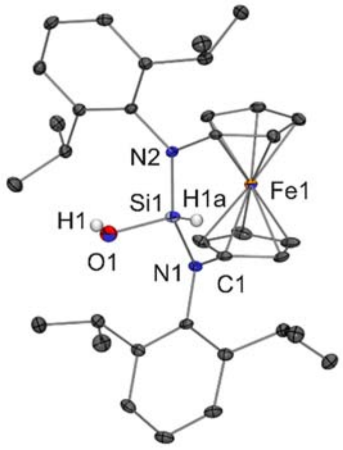

Figure S6. Molecular structure of **1DippSi**(H)(OH) (ORTEP plot, 30 % ellipsoids) in the crystal. C-bonded hydrogen atoms have been omitted for clarity. Selected bond lengths [Å] and angles [°]: Si1–N1 1.7197(16), Si1–N2 1.7199(16), Si1–O1 1.6071(16), N1–Si1–N2 111.49(8); Σ∡ at N1 359.9, at N2 359.4.

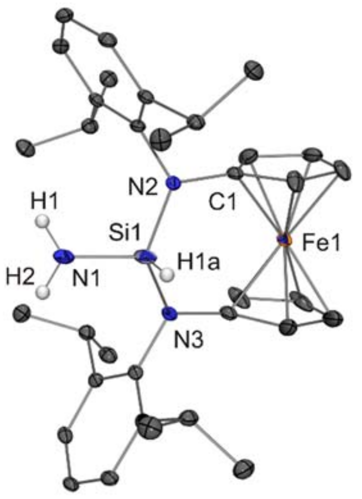

Figure S7. Molecular structure of **1DippSi**(H)(NH_2)·C_6H_6 (ORTEP plot, 30 % ellipsoids) in the crystal. C-bonded hydrogen atoms and the solvent molecule have been omitted for clarity. Selected bond lengths [Å] and angles [°]: Si1–N1 1.691(4), Si1–N2 1.721(3), Si1–N3 1.730(3), N2–Si1–N3 111.72(14); Σ∡ at N2 358.5, at N3 358.0.

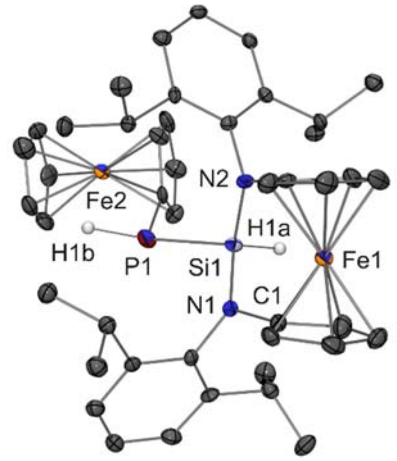

Figure S8. Molecular structure of **1DippSi**(H)(PHFc) (ORTEP plot, 30 % ellipsoids) in the crystal. C-bonded hydrogen atoms have been omitted for clarity. Selected bond lengths [Å] and angles [°]: Si1–N1 1.734(7), Si1–N2 1.721(7), Si1–P1 2.255(4), N1–Si–N2 110.3(3), Si1–P1–C35 104.4(3); Σ∡ at N1 357.9, at N2 357.8.

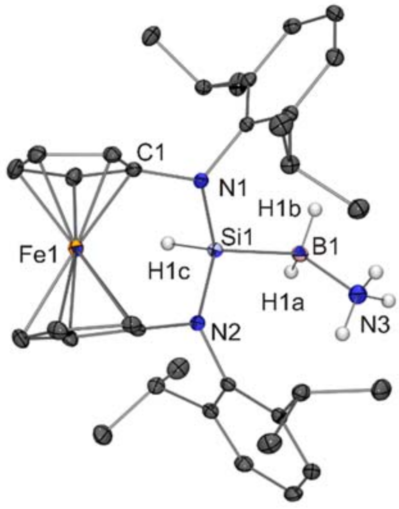

Figure S9. Molecular structure of **1DippSi**(H)(BH_2NH_3) (ORTEP plot, 30 % ellipsoids) in the crystal. C-bonded hydrogen atoms have been omitted for clarity. Selected bond lengths [Å] and angles [°]: Si1–N1 1.7571(14), Si1–N2 1.7694(14), Si1–B1 2.008(2), N3–B1 1.600(3), N1–Si–N2 107.84(7), Si1–B1–N1 116.41(13); Σ∡ at N1 358.3, at N2 356.6

C Plots of NMR Spectra

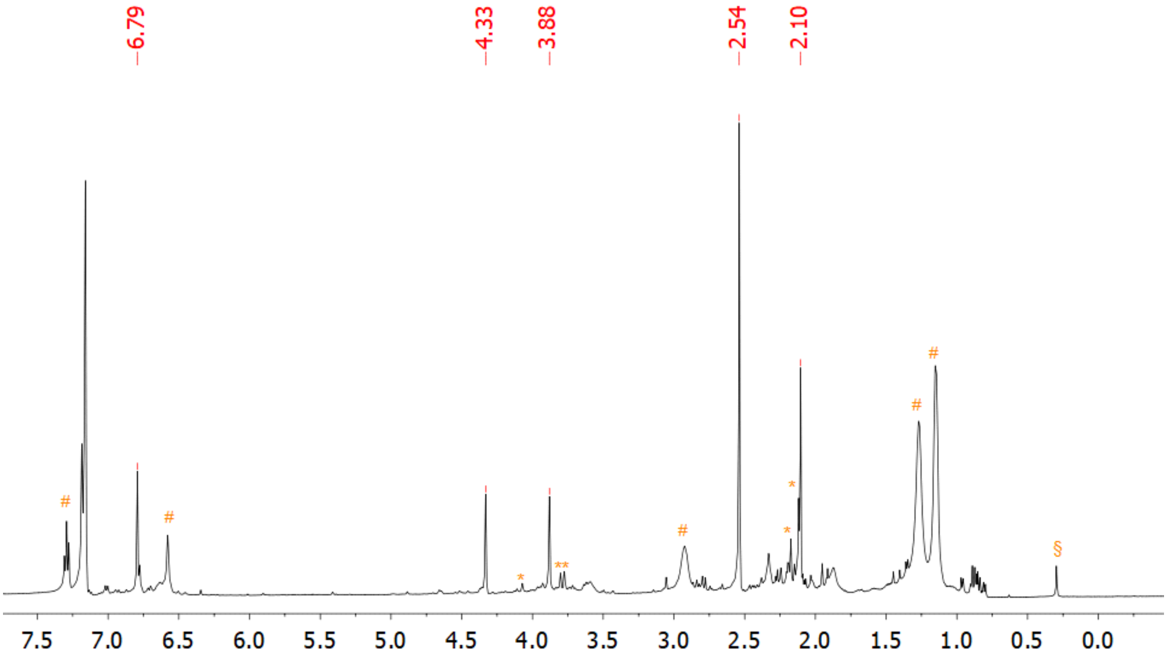

Figure S10. ^{1}H NMR spectrum of **1MesSi** (C_6D_6, 499.7 MHz) generated from **1Mes**Li_2 and [$SiCl_2$(IPr)] in an NMR experiment. Signals marked belong to IPr (#), **1Mes**H_2 (*) and silicon grease (§).

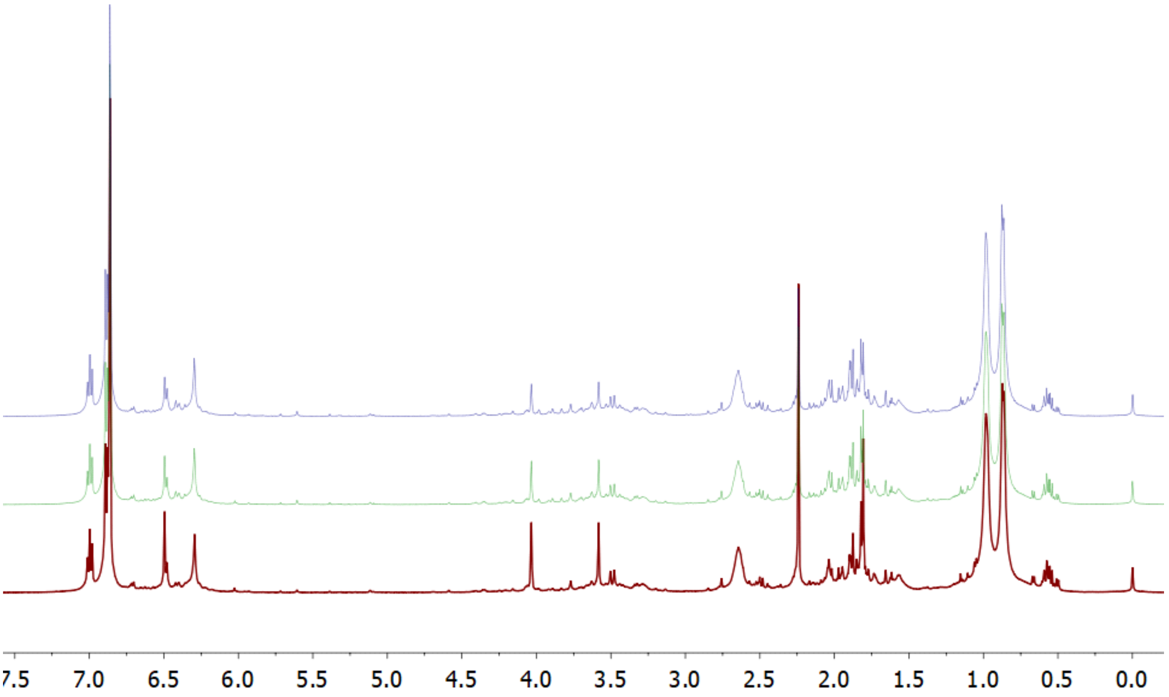

Figure S11. ^{1}H NMR spectra of **1MesSi** (generated from **1Mes**Li_2 and [$SiCl_2$(IPr)] in an NMR experiment) after 15 minutes (red), 1 h (green) and 2.5 h (violett) (C_6D_6, 499.7 MHz).

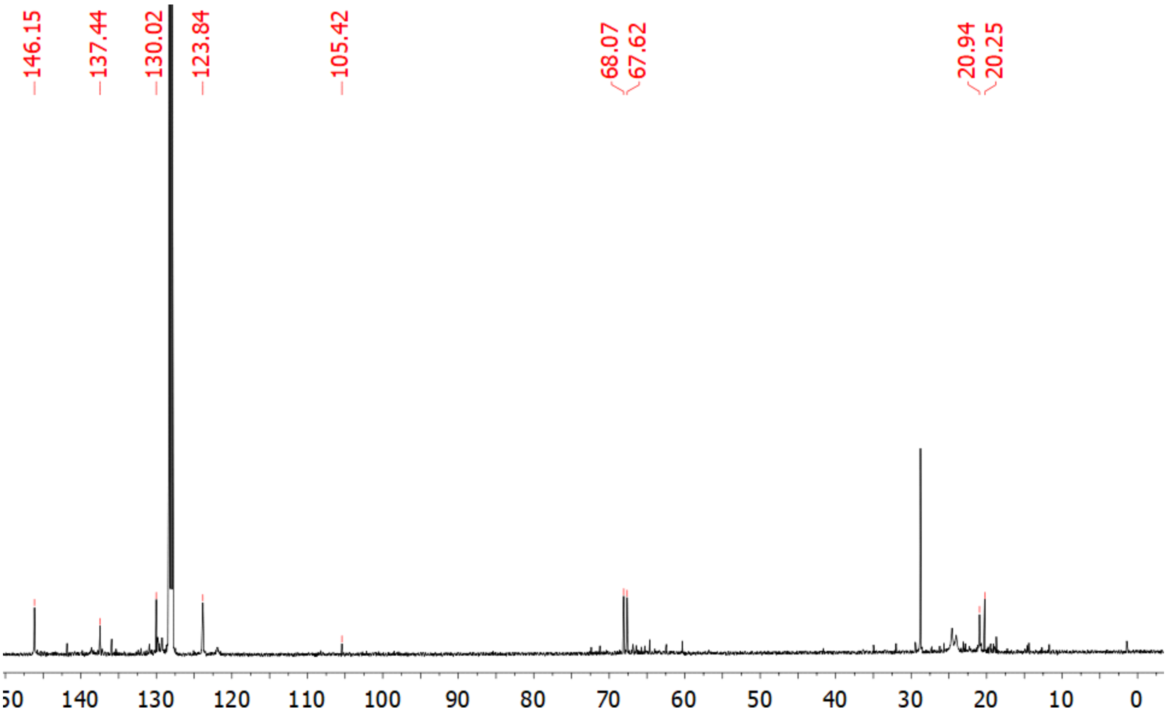

Figure S12. $^{13}C\{^{1}H\}$ NMR spectrum of **1MesSi** (C_6D_6, 100.5 MHz) generated from **1Mes**Li_2 and $[SiCl_2(IPr)]$ in an NMR experiment.

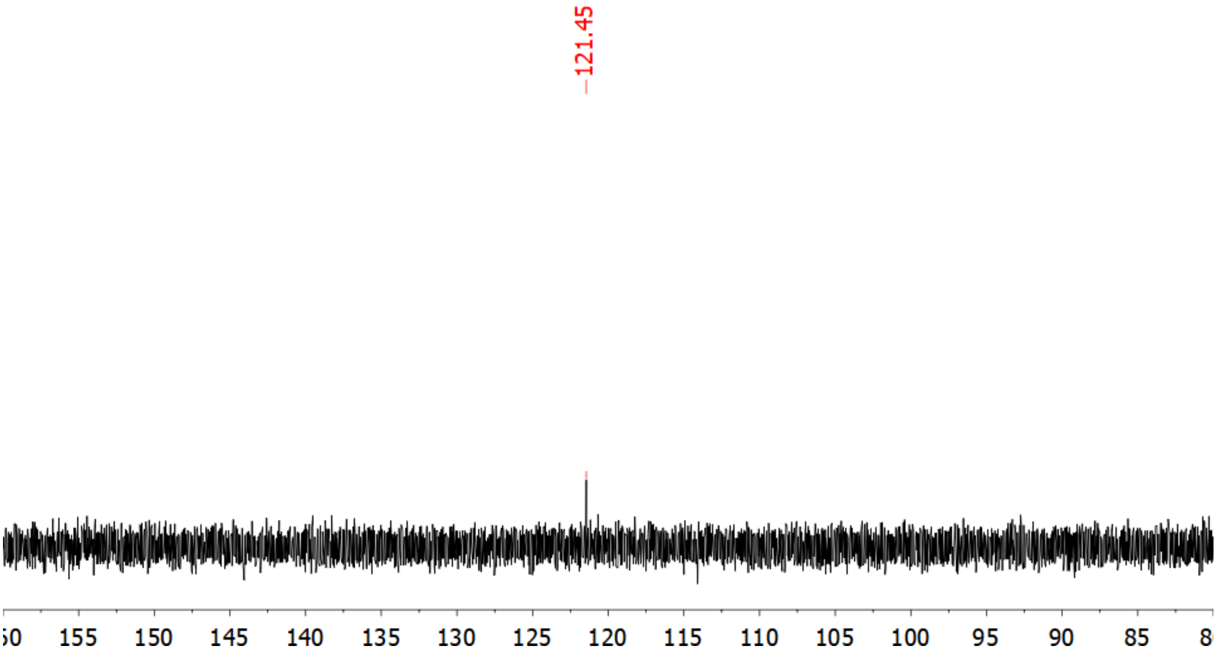

Figure S13. $^{29}Si\{^{1}H\}$ NMR spectrum of **1MesSi** (C_6D_6, 99.3 MHz) generated from **1Mes**Li_2 and $[SiCl_2(IPr)]$ in an NMR experiment.

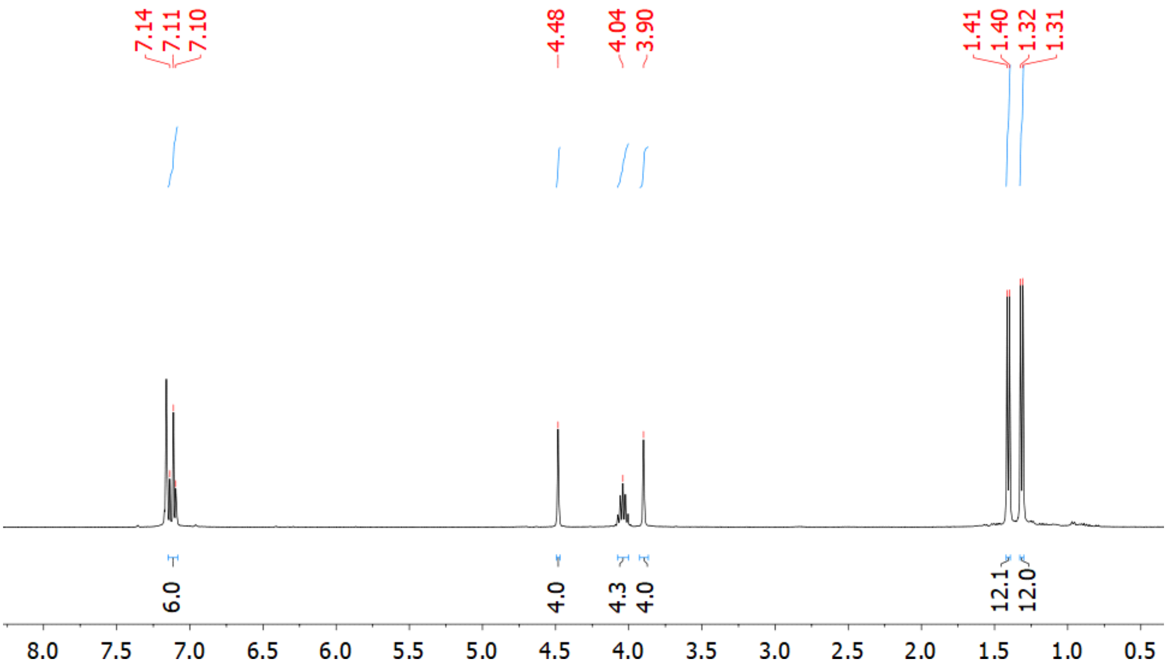

Figure S14. ^{1}H NMR spectrum of **1DippSi** (C_6D_6, 499.7 MHz).

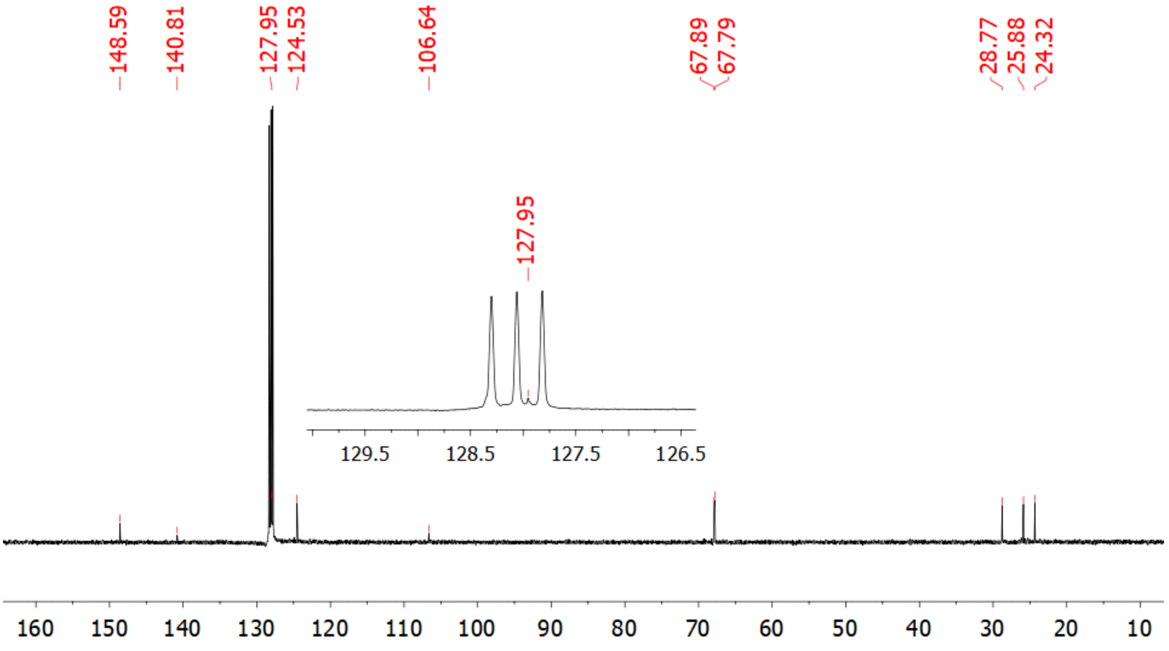

Figure S15. $^{13}C\{^{1}H\}$ NMR spectrum **1DippSi** (C_6D_6, 100.5 MHz).

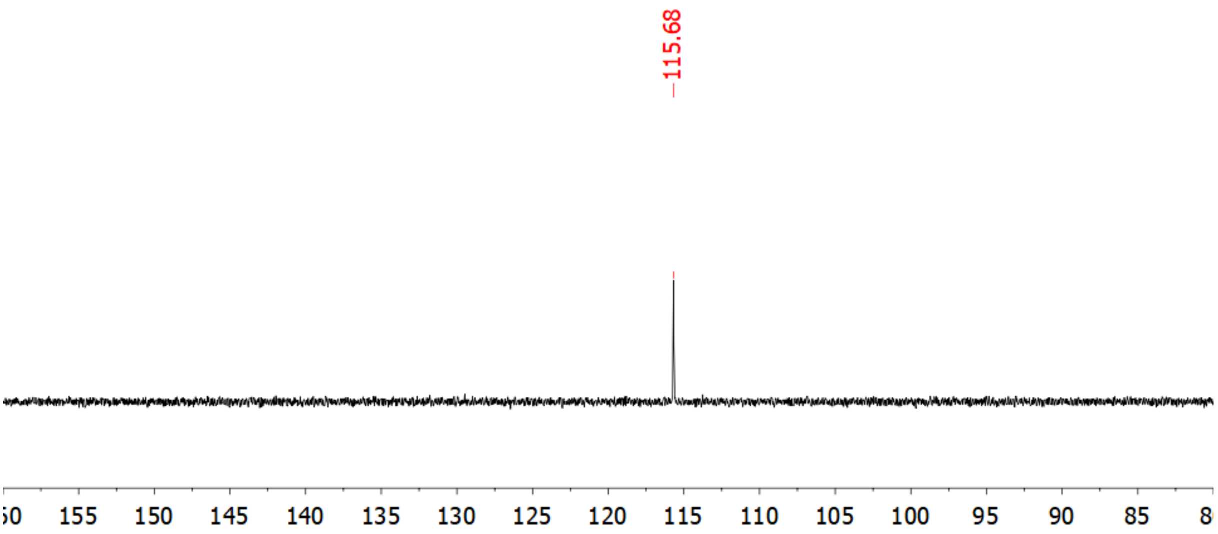

Figure S16. $^{29}Si\{^1H\}$ NMR spectrum of **1DippSi** (C_6D_6, 99.3 MHz).

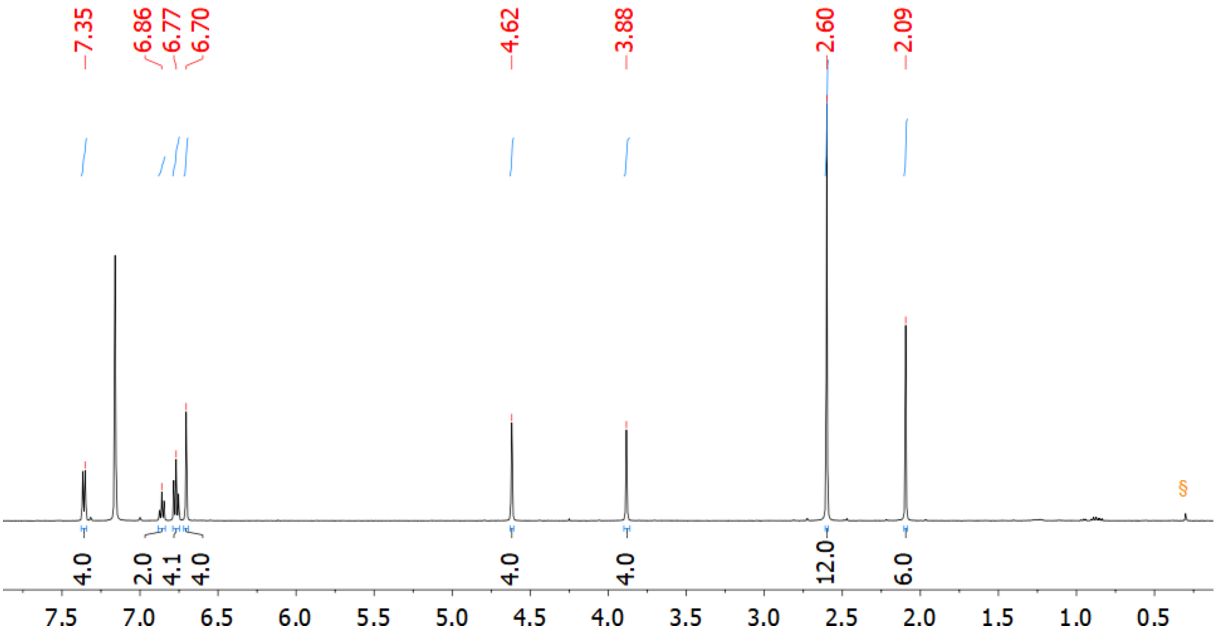

Figure S17. ^1H NMR spectrum of **1MesSi**$(SePh)_2$ (C_6D_6, 499.7 MHz). The signal marked belongs to silicon grease (§).

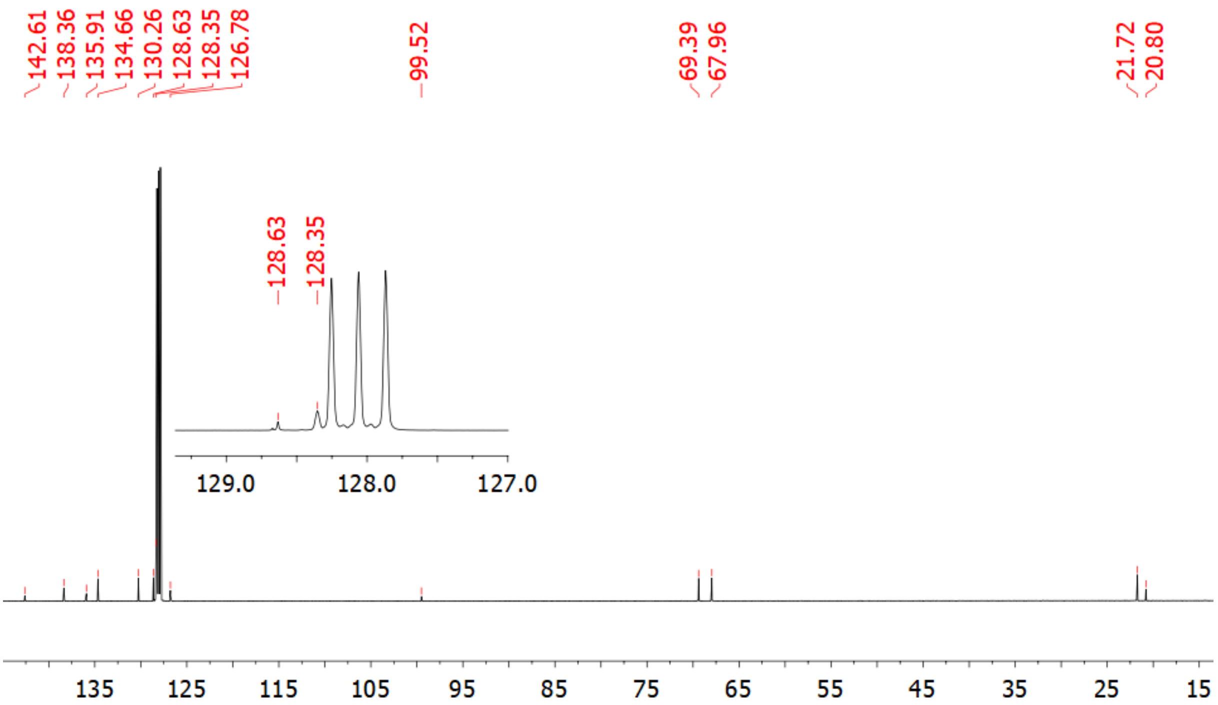

Figure S18. $^{13}C\{^{1}H\}$ NMR spectrum of **1MesSi**(SePh)$_2$ (C_6D_6, 100.5 MHz).

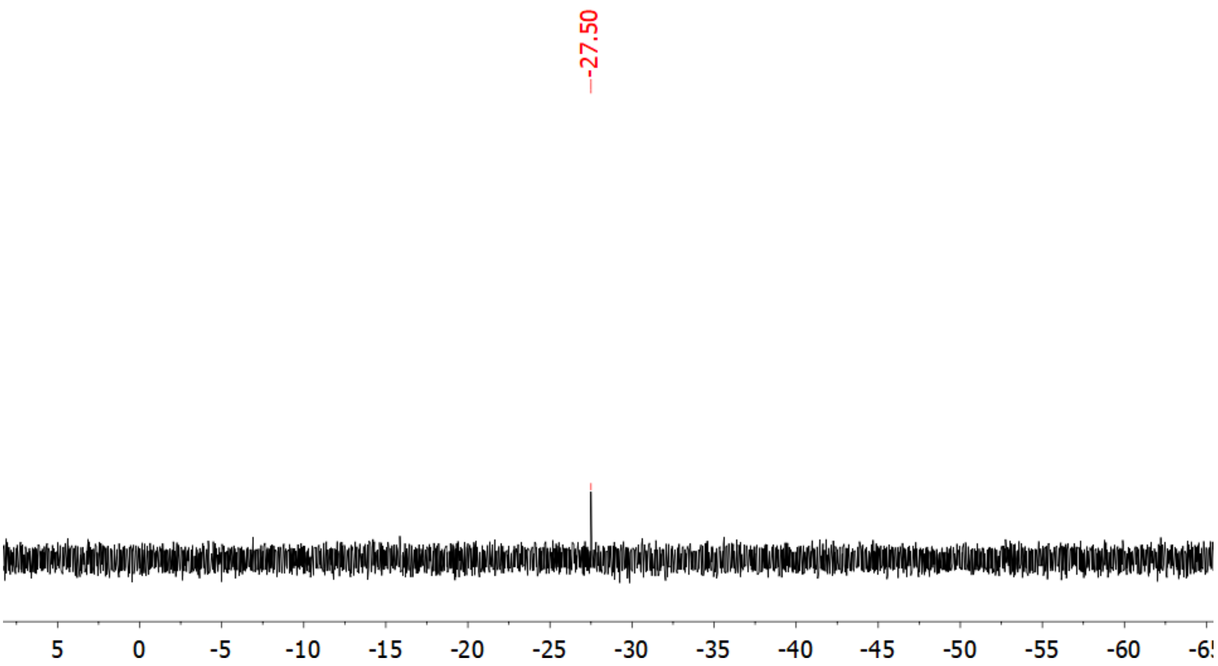

Figure S19. $^{29}Si\{^{1}H\}$ NMR spectrum of **1MesSi**(SePh)$_2$ (C_6D_6, 99.3 MHz).

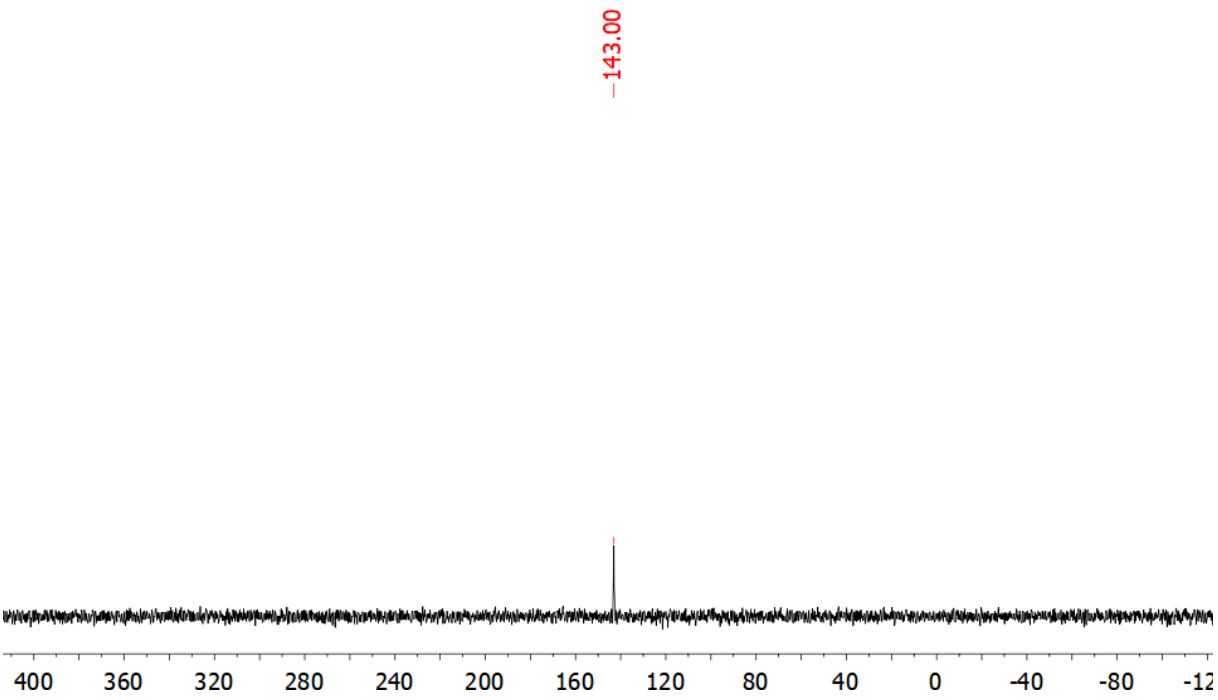

Figure S20. $^{77}Se\{^1H\}$ NMR spectrum of **1MesSi**$(SePh)_2$ (C_6D_6, 95.3 MHz).

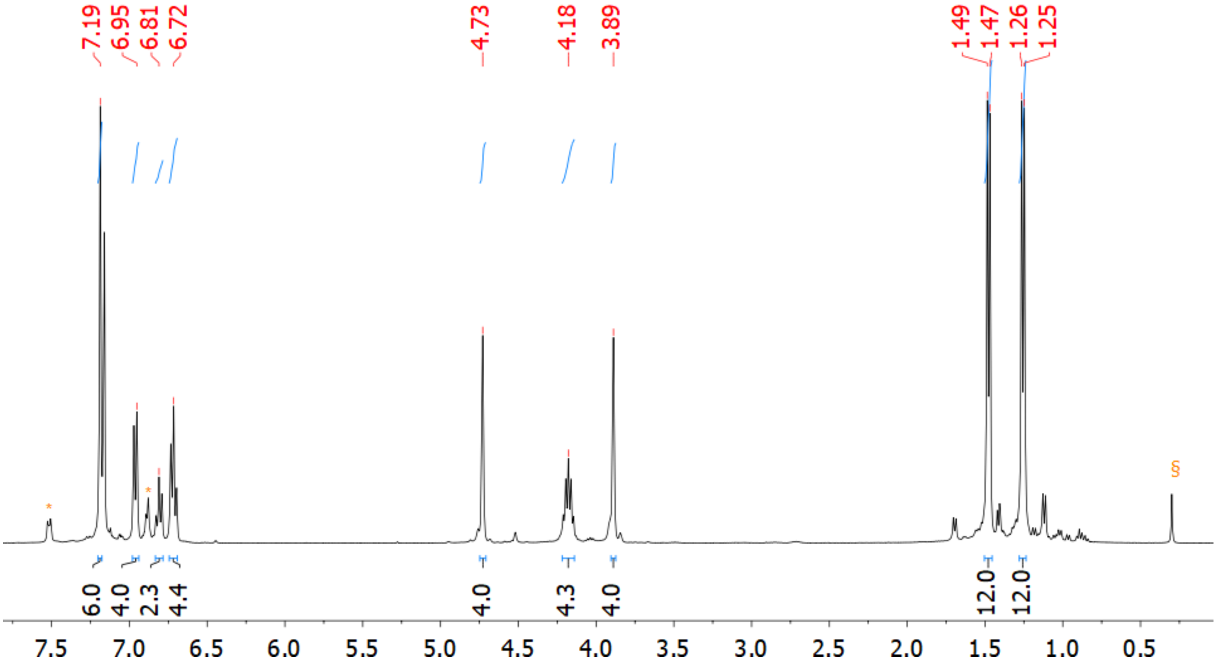

Figure S21. ^1H NMR spectrum of **1DippSi**$(SePh)_2$ (C_6D_6, 499.7 MHz). Signals marked belong to Se_2Ph_2 (*) and silicon grease (§).

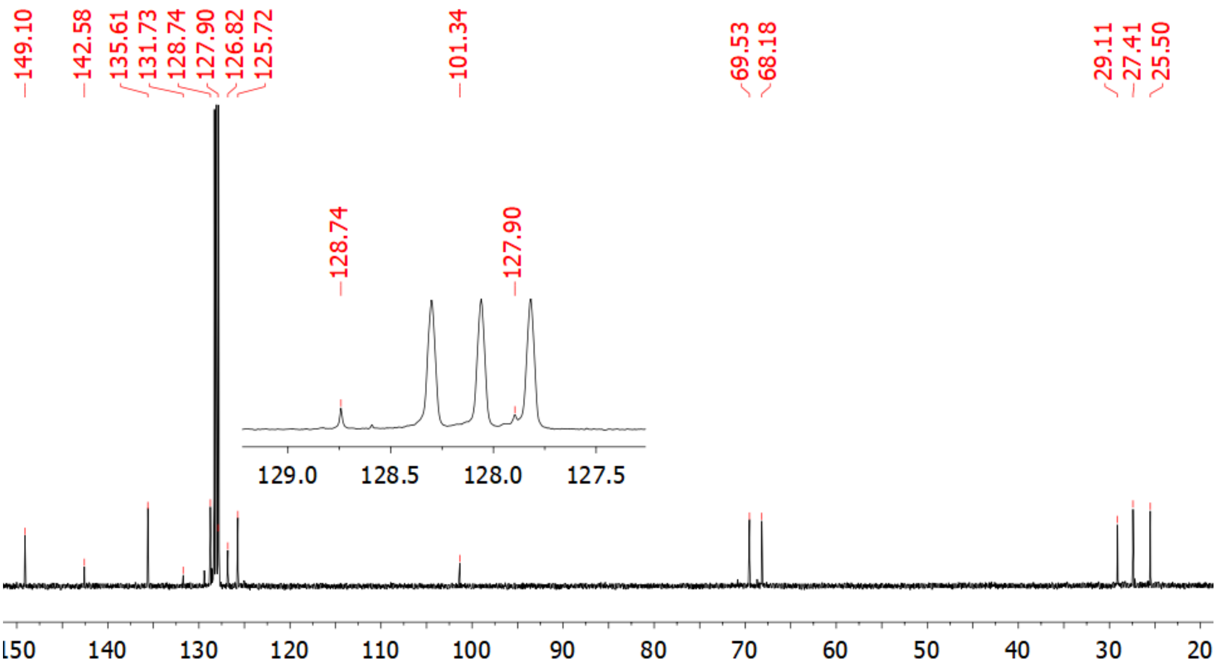

Figure S22. $^{13}C\{^{1}H\}$ NMR spectrum of **1DippSi**$(SePh)_2$ (C_6D_6, 100.5 MHz).

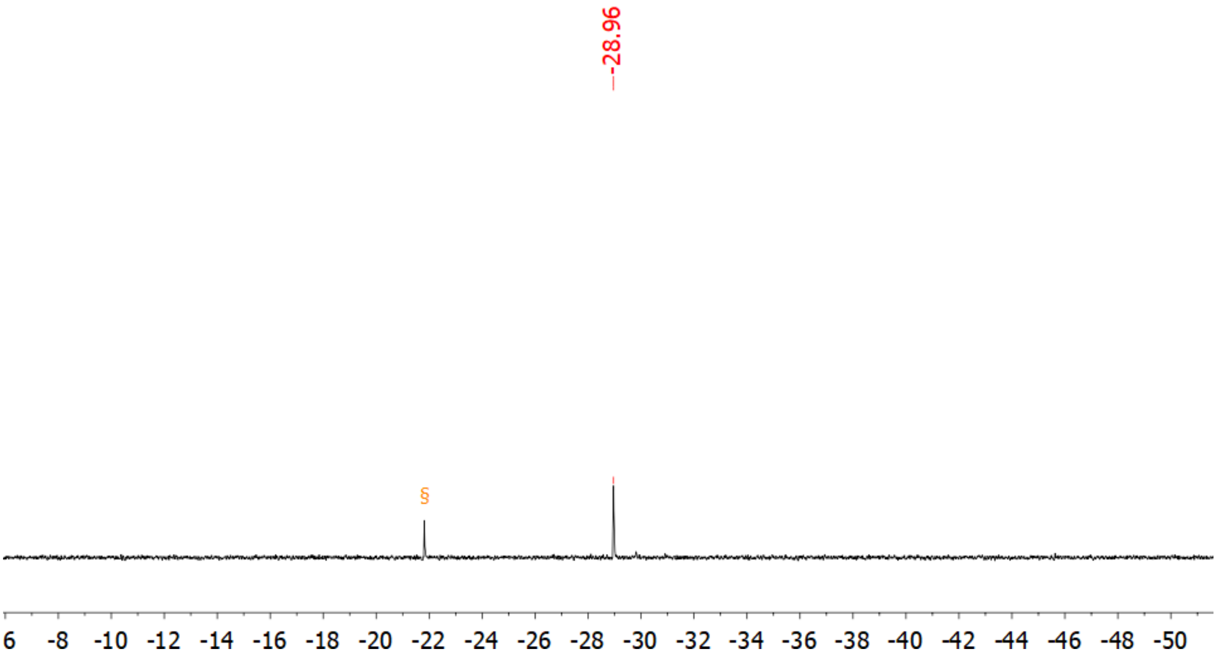

Figure S23. $^{29}Si\{^{1}H\}$ NMR spectrum of **1DippSi**$(SePh)_2$ (C_6D_6, 99.3 MHz). The signal marked belongs to silicon grease (§).

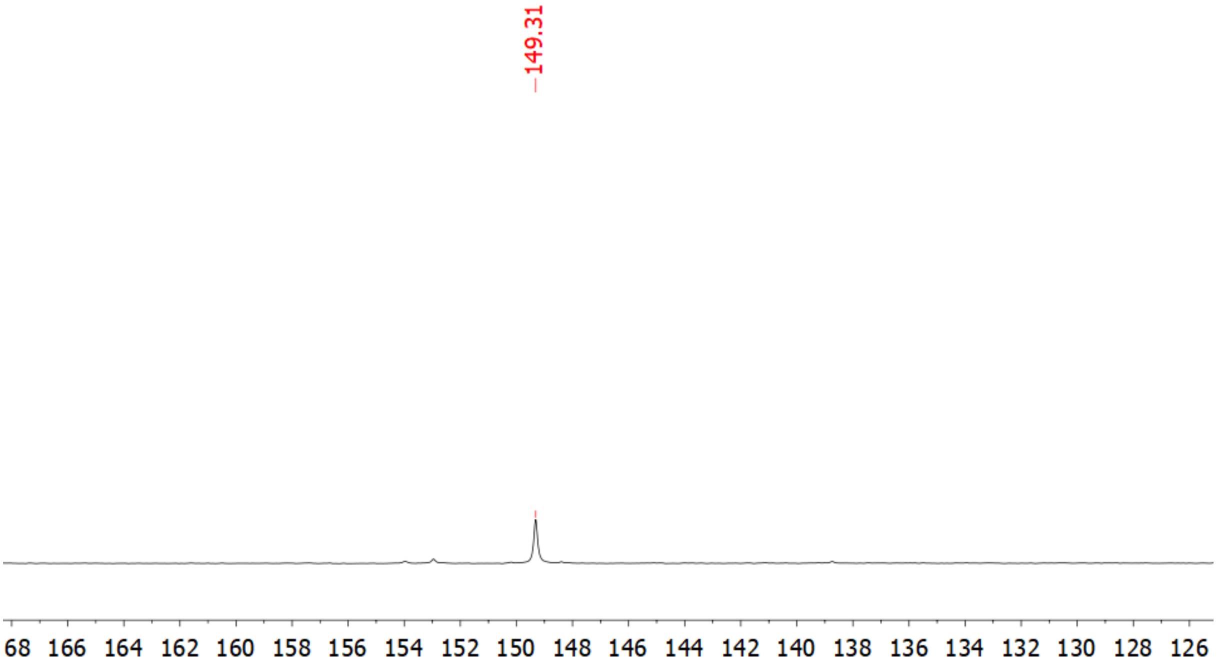

Figure S24. $^{77}Se\{^1H\}$ NMR spectrum of **1DippSi**$(SePh)_2$ (C_6D_6, 95.3 MHz).

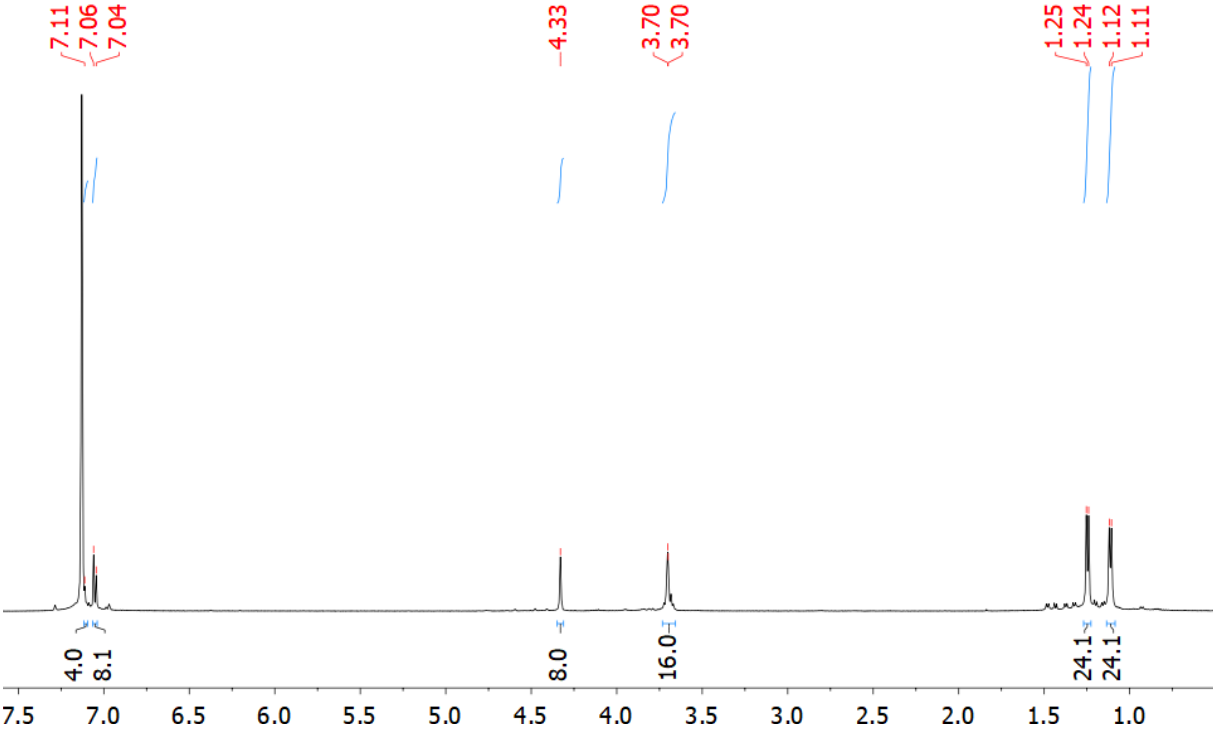

Figure S25. ^1H NMR spectrum of (**1DippSi**$O_2)_2C$ (C_6D_6, 499.7 MHz).

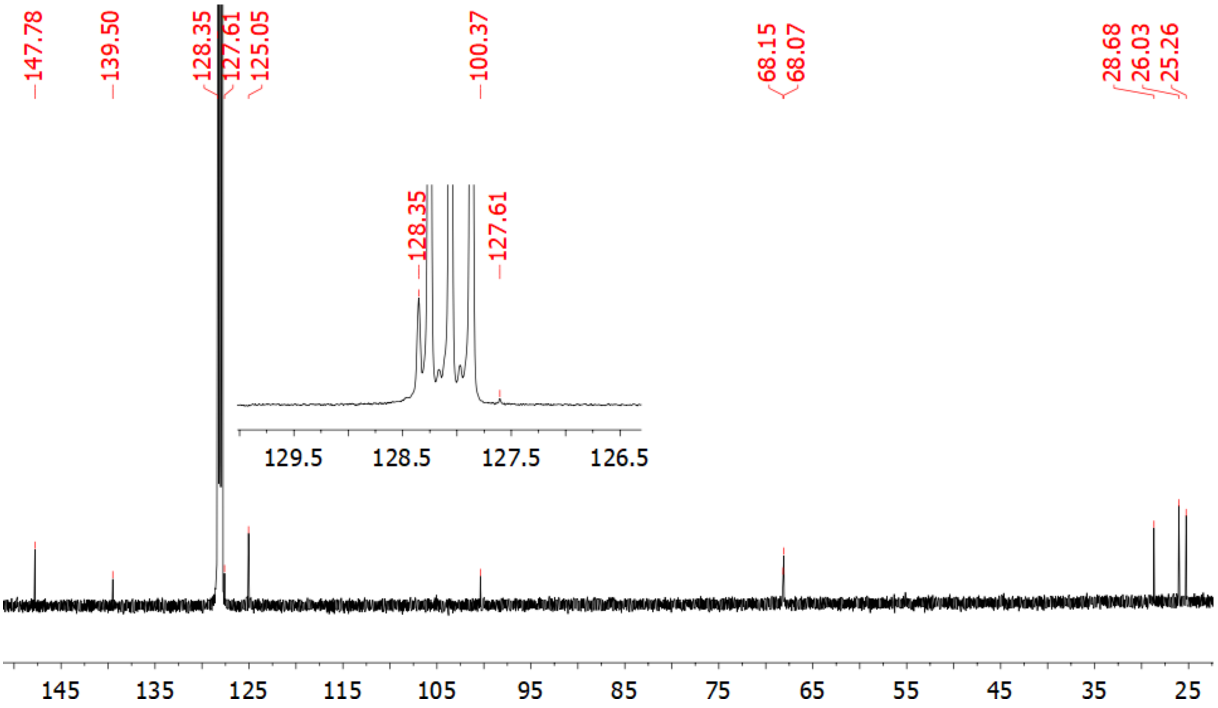

Figure S26. $^{13}C\{^{1}H\}$ NMR spectrum of (**1DippSi**O_2)$_2$C (C_6D_6, 100.5 MHz).

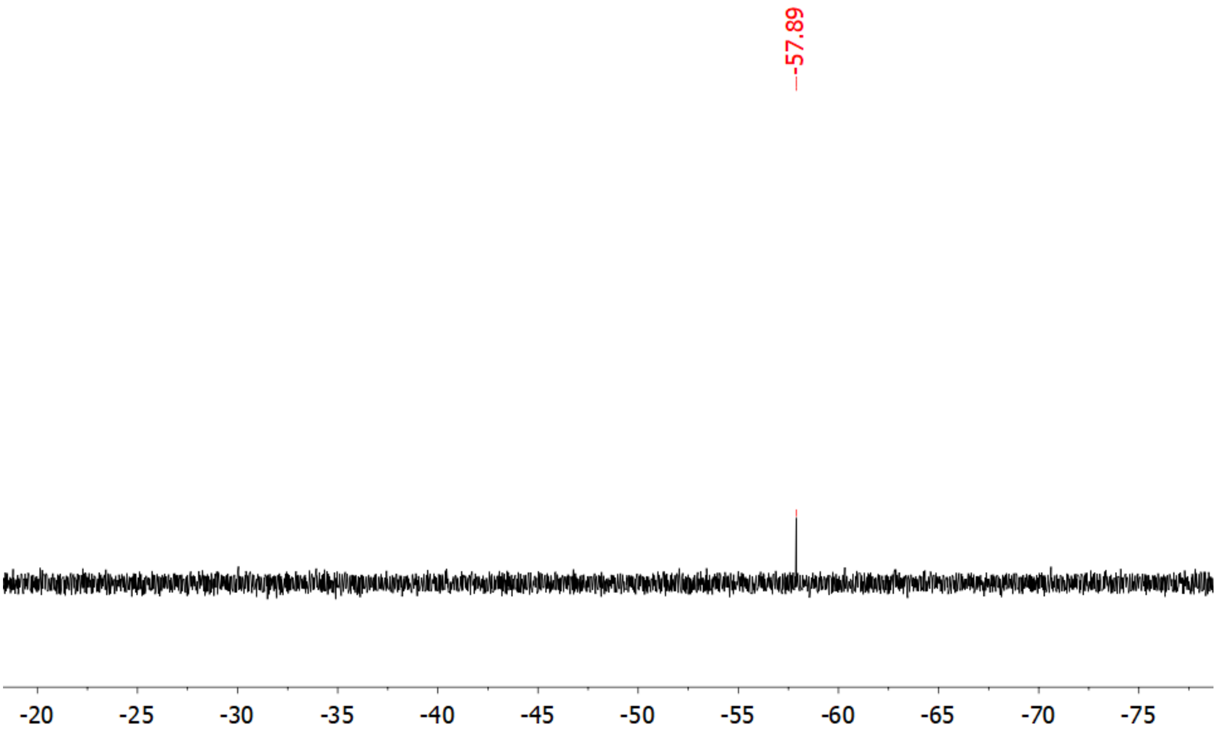

Figure S27. $^{29}Si\{^{1}H\}$ NMR spectrum of (**1DippSi**O_2)$_2$C (C_6D_6, 99.3 MHz).

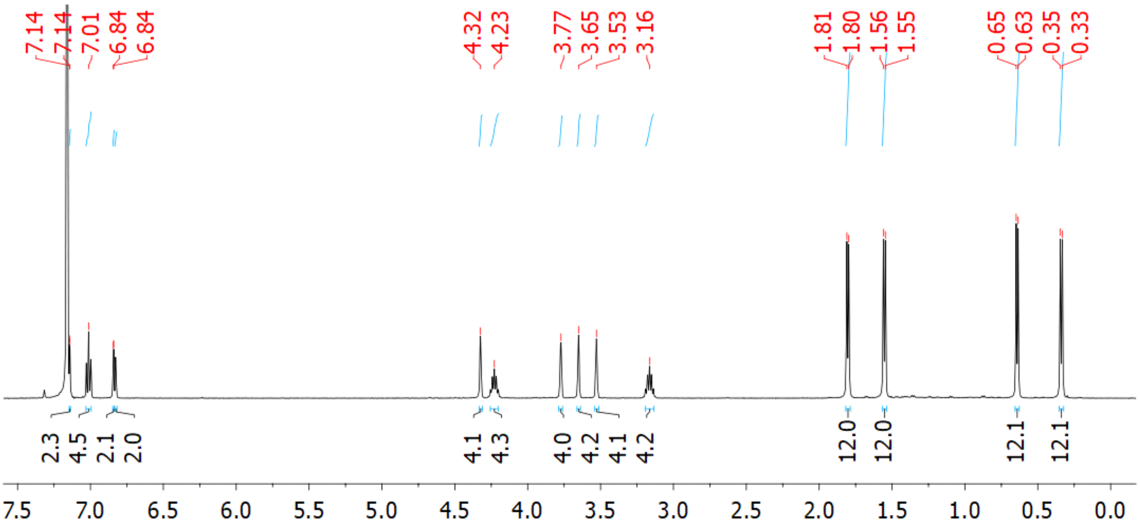

Figure S28. ^{1}H NMR spectrum of (**1DippSi**O)$_2$ (C_6D_6, 499.7 MHz).

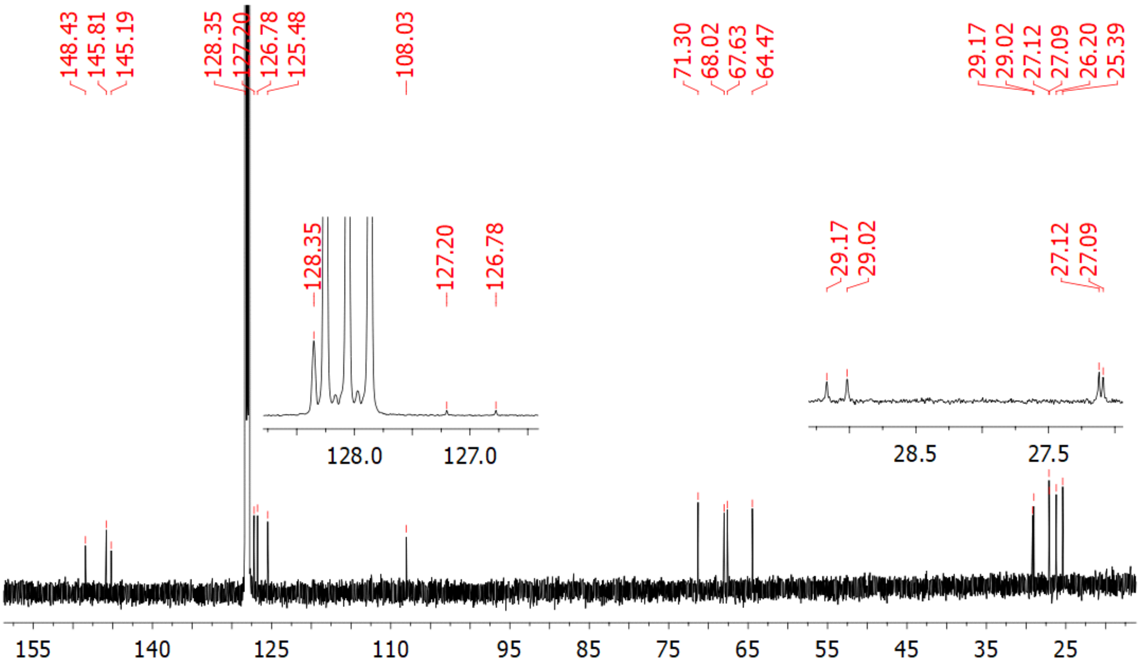

Figure S29. $^{13}C\{^{1}H\}$ NMR spectrum of (**1DippSi**O)$_2$ (C_6D_6, 100.5 MHz).

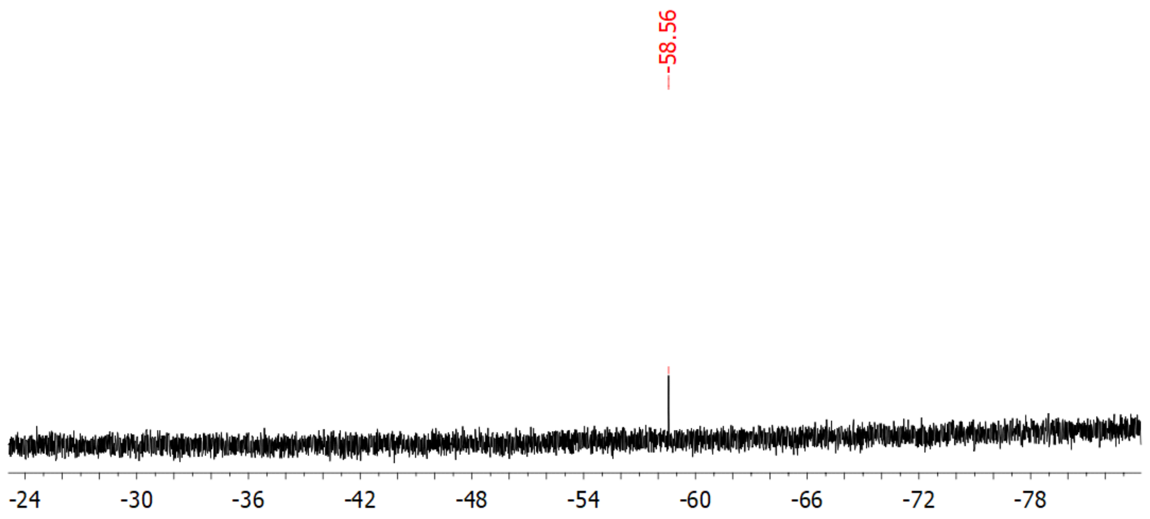

Figure S30. $^{29}Si\{^{1}H\}$ NMR spectrum of (**1DippSi**O)$_2$ (C_6D_6, 99.3 MHz).

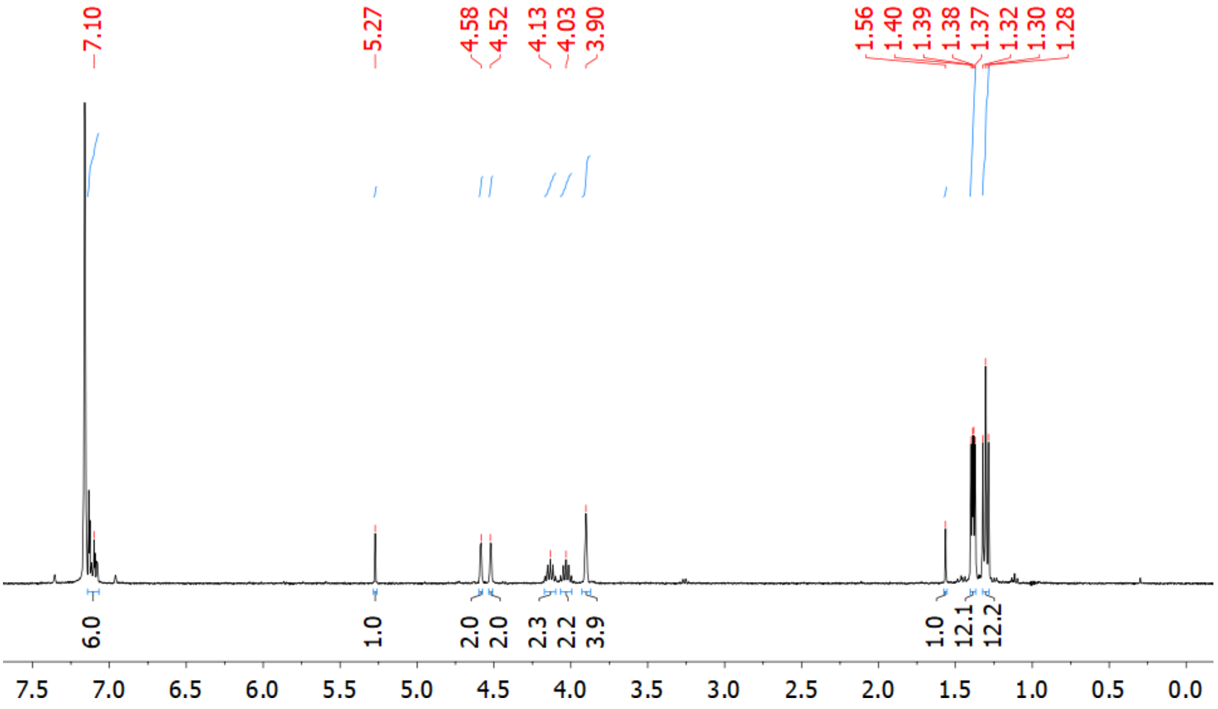

Figure S31. ^{1}H NMR spectrum of **1DippSi**(H)OH (C_6D_6, 499.7 MHz).

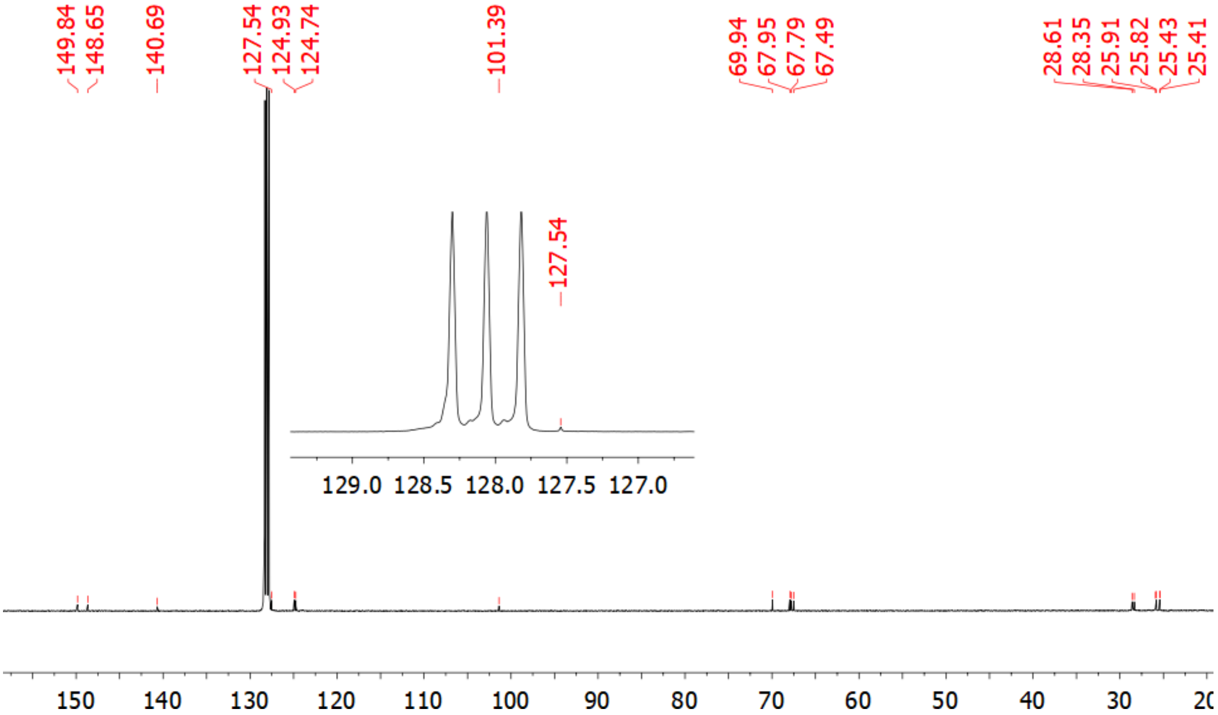

Figure S32. $^{13}C\{^{1}H\}$ NMR spectrum of **1DippSi**(H)OH (C_6D_6, 100.5 MHz).

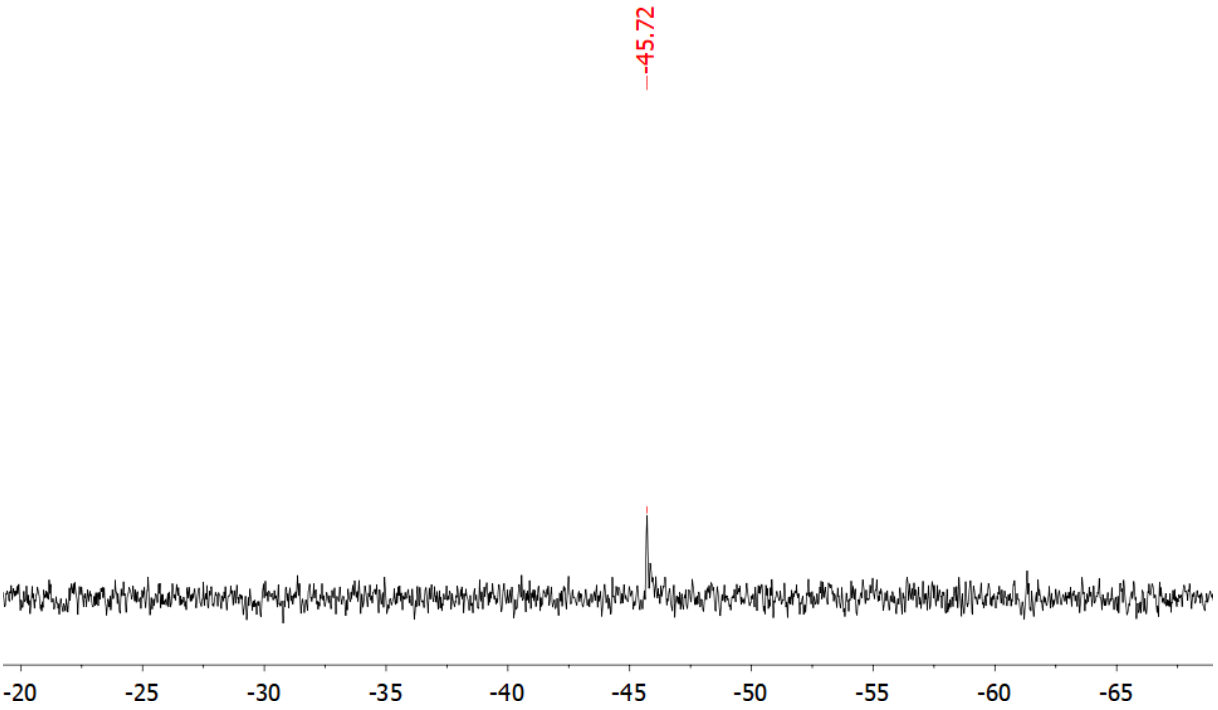

Figure S33. $^{29}Si\{^{1}H\}$ NMR spectrum of **1DippSi**(H)OH (C_6D_6, 99.3 MHz).

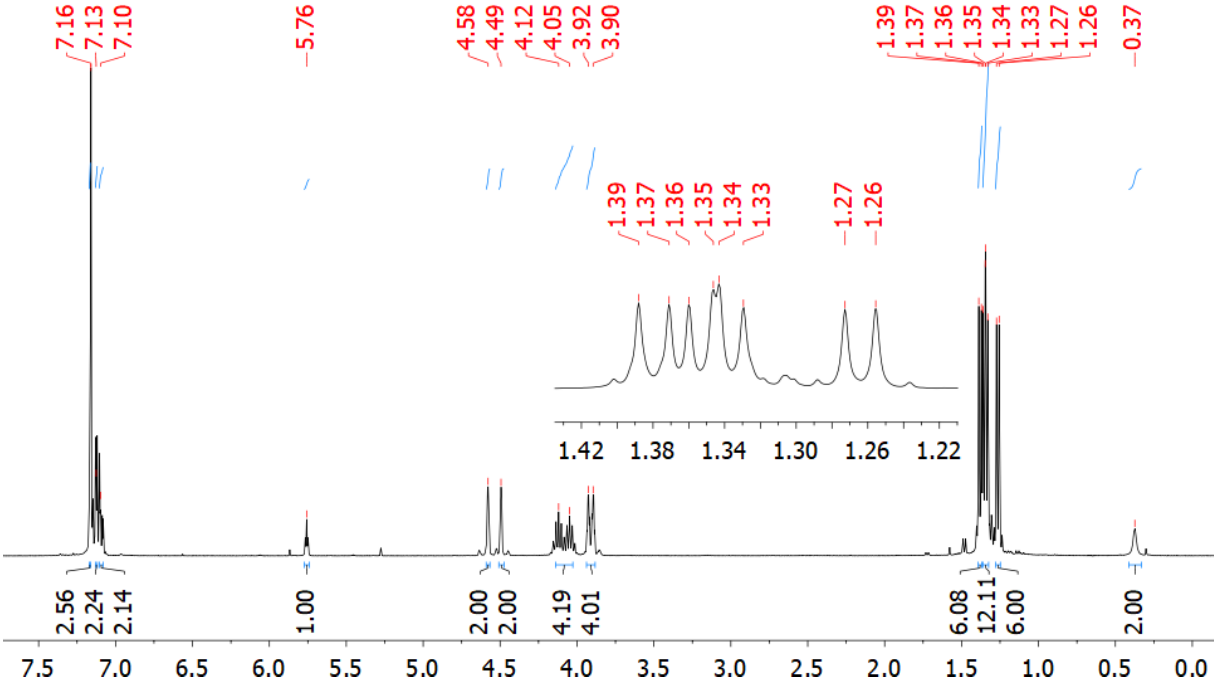

Figure S34. ^{1}H NMR spectrum of **1DippSi**(H)NH_2 (C_6D_6, 499.7 MHz).

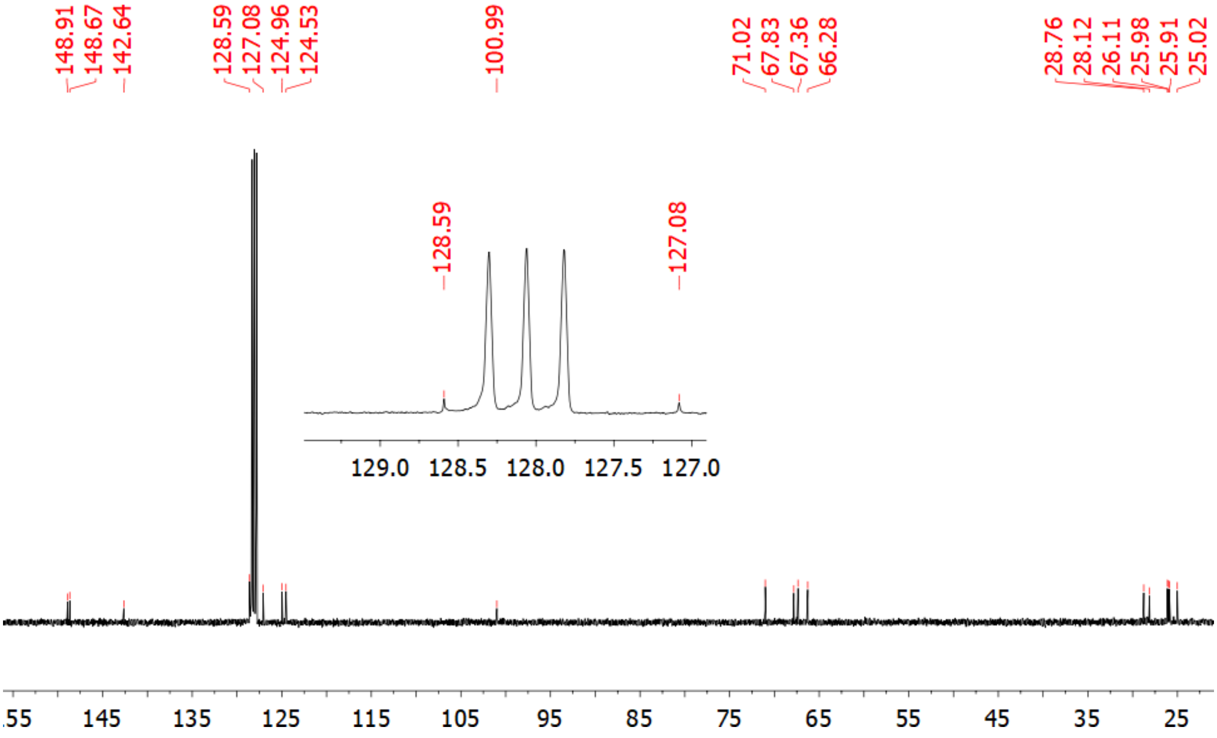

Figure S35. $^{13}C\{^{1}H\}$ NMR spectrum of **1DippSi**(H)NH_2 (C_6D_6, 100.5 MHz).

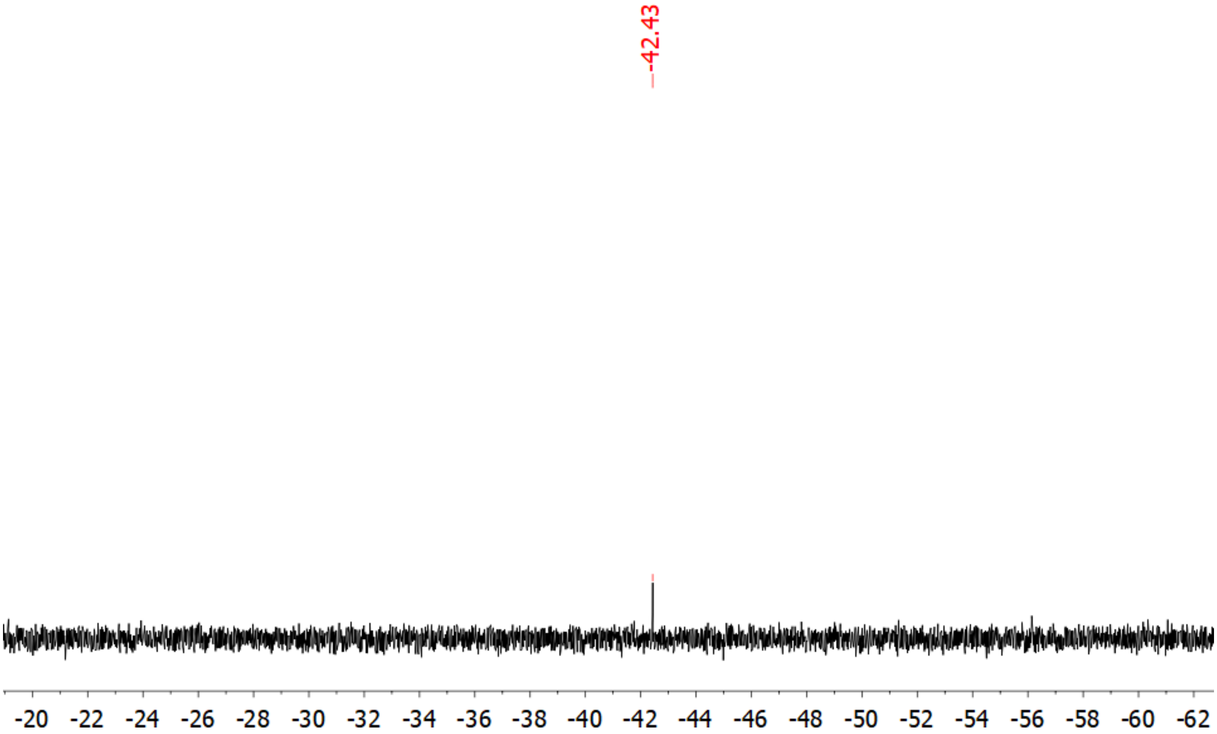

Figure S36. $^{29}Si\{^{1}H\}$ NMR spectrum of **1DippSi**(H)NH_2 (C_6D_6, 99.3 MHz).

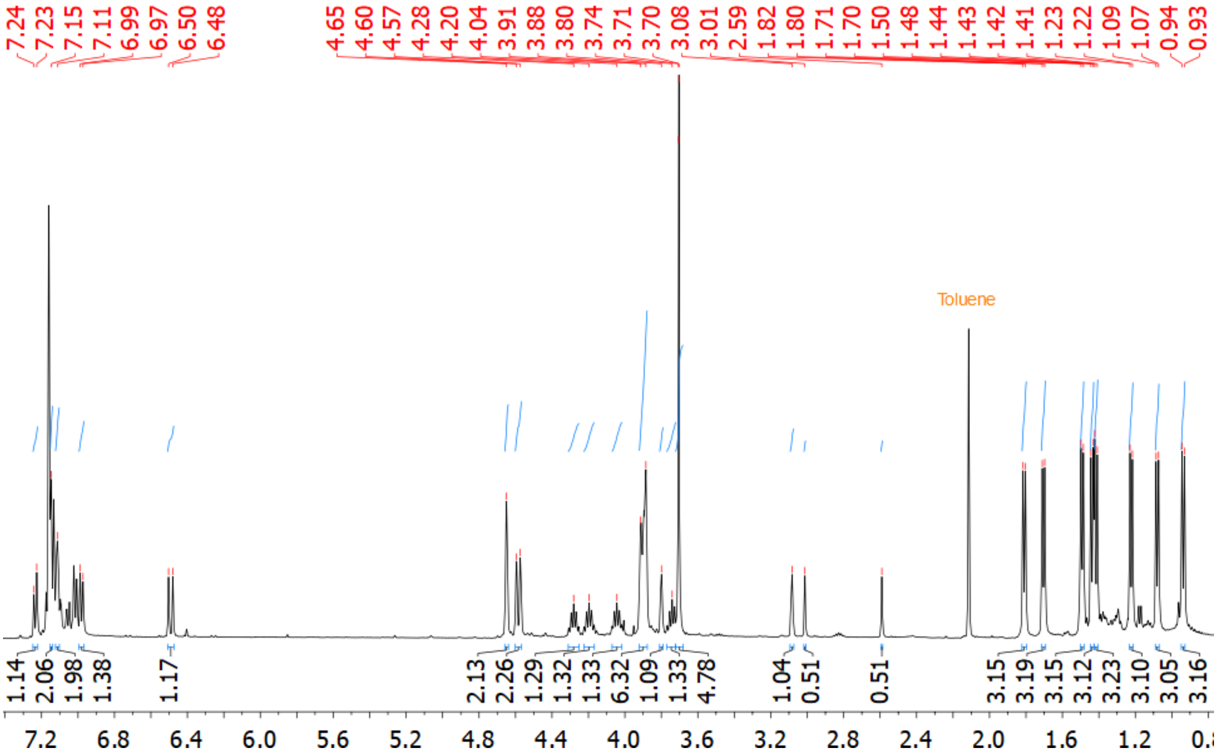

Figure S37. ^{1}H NMR spectrum of of **1DippSi**(H)PHFc (C_6D_6, 499.7 MHz).

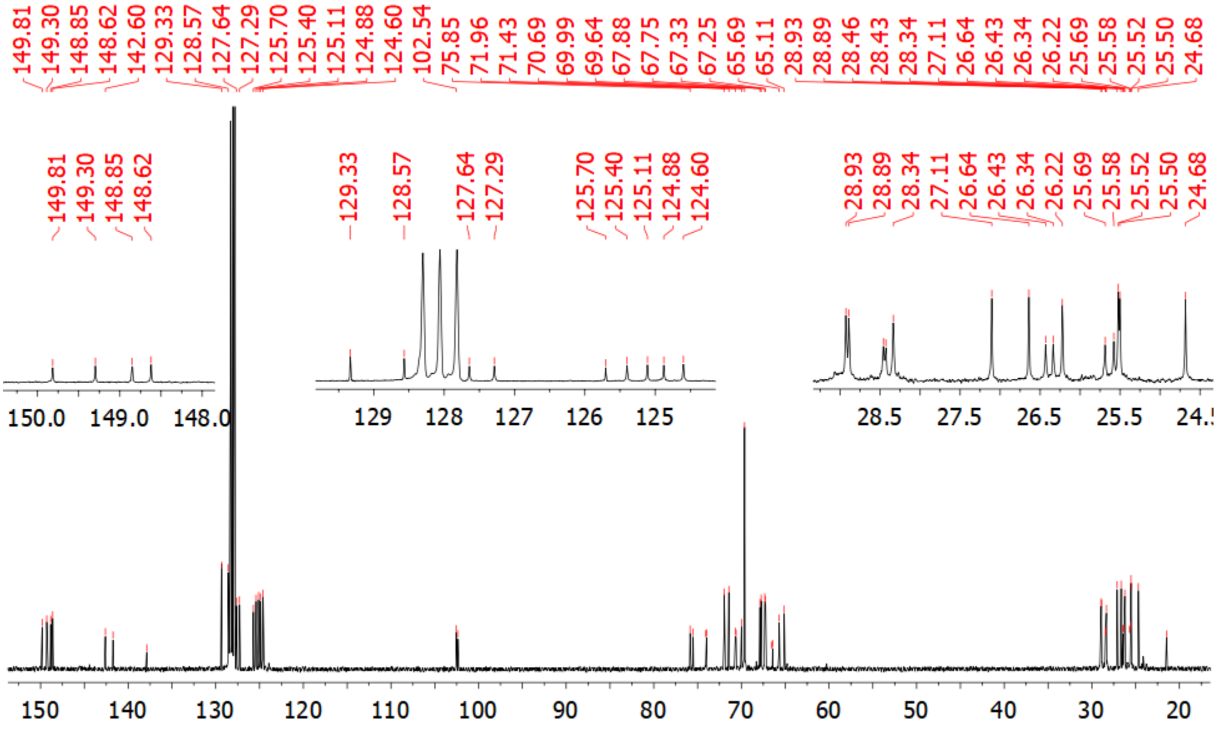

Figure S38. $^{13}C\{^{1}H\}$ NMR spectrum of of **1DippSi**(H)PHFc (C_6D_6, 100.5 MHz).

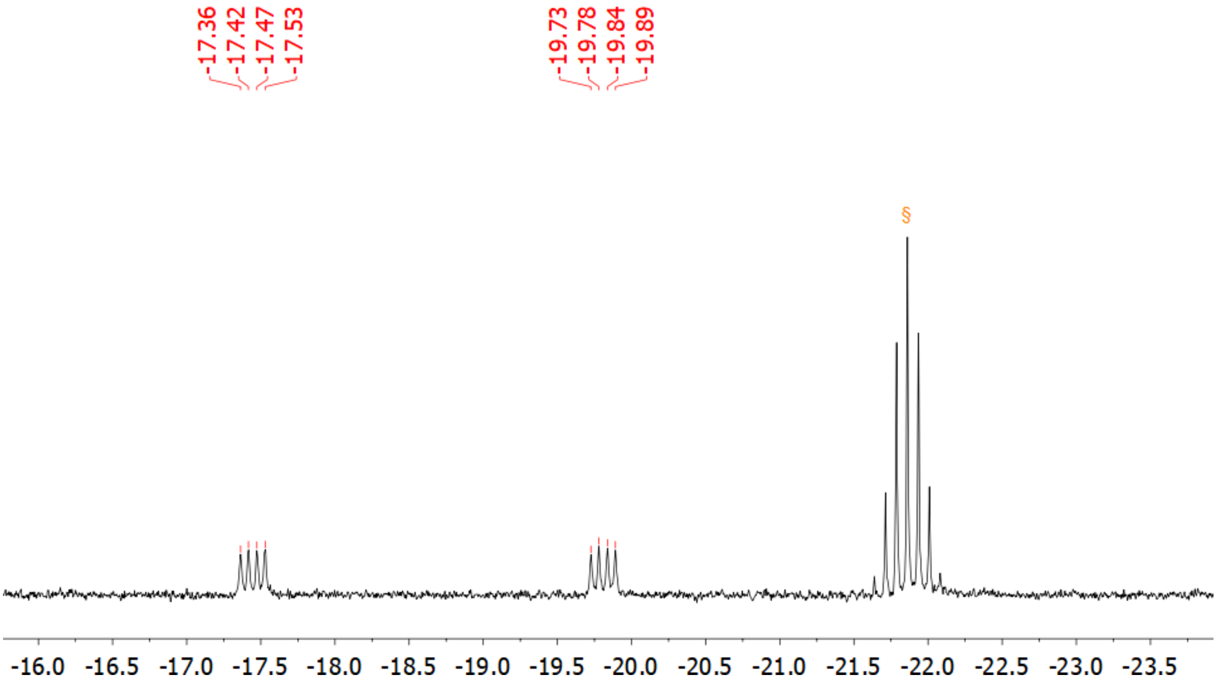

Figure S39. ^{29}Si NMR spectrum of **1DippSi**(H)PHFc (C_6D_6, 99.3 MHz). The signal marked belongs to silicon grease (§).

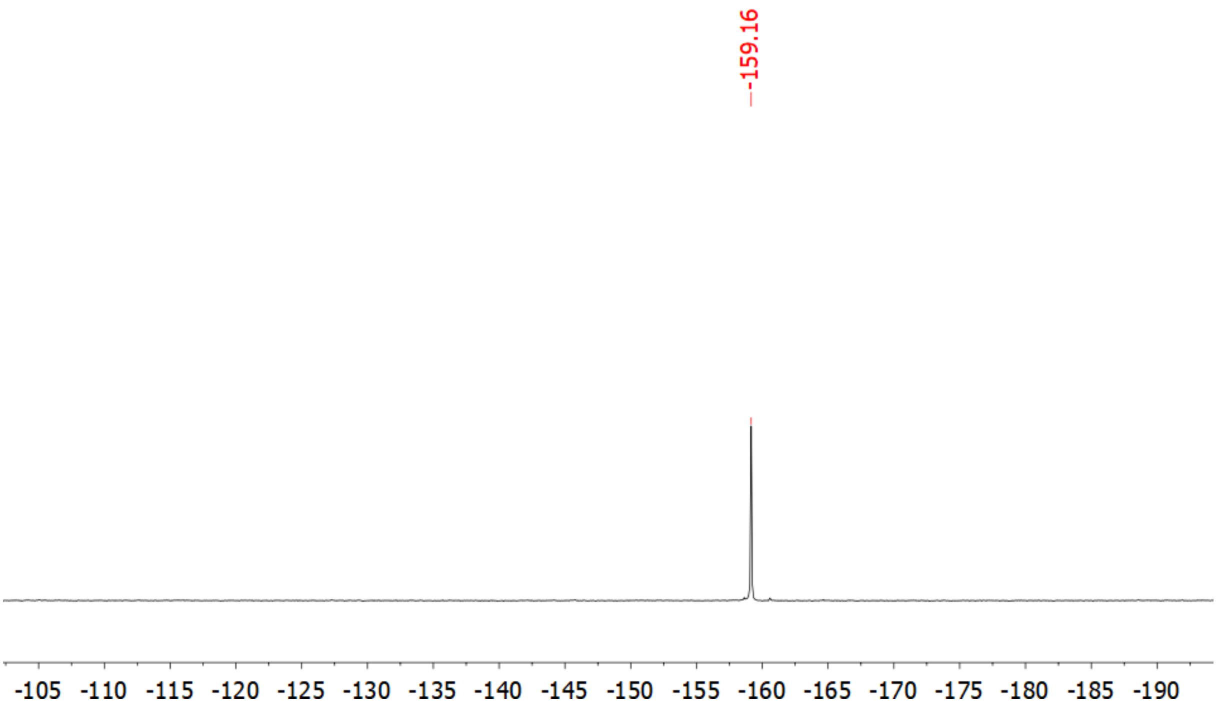

Figure S40. $^{31}P\{^{1}H\}$ NMR spectrum of **1DippSi**(H)PHFc (C_6D_6, 202.3 MHz).

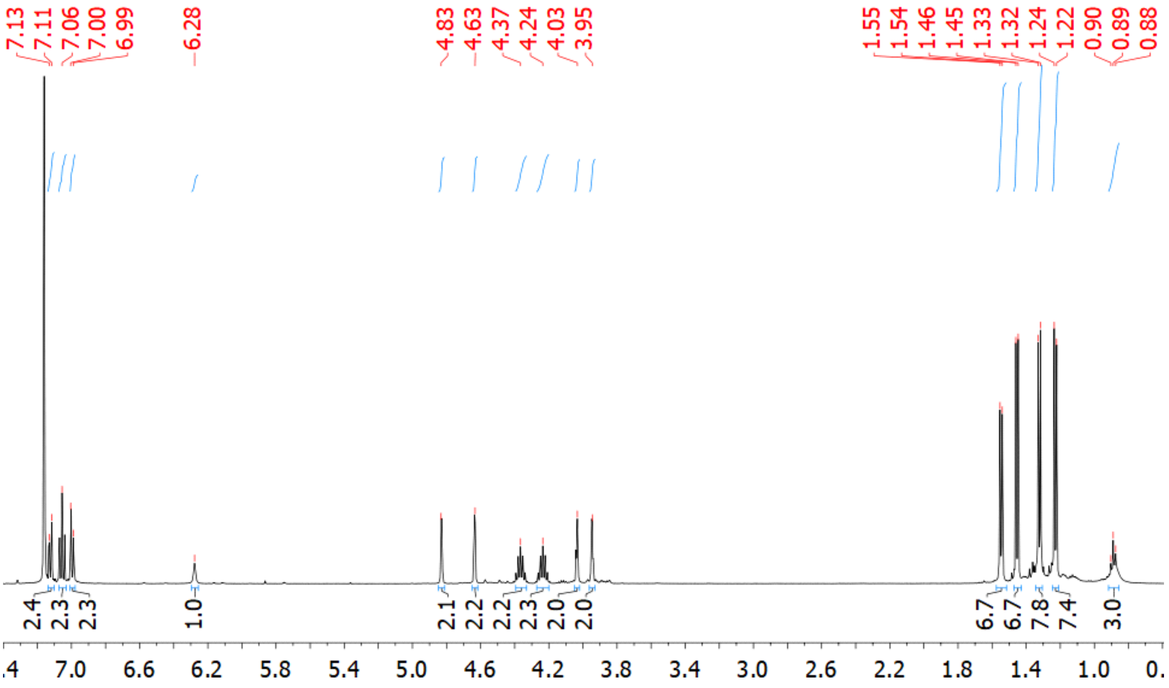

Figure S41. ^{1}H NMR spectrum of **1DippSi**(H)(BH_2NH_3) (C_6D_6, 499.7 MHz).

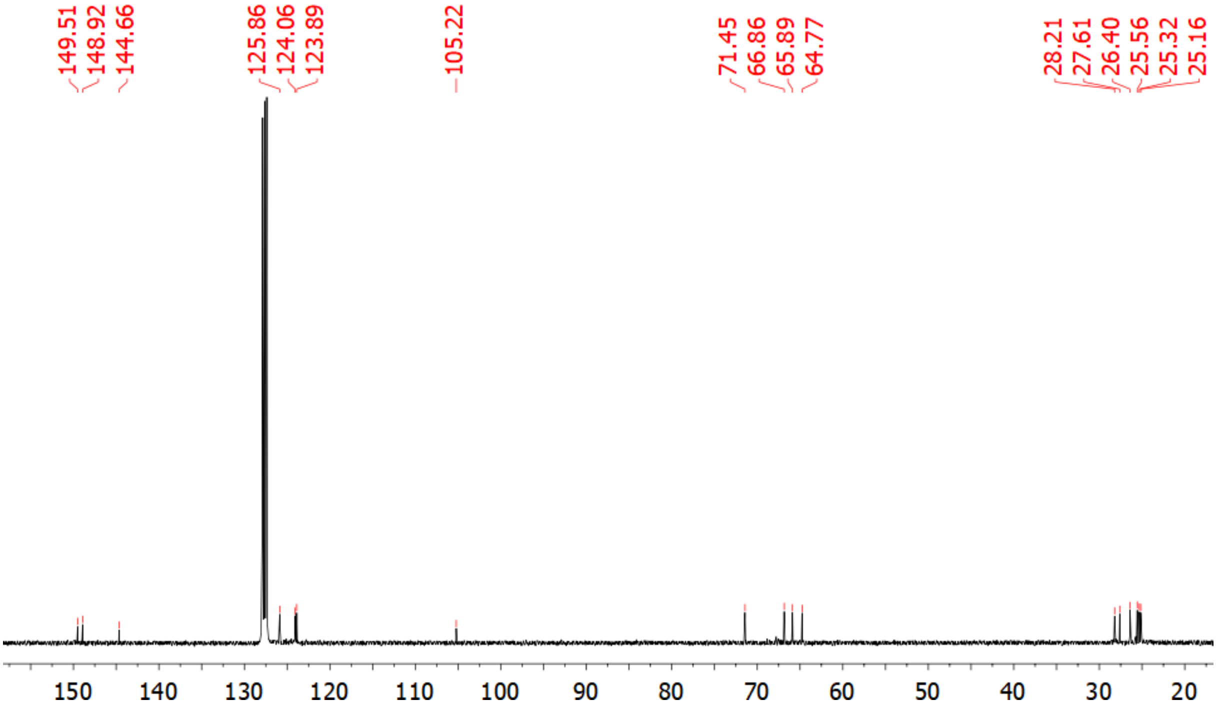

Figure S42. ^{13}C NMR spectrum of **1DippSi**(H)(BH_2NH_3) (C_6D_6, 100.5 MHz).

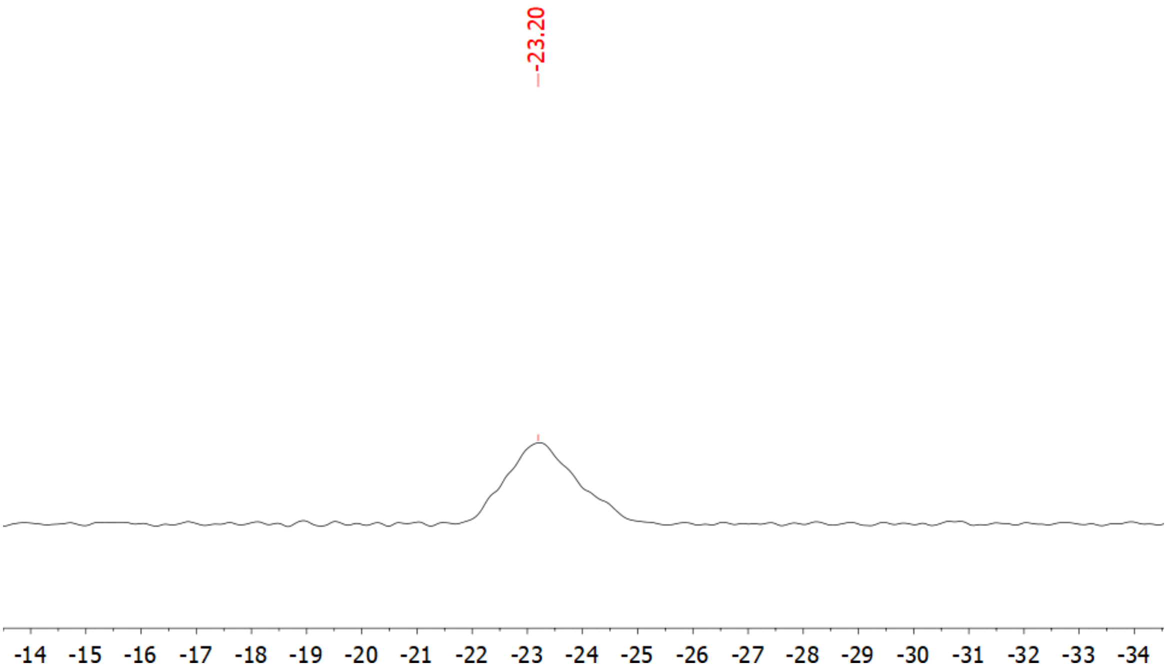

Figure S43. $^{11}B\{^1H\}$ NMR spectrum of **1DippSi**(H)(BH_2NH_3) (C_6D_6, 160.3 MHz).

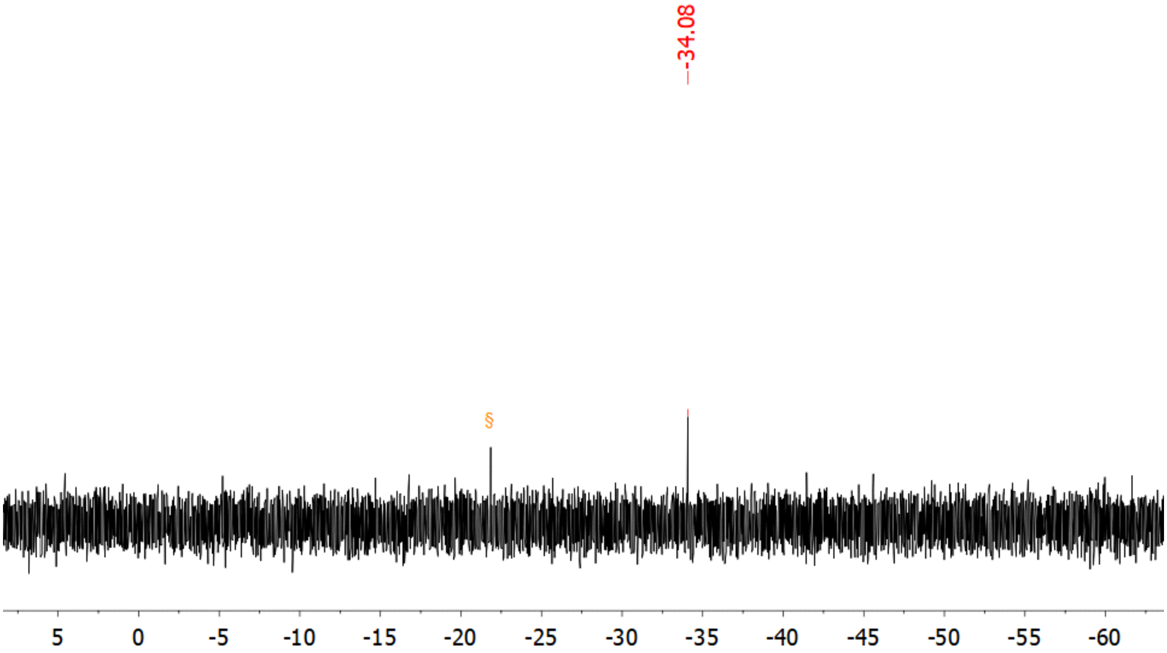

Figure S44. $^{29}Si\{^1H\}$ NMR spectrum of crude **1DippSi**(H)(BH_2NH_3) (C_6D_6, 99.3 MHz). Although data collection for this spectrum took 3 d, the signal of the product could not be detected. Instead, the spectrum reveals weak signals due to silicon grease (§) and another silicon-containing species present in very low concentration, which is assumed to be **1DippSi**H_2. For comparison, the corresponding signal of the TMS homologue **1TMSSi**H_2 was observed at $\delta = -44.0$ ppm (see Ref. [S6]). The ^{29}Si NMR chemical shifts computed (BLYP/TZ2P) for **1DippSi**H_2 (compound **7** according to the computational numbering scheme) and **1TMSSi**H_2 are −40.6 and −54.1 ppm, respectively, while $\delta = -12.9$ ppm was computed for **1DippSi**(H)(BH_2NH_3).

II Computational Details

Geometry optimizations and harmonic frequency calculations were performed using the ORCA program package[S7] (Version 4.1.2) employing the PBEh-3c density functional composite method[S8] combined with a higher integration grid (Grid5) to avoid spurious imaginary frequencies. Optimized structures were characterized as minima or first order saddle points by eigenvalue analysis of the computed Hessians. Connectivities between minima and the corresponding transition states were validated by intrinsic reaction coordinate (IRC) calculations[S9] or by displacing the transition-state geometries along both directions of the transition mode, followed by unconstrained optimizations to the respective minima.

For selected structures, relative energies from correlated wavefunction theory were computed on a smaller model system as well as on the full system. For a smaller system with H-truncated nitrogen atoms single-point energies were obtained using the explicitly correlated coupled-cluster ansatz[S10] CCSD(T)-F12b[S11] as implemented in the Molpro2015.1 program[S12] in combination with the F12-optimized correlation consistent polarized triple-zeta orbital[S13] and auxiliary[S14-S16] basis sets of the cc-pVTZ-F12 family for all non-metal atoms. For the Fe atom, the aug-cc-pVTZ orbital basis[S13] was used in conjunction with the universal JKfit option for integral fitting in the Fock matrix construction along with the aug-cc-pVTZ/MP2Fit auxiliary basis used for the many-electron integrals and CABS representation. For the full system single-point energies were obtained from correlated DLPNO-CCSD(T)-F12[S11, S17] computations as implemented in ORCA 4.2.1 using the F12-optimized correlation consistent polarized triple-zeta orbital and auxiliary basis sets for non-metal atoms. For the Fe atom, the aug-cc-pVTZ orbital basis and associated auxiliary sets were employed.

Structures for NMR calculations were reoptimized using the Gaussian09, Rev. D.01 program[S18] employing the M06-L density functional[S19] together with the 6-31+G(d,p) basis set.[S20, S21] Based on these geometries, ^{29}Si NMR chemical shifts were computed relative to tetramethylsilane with the ADF2013.01 program.[S22-S24] The BLYP functional[S25, S26] was used in these calculations in combination with the TZ2P Slater-type basis set.[S27]

Pictures of molecular structures were generated with the Cylview[S28] and ChemCraft[S29] programs. All energies given are relative free energies at 298.15 K and 1 atm (ΔG^{298}) in kcal mol^{-1}.

For convenience, the following Chart shows the numbers of the computed structures for experimentally observed compounds:

**1DippSi**	**1**
**1DippSi**H(NH_2)	**3**
**1DippSi**H(OH)	**6**
**1DippSi**H_2	**7**
**1DippSi**H(BH_2NH_3)	**10**

Structural Metrics of [Fe{(η^5-C_5H_4)N(2,6-*i*$Pr_2C_6H_3$)}$_2$Si:] and the reaction products 3 and 10

Table S2. Selected metrics of the PBEh-3c optimized and the X-ray structure for **1DippSi**. Hydrogen atoms and isopropyl groups of Dipp substituents are omitted.

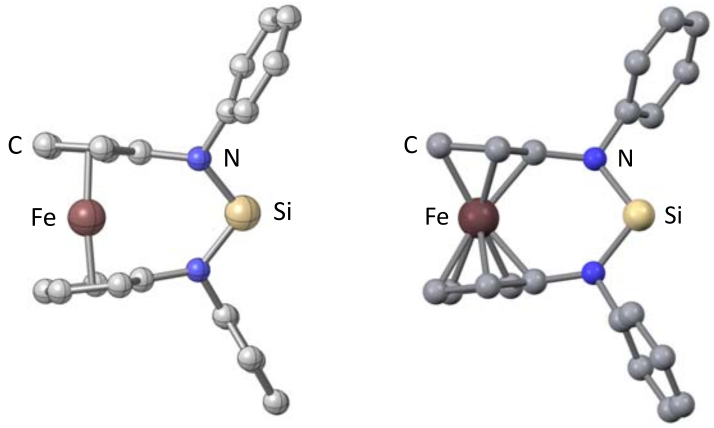

	PBEh-3c	exp. X-RAY
Si–N	1.75 Å	1.74 Å
N–Si–N	105°	106°
Σ∡(N)	360°	360°

Table S3. Selected metrics of the PBEh-3c optimized and the X-ray structure for **3**. Carbon-bound hydrogen atoms and isopropyl groups of Dipp substituents omitted.

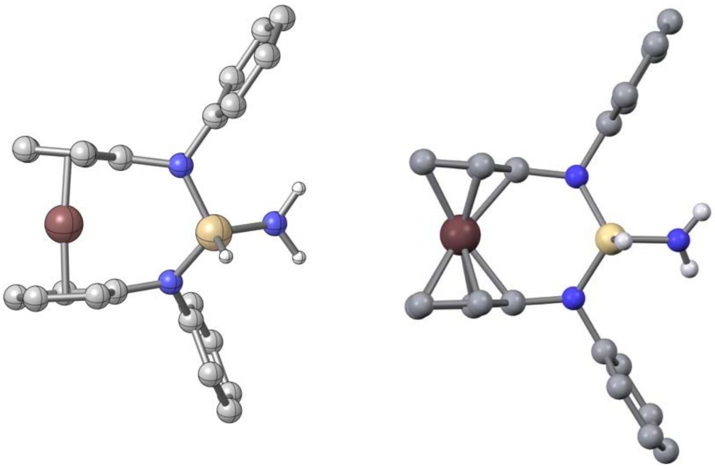

	PBEh-3c	exp. X-RAY
Si–N^{Dipp}	1.75 Å	1.72, 1.73 Å
Si–NH_2	1.72 Å	1.69 Å
N–Si–N	113°	112°
Σ∡(N^{Dipp})	359, 360°	358°

Table S4. Selected metrics of the PBEh-3c optimized and X-ray structure for **10**. Carbon-bound hydrogen atoms and isopropyl groups of Dipp substituents omitted.

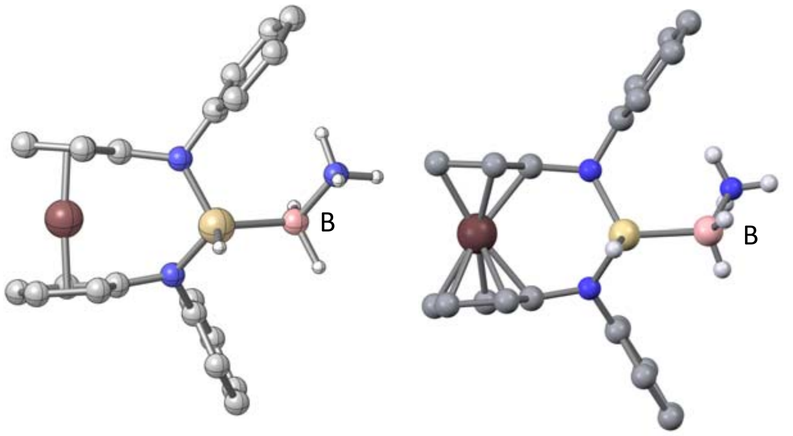

	PBEh-3c	exp. X-RAY
Si–N^{Dipp}	1.79, 1.76 Å	1.77, 1.76 Å
Si–B	2.01 Å	2.01 Å
B–N	1.64 Å	1.60 Å
N–Si–N	109°	108°
Σ∡(N^{Dipp})	358, 359°	357, 358°

Comparison of frontier molecular orbitals and spin densities

For comparison with **1** HOMO-LUMO gaps and singlet-triplet splittings have been calculated for three experimentally characterized silylenes at the PBEh-3c level of theory. Figure S45 shows the respective Lewis structures, frontier molecular orbitals and spin densities of the triplet state. All energies given are in eV.

As an illustration of the method dependence of the HOMO-LUMO gap we computed the orbital energies of **1** with an exemplary choice of functionals commonly in use (Figure S46). We note a significant reordering of the occupied molecular orbitals obtained with the various methods, and for the difference between the silylene lone pair and the empty p-orbital at Si we obtain values ranging from 3.7 to 9.1 eV.

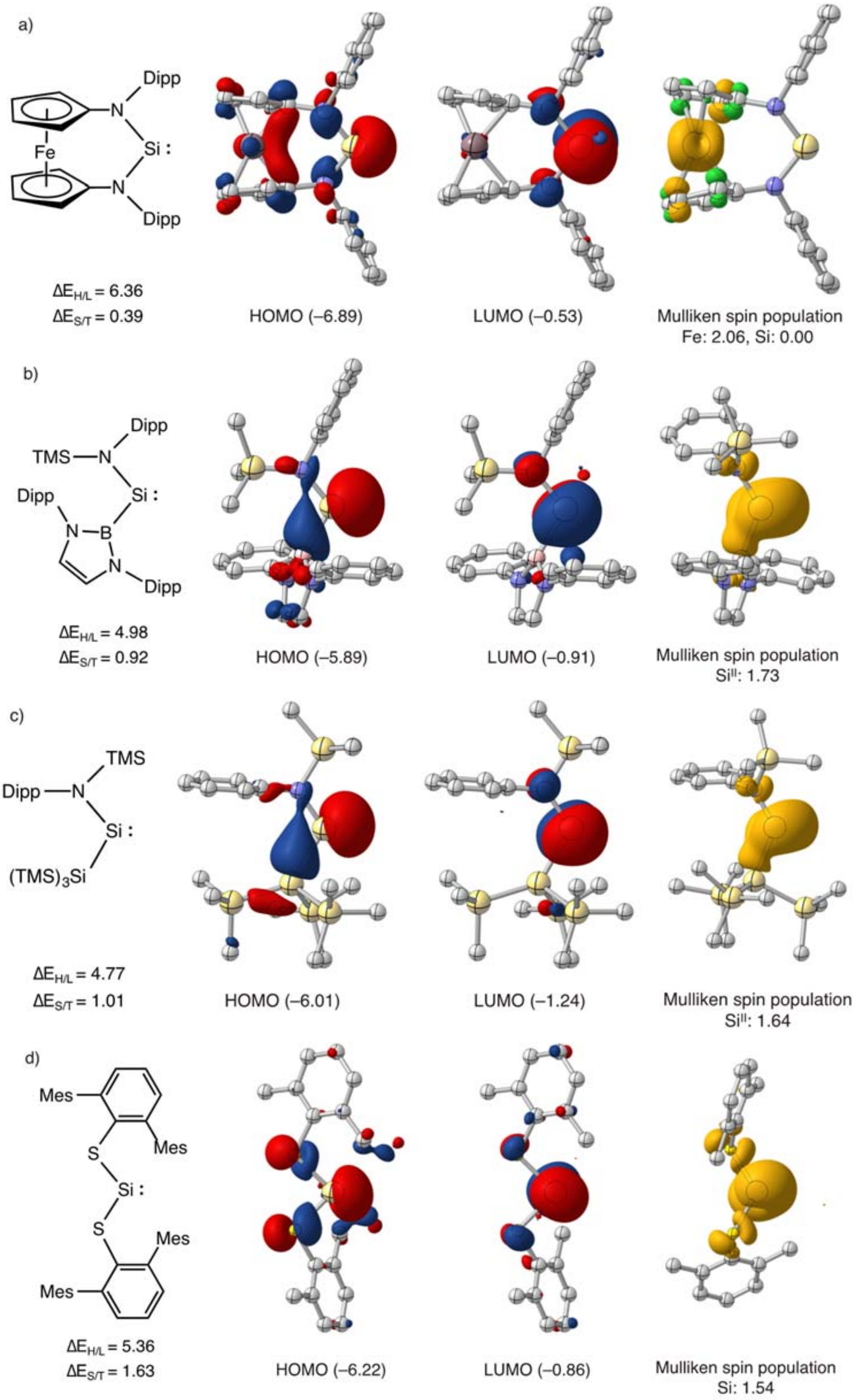

Figure S45. Comparison of electronic characteristics for the full molecular models of a) **1** (≙ **1DippSi**), b) the acyclic silylene Si{B(NDippCH)$_2$}{N(SiMe$_3$)Dipp} of Protchenko *et al.*,[S30] c) the acyclic silylene Si{Si(SiMe$_3$)$_3$}{N(SiMe$_3$)Dipp} of Protchenko *et al.*[S31] and d) the acyclic silylene Si{S(C$_6$H$_3$-2,6(C$_6$H$_2$-2,4,6-Me$_3$)$_2$)}$_2$ of Rekken *et al.*;[S32] Lewis structures, frontier molecular orbitals and triplet spin density distributions computed for the full molecular models (orbital energies and singlet-triplet gaps in eV, isocontour surfaces at ±0.05 $a_0^{-3/2}$ for orbitals and ±0.005 a_0^{-3} for the spin density, α spin: yellow, β spin: green; *i*Pr groups, Mes groups and hydrogen atoms not shown)

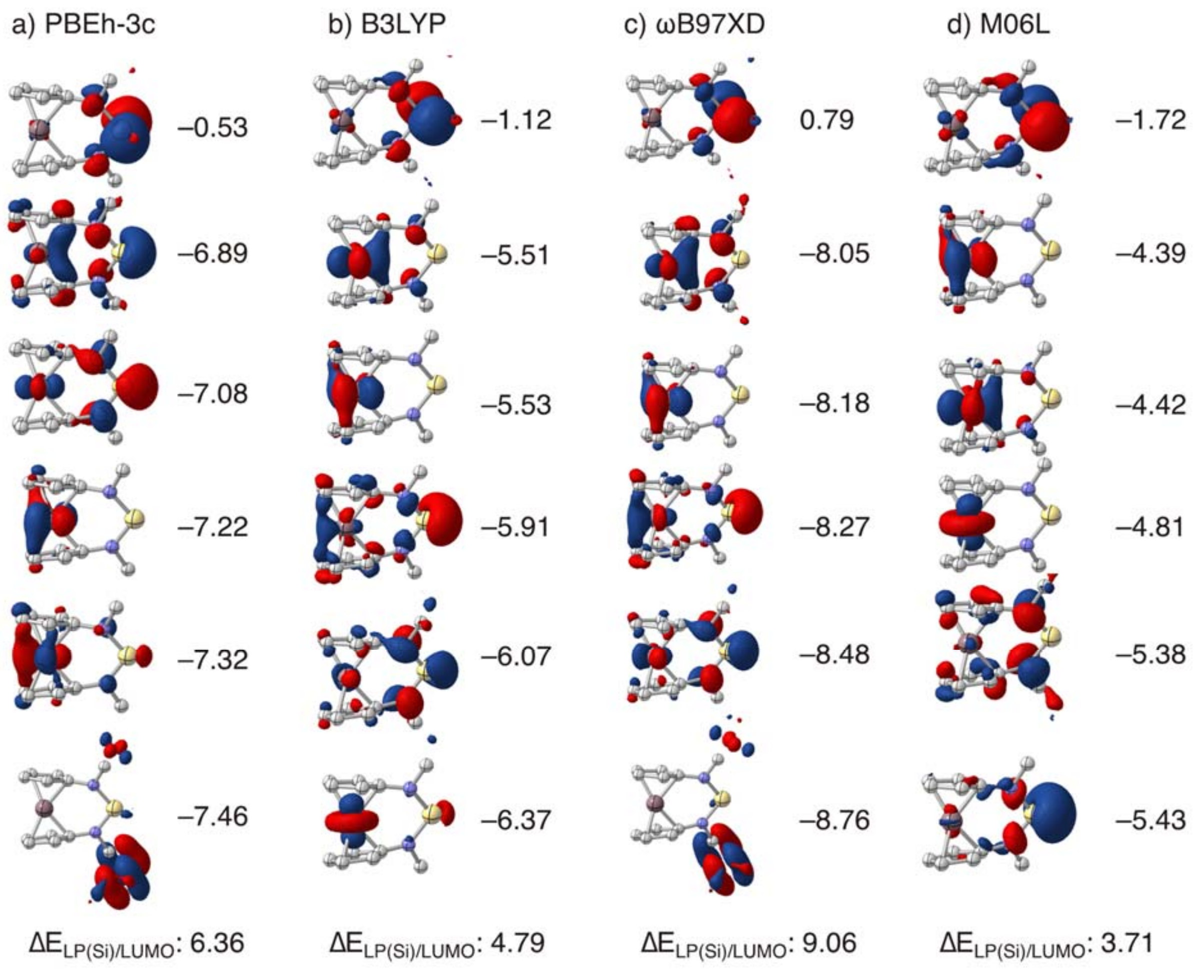

Figure S46. Comparison of frontier molecular orbitals calculated with four density functional methods; a) PBEh-3c, b) B3LYP/def2-TZVP,[S33, S34] c) ωB97XD/def2-TZVP[S35] and d) M06-L/def2-TZVP[S19] (orbital energies in eV, isocontour surfaces at ±0.05 $a_0^{-3/2}$; Dipp groups are indicated by the *ipso*-C atom, hydrogen atoms not shown).

Evaluation of the PBEh-3c method for the full and the H-truncated molecular system

The PBEh-3c method has primarily been designed for the efficient assessment of molecular structures but it also provides reasonable relative energies with respect to benchmark databases.[S8] To test the suitability of PBEh-3c for the reactions investigated here, the thermochemistry for two representative reactions were compared to single-point energies from correlated wavefunction theory (Schemes S1 and S2). Unless stated otherwise in the following, relative free energies ΔG^{298} are given in kcal mol^{-1} (imaginary wave numbers of transition modes in parentheses, cm^{-1}). Bonds formed or broken in transition states are shown dashed, unreactive H atoms are omitted and the orientation of Dipp-substituents in transition state structures are indicated by showing the C_{ipso} atom only.

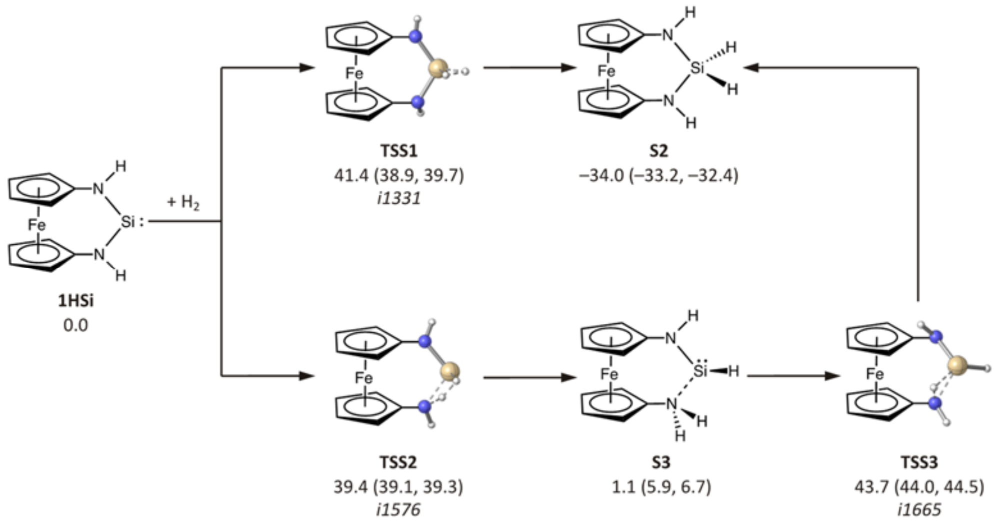

Scheme S1. Computed reaction paths for the H−H bond insertion in the H-truncated molecular model **1HSi**; PBEh-3c relative total energies Δ*E* in kcal mol^{-1} (CCSD(T)-F12b/cc-pVTZ//PBEh-3c, DLPNO-CCSD(T)-F12/cc-pVTZ//PBEh-3c in parentheses, in this order).

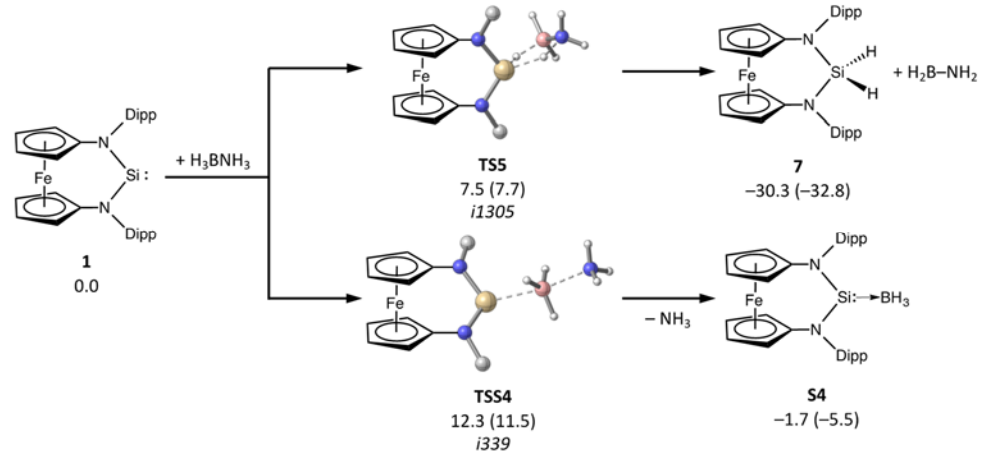

Scheme S2. Computed reaction paths for the initial reaction of **1** with H_3BNH_3 employing the full molecular model; PBEh-3c relative total energies Δ*E* in kcal mol^{-1}, DLPNO-CCSD(T)-F12/cc-pVTZ//PBEh-3c results in parentheses).

Additional Information on reaction paths investigated

The silylene **1DippSi** does not react with H_2 to the corresponding silane, which is in line with reaction barriers above 50 kcal mol^{-1} calculated for **1** for both addition pathways identified (Scheme S3).

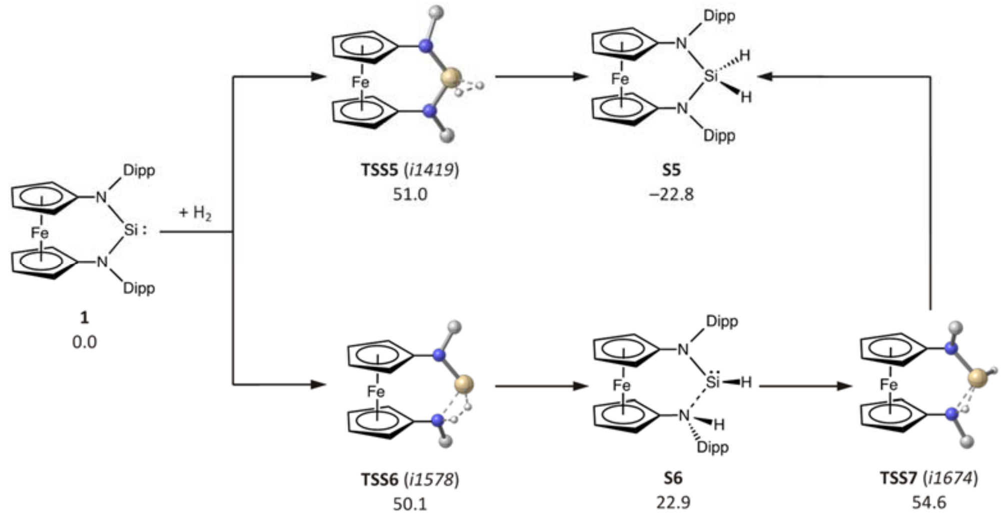

Scheme S3. Computed reaction paths for the H−H bond insertion by **1**.

For the reaction of **1** with H_2O multiple paths involving one or two additional H_2O molecules acting as proton shuttles were investigated. For the rate determining transition state of the most favorable path the corresponding kinetic isotope effect was calculated. In order to estimate the effect of hydrogen tunneling, a 1-D Wigner correction[S36] was applied. The direct oxidative addition of water to **1** is kinetically least favorable (Scheme S4 path d) while O−H addition across a Si−N bond with subsequent proton shift is effectively catalyzed by a proton-shuttle mechanism with additional water molecules (Scheme S4, a-c and Scheme 3 in the main text).

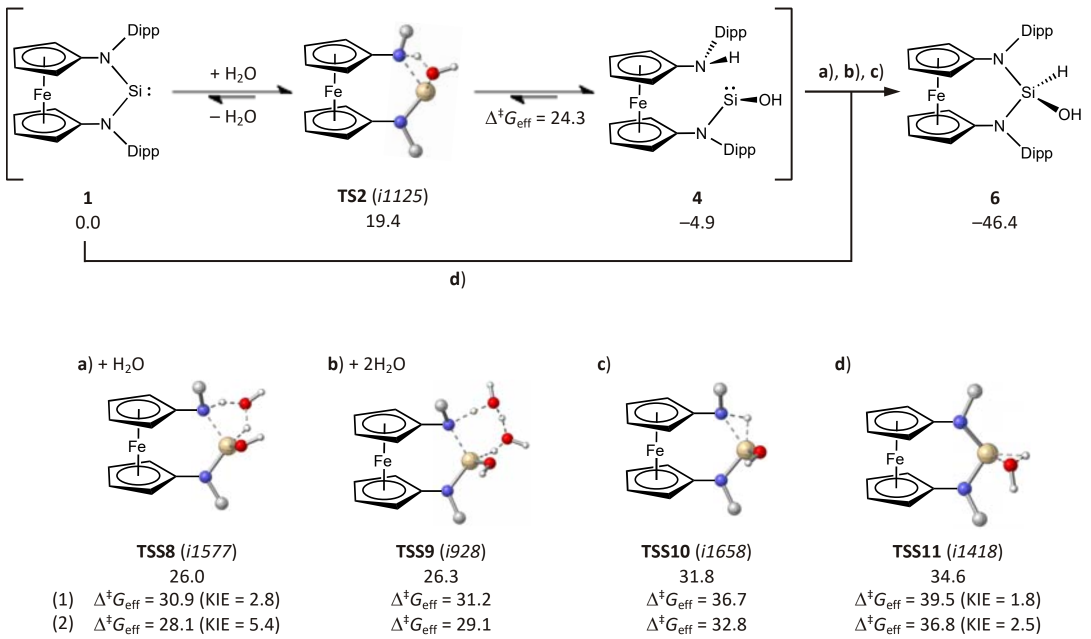

Scheme S4. Computed reaction paths for water addition by **1DippSi**, (1) effective reaction barriers relative to **4** and (2) including 1-D Wigner correction[S36] for tunneling effects.

Alternatively, the formation of silanone **5** from **4** through a proton shift and rearrangement to **6** was examined (Scheme S5, a-c).

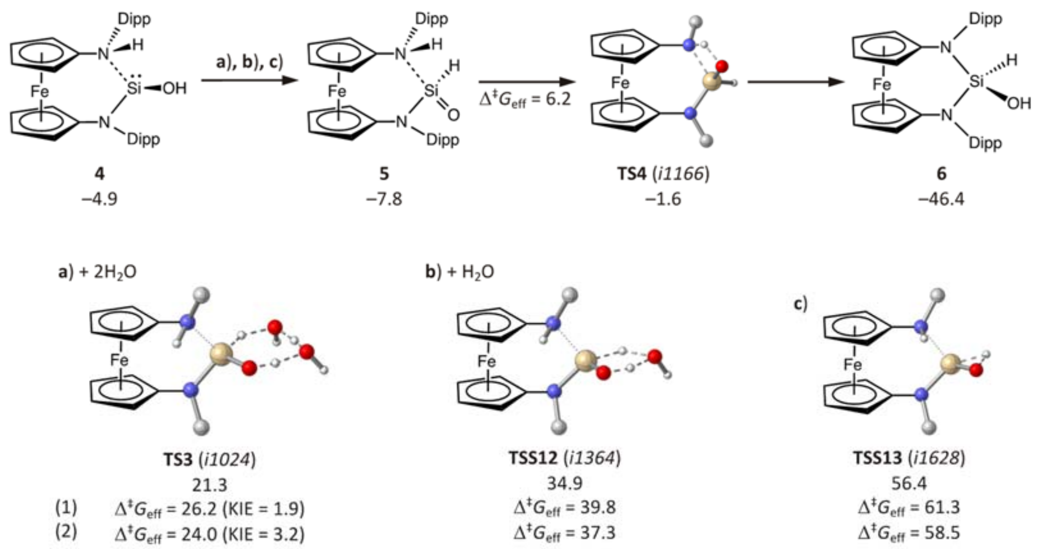

Scheme S5. Computed isomerization paths for **4** to **6**, (1) effective reaction barriers relative to **4** and (2) including 1-D Wigner correction[S36] for tunneling effects.

Overall the application of the 1-D Wigner correction lowers activation barriers by ca. 2-3 kcal mol^{-1} (Schemes S4 and S5). This is accompanied with an increase of the predicted KIEs (Scheme S4a).

As for the reaction with H_2O, two pathways, i.e. direct N–H bond insertion as well as N–H bond addition across an N−Si bond of **1**, were examined for ammonia and, as before, proton shuttling involving an additional NH_3 molecule reduces the activation barrier substantially (Scheme S6).

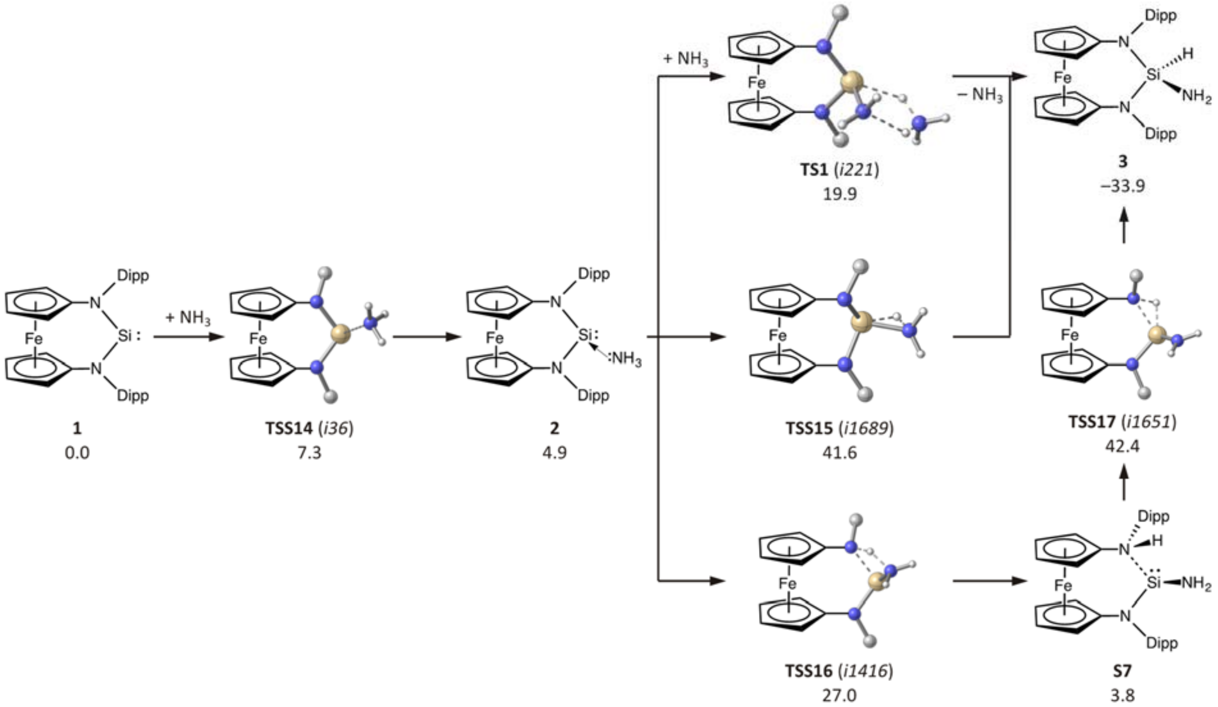

Scheme S6. Computed reaction paths for the N−H bond cleavage in NH_3 by **1** and subsequent hydrogen migration.

A reaction involving the silaimine **S8** can clearly be ruled out because of the very high computed activation barrier of 76 kcal mol^{-1} (Scheme S7).

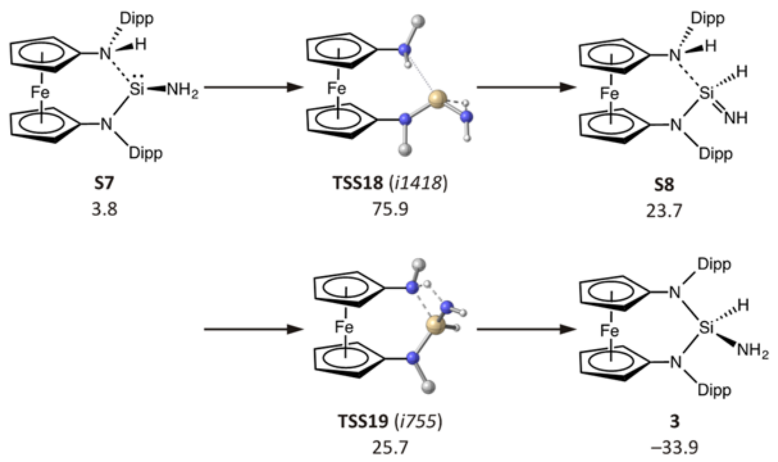

Scheme S7. Computed isomerization path for **S7** to **3**.

For the reaction of **1** with H_3BNH_3 both, insertion into an N−H or a B−H bond, are kinetically strongly disfavored (Scheme S8).

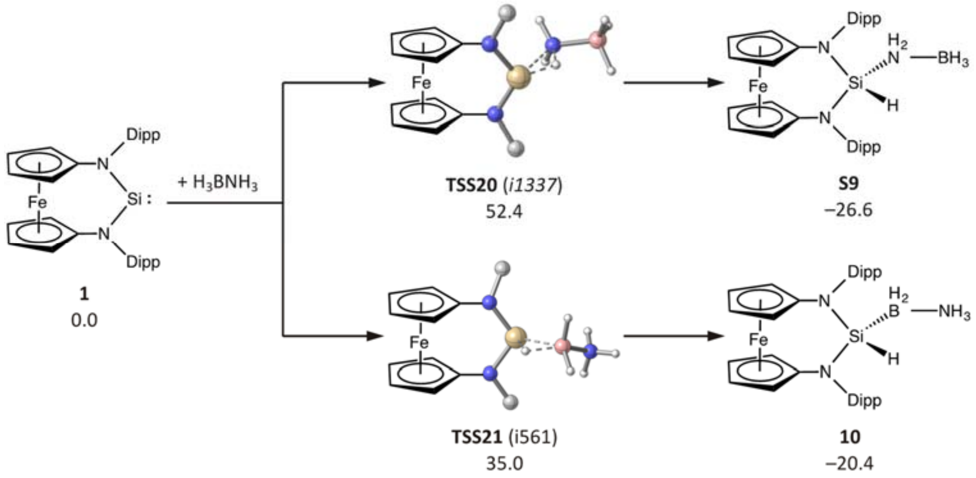

Scheme S8. Computed reaction paths for the direct B/N−H bond insertion in H_3BNH_3 by **1**.

In contrast to expectation silane **7** is not the primary product of the reaction of **1** with H_3BNH_3. Extrusion of H_2BNH_2 from **10** is hindered by an unreasonably high effective barrier of 47 kcal mol^{-1} (Scheme S9).

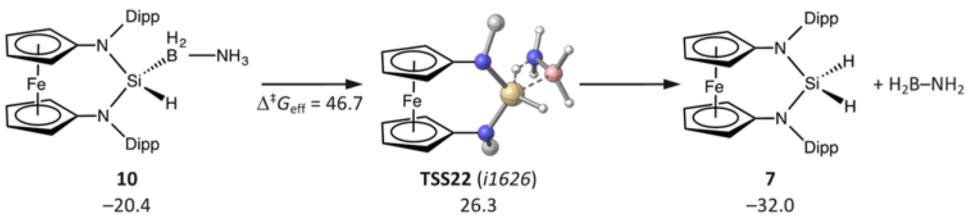

Scheme S9. Computed reaction path for the release of H_2BNH_2 from **10**, effective barrier relative to **10**.

Further, reaction of H_3BNH_3 with the partial Si−N double bond was examined (Scheme S10). For either orientation the computed barrier is too high for an efficient reaction at room temperature.

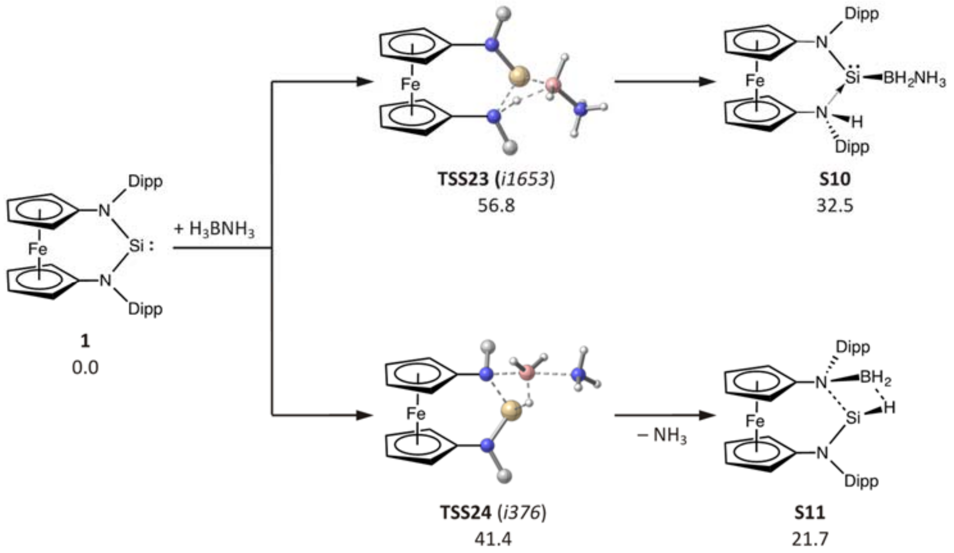

Scheme S10. Computed reaction paths for the B−H bond cleavage in H_3BNH_3 by the Si−N bond in **1**.

Alternatively, we investigated an S_N-like reaction of H_3BNH_3 with **1** (Scheme S11). After formation of the borane adduct **S12** reversible coordination of ammonia leads to the push-pull-stabilized silylene **S13**. Alternatively, the silylene can insert in a B–H bond in **S12** and subsequent coordination of ammonia leads to **10**. With reference to **S13**, this sequence has an effective barrier of 24 kcal mol^{-1} and cannot compete with the initial dehydrogenation scenario shown in the main text (Scheme 4).

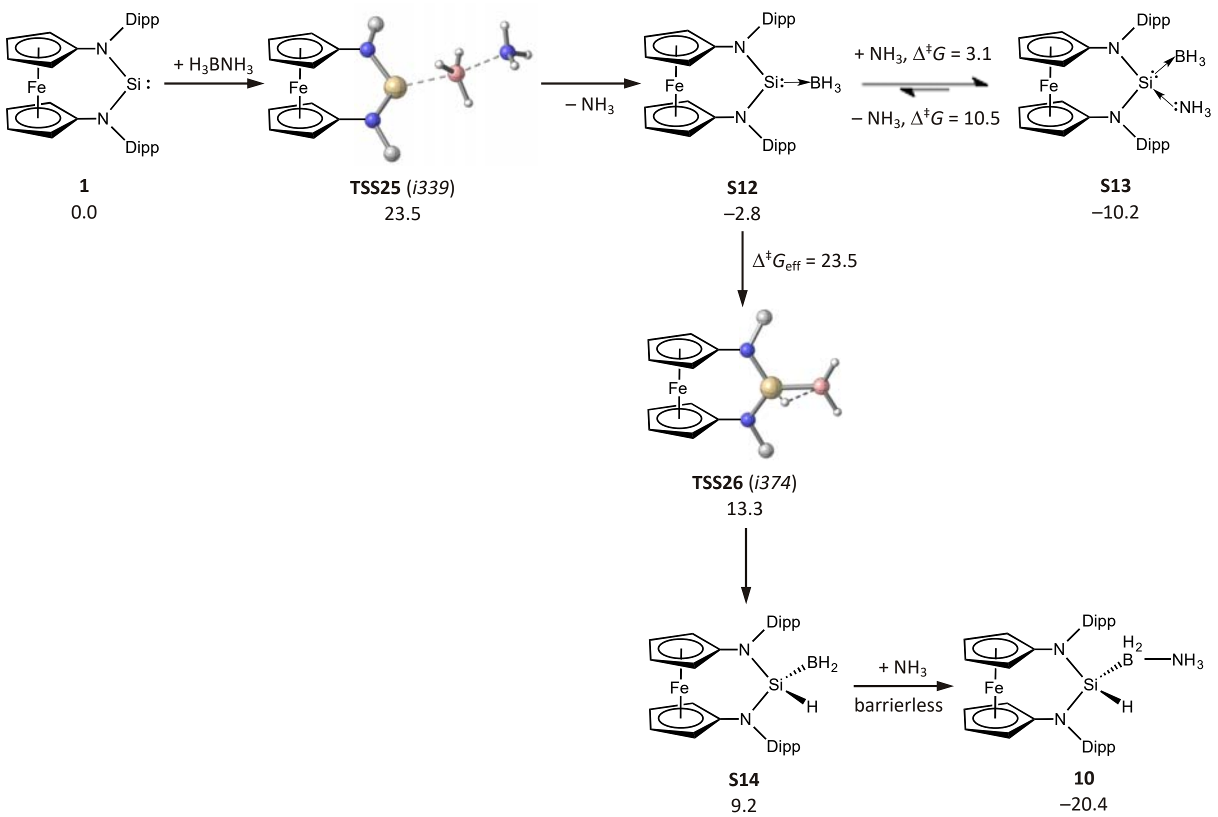

Scheme S11. Computed reaction paths for the S_N-like reaction of H_3BNH_3 with **1**.

Finally, following a reviewer's suggestion, we elucidated the role of the BH_3 adduct **S12** as resting state in the catalytic cycle sketched in Scheme S12: low-barrier B–H insertion leads to **S14** and NH_3 transfer from H_3BNH_3 yields the product **10**. The catalytic cycle is closed by regeneration of **S12** with another equivalent of **1** and the liberated BH_3. The rate-limiting ammonia transfer via **TSS27** is, however, connected with a prohibitively high activation barrier of 30 kcal mol^{-1}. Hence this scenario cannot compete with the initial dehydrogenation of H_3BNH_3 by **1** (Scheme 4 in the main text).

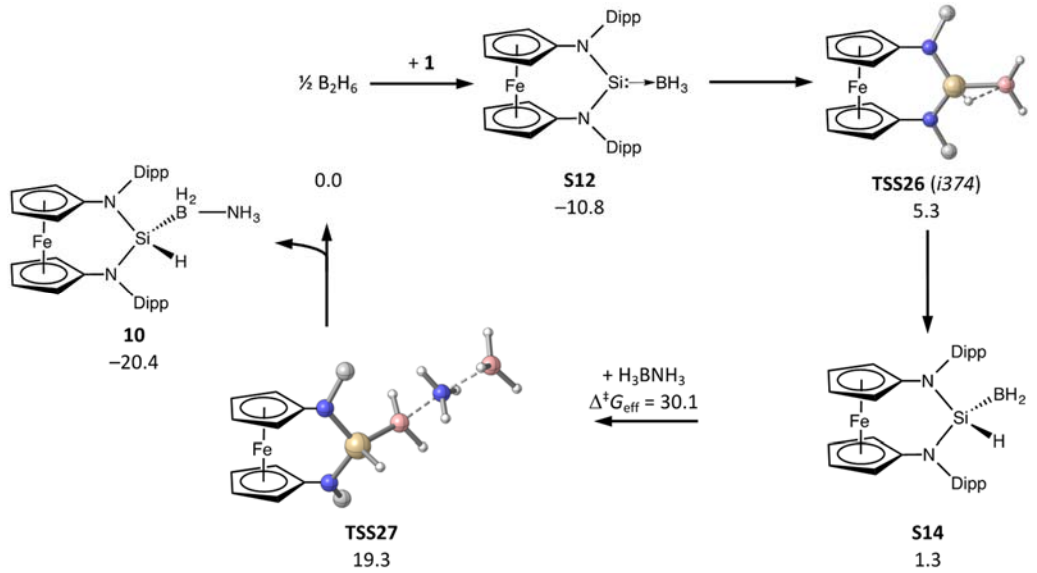

Scheme S12. Computed reaction path for the reaction of B_2H_6 with **1.**

Cartesian coordinates

Compound (point group): total energy/Hartree (PBEh-3c)

1 (C_2): E_{tot} = -2979.40853194

C	4.87836	17.81194	1.95428
C	3.99092	18.65654	1.22975
H	2.93130	18.76284	1.41570
C	4.74685	19.35782	0.25862
H	4.36163	20.08665	-0.43869
C	6.09282	18.92579	0.35392
H	6.91412	19.27030	-0.25638
C	6.17773	17.96968	1.39304
H	7.07257	17.45899	1.71659
C	4.95740	20.01912	4.07211
C	4.50328	21.12012	3.29029
H	3.47656	21.43371	3.17451
C	5.62899	21.71541	2.67521
H	5.60958	22.56533	2.00962
C	6.78342	20.99896	3.07638
H	7.79823	21.20806	2.77260
C	6.37553	19.96630	3.95557
H	7.02013	19.23931	4.42905
C	4.73190	15.59033	2.83797
C	3.76181	14.85549	2.14084
C	3.97823	13.49799	1.93618
H	3.24438	12.91525	1.39285
C	5.11062	12.87259	2.42510
H	5.25876	11.81296	2.26262
C	6.04945	13.60394	3.12814
H	6.92726	13.10191	3.51601
C	5.88583	14.96906	3.34042
C	2.49728	15.49060	1.60711
H	2.43347	16.50419	2.00338
C	2.54097	15.59044	0.08241
H	3.41135	16.15501	-0.25181
H	1.64904	16.09157	-0.29815
H	2.58616	14.60174	-0.37820
C	1.24463	14.75585	2.08296
H	1.18393	13.74147	1.68581
H	0.34834	15.28466	1.75467
H	1.21548	14.68980	3.17075
C	6.94184	15.72194	4.12430
H	6.74847	16.79103	4.01756
C	6.85817	15.38586	5.61413
H	5.86861	15.60486	6.01864
H	7.58944	15.96185	6.18492
H	7.05603	14.32735	5.79194
C	8.35183	15.46976	3.59103
H	8.67396	14.43973	3.74961
H	9.07062	16.11245	4.10174
H	8.42065	15.67180	2.52148
C	3.49673	19.70917	5.94153
C	2.16483	20.13695	5.83334
C	1.55822	20.70456	6.94891
H	0.52823	21.03414	6.89007
C	2.24733	20.85695	8.13762
H	1.76129	21.30800	8.99287
C	3.55574	20.42054	8.23298
H	4.08424	20.53105	9.17221
C	4.19827	19.83247	7.14956
C	1.35417	19.96485	4.56519
H	2.03434	19.68344	3.75933
C	0.65959	21.25685	4.13645
H	-0.11666	21.55994	4.84037
H	0.17808	21.12462	3.16631
H	1.36438	22.08501	4.05278
C	0.34169	18.82934	4.72408
H	0.83429	17.89001	4.98034
H	-0.21843	18.67502	3.79977
H	-0.37685	19.04785	5.51599
C	5.61731	19.33663	7.32000
H	5.91114	18.81908	6.40683
C	6.58768	20.49949	7.52317
H	6.53960	21.20369	6.69255
H	7.61446	20.13668	7.59832
H	6.36581	21.04888	8.43992
C	5.72192	18.31477	8.45188
H	5.49364	18.75288	9.42473
H	6.73437	17.91090	8.50704
H	5.03698	17.48130	8.29370
Fe	5.45522	19.71564	2.16061
N	4.53967	16.99393	3.05340
N	4.14424	19.12448	4.80349
Si	3.87163	17.40287	4.62251
N	1.44357	-0.01023	0.24715
N	-1.40790	-0.08869	0.13310
Si	-0.02833	0.13213	1.18945
H	0.01127	-0.88403	2.27677

2 (C_1): E_{tot} = -3035.85190114

C	-0.92607	1.11377	1.26004
C	0.17341	1.67804	1.97121
H	1.07929	2.06001	1.52292
C	-0.09835	1.60197	3.35816
H	0.55478	1.93254	4.15212
C	-1.37889	1.01831	3.52175
H	-1.87274	0.82409	4.46235
C	-1.89071	0.71842	2.23775
H	-2.83736	0.24009	2.04125
C	-1.74893	2.06222	-0.75867
C	-1.07238	3.21196	-1.21024
C	-1.80548	4.25974	-1.75873
H	-1.28829	5.15008	-2.09480
C	-3.17848	4.19736	-1.88369
H	-3.72883	5.02597	-2.30984
C	-3.83864	3.06161	-1.46111
H	-4.91612	3.00885	-1.56427
C	-3.14923	1.99117	-0.90111
C	0.42825	3.38940	-1.13043
H	0.87248	2.46068	-0.76842
C	1.02124	3.66112	-2.51278
H	0.62676	4.57777	-2.95345
H	0.80518	2.83881	-3.19471
H	2.10386	3.77378	-2.45294
C	0.79947	4.49830	-0.14467
H	1.88357	4.56399	-0.03107
H	0.36526	4.32054	0.83912
H	0.44814	5.47321	-0.48765
C	-3.97127	0.79662	-0.46899
H	-3.28703	0.04933	-0.06568
C	-4.72279	0.17385	-1.64947
H	-4.07462	-0.00291	-2.51067
H	-5.52020	0.82815	-2.00386
H	-5.18684	-0.77301	-1.36582
C	-4.96292	1.16422	0.63698
H	-5.47111	0.27556	1.01753
H	-5.73212	1.84479	0.26897
H	-4.46681	1.65765	1.47149
Fe	-0.13790	-0.26542	2.48997
N	-1.02847	0.98307	-0.14423
Si	-0.09397	-0.07656	-1.27289
C	0.68102	-1.50883	1.14845
C	-0.44417	-2.13427	1.76278
H	-1.34068	-2.47823	1.26688
C	-0.21060	-2.20757	3.15893
H	-0.88833	-2.61504	3.89437
C	1.07198	-1.66539	3.41519
H	1.54351	-1.58229	4.38297
C	1.62288	-1.24002	2.18413
H	2.58548	-0.77115	2.04677
C	1.99749	-1.80003	-0.80027
C	1.93868	-3.15886	-1.15774
C	3.05291	-3.76171	-1.72840
H	3.01457	-4.81000	-2.00068
C	4.21283	-3.04620	-1.95902
H	5.07027	-3.52597	-2.41261
C	4.26920	-1.71461	-1.59706
H	5.18357	-1.15995	-1.77074
C	3.18399	-1.07593	-1.00421
C	0.70738	-4.00489	-0.92121
H	-0.08000	-3.35201	-0.54947
C	0.20079	-4.65180	-2.21100
H	0.90491	-5.38578	-2.60466
H	0.04545	-3.91216	-2.99999
H	-0.74221	-5.17499	-2.03860
C	0.95704	-5.05084	0.16440
H	0.05236	-5.62868	0.36396
H	1.26394	-4.57604	1.09602
H	1.73941	-5.75282	-0.12938
C	3.34451	0.37347	-0.60165
H	2.44093	0.68207	-0.07116
C	3.49633	1.26675	-1.83031
H	2.63296	1.17426	-2.48843
H	4.38794	1.00587	-2.40352
H	3.59055	2.31357	-1.53602
C	4.52293	0.57318	0.35306
H	4.55113	1.60361	0.71145
H	5.47908	0.37414	-0.13342
H	4.46081	-0.08210	1.22232
N	0.85526	-1.17543	-0.20754
N	-1.70187	-1.50301	-1.53444
H	-2.42683	-1.07375	-2.09701
H	-2.13238	-1.84096	-0.68112
H	-1.34043	-2.29958	-2.04743

3 (C_1): E_{tot} = -3035.91032832349

C	1.63675	-1.12846	-0.58265
C	2.15691	-1.09973	-1.90638
H	2.55238	-0.22916	-2.40706
C	2.06468	-2.40597	-2.44463
H	2.37483	-2.70464	-3.43464
C	1.48604	-3.24723	-1.46434
H	1.28035	-4.30179	-1.57182
C	1.24437	-2.46828	-0.30462
H	0.80926	-2.82310	0.61970
C	-1.37435	-0.67154	-1.15545
C	-0.91909	-0.05780	-2.35659
H	-0.53453	0.94798	-2.44487
C	-1.00367	-1.00895	-3.40218
H	-0.71462	-0.84752	-4.43001
C	-1.54418	-2.20385	-2.86566
H	-1.73642	-3.11486	-3.41251
C	-1.77356	-2.00162	-1.48642
H	-2.16556	-2.73349	-0.79729
C	2.58320	0.81698	0.45775
C	3.65752	0.32515	1.21868
C	4.80468	1.10067	1.33913
H	5.64667	0.72534	1.90799
C	4.88965	2.34919	0.75117
H	5.79050	2.94010	0.85358
C	3.81195	2.84151	0.03982
H	3.88119	3.82512	-0.40915
C	2.64840	2.09530	-0.11907
C	3.60962	-1.02596	1.89990
H	2.58490	-1.39561	1.85512
C	3.97482	-0.93257	3.38142

H	3.34779	-0.20747	3.90110
H	5.01501	-0.64163	3.53543
H	3.83717	-1.90090	3.86505
C	4.50115	-2.03650	1.17761
H	4.42775	-3.01989	1.64535
H	5.54902	-1.73120	1.21015
H	4.21557	-2.14323	0.13148
C	1.51155	2.69485	-0.91800
H	0.64776	2.03560	-0.82552
C	1.85084	2.78639	-2.40571
H	2.07028	1.80718	-2.82932
H	2.72028	3.42310	-2.57802
H	1.01572	3.21000	-2.96757
C	1.10805	4.07068	-0.38731
H	1.88104	4.81937	-0.56637
H	0.92423	4.05194	0.68705
H	0.20065	4.42111	-0.88155
C	-2.71605	0.18817	0.64259
C	-3.34534	1.39465	0.29167
C	-4.63140	1.64348	0.75459
H	-5.13203	2.56329	0.47751
C	-5.28501	0.74061	1.57250
H	-6.28455	0.95209	1.92948
C	-4.65031	-0.43099	1.93395
H	-5.16395	-1.13063	2.58237
C	-3.37103	-0.73338	1.47584
C	-2.67759	2.42993	-0.58557
H	-1.62928	2.14869	-0.69765
C	-3.30252	2.45072	-1.98075
H	-4.35019	2.75469	-1.93851
H	-3.25946	1.46746	-2.44818
H	-2.77995	3.15611	-2.63025
C	-2.70939	3.82107	0.04724
H	-2.13847	4.52924	-0.55546
H	-2.28508	3.81297	1.05216
H	-3.72347	4.21503	0.12614
C	-2.75124	-2.04289	1.91719
H	-1.79312	-2.16019	1.40876
C	-2.47511	-2.03877	3.42136
H	-3.40075	-1.97165	3.99579
H	-1.84701	-1.19597	3.70761
H	-1.96550	-2.95550	3.72361
C	-3.62262	-3.24236	1.54010
H	-3.10274	-4.17620	1.76082
H	-3.88066	-3.24039	0.48088
H	-4.55940	-3.25503	2.09919
Fe	0.20227	-1.68985	-1.87487
N	-0.17633	1.62879	1.99581
H	-0.34554	2.49974	1.52344
H	0.17024	1.76005	2.92959
N	1.44357	-0.01023	0.24715
N	-1.40790	-0.08869	0.13310
Si	-0.02833	0.13213	1.18945
H	0.01127	-0.88403	2.27677

4 (C_1): E_{tot} = -3055.69513045

C	1.31963	-0.59853	1.21906
C	0.89826	-1.95508	1.18042
H	0.61688	-2.51018	0.29644
C	0.89982	-2.45565	2.50622
H	0.62564	-3.45686	2.80371
C	1.33159	-1.41547	3.36356
H	1.44122	-1.47844	4.43587
C	1.57678	-0.26585	2.57455
H	1.90476	0.69367	2.94234
C	2.85769	0.56762	-0.21617
C	3.58619	-0.30058	-1.04782
C	4.90839	0.01198	-1.34210
H	5.48509	-0.64970	-1.97646
C	5.50924	1.14547	-0.82852
H	6.54157	1.36914	-1.06344
C	4.77990	1.99206	-0.01723
H	5.25092	2.88608	0.37255
C	3.45103	1.72763	0.29988
C	3.01660	-1.58657	-1.61488
H	1.93802	-1.61590	-1.44485
C	3.21175	-1.69094	-3.12722
H	4.26359	-1.78188	-3.40054
H	2.80965	-0.81937	-3.64387
H	2.70298	-2.57539	-3.51412
C	3.61046	-2.79877	-0.89648
H	3.16484	-3.72435	-1.26589
H	3.43745	-2.74556	0.17792
H	4.68780	-2.86174	-1.05886
C	2.70207	2.73477	1.14478
H	1.71505	2.33405	1.37502
C	2.47657	4.02462	0.35661
H	1.96001	3.82541	-0.58287
H	3.41920	4.51984	0.11543
H	1.86381	4.72309	0.92830
C	3.41243	3.02847	2.46606
H	2.79318	3.67071	3.09412
H	4.36248	3.54417	2.31686
H	3.62576	2.11591	3.02372
Fe	-0.37600	-0.85905	2.36036
N	1.48852	0.27315	0.11264
Si	-0.68280	2.11176	-0.04638
C	-1.89529	0.17536	1.42971
C	-1.58203	0.77706	2.67958
H	-1.24857	1.79332	2.83093
C	-1.77898	-0.19233	3.69567
H	-1.62600	-0.03563	4.75319
C	-2.22697	-1.38600	3.08340
H	-2.47355	-2.30819	3.58803
C	-2.28977	-1.16514	1.68525
H	-2.60477	-1.88092	0.94069
C	-2.84001	0.40489	-0.75718
C	-4.09736	1.01750	-0.62340
C	-5.11537	0.64722	-1.49391
H	-6.09293	1.10379	-1.39848
C	-4.90392	-0.29289	-2.48606
H	-5.70674	-0.56436	-3.15911
C	-3.66163	-0.88415	-2.61079
H	-3.50106	-1.62011	-3.38970
C	-2.61781	-0.55666	-1.75139
C	-4.38872	2.04049	0.45415
H	-3.44428	2.34510	0.90772
C	-5.03442	3.30559	-0.10908
H	-6.03260	3.11936	-0.50833
H	-4.43188	3.73551	-0.90963
H	-5.13751	4.05736	0.67492
C	-5.24549	1.42743	1.56269
H	-5.42418	2.15284	2.35830
H	-4.75693	0.55970	2.00608
H	-6.21675	1.10739	1.18010
C	-1.28859	-1.25346	-1.92565
H	-0.65406	-0.93324	-1.09891
C	-0.61924	-0.85148	-3.23939
H	-0.49433	0.22755	-3.30016
H	-1.21477	-1.16877	-4.09725
H	0.36236	-1.32039	-3.33691
C	-1.41132	-2.77350	-1.82608
H	-0.42607	-3.24109	-1.89326
H	-2.01731	-3.18808	-2.63285
H	-1.86222	-3.08203	-0.88270
N	-1.80771	0.76992	0.15879
O	-0.78885	2.23083	-1.71436
H	1.02190	-0.11346	-0.69608
H	-0.31704	2.98495	-2.07386

5 (C_1): E_{tot} = -3055.70217014

C	0.45467	3.92981	3.40508
C	-0.17343	3.77665	2.13499
H	0.26981	4.00451	1.17714
C	-1.46466	3.23518	2.34229
H	-2.18095	2.99455	1.57068
C	-1.65780	3.07947	3.73647
H	-2.54696	2.69760	4.21561
C	-0.47880	3.50358	4.39297
H	-0.31056	3.50672	5.46002
C	1.89656	5.56157	4.40397
C	2.09636	5.51834	5.78886
C	2.15344	6.71504	6.49471
H	2.31300	6.70064	7.56582
C	2.02312	7.93039	5.84906
H	2.06707	8.85295	6.41316
C	1.85746	7.96181	4.47714
H	1.78475	8.91837	3.97387
C	1.79742	6.78885	3.73334
C	2.34540	4.22009	6.51998
H	2.03244	3.40240	5.86430
C	3.84457	4.07072	6.78155
H	4.41098	4.07534	5.84862
H	4.05754	3.14608	7.32003
H	4.21339	4.89563	7.39358
C	1.54195	4.08737	7.81148
H	1.85393	4.80993	8.56659
H	1.68083	3.09534	8.24510
H	0.47430	4.23374	7.64120
C	1.69928	6.88389	2.22665
H	1.57127	5.87976	1.82239
C	3.00551	7.43289	1.64968
H	3.85384	6.81774	1.95173
H	3.19418	8.45296	1.98913
H	2.96758	7.44954	0.55873
C	0.49011	7.69804	1.77234
H	0.41758	7.69580	0.68347
H	0.55460	8.74032	2.08885
H	-0.43625	7.28473	2.17205
Fe	-0.02599	1.98493	3.11116
N	1.77319	4.34544	3.66263
Si	3.12523	3.70173	2.75232
C	1.76534	1.06397	3.18958
C	1.14701	0.78567	1.94258
H	1.52186	1.03913	0.96376
C	-0.07437	0.12489	2.21283
H	-0.78031	-0.21395	1.46988
C	-0.21387	-0.00397	3.61575
H	-1.04321	-0.46006	4.13476
C	0.92681	0.56986	4.22506
H	1.11484	0.63737	5.28710
C	4.18760	0.86291	3.25595
C	4.75781	0.63337	1.99868
C	5.83552	-0.24244	1.91887
H	6.29625	-0.42020	0.95591
C	6.33619	-0.88312	3.03198
H	7.17358	-1.56216	2.94339
C	5.75982	-0.64392	4.26165
H	6.15350	-1.14922	5.13373
C	4.68583	0.22762	4.40523
C	4.31797	1.28535	0.70666
H	3.41675	1.86382	0.88398
C	5.37677	2.27168	0.21264
H	6.30865	1.76281	-0.04040
H	5.59237	3.02364	0.97094
H	5.02652	2.78294	-0.68570
C	3.96707	0.25018	-0.36259
H	3.55678	0.74646	-1.24346
H	3.22873	-0.46731	-0.00212
H	4.83984	-0.31464	-0.69230
C	4.13060	0.38450	5.81059
H	3.40069	1.20027	5.85602
C	5.22330	0.75631	6.81343
H	5.76816	1.64484	6.49832
H	5.94411	-0.05066	6.94530
H	4.78500	0.95523	7.79229
C	3.40598	-0.89100	6.24662
H	2.93494	-0.75422	7.22161

H	4.10578	-1.72318	6.33420
H	2.63776	-1.18632	5.53278
N	3.02104	1.74783	3.40018
H	3.01832	2.03064	4.37948
O	4.54973	4.16132	3.09317
H	2.61001	3.51736	1.37050

6 (C_1): E_{tot} = -3055.76098406

H	-2.21006	-2.60860	1.02314
C	-1.84037	-1.81895	1.65750
C	-1.43037	-0.52163	1.22715
H	-1.84553	-2.76924	3.66937
C	-0.99737	0.18868	2.38268
C	-1.10625	-0.67266	3.50069
H	-0.83715	-0.42793	4.51740
C	-1.64027	-1.90633	3.05360
Fe	0.12495	-1.48435	2.05771
H	0.74978	-2.83593	-0.31675
C	1.17971	-2.41616	0.58239
C	1.38883	-3.09177	1.81038
H	1.16084	-4.12843	2.00876
C	1.96233	-2.17521	2.72547
H	2.24710	-2.39041	3.74439
C	2.08704	-0.92373	2.07653
C	1.59186	-1.06517	0.75005
H	2.47865	-0.01556	2.50934
H	-0.61388	1.19871	2.39594
N	1.44877	-0.01965	-0.18099
N	-1.42902	-0.05192	-0.10712
Si	-0.02090	0.25442	-1.07327
C	2.63744	0.68414	-0.54251
C	4.96193	2.04332	-1.23971
C	2.87772	1.96444	-0.01975
C	3.56194	0.08252	-1.41312
C	4.72107	0.77673	-1.73994
C	4.04246	2.62893	-0.39062
H	5.44912	0.32227	-2.40078
H	4.23897	3.62039	-0.00037
H	5.86655	2.57210	-1.51036
C	-2.70182	0.33077	-0.64704
C	-5.18743	1.08963	-1.65237
C	-3.16411	1.64512	-0.45616
C	-3.48075	-0.59870	-1.35816
C	-4.71744	-0.19365	-1.85068
C	-4.41124	1.99836	-0.95994
H	-5.32858	-0.90006	-2.39919
H	-4.78472	3.00349	-0.80762
H	-6.15561	1.38158	-2.03803
C	1.92687	2.64732	0.93830
H	1.10884	1.95649	1.14757
C	2.60387	2.97130	2.27069
H	3.05480	2.08762	2.72227
H	1.87938	3.37632	2.97925
H	3.39433	3.71428	2.15343
C	1.32783	3.91145	0.32446
H	0.81108	3.69501	-0.60980
H	2.09592	4.65771	0.11394
H	0.60794	4.36652	1.00742
C	3.35236	-1.30548	-1.97927
H	2.32857	-1.61270	-1.77188
C	4.27862	-2.31111	-1.29505
H	4.09697	-3.32028	-1.66893
H	4.12014	-2.32162	-0.21688
H	5.32803	-2.07224	-1.47834
C	3.52453	-1.34308	-3.49765
H	3.26034	-2.32842	-3.88414
H	4.55151	-1.14148	-3.80527
H	2.88923	-0.60700	-3.99387
C	-2.37667	2.69548	0.29751
H	-1.37305	2.31000	0.48337
C	-3.02094	2.98475	1.65392
H	-3.12485	2.07637	2.24685
H	-4.01559	3.41822	1.53448
H	-2.41883	3.69478	2.22479
C	-2.21706	3.97915	-0.51640
H	-1.74522	3.78201	-1.47937
H	-1.59488	4.69611	0.02036
H	-3.17382	4.46589	-0.70969
C	-3.05899	-2.03073	-1.60824
H	-2.06937	-2.17952	-1.17660
C	-4.02899	-3.01151	-0.94351
H	-3.65587	-4.03440	-1.02336
H	-5.01037	-2.98784	-1.42046
H	-4.18102	-2.78632	0.11215
C	-2.94988	-2.33295	-3.10291
H	-2.57181	-3.34491	-3.25857
H	-2.27074	-1.64135	-3.59519
H	-3.92002	-2.26863	-3.59896
H	0.04337	1.65957	-1.54695
O	-0.14745	-0.72512	-2.41271
H	0.38857	-0.47555	-3.16825

7 (C_1): E_{tot} = -2980.63295189

C	1.41677	-0.70335	-1.17879
C	0.97108	-0.11509	-2.39614
H	0.60904	0.89620	-2.51254
C	1.03822	-1.09578	-3.41477
H	0.75200	-0.95754	-4.44675
C	1.55941	-2.28404	-2.84599
H	1.73685	-3.21253	-3.36789
C	1.79250	-2.04873	-1.47243
H	2.16920	-2.76939	-0.76359
C	2.71249	0.21893	0.66038
C	3.31742	1.44909	0.34525
C	4.56083	1.74342	0.89131
H	5.04730	2.67886	0.64440
C	5.18989	0.86438	1.75360
H	6.15600	1.11223	2.17367
C	4.57605	-0.32872	2.08041
H	5.07159	-1.00779	2.76376
C	3.33999	-0.67664	1.54357
C	2.67204	2.44784	-0.58968
H	1.62868	2.15730	-0.72489
C	2.66896	3.86309	-0.01417
H	3.67568	4.26802	0.09807
H	2.18584	3.89425	0.96259
H	2.12591	4.53916	-0.67589
C	3.34946	2.41506	-1.96050
H	2.84929	3.09160	-2.65649
H	3.32900	1.41421	-2.39102
H	4.39354	2.72620	-1.88982
C	2.74185	-2.01075	1.93778
H	1.79353	-2.13062	1.41100
C	2.44446	-2.07115	3.43659
H	1.78747	-1.25932	3.74628
H	3.35777	-2.00413	4.03014
H	1.95664	-3.01316	3.69321
C	3.65319	-3.17131	1.53244
H	3.15949	-4.12792	1.71232
H	4.58082	-3.17274	2.10695
H	3.92893	-3.12389	0.47894
Fe	-0.17824	-1.71859	-1.86988
N	1.43826	-0.09574	0.09517
Si	0.04729	0.26894	1.08992
C	-1.62219	-1.12984	-0.59014
C	-1.23903	-2.46637	-0.29328
H	-0.81423	-2.81013	0.64020
C	-1.47864	-3.25695	-1.44555
H	-1.28124	-4.31439	-1.53929
C	-2.04213	-2.42236	-2.44109
H	-2.34567	-2.73076	-3.43019
C	-2.12709	-1.10897	-1.91970
H	-2.50394	-0.23786	-2.43445
C	-2.58662	0.82027	0.45724
C	-3.60438	0.36226	1.31027
C	-4.73144	1.15570	1.48828
H	-5.53242	0.81164	2.13093
C	-4.84606	2.38608	0.86674
H	-5.72793	2.99376	1.02232
C	-3.82689	2.83735	0.05000
H	-3.92129	3.80447	-0.42933
C	-2.68994	2.06806	-0.17609
C	-3.51952	-0.97409	2.01561
H	-2.49338	-1.33570	1.93842
C	-3.83358	-0.86388	3.50710
H	-4.87246	-0.58791	3.69379
H	-3.20000	-0.12228	3.99418
H	-3.66420	-1.82314	3.99847
C	-4.42684	-2.00152	1.33721
H	-4.33188	-2.97735	1.81680
H	-4.17476	-2.12212	0.28386
H	-5.47480	-1.70135	1.39915
C	-1.61904	2.60747	-1.09807
H	-0.82699	1.86069	-1.16544
C	-1.00200	3.89312	-0.54946
H	-0.58146	3.74232	0.44440
H	-1.73873	4.69523	-0.48027
H	-0.19973	4.24260	-1.20168
C	-2.15197	2.83074	-2.51354
H	-1.34820	3.14800	-3.18049
H	-2.92077	3.60464	-2.53927
H	-2.58988	1.92292	-2.92832
N	-1.44432	-0.00224	0.23013
H	0.11066	-0.55443	2.32975
H	0.05758	1.69100	1.51150

8 (C_1): E_{tot} = -3061.30468923

C	-0.72430	4.05064	4.66527
C	-1.72747	4.29327	3.68635
H	-1.64452	4.97937	2.85687
C	-2.84043	3.47038	3.98206
H	-3.75948	3.42282	3.41729
C	-2.53320	2.71224	5.13770
H	-3.17842	1.98841	5.61303
C	-1.23759	3.08212	5.57499
H	-0.71537	2.67732	6.42971
C	0.67554	6.01082	4.72914
C	0.50462	6.65043	5.96773
C	0.56898	8.03754	6.01907
H	0.42809	8.54522	6.96569
C	0.81931	8.78462	4.88219
H	0.87185	9.86405	4.94068
C	1.00629	8.14418	3.67240
H	1.20850	8.73481	2.78672
C	0.93177	6.75815	3.57138
C	0.24314	5.87858	7.24255
H	0.38814	4.81801	7.03706
C	1.23176	6.24899	8.34738
H	1.07720	5.61503	9.22197
H	1.11900	7.28368	8.67493
H	2.26201	6.11669	8.01515
C	-1.20300	6.06301	7.70187
H	-1.41363	7.10709	7.94190
H	-1.40355	5.47048	8.59638
H	-1.90412	5.74927	6.92849
C	1.14502	6.11411	2.22029
H	0.97386	5.04123	2.32903
C	2.58349	6.31318	1.74719
H	3.29779	5.86819	2.44071
H	2.83075	7.37212	1.65369
H	2.73741	5.85302	0.77027
C	0.16137	6.63769	1.17366
H	0.32730	7.69284	0.95034
H	-0.87284	6.53633	1.50321
H	0.26994	6.08759	0.23721
Fe	-1.13499	2.34153	3.67129
N	0.57519	4.59167	4.65384
Si	1.93678	3.51371	4.89297
C	0.71238	1.67936	3.17164

C	0.01675	2.11826	2.00849
H	0.32360	2.92630	1.36050
C	-1.15960	1.34324	1.87289
H	-1.90034	1.44546	1.09377
C	-1.19429	0.40901	2.93836
H	-1.96759	-0.32402	3.11409
C	-0.04697	0.61098	3.73806
H	0.20100	0.06100	4.63325
C	3.10528	1.47482	3.43267
C	3.81947	1.67112	2.23285
C	4.98515	0.94422	2.01402
H	5.53330	1.07905	1.09015
C	5.46568	0.05240	2.95495
H	6.37927	-0.49739	2.76977
C	4.76671	-0.13169	4.13058
H	5.14478	-0.83180	4.86568
C	3.58191	0.55417	4.38529
C	3.35582	2.62651	1.15644
H	2.59251	3.27396	1.59199
C	4.48147	3.52737	0.64946
H	5.27066	2.96380	0.14998
H	4.93516	4.09760	1.46020
H	4.09497	4.24002	-0.07981
C	2.73207	1.85088	-0.00594
H	3.47998	1.23248	-0.50637
H	2.31740	2.53355	-0.75056
H	1.93190	1.19323	0.33093
C	2.87332	0.26144	5.69111
H	1.94219	0.82514	5.71575
C	3.70697	0.72034	6.88747
H	3.95366	1.78012	6.81747
H	4.64489	0.16683	6.96048
H	3.16036	0.56722	7.81932
C	2.51074	-1.21929	5.81211
H	1.91615	-1.39279	6.71076
H	3.39546	-1.85387	5.88288
H	1.93281	-1.56020	4.95269
N	1.91549	2.22667	3.67803
N	4.87082	4.05719	4.48785
H	4.98033	3.16704	4.02828
H	5.73343	4.56660	4.58098
B	3.66419	4.54934	4.96047
H	3.67641	5.61884	5.49983
H	1.72843	2.89098	6.24333

9 (C_1): E_{tot} = -3144.33725214

C	0.67310	3.99949	3.24104
C	0.35313	3.62388	1.90538
H	1.02548	3.67898	1.06169
C	-0.97481	3.13493	1.88264
H	-1.49940	2.76274	1.01517
C	-1.49754	3.22763	3.19663
H	-2.48972	2.93491	3.50683
C	-0.49041	3.75828	4.03364
H	-0.58328	3.93259	5.09447
C	1.96013	5.90920	3.91624
C	1.64457	6.43844	5.17941
C	1.65924	7.81868	5.35464
H	1.42200	8.23607	6.32595
C	1.96735	8.67122	4.31281
H	1.96178	9.74292	4.46403
C	2.29618	8.14241	3.07936
H	2.55344	8.81336	2.26863
C	2.31513	6.76919	2.86416
C	1.32376	5.57551	6.38164
H	1.25339	4.53840	6.05619
C	2.45364	5.64065	7.41009
H	3.40589	5.34293	6.96991
H	2.24954	4.97570	8.25164
H	2.57619	6.65038	7.80610
C	-0.01440	5.94777	7.02019
H	0.00741	6.94259	7.46771
H	-0.26736	5.24318	7.81467
H	-0.82302	5.93841	6.28878
C	2.73852	6.25828	1.50641
H	2.81219	5.17160	1.56912
C	4.12035	6.78781	1.12232
H	4.87083	6.51100	1.86403
H	4.13057	7.87502	1.03066
H	4.43424	6.38157	0.15967
C	1.70258	6.60034	0.43591
H	1.98369	6.16670	-0.52646
H	1.61665	7.67958	0.29512
H	0.71507	6.22288	0.70161
Fe	0.18966	2.03418	3.17742
N	1.91961	4.49767	3.68587
Si	3.21050	3.45913	4.29473
C	1.97697	1.15259	3.49987
C	1.32571	0.53208	2.39579
H	1.69500	0.48965	1.38356
C	0.09952	-0.00911	2.85119
H	-0.62129	-0.53732	2.24519
C	-0.02028	0.27511	4.23212
H	-0.84786	-0.00025	4.86889
C	1.14375	0.97078	4.64145
H	1.34658	1.33550	5.63735
C	4.32016	1.25046	2.93489
C	4.69526	1.40984	1.59001
C	5.85762	0.78965	1.13855
H	6.16833	0.92359	0.10951
C	6.61120	-0.01641	1.97221
H	7.50111	-0.50650	1.59804
C	6.20607	-0.21255	3.28366
H	6.78390	-0.87010	3.92172
C	5.07142	0.41729	3.78674
C	3.85007	2.17946	0.59848
H	2.95026	2.50705	1.12064
C	4.55366	3.42335	0.06699
H	5.46559	3.17003	-0.47727
H	4.83155	4.10092	0.86937
H	3.89905	3.96367	-0.62014
C	3.42543	1.28621	-0.57037
H	2.69161	1.80058	-1.19297
H	2.98447	0.34651	-0.23735
H	4.27192	1.03356	-1.21132
C	4.63877	0.13779	5.20997
H	3.93141	0.91310	5.50885
C	5.79388	0.17121	6.20962
H	6.35065	1.10772	6.15630
H	6.50053	-0.64613	6.05746
H	5.41126	0.07137	7.22631
C	3.90919	-1.20592	5.27897
H	3.54227	-1.39480	6.28938
H	4.57647	-2.02635	5.00701
H	3.05434	-1.23061	4.60454
N	3.16603	1.90881	3.43798
B	4.99342	4.41322	4.46174
H	2.85940	3.07645	5.71307
H	4.90415	5.60557	4.36736
N	5.80399	3.87915	5.61102
H	6.25390	4.59563	6.16190
H	5.30519	3.26281	6.23554
B	6.86371	4.19067	2.94787
N	7.46788	2.95364	3.71562
H	6.86566	4.04176	1.76097
H	7.34311	5.21039	3.35436
H	5.59673	4.11251	3.22072
H	7.02791	3.04895	4.67266
H	7.20532	2.05958	3.30729
H	8.47686	2.99578	3.78748

10 (C_1): E_{tot} = -3062.49096653

C	4.76031	17.74520	1.80320
C	3.87408	18.57946	1.06363
H	2.80924	18.66558	1.22272
C	4.63772	19.30824	0.11840
H	4.25116	20.03193	-0.58386
C	5.98896	18.90557	0.24182
H	6.81778	19.27110	-0.34588
C	6.06857	17.94755	1.28082
H	6.96718	17.46142	1.62638
C	4.78666	20.04504	3.95688
C	4.34054	21.11619	3.12190
H	3.31811	21.42685	2.97339
C	5.46860	21.69168	2.49415
H	5.45300	22.51690	1.79766
C	6.62053	20.98896	2.92663
H	7.63701	21.18565	2.61965
C	6.20499	19.98675	3.83523
H	6.84586	19.27104	4.32940
C	4.76994	15.57168	2.84758
C	3.91220	14.69475	2.16278
C	4.25477	13.35087	2.07585
H	3.60574	12.66601	1.54347
C	5.41055	12.86862	2.66232
H	5.66029	11.81787	2.59071
C	6.24504	13.73698	3.33912
H	7.14919	13.35271	3.79647
C	5.94926	15.09320	3.43827
C	2.64280	15.17380	1.49246
H	2.44947	16.19722	1.81408
C	2.81350	15.19798	-0.02674
H	3.64841	15.83545	-0.31803
H	1.91390	15.58197	-0.51170
H	3.00197	14.19742	-0.42115
C	1.42533	14.34786	1.90412
H	1.48672	13.31545	1.55585
H	0.51631	14.77700	1.47898
H	1.31499	14.32880	2.98867
C	6.91800	15.99248	4.17359
H	6.54483	17.01538	4.09553
C	6.99375	15.63387	5.65659
H	6.01207	15.68185	6.12434
H	7.65863	16.32111	6.18461
H	7.38380	14.62489	5.80382
C	8.31756	15.94672	3.55714
H	8.78774	14.97252	3.70174
H	8.96632	16.69120	4.02237
H	8.30336	16.13787	2.48428
C	3.45279	19.62687	5.94768
C	2.17054	20.20982	5.99692
C	1.63038	20.56239	7.23268
H	0.64469	21.01071	7.27573
C	2.33113	20.36348	8.40593
H	1.89926	20.64892	9.35647
C	3.59669	19.80357	8.35439
H	4.14551	19.66000	9.27659
C	4.17322	19.42534	7.14497
C	1.34474	20.49784	4.75991
H	1.89528	20.14188	3.89054
C	1.12212	22.00294	4.59923
H	0.49993	22.40851	5.39895
H	0.61925	22.21807	3.65461
H	2.06573	22.54819	4.61246
C	0.00959	19.75431	4.76799
H	0.15657	18.67263	4.77460
H	-0.56178	19.98573	3.86798
H	-0.60992	20.02339	5.62534
C	5.56382	18.83314	7.14862
H	5.70656	18.32861	6.19175
C	6.60539	19.94768	7.27083
H	6.48594	20.69726	6.48951
H	7.61606	19.54141	7.19571
H	6.52362	20.45501	8.23441
C	5.76473	17.79033	8.24607
H	5.70081	18.21820	9.24788
H	6.75350	17.34012	8.15749
H	5.03424	16.98385	8.17111

B	2.93730	16.28098	5.59553
H	2.35625	15.33219	5.11867
H	3.92188	15.92709	6.19772
Fe	5.30180	19.68273	2.03156
N	4.43658	16.95628	2.92223
N	3.99041	19.16114	4.71780
N	1.93605	16.91430	6.72454
H	1.65348	16.19210	7.37817
H	1.09301	17.31545	6.32677
H	2.39290	17.65348	7.25541
Si	3.33631	17.60681	4.13248
H	2.07980	17.97184	3.37876

1H (C_{2v}): E_{tot} = -2047.57503431

C	0.64917	3.62491	2.77640
C	0.23219	3.12266	1.51085
H	0.86737	3.00596	0.64470
C	-1.13586	2.77365	1.60736
H	-1.73935	2.34937	0.81876
C	-1.57711	3.07691	2.92068
H	-2.57480	2.92385	3.30447
C	-0.48321	3.61409	3.63990
H	-0.48561	3.93451	4.67168
Fe	-0.00349	1.74381	2.99045
N	1.95137	4.03524	3.11916
Si	3.34280	3.22718	3.76850
C	1.64246	0.88085	3.73691
C	1.27699	0.23099	2.52368
H	1.88585	0.18275	1.63246
C	-0.02648	-0.29634	2.68437
H	-0.58995	-0.83382	1.93626
C	-0.46457	0.00374	3.99946
H	-1.41948	-0.26576	4.42564
C	0.56629	0.71768	4.65520
H	0.54169	1.10300	5.66425
N	2.84091	1.57930	3.97649
H	3.56687	0.98601	4.35433
H	2.10633	5.01911	2.94664

S2 (C_S): E_{tot} = -2048.79719499

C	0.70771	3.69191	2.66465
C	0.20052	3.11129	1.46922
H	0.75720	2.96246	0.55444
C	-1.15865	2.76739	1.68524
H	-1.82268	2.32386	0.95849
C	-1.48796	3.10895	3.01948
H	-2.44871	2.97258	3.49309
C	-0.34151	3.68513	3.62154
H	-0.26706	4.05159	4.63512
Fe	0.06564	1.76574	2.99815
N	2.02756	4.10575	2.90582
Si	2.96088	3.21757	4.08318
C	1.76888	0.82618	3.66860
C	1.27493	0.20149	2.48997
H	1.80992	0.10414	1.55554
C	-0.03535	-0.27302	2.75538
H	-0.66717	-0.80820	2.06240
C	-0.36501	0.07875	4.08686
H	-1.29392	-0.14047	4.59184
C	0.74793	0.74939	4.65284
H	0.81474	1.14370	5.65643
N	2.99545	1.49282	3.81813
H	3.73666	1.10501	3.25625
H	2.49574	4.45897	2.08629
H	4.35648	3.71319	4.02293
H	2.35266	3.45601	5.41010

S3 (C_S): E_{tot} = -2048.7412703

C	0.73912	3.70136	2.42750
C	0.29780	3.17517	1.18581
H	0.88725	3.08216	0.28418
C	-1.05555	2.78799	1.34367
H	-1.67942	2.35279	0.57776
C	-1.44630	3.08400	2.67342
H	-2.42087	2.90934	3.10333
C	-0.33592	3.64461	3.35033
H	-0.29613	3.95281	4.38508
Fe	0.14991	1.77278	2.67458
N	2.05556	4.17222	2.73248
Si	2.95717	3.19698	4.44982
C	1.85826	0.88804	3.42291
C	1.45281	0.27101	2.20582
H	2.04374	0.21224	1.30189
C	0.14599	-0.25807	2.38083
H	-0.42133	-0.80312	1.64123
C	-0.27003	0.04937	3.69686
H	-1.21584	-0.21732	4.14474
C	0.78148	0.75422	4.33973
H	0.76920	1.14166	5.34803
N	3.02393	1.61443	3.62762
H	4.32410	3.65360	3.94478
H	2.04282	5.16897	2.92493
H	3.73103	1.41916	2.93615
H	2.67058	4.03189	1.93841

S4 (C_1): E_{tot} = -3006.00793736

C	-1.48429	-0.02660	1.35371
C	-1.16537	1.07234	2.20005
H	-0.82368	2.04428	1.87377
C	-1.33208	0.65162	3.54120
H	-1.15054	1.25251	4.41964
C	-1.78688	-0.68986	3.52881
H	-2.00978	-1.29179	4.39690
C	-1.88740	-1.11110	2.18342
H	-2.19756	-2.08789	1.84511
C	-2.67305	-0.03002	-0.73387
C	-3.29826	1.19881	-0.97949
C	-4.54794	1.19100	-1.58793
H	-5.05235	2.12925	-1.78408
C	-5.15246	0.00621	-1.96368
H	-6.12409	0.01971	-2.43976
C	-4.50352	-1.19484	-1.74753
H	-4.97354	-2.11515	-2.07122
C	-3.25729	-1.23936	-1.13234
C	-2.65761	2.52912	-0.64906
H	-1.66604	2.34481	-0.23355
C	-2.45176	3.36601	-1.91199
H	-3.40000	3.64849	-2.37174
H	-1.87087	2.81915	-2.65531
H	-1.91626	4.28713	-1.67534
C	-3.45720	3.28876	0.40819
H	-2.95689	4.22242	0.67157
H	-3.57301	2.69875	1.31771
H	-4.45558	3.54406	0.04908
C	-2.56759	-2.57868	-0.97913
H	-1.67484	-2.44308	-0.36488
C	-2.10740	-3.09901	-2.34265
H	-1.46265	-2.38169	-2.85056
H	-2.95919	-3.29298	-2.99676
H	-1.55584	-4.03460	-2.23228
C	-3.44812	-3.60840	-0.27166
H	-2.88704	-4.52603	-0.08822
H	-4.31854	-3.88012	-0.87030
H	-3.81638	-3.23920	0.68638
Fe	0.03229	-0.53608	2.54980
N	-1.40113	-0.04704	-0.06030
Si	0.00499	0.01814	-1.04018
C	1.53598	-0.61553	1.23477
C	1.21738	-1.93376	1.66350
H	0.87225	-2.73739	1.02821
C	1.39929	-1.99044	3.06621
H	1.22405	-2.85212	3.69270
C	1.86354	-0.72459	3.50147
H	2.10076	-0.45201	4.51879
C	1.95359	0.12589	2.37533
H	2.27203	1.15753	2.37957
C	2.65667	0.30033	-0.69315
C	3.49456	-0.65345	-1.28375
C	4.68934	-0.21912	-1.84558
H	5.35730	-0.93828	-2.30290
C	5.03508	1.11938	-1.84492
H	5.96541	1.43917	-2.29569
C	4.18650	2.04995	-1.27507
H	4.46220	3.09700	-1.29220
C	2.98921	1.66250	-0.68398
C	3.15053	-2.12540	-1.32309
H	2.11299	-2.24275	-1.00723
C	3.24214	-2.69653	-2.73730
H	4.26438	-2.69326	-3.11853
H	2.62060	-2.12896	-3.42940
H	2.89906	-3.73228	-2.74723
C	4.02522	-2.91091	-0.34530
H	3.74464	-3.96572	-0.33812
H	3.92816	-2.53027	0.67163
H	5.07903	-2.85224	-0.62399
C	2.08667	2.72537	-0.08978
H	1.30320	2.23025	0.48846
C	1.40264	3.53854	-1.19048
H	0.85060	2.90240	-1.88299
H	2.13383	4.09425	-1.77998
H	0.70449	4.26042	-0.76171
C	2.83164	3.64561	0.87706
H	2.13282	4.32409	1.36873
H	3.57172	4.26299	0.36648
H	3.35595	3.08298	1.65051
N	1.42475	-0.12094	-0.08424
B	0.03727	0.13318	-2.99870
H	0.82139	1.00250	-3.29531
H	0.47935	-0.95688	-3.31030
H	-1.08754	0.31408	-3.38943

S5 (C_1): E_{tot} = -2980.63295189

C	1.41677	-0.70335	-1.17879
C	0.97108	-0.11509	-2.39614
H	0.60904	0.89620	-2.51254
C	1.03822	-1.09578	-3.41477
H	0.75200	-0.95754	-4.44675
C	1.55941	-2.28404	-2.84599
H	1.73685	-3.21253	-3.36789
C	1.79250	-2.04873	-1.47243
H	2.16920	-2.76939	-0.76359
C	2.71249	0.21893	0.66038
C	3.31742	1.44909	0.34525
C	4.56083	1.74342	0.89131
H	5.04730	2.67886	0.64440
C	5.18989	0.86438	1.75360
H	6.15600	1.11223	2.17367
C	4.57605	-0.32872	2.08041
H	5.07159	-1.00779	2.76376
C	3.33999	-0.67664	1.54357
C	2.67204	2.44784	-0.58968
H	1.62868	2.15730	-0.72489
C	2.66896	3.86309	-0.01417
H	3.67568	4.26802	0.09807
H	2.18584	3.89425	0.96259
H	2.12591	4.53916	-0.67589
C	3.34946	2.41506	-1.96050
H	2.84929	3.09160	-2.65649
H	3.32900	1.41421	-2.39102
H	4.39354	2.72620	-1.88982
C	2.74185	-2.01075	1.93778
H	1.79353	-2.13062	1.41100
C	2.44446	-2.07115	3.43659
H	1.78747	-1.25932	3.74628
H	3.35777	-2.00413	4.03014
H	1.95664	-3.01316	3.69321
C	3.65319	-3.17131	1.53244
H	3.15949	-4.12792	1.71232
H	4.58082	-3.17274	2.10695
H	3.92893	-3.12389	0.47894
Fe	-0.17824	-1.71859	-1.86988

N	1.43826	-0.09574	0.09517
Si	0.04729	0.26894	1.08992
C	-1.62219	-1.12984	-0.59014
C	-1.23903	-2.46637	-0.29328
H	-0.81423	-2.81013	0.64020
C	-1.47864	-3.25695	-1.44555
H	-1.28124	-4.31439	-1.53929
C	-2.04213	-2.42236	-2.44109
H	-2.34567	-2.73076	-3.43019
C	-2.12709	-1.10897	-1.91970
H	-2.50394	-0.23786	-2.43445
C	-2.58662	0.82027	0.45724
C	-3.60438	0.36226	1.31027
C	-4.73144	1.15570	1.48828
H	-5.53242	0.81164	2.13093
C	-4.84606	2.38608	0.86674
H	-5.72793	2.99376	1.02232
C	-3.82689	2.83735	0.05000
H	-3.92129	3.80447	-0.42933
C	-2.68994	2.06806	-0.17609
C	-3.51952	-0.97409	2.01561
H	-2.49338	-1.33570	1.93842
C	-3.83358	-0.86388	3.50710
H	-4.87246	-0.58791	3.69379
H	-3.20000	-0.12228	3.99418
H	-3.66420	-1.82314	3.99847
C	-4.42684	-2.00152	1.33721
H	-4.33188	-2.97735	1.81680
H	-4.17476	-2.12212	0.28386
H	-5.47480	-1.70135	1.39915
C	-1.61904	2.60747	-1.09807
H	-0.82699	1.86069	-1.16544
C	-1.00200	3.89312	-0.54946
H	-0.58146	3.74232	0.44440
H	-1.73873	4.69523	-0.48027
H	-0.19973	4.24260	-1.20168
C	-2.15197	2.83074	-2.51354
H	-1.34820	3.14800	-3.18049
H	-2.92077	3.60464	-2.53927
H	-2.58988	1.92292	-2.92832
N	-1.44432	-0.00224	0.23013
H	0.11066	-0.55443	2.32975
H	0.05758	1.69100	1.51150

S6 (C_1): E_{tot} = -2980.56273133

C	1.68604	3.89813	1.41079
C	1.37162	3.44727	0.09818
H	2.05435	3.42025	-0.73869
C	0.02359	3.00746	0.09471
H	-0.50696	2.60643	-0.75628
C	-0.50759	3.20222	1.39178
H	-1.51429	2.97381	1.70866
C	0.51640	3.74564	2.20628
H	0.43228	4.00517	3.25153
C	2.94071	5.48896	2.67021
C	3.07742	5.44656	4.06565
C	3.05720	6.63760	4.78335
H	3.16871	6.61473	5.86114
C	2.88500	7.85298	4.14891
H	2.86187	8.76993	4.72321
C	2.74394	7.88760	2.77379
H	2.60930	8.84281	2.28104
C	2.78070	6.72393	2.01438
C	3.25621	4.15083	4.81994
H	3.12928	3.34241	4.09706
C	4.65919	4.06229	5.41853
H	4.79545	3.13225	5.97234
H	4.84090	4.88208	6.11530
H	5.42239	4.11390	4.64160
C	2.19062	3.94944	5.89640
H	1.18420	4.01130	5.48165
H	2.26441	4.69487	6.68938
H	2.29942	2.96903	6.36547
C	2.61890	6.82464	0.51202
H	2.87434	5.86020	0.07120
C	3.56874	7.85017	-0.10560
H	4.60497	7.64455	0.16375
H	3.33974	8.87039	0.20640
H	3.49477	7.82301	-1.19381
C	1.16587	7.12901	0.14429
H	1.03987	7.15840	-0.93947
H	0.85240	8.09664	0.54164
H	0.48942	6.37031	0.53776
Fe	1.12144	1.94087	1.46132
N	2.93817	4.29537	1.89606
Si	4.39837	3.56311	1.13771
C	2.82890	0.97018	1.99683
C	2.28809	0.38598	0.82197
H	2.76140	0.32212	-0.14356
C	0.99342	-0.09209	1.13575
H	0.32563	-0.58819	0.44773
C	0.72434	0.20451	2.49346
H	-0.18184	-0.02905	3.03158
C	1.86093	0.85644	3.03083
H	1.96747	1.21364	4.04610
C	5.23493	0.63846	2.12320
C	5.84323	0.27882	0.91197
C	6.92018	-0.60359	0.95912
H	7.40799	-0.88445	0.03448
C	7.38350	-1.12752	2.14657
H	8.22226	-1.81081	2.15329
C	6.76010	-0.77687	3.32753
H	7.11376	-1.20515	4.25603
C	5.68338	0.10068	3.34439
C	5.42250	0.75700	-0.46425
H	4.56006	1.41743	-0.37273
C	6.51902	1.59501	-1.11882
H	7.43405	1.02096	-1.27499
H	6.76171	2.46546	-0.51058
H	6.18397	1.95943	-2.09089
C	5.03363	-0.41929	-1.36420
H	4.60937	-0.05222	-2.30017
H	4.29949	-1.07271	-0.89186
H	5.89448	-1.03770	-1.62302
C	5.01907	0.35444	4.68652
H	4.35998	1.22546	4.63032
C	6.02431	0.67320	5.79257
H	6.67708	1.50038	5.51552
H	6.65272	-0.18305	6.03815
H	5.49871	0.95077	6.70730
C	4.14470	-0.84090	5.07153
H	3.61754	-0.64890	6.00785
H	4.75381	-1.73549	5.21040
H	3.40325	-1.06059	4.30404
N	4.12268	1.57842	2.14762
H	4.12695	2.01444	3.06250
H	5.34209	3.95939	2.27067

S7 (C_1): E_{tot} = -3035.85089421

C	1.46563	-0.80430	1.10241
C	0.76295	-2.03565	1.06191
H	0.39139	-2.52472	0.17350
C	0.64592	-2.52509	2.38929
H	0.16736	-3.44773	2.68257
C	1.27806	-1.59777	3.24849
H	1.36334	-1.67856	4.32197
C	1.76534	-0.52522	2.45930
H	2.29627	0.34039	2.82417
C	3.19477	0.37777	-0.09794
C	4.12800	-0.50210	-0.67394
C	5.45133	-0.09512	-0.77711
H	6.18655	-0.75255	-1.22222
C	5.85487	1.14145	-0.30136
H	6.89238	1.43955	-0.37973
C	4.92986	1.99202	0.27011
H	5.25292	2.96108	0.63081
C	3.58768	1.63404	0.37562
C	3.69293	-1.84604	-1.22218
H	2.85307	-2.20222	-0.62188
C	3.21982	-1.70004	-2.67158
H	4.04103	-1.37810	-3.31398
H	2.42507	-0.95890	-2.77829
H	2.84242	-2.64819	-3.05964
C	4.76820	-2.92456	-1.12582
H	4.35048	-3.89220	-1.40640
H	5.15652	-3.01079	-0.11124
H	5.60878	-2.73581	-1.79541
C	2.60179	2.63279	0.93909
H	1.63062	2.14269	1.01932
C	2.43653	3.80686	-0.02522
H	2.12877	3.46193	-1.01180
H	3.36759	4.36643	-0.13859
H	1.66962	4.49438	0.33448
C	3.00121	3.13283	2.32679
H	2.22299	3.78054	2.73367
H	3.92486	3.71392	2.30468
H	3.15176	2.31337	3.03036
Fe	-0.27563	-0.70625	2.25199
N	1.82150	-0.00353	0.00781
Si	-0.92728	2.17900	-0.65503
C	-1.76403	0.42316	1.30457
C	-1.17182	1.15138	2.36996
H	-0.67723	2.10864	2.28969
C	-1.36376	0.41435	3.56773
H	-1.03463	0.71630	4.55101
C	-2.06495	-0.77005	3.24591
H	-2.36388	-1.54340	3.93786
C	-2.29729	-0.77646	1.84707
H	-2.81718	-1.54235	1.29037
C	-2.98785	0.28362	-0.74520
C	-4.27016	0.81348	-0.52414
C	-5.33571	0.30457	-1.25711
H	-6.33105	0.70159	-1.09730
C	-5.14801	-0.69390	-2.19649
H	-5.98805	-1.07126	-2.76490
C	-3.88181	-1.20793	-2.40358
H	-3.74256	-1.99197	-3.13821
C	-2.78712	-0.74235	-1.68020
C	-4.52780	1.92144	0.47419
H	-3.58001	2.20989	0.92841
C	-5.09235	3.16497	-0.21255
H	-6.06860	2.97697	-0.66286
H	-4.42349	3.51549	-0.99940
H	-5.21596	3.97550	0.50741
C	-5.43545	1.44277	1.60664
H	-5.57378	2.23381	2.34523
H	-5.00137	0.58295	2.11687
H	-6.42373	1.15640	1.24200
C	-1.42801	-1.35935	-1.92550
H	-0.74478	-0.94540	-1.18185
C	-0.88991	-1.00639	-3.31244
H	-0.80099	0.07190	-3.44038
H	-1.54191	-1.38554	-4.10161
H	0.09758	-1.44591	-3.46878
C	-1.45962	-2.87452	-1.72777
H	-0.46071	-3.29912	-1.84881
H	-2.10458	-3.36853	-2.45590
H	-1.82008	-3.13859	-0.73307
N	-1.88396	0.83294	-0.03498
N	-1.66736	2.38246	-2.19547
H	1.45414	-0.37043	-0.85683
H	-1.33374	3.10666	-2.80993
H	-2.44019	1.86923	-2.59460

S8 (C_1): E_{tot} = -3035.82011005

C	1.36807	-0.44270	1.50131
C	1.34701	-1.85605	1.65356
H	1.27715	-2.58330	0.85713
C	1.40917	-2.14234	3.03819

H	1.40617	-3.12758	3.47941
C	1.47809	-0.91316	3.73702
H	1.53431	-0.79290	4.80830
C	1.44906	0.14041	2.79290
H	1.47131	1.19297	3.02554
C	2.60107	0.69264	-0.25947
C	3.30969	-0.16119	-1.12295
C	4.55422	0.25062	-1.58685
H	5.11707	-0.39720	-2.24592
C	5.09506	1.46516	-1.22022
H	6.06320	1.76873	-1.59555
C	4.38654	2.28554	-0.36898
H	4.80785	3.24124	-0.08525
C	3.13977	1.92459	0.13194
C	2.85103	-1.54156	-1.56102
H	1.79884	-1.70069	-1.30366
C	2.93759	-1.72575	-3.07637
H	3.96898	-1.72500	-3.42889
H	2.40291	-0.93938	-3.60728
H	2.50546	-2.68502	-3.36492
C	3.65914	-2.62324	-0.84036
H	3.28492	-3.61806	-1.08924
H	3.62170	-2.50404	0.24177
H	4.70816	-2.58233	-1.13701
C	2.47423	2.91551	1.06110
H	1.54640	2.49014	1.43014
C	2.09765	4.19746	0.31812
H	1.43844	3.97571	-0.52158
H	2.98161	4.71708	-0.05670
H	1.57619	4.88132	0.99039
C	3.34518	3.20687	2.28454
H	2.80314	3.84073	2.98837
H	4.26175	3.73544	2.01940
H	3.63375	2.29192	2.80398
Fe	-0.24083	-0.99429	2.59664
N	1.29402	0.25630	0.24283
Si	-0.44373	1.50243	-0.06650
C	-1.80129	-0.36243	1.46902
C	-1.82462	0.29517	2.73280
H	-1.75142	1.36044	2.89335
C	-1.91954	-0.69112	3.74352
H	-1.94675	-0.50591	4.80715
C	-1.98816	-1.95850	3.11611
H	-2.07580	-2.91132	3.61659
C	-1.91317	-1.76053	1.71726
H	-1.94020	-2.53552	0.96549
C	-2.70447	0.05490	-0.71567
C	-3.81367	0.91169	-0.65349
C	-4.86445	0.70675	-1.54044
H	-5.72702	1.36132	-1.50582
C	-4.82539	-0.31145	-2.47464
H	-5.65238	-0.45564	-3.15768
C	-3.71855	-1.13686	-2.54047
H	-3.68788	-1.92017	-3.28797
C	-2.64737	-0.96982	-1.66916
C	-3.89287	2.07387	0.31208
H	-3.01160	2.05386	0.95302
C	-3.86021	3.40322	-0.44272
H	-4.71690	3.50944	-1.11084
H	-2.94909	3.48475	-1.03631
H	-3.88323	4.24070	0.25704
C	-5.10922	1.96624	1.22983
H	-5.11393	2.78333	1.95313
H	-5.10258	1.02710	1.78375
H	-6.04701	2.01980	0.67420
C	-1.42802	-1.84860	-1.82817
H	-0.80872	-1.72720	-0.93503
C	-0.61350	-1.38104	-3.03417
H	-0.26443	-0.35510	-2.90030
H	-1.21356	-1.40638	-3.94514
H	0.25482	-2.02057	-3.19568
C	-1.76686	-3.33350	-1.93901
H	-0.85462	-3.93274	-1.94105
H	-2.30280	-3.56649	-2.85996
H	-2.38740	-3.66838	-1.10690
N	-1.62786	0.23025	0.20590
N	-0.26668	2.32424	-1.42272
H	0.95002	-0.42390	-0.43081
H	-0.32652	2.23983	1.20624
H	-0.52404	2.03899	-2.35108

S12 (C_1): E_{tot} = -3006.00793736

C	-1.48429	-0.02660	1.35371
C	-1.16537	1.07234	2.20005
H	-0.82368	2.04428	1.87377
C	-1.33208	0.65162	3.54120
H	-1.15054	1.25251	4.41964
C	-1.78688	-0.68986	3.52881
H	-2.00978	-1.29179	4.39690
C	-1.88740	-1.11110	2.18342
H	-2.19756	-2.08789	1.84511
C	-2.67305	-0.03002	-0.73387
C	-3.29826	1.19881	-0.97949
C	-4.54794	1.19100	-1.58793
H	-5.05235	2.12925	-1.78408
C	-5.15246	0.00621	-1.96368
H	-6.12409	0.01971	-2.43976
C	-4.50352	-1.19484	-1.74753
H	-4.97354	-2.11515	-2.07122
C	-3.25729	-1.23936	-1.13234
C	-2.65761	2.52912	-0.64906
H	-1.66604	2.34481	-0.23355
C	-2.45176	3.36601	-1.91199
H	-3.40000	3.64849	-2.37174
H	-1.87087	2.81915	-2.65531
H	-1.91626	4.28713	-1.67534
C	-3.45720	3.28876	0.40819
H	-2.95689	4.22242	0.67157
H	-3.57301	2.69875	1.31771
H	-4.45558	3.54406	0.04908
C	-2.56759	-2.57868	-0.97913
H	-1.67484	-2.44308	-0.36488
C	-2.10740	-3.09901	-2.34265
H	-1.46265	-2.38169	-2.85056
H	-2.95919	-3.29298	-2.99676
H	-1.55584	-4.03460	-2.23228
C	-3.44812	-3.60840	-0.27166
H	-2.88704	-4.52603	-0.08822
H	-4.31854	-3.88012	-0.87030
H	-3.81638	-3.23920	0.68638
Fe	0.03229	-0.53608	2.54980
N	-1.40113	-0.04704	-0.06030
Si	0.00499	0.01814	-1.04018
C	1.53598	-0.61553	1.23477
C	1.21738	-1.93376	1.66350
H	0.87225	-2.73739	1.02821
C	1.39929	-1.99044	3.06621
H	1.22405	-2.85212	3.69270
C	1.86354	-0.72459	3.50147
H	2.10076	-0.45201	4.51879
C	1.95359	0.12589	2.37533
H	2.27203	1.15753	2.37957
C	2.65667	0.30033	-0.69315
C	3.49456	-0.65345	-1.28375
C	4.68934	-0.21912	-1.84558
H	5.35730	-0.93828	-2.30290
C	5.03508	1.11938	-1.84492
H	5.96541	1.43917	-2.29569
C	4.18650	2.04995	-1.27507
H	4.46220	3.09700	-1.29220
C	2.98921	1.66250	-0.68398
C	3.15053	-2.12540	-1.32309
H	2.11299	-2.24275	-1.00723
C	3.24214	-2.69653	-2.73730
H	4.26438	-2.69326	-3.11853
H	2.62060	-2.12896	-3.42940
H	2.89906	-3.73228	-2.74723
C	4.02522	-2.91091	-0.34530
H	3.74464	-3.96572	-0.33812
H	3.92816	-2.53027	0.67163
H	5.07903	-2.85224	-0.62399
C	2.08667	2.72537	-0.08978
H	1.30320	2.23025	0.48846
C	1.40264	3.53854	-1.19048
H	0.85060	2.90240	-1.88299
H	2.13383	4.09425	-1.77998
H	0.70449	4.26042	-0.76171
C	2.83164	3.64561	0.87706
H	2.13282	4.32409	1.36873
H	3.57172	4.26299	0.36648
H	3.35595	3.08298	1.65051
N	1.42475	-0.12094	-0.08424
B	0.03727	0.13318	-2.99870
H	0.82139	1.00250	-3.29531
H	0.47935	-0.95688	-3.31030
H	-1.08754	0.31408	-3.38943

S14 (C_1): E_{tot} = -3005.98879087

C	1.39555	-0.77867	-1.12591
C	1.01354	-0.22387	-2.38072
H	0.70173	0.79676	-2.54913
C	1.07421	-1.24557	-3.35834
H	0.82966	-1.13732	-4.40454
C	1.52310	-2.43120	-2.72535
H	1.67607	-3.38705	-3.20389
C	1.72034	-2.15004	-1.35490
H	2.04261	-2.85794	-0.60743
C	2.70386	0.18004	0.67588
C	3.36731	1.36590	0.30689
C	4.61173	1.64107	0.85923
H	5.13771	2.54323	0.57344
C	5.19402	0.78659	1.77736
H	6.16072	1.02132	2.20353
C	4.53350	-0.36767	2.14498
H	4.99519	-1.03375	2.86388
C	3.29435	-0.69820	1.60211
C	2.78751	2.33211	-0.70142
H	1.72639	2.09916	-0.81268
C	2.89027	3.78804	-0.24973
H	3.92432	4.13017	-0.19043
H	2.43246	3.94052	0.72751
H	2.38442	4.44163	-0.96208
C	3.46166	2.14870	-2.06298
H	2.99974	2.79121	-2.81561
H	3.39235	1.11914	-2.41155
H	4.52024	2.41074	-2.00997
C	2.66761	-2.00859	2.03251
H	1.72259	-2.13195	1.50211
C	2.35589	-2.01994	3.52930
H	1.70936	-1.18906	3.80945
H	3.26448	-1.95009	4.12969
H	1.84927	-2.94522	3.80910
C	3.56286	-3.19597	1.66980
H	3.05038	-4.13810	1.87270
H	4.48562	-3.19762	2.25200
H	3.84732	-3.18337	0.61760
Fe	-0.22035	-1.75414	-1.83840
N	1.41921	-0.10575	0.11620
Si	0.01687	0.19312	1.13957
C	-1.67258	-1.09544	-0.60345
C	-1.36805	-2.45094	-0.29999
H	-0.99030	-2.81815	0.64434
C	-1.61106	-3.22437	-1.46304
H	-1.46522	-4.29028	-1.55560
C	-2.09702	-2.35898	-2.47324
H	-2.38236	-2.64869	-3.47338
C	-2.13144	-1.04437	-1.94882
H	-2.44591	-0.15300	-2.47166

C	-2.57707	0.88934	0.41185
C	-3.63718	0.48689	1.23995
C	-4.72934	1.33424	1.38248
H	-5.56121	1.03459	2.00804
C	-4.77012	2.56379	0.74946
H	-5.62529	3.21415	0.87927
C	-3.71127	2.95877	-0.04564
H	-3.74726	3.92521	-0.53436
C	-2.60884	2.13189	-0.23637
C	-3.62825	-0.84282	1.96190
H	-2.61946	-1.25337	1.90476
C	-3.95895	-0.69669	3.44665
H	-4.98615	-0.36878	3.61330
H	-3.29798	0.02148	3.93269
H	-3.84245	-1.65551	3.95401
C	-4.57266	-1.83446	1.28128
H	-4.53231	-2.80732	1.77440
H	-4.30838	-1.98049	0.23405
H	-5.60616	-1.48448	1.32115
C	-1.47730	2.60843	-1.11785
H	-0.76902	1.78605	-1.22725
C	-0.74017	3.77898	-0.47220
H	-0.32351	3.51547	0.50634
H	-1.40037	4.63206	-0.30858
H	0.09306	4.11213	-1.09165
C	-1.95370	2.98319	-2.52067
H	-1.10482	3.23682	-3.15807
H	-2.62115	3.84615	-2.51095
H	-2.49054	2.15998	-2.99239
N	-1.46500	0.01974	0.22601
B	0.05836	1.96243	2.09173
H	-0.91562	2.36154	2.65202
H	1.06321	2.58868	2.23001
H	0.01059	-0.82341	2.24567

S11 (C_1): E_{tot} = -3005.97389598

C	1.57611	3.18295	1.83317
C	1.40698	2.44321	0.62573
H	2.12140	2.37266	-0.18143
C	0.15308	1.78929	0.67893
H	-0.25424	1.13744	-0.07951
C	-0.47714	2.14849	1.89571
H	-1.44721	1.81239	2.23048
C	0.39080	3.01083	2.60428
H	0.20338	3.44081	3.57644
C	2.50532	5.35741	2.23172
C	2.23226	6.00015	3.45082
C	2.03291	7.37712	3.45036
H	1.82575	7.88737	4.38321
C	2.09106	8.10829	2.27990
H	1.92525	9.17759	2.29746
C	2.37016	7.46822	1.08657
H	2.42153	8.05038	0.17463
C	2.59286	6.09721	1.03944
C	2.15651	5.26201	4.77067
H	2.25338	4.19309	4.57236
C	3.30845	5.66804	5.69153
H	3.29971	5.07242	6.60565
H	3.23633	6.71864	5.97873
H	4.27706	5.52585	5.21227
C	0.81441	5.48192	5.47070
H	-0.02793	5.24305	4.82082
H	0.68888	6.51691	5.79229
H	0.74252	4.85622	6.36180
C	2.90758	5.45835	-0.29490
H	3.20894	4.42549	-0.11504
C	4.08137	6.14371	-0.99288
H	4.96873	6.14699	-0.35985
H	3.85669	7.17698	-1.26095
H	4.33044	5.62110	-1.91823
C	1.66869	5.43275	-1.19059
H	1.88126	4.92086	-2.13126
H	1.33631	6.44366	-1.43414
H	0.83957	4.91705	-0.70663
Fe	1.34289	1.24703	2.26971
N	2.70551	3.93621	2.20615
Si	4.41171	3.50545	2.44320
C	3.15726	0.77607	3.02047
C	2.77433	-0.19412	2.05295
H	3.31999	-0.44966	1.15853
C	1.56793	-0.79306	2.48545
H	1.02658	-1.56597	1.96106
C	1.20164	-0.20082	3.71656
H	0.32646	-0.43646	4.30339
C	2.18426	0.76179	4.05618
H	2.17758	1.38567	4.93517
C	5.44503	0.75870	2.36321
C	5.81472	0.92173	1.01790
C	6.89982	0.18933	0.53829
H	7.21523	0.32654	-0.48830
C	7.57234	-0.71598	1.32916
H	8.41463	-1.26875	0.93475
C	7.13286	-0.93406	2.61994
H	7.62615	-1.68238	3.22724
C	6.06600	-0.22420	3.15539
C	5.10015	1.78418	-0.00710
H	4.23398	2.26413	0.45656
C	6.00469	2.88118	-0.56588
H	6.83704	2.45782	-1.12972
H	6.41431	3.50279	0.22834
H	5.44662	3.52771	-1.24470
C	4.52630	0.94103	-1.14964
H	3.97257	1.57778	-1.84229
H	3.84368	0.17197	-0.79007
H	5.30981	0.44475	-1.72291
C	5.60114	-0.62688	4.54211
H	4.80561	0.03424	4.87253
C	6.71625	-0.51944	5.58038
H	7.13016	0.48772	5.60870
H	7.53402	-1.21470	5.38443
H	6.32739	-0.74960	6.57327
C	5.00275	-2.03477	4.50531
H	4.62508	-2.30979	5.49140
H	5.74024	-2.78445	4.21304
H	4.17034	-2.08986	3.80329
N	4.37120	1.55866	2.96277
B	4.77158	2.26942	4.34919
H	5.96019	2.34906	4.46569
H	4.37120	3.47229	4.20235
H	4.16787	1.91152	5.31564

S9 (C_1): E_{tot} = -3062.49810415

C	1.69363	-1.20671	-0.46659
C	1.43696	-2.48816	0.09730
H	1.08317	-2.68177	1.10036
C	1.70877	-3.46372	-0.89310
H	1.60183	-4.53148	-0.77393
C	2.16834	-2.79580	-2.05303
H	2.46891	-3.26408	-2.97820
C	2.15430	-1.40395	-1.79663
H	2.43893	-0.62674	-2.48819
C	-1.39546	-1.09925	-1.08333
C	-1.67096	-2.49884	-1.02109
H	-1.98955	-3.04567	-0.14742
C	-1.43406	-3.05212	-2.29863
H	-1.54520	-4.09183	-2.56802
C	-1.01105	-2.00984	-3.16028
H	-0.74675	-2.11501	-4.20193
C	-1.00261	-0.80450	-2.41929
H	-0.71866	0.16882	-2.79219
C	2.49447	0.98287	0.22215
C	3.48979	0.81367	1.20090
C	4.52333	1.73986	1.26041
H	5.30045	1.62546	2.00487
C	4.57453	2.81697	0.39410
H	5.38294	3.53275	0.46503
C	3.59112	2.97409	-0.56199
H	3.64368	3.81610	-1.24165
C	2.54454	2.06261	-0.67455
C	3.49729	-0.36633	2.14811
H	2.49423	-0.79482	2.16926
C	4.46085	-1.44131	1.63864
H	4.21267	-1.76453	0.62793
H	4.43377	-2.31929	2.28632
H	5.48709	-1.06862	1.62785
C	3.83260	0.02551	3.58566
H	4.86438	0.36350	3.69489
H	3.71102	-0.83764	4.24144
H	3.17171	0.81339	3.94459
C	1.53149	2.26402	-1.78199
H	0.78970	1.46632	-1.70986
C	0.80212	3.60163	-1.66134
H	0.27887	3.70840	-0.71088
H	0.05922	3.70541	-2.45354
H	1.48874	4.44482	-1.74671
C	2.18929	2.16749	-3.16027
H	2.88072	2.99403	-3.33102
H	1.43649	2.20282	-3.94974
H	2.75699	1.24578	-3.27987
C	-2.75305	0.23681	0.43350
C	-3.41087	-0.44418	1.47331
C	-4.65836	0.01464	1.88607
H	-5.17115	-0.49420	2.69285
C	-5.26048	1.10210	1.28663
H	-6.23405	1.43768	1.61879
C	-4.61035	1.76150	0.26065
H	-5.09147	2.61126	-0.20672
C	-3.35411	1.35574	-0.17444
C	-2.84792	-1.66564	2.16985
H	-1.88851	-1.91474	1.71615
C	-3.76763	-2.87530	1.98864
H	-4.71937	-2.73905	2.50417
H	-3.30275	-3.77228	2.40145
H	-3.99247	-3.06100	0.93787
C	-2.59175	-1.39833	3.65335
H	-1.93255	-0.54319	3.80263
H	-2.12733	-2.26764	4.12195
H	-3.52044	-1.19633	4.18983
C	-2.70135	2.11388	-1.31021
H	-1.65081	1.81806	-1.35586
C	-3.35219	1.74174	-2.64379
H	-3.32578	0.66647	-2.81614
H	-2.84102	2.23045	-3.47578
H	-4.39730	2.05578	-2.66772
C	-2.74310	3.62761	-1.10055
H	-3.76044	4.01935	-1.12089
H	-2.19155	4.13682	-1.89115
H	-2.30167	3.92279	-0.14707
H	0.46540	2.48753	1.08051
H	-1.13485	2.28055	1.34086
Fe	0.26872	-2.14305	-1.54967
N	1.42498	0.03904	0.13721
N	-1.45489	-0.17941	-0.00826
H	0.07550	3.21243	3.42443
H	1.10712	1.48073	3.39433
H	-0.87364	1.45612	3.72017
Si	-0.07658	0.17606	0.99934
H	-0.07721	-0.64948	2.22049
B	0.04698	2.02871	3.18102
N	-0.21698	1.89233	1.54407

S10 (C_1): E_{tot} = -3062.40913285

C	1.50360	3.76727	1.66527
C	1.12983	3.49085	0.31787
H	1.77866	3.59077	-0.53994
C	-0.19683	2.99443	0.31570
H	-0.75495	2.68677	-0.55646
C	-0.66476	2.98000	1.65154
H	-1.64255	2.66146	1.98142

C	0.38036	3.45108	2.48395
H	0.33816	3.53746	3.55823
C	2.88686	5.33075	2.86006
C	2.99688	5.32958	4.26574
C	3.23380	6.53113	4.93020
H	3.33059	6.53328	6.00962
C	3.32307	7.72994	4.24539
H	3.50281	8.65336	4.78062
C	3.15370	7.73791	2.87195
H	3.19204	8.68193	2.34188
C	2.93813	6.55975	2.16339
C	2.79655	4.08303	5.09780
H	2.67488	3.24625	4.40570
C	3.97843	3.78186	6.01584
H	3.80067	2.86192	6.57630
H	4.13168	4.57519	6.74996
H	4.90101	3.65961	5.45106
C	1.51918	4.20220	5.93500
H	0.65733	4.49218	5.33520
H	1.63017	4.95564	6.71701
H	1.28863	3.25457	6.42530
C	2.71847	6.64151	0.66611
H	2.80470	5.63895	0.24651
C	3.76311	7.49541	-0.04920
H	4.77530	7.12717	0.13198
H	3.73061	8.54485	0.24781
H	3.60072	7.46061	-1.12700
C	1.30399	7.14495	0.37466
H	1.11447	7.16348	-0.69997
H	1.15484	8.15644	0.75856
H	0.55662	6.49769	0.83292
Fe	1.01906	1.80322	1.46748
N	2.77919	4.12124	2.13305
Si	4.23942	3.50739	1.17547
C	2.78805	0.86872	1.83316
C	2.27136	0.45518	0.57828
H	2.73349	0.59971	-0.38433
C	1.01371	-0.15146	0.80966
H	0.36870	-0.56703	0.05019
C	0.74347	-0.10359	2.19892
H	-0.13996	-0.48144	2.69106
C	1.84361	0.52086	2.83565
H	1.94130	0.71150	3.89538
C	5.17948	0.62605	2.08603
C	5.81501	0.27520	0.88700
C	6.87077	-0.62975	0.94450
H	7.37260	-0.90838	0.02654
C	7.30059	-1.17308	2.13681
H	8.12206	-1.87705	2.15429
C	6.67508	-0.80127	3.30926
H	7.01587	-1.22539	4.24517
C	5.61501	0.09791	3.31328
C	5.46375	0.82771	-0.47885
H	4.60982	1.50158	-0.39085
C	6.60260	1.69089	-1.02054
H	7.51354	1.11132	-1.18297
H	6.83500	2.50184	-0.33096
H	6.31442	2.13724	-1.97351
C	5.10104	-0.28433	-1.46409
H	4.74334	0.14522	-2.40147
H	4.32114	-0.93725	-1.07087
H	5.95922	-0.91360	-1.70667
C	4.99761	0.41680	4.66308
H	4.27047	1.22819	4.57188
C	6.04674	0.91503	5.65633
H	6.57920	1.77848	5.25866
H	6.78190	0.14584	5.89437
H	5.57309	1.20898	6.59377
C	4.24420	-0.79415	5.21548
H	3.74388	-0.54321	6.15277
H	4.92579	-1.62162	5.41806
H	3.49255	-1.15379	4.51308
N	4.04011	1.53452	2.07765
H	3.97899	1.94995	3.00182
B	5.81586	3.90170	2.46464
H	6.84300	3.48754	1.96757
H	5.68764	3.43363	3.58196
N	6.05312	5.50396	2.70128
H	6.93914	5.67548	3.16353
H	5.31671	5.90130	3.27981
H	6.05650	6.02396	1.82963

S13 (C_1): E_{tot} = -3062.47254885

C	-1.76620	1.21874	-0.43136
C	-1.53889	2.47089	0.20695
H	-1.19119	2.60811	1.22317
C	-1.82109	3.49784	-0.72722
H	-1.73073	4.55851	-0.54652
C	-2.26377	2.89076	-1.92669
H	-2.56808	3.40669	-2.82493
C	-2.22769	1.48650	-1.74986
H	-2.49722	0.74796	-2.48745
C	1.30928	1.24165	-0.90053
C	1.54610	2.65048	-0.86647
H	1.80439	3.22710	0.00667
C	1.34161	3.16926	-2.16436
H	1.43608	4.20521	-2.45390
C	0.97029	2.09788	-3.01351
H	0.73691	2.17149	-4.06527
C	0.96606	0.90996	-2.24319
H	0.70857	-0.07442	-2.60645
C	-2.51594	-1.00719	0.14418
C	-3.58444	-0.84553	1.04880
C	-4.59502	-1.80078	1.06346
H	-5.42397	-1.68608	1.74989
C	-4.56442	-2.89958	0.22510
H	-5.35928	-3.63321	0.25924
C	-3.51736	-3.04937	-0.66298
H	-3.50984	-3.90222	-1.33103
C	-2.48908	-2.11333	-0.72842
C	-3.70378	0.35742	1.95956
H	-2.72341	0.82264	2.05001
C	-4.67018	1.37797	1.35280
H	-4.36466	1.68398	0.35273
H	-4.71751	2.27377	1.97413
H	-5.67981	0.96712	1.28390
C	-4.12992	-0.00910	3.37939
H	-5.15401	-0.38388	3.42771
H	-4.08187	0.87547	4.01498
H	-3.46514	-0.75554	3.81143
C	-1.42171	-2.30194	-1.78810
H	-0.63817	-1.56175	-1.61074
C	-0.79875	-3.70066	-1.76882
H	-0.45073	-4.01036	-0.78163
H	0.05083	-3.75000	-2.45112
H	-1.51018	-4.46144	-2.09209
C	-1.98601	-2.04371	-3.18717
H	-2.75630	-2.77430	-3.43950
H	-1.20058	-2.11532	-3.94198
H	-2.43708	-1.05774	-3.26939
C	2.65043	-0.01684	0.69517
C	3.26531	0.67645	1.75586
C	4.48490	0.21040	2.23991
H	4.96169	0.73131	3.06054
C	5.10980	-0.89152	1.69261
H	6.05880	-1.23220	2.08565
C	4.52198	-1.54491	0.62648
H	5.03130	-2.39050	0.18151
C	3.29792	-1.12829	0.11573
C	2.72678	1.96203	2.34719
H	1.71681	2.12311	1.96733
C	3.61514	3.13249	1.90765
H	4.59564	3.07526	2.38408
H	3.16823	4.08536	2.19827
H	3.78380	3.14696	0.83119
C	2.63200	1.93676	3.87186
H	2.01409	1.11258	4.21715
H	2.17730	2.86216	4.22836
H	3.61512	1.86130	4.34043
C	2.75345	-1.84492	-1.10279
H	1.70385	-1.56645	-1.22357
C	3.48903	-1.37639	-2.36043
H	3.43120	-0.29506	-2.47402
H	3.06205	-1.83344	-3.25563
H	4.54421	-1.65194	-2.31675
C	2.82807	-3.36870	-0.99031
H	3.85666	-3.72974	-0.98924
H	2.33059	-3.83879	-1.83929
H	2.36131	-3.74807	-0.07851
H	0.94199	-2.27625	1.83112
H	-0.64630	-2.52168	1.51159
Fe	-0.35767	2.23438	-1.44231
N	-1.45971	-0.04922	0.09815
N	1.35719	0.36222	0.20820
Si	-0.04384	-0.13997	1.16582
N	0.20346	-2.08866	1.15614
H	0.75871	-0.48110	3.60938
H	-0.44934	1.13535	3.52091
H	-1.19254	-0.71878	3.31379
H	0.44857	-2.52362	0.27256
B	-0.23612	0.01692	3.11341

NH_3 (C_{3v}): E_{tot} = -56.42815156

N	0.01434	0.02491	1.03017
H	-0.94389	-0.01349	1.34546
H	0.46024	-0.82416	1.34538
H	-0.00775	-0.01354	0.02163

H_2O (C_{2v}): E_{tot} = -76.25805141

O	0.00000	0.05858	0.05858
H	0.00000	-0.06632	1.00773
H	0.00000	1.00773	-0.06632

BH_3NH_3 (C_{3v}): E_{tot} = -83.0248408

N	0.67105	-0.21527	0.65169
H	0.66987	-0.74876	1.51318
H	0.02841	0.56045	0.76152
H	0.31962	-0.81205	-0.08799
B	2.19787	0.32879	0.29688
H	2.51810	1.02286	1.23338
H	2.08799	0.94583	-0.73678
H	2.87687	-0.66537	0.18759

BH_2NH_2 (C_{2v}): E_{tot} = -81.84862721

N	3.36822	0.35251	7.51437
B	2.12712	0.07720	8.07042
H	3.58645	1.22047	7.05820
H	4.13443	-0.29692	7.52913
H	1.26416	0.90113	8.01327
H	1.94259	-0.97900	8.59690

H_2 ($D_{\infty h}$): E_{tot} = -1.16803293

H	0.00000	0.00000	0.00000
H	0.00000	0.00000	0.74000

TS1 (C_1): E_{tot} = -3092.27494143

C	-0.75816	1.16408	1.24339
C	0.44696	1.59319	1.87408
H	1.37062	1.83455	1.36812
C	0.25216	1.58740	3.27642
H	0.99072	1.84695	4.02038
C	-1.08363	1.19077	3.53047
H	-1.54257	1.09198	4.50303
C	-1.70658	0.92909	2.28820
H	-2.71802	0.57755	2.15933
C	-1.70662	1.92780	-0.84636
C	-1.05409	3.04494	-1.41416
C	-1.77662	3.92013	-2.22127
H	-1.28332	4.78230	-2.65236
C	-3.12219	3.71945	-2.47856
H	-3.66732	4.40942	-3.11014
C	-3.76790	2.64626	-1.89419
H	-4.82778	2.50891	-2.07212

C	-3.08815	1.75072	-1.07166
C	0.40113	3.35465	-1.14255
H	0.87406	2.44679	-0.76769
C	1.16834	3.76472	-2.39718
H	0.80527	4.69964	-2.82686
H	1.11630	2.99107	-3.16433
H	2.22168	3.91451	-2.16048
C	0.50861	4.43564	-0.06483
H	1.55348	4.62305	0.19090
H	-0.01168	4.14238	0.84616
H	0.07708	5.37799	-0.40872
C	-3.89540	0.65571	-0.40995
H	-3.19611	-0.00511	0.10361
C	-4.69428	-0.18663	-1.40458
H	-4.04773	-0.62898	-2.16096
H	-5.46322	0.39851	-1.91197
H	-5.20613	-0.99970	-0.88630
C	-4.83724	1.26226	0.63423
H	-5.34106	0.47873	1.20397
H	-5.61029	1.87072	0.16097
H	-4.30489	1.90408	1.33545
Fe	-0.08570	-0.28062	2.46938
N	-0.96150	0.94616	-0.13407
Si	-0.07429	-0.19174	-1.25751
C	0.64882	-1.59103	1.12346
C	-0.59416	-2.06349	1.63145
H	-1.48783	-2.24002	1.05040
C	-0.47203	-2.21875	3.03614
H	-1.25013	-2.55219	3.70681
C	0.85293	-1.87656	3.40062
H	1.26261	-1.89957	4.39936
C	1.54363	-1.48719	2.22785
H	2.57091	-1.15886	2.17052
C	2.09703	-1.83660	-0.75098
C	2.04250	-3.19635	-1.10835
C	3.18068	-3.80756	-1.62072
H	3.15154	-4.85803	-1.88522
C	4.35267	-3.09726	-1.80719
H	5.22886	-3.58617	-2.21317
C	4.39519	-1.75822	-1.46904
H	5.31586	-1.20526	-1.61510
C	3.28660	-1.11205	-0.93075
C	0.78569	-4.02117	-0.93285
H	-0.03143	-3.34295	-0.69040
C	0.39095	-4.74896	-2.21799
H	1.11756	-5.51088	-2.50398
H	0.30030	-4.05624	-3.05663
H	-0.56889	-5.25315	-2.09084
C	0.93284	-5.00042	0.23157
H	0.00957	-5.56222	0.38636
H	1.16327	-4.47614	1.15865
H	1.73230	-5.72027	0.04442
C	3.41220	0.34640	-0.55428
H	2.46848	0.65180	-0.10108
C	3.63606	1.21425	-1.79022
H	2.80918	1.09961	-2.49082
H	4.56119	0.94968	-2.30607
H	3.70524	2.26854	-1.51515
C	4.51391	0.57673	0.47998
H	4.53054	1.62182	0.79513
H	5.50287	0.34166	0.08282
H	4.36598	-0.03614	1.36944
N	0.94677	-1.21153	-0.19340
N	-1.49974	-1.20703	-1.82313
H	-1.84320	0.06166	-3.37305
H	-2.14379	-1.54067	-1.11520
H	-1.18569	-2.02258	-2.33970
N	-1.24736	0.77800	-3.82846
H	-1.65559	1.70488	-3.72166
H	-1.05569	0.56288	-4.80014
H	-0.34340	0.66331	-3.14209

TS2 (C_1): E_{tot} = -3055.6544025

C	1.55827	-0.94340	0.86179
C	1.32871	-2.34647	0.78580
H	1.03593	-2.89369	-0.09900
C	1.50959	-2.88766	2.08209
H	1.38978	-3.92539	2.35534
C	1.87712	-1.83283	2.95263
H	2.08799	-1.92320	4.00761
C	1.91450	-0.63334	2.20213
H	2.16964	0.34474	2.57944
C	2.61705	0.85027	-0.29241
C	3.82094	0.35256	-0.82263
C	4.92156	1.19796	-0.89063
H	5.85565	0.82608	-1.29319
C	4.85096	2.50557	-0.44941
H	5.71616	3.15197	-0.51571
C	3.67003	2.97561	0.08830
H	3.62383	3.99697	0.44581
C	2.54276	2.16524	0.19229
C	4.00105	-1.08257	-1.27866
H	3.03752	-1.59038	-1.28957
C	4.55575	-1.17356	-2.70004
H	5.57059	-0.78105	-2.77427
H	3.93804	-0.61794	-3.40668
H	4.58861	-2.21375	-3.02734
C	4.87675	-1.85019	-0.28732
H	4.97654	-2.89380	-0.59028
H	4.44392	-1.83215	0.71287
H	5.87950	-1.42328	-0.22837
C	1.31228	2.76254	0.84370
H	0.53807	1.99397	0.91111
C	0.75638	3.92164	0.01827
H	0.48689	3.59649	-0.98573
H	1.48323	4.73090	-0.06888
H	-0.13636	4.33444	0.48915
C	1.59180	3.22078	2.27672
H	0.67025	3.56701	2.74792
H	2.30181	4.04833	2.30711
H	1.99882	2.41977	2.89316
Fe	0.04487	-1.44431	2.07991
N	1.48107	-0.03337	-0.23229
Si	-0.16419	0.59818	-1.15142
C	-1.51803	-0.60787	1.13940
C	-1.28246	0.06743	2.37315
H	-1.02567	1.11134	2.48301
C	-1.40152	-0.87182	3.42528
H	-1.26096	-0.66799	4.47643
C	-1.74142	-2.12457	2.85709
H	-1.90103	-3.04470	3.39942
C	-1.81878	-1.96540	1.45439
H	-2.03701	-2.74517	0.74116
C	-2.79472	0.22057	-0.69614
C	-3.47251	1.41297	-0.38609
C	-4.75296	1.60924	-0.89151
H	-5.28874	2.51871	-0.64830
C	-5.35778	0.66897	-1.70305
H	-6.35477	0.84054	-2.08762
C	-4.67556	-0.48739	-2.02578
H	-5.14770	-1.21613	-2.67364
C	-3.39765	-0.73430	-1.53416
C	-2.87650	2.50016	0.48016
H	-1.83503	2.24513	0.68018
C	-2.87362	3.84987	-0.23676
H	-3.88279	4.19836	-0.46069
H	-2.32149	3.79507	-1.17484
H	-2.40340	4.61259	0.38622
C	-3.60530	2.59329	1.82117
H	-3.12669	3.32862	2.47138
H	-3.60990	1.63513	2.34024
H	-4.64373	2.90205	1.68694
C	-2.71401	-2.01871	-1.95083
H	-1.75976	-2.08639	-1.43150
C	-2.41298	-2.00830	-3.45024
H	-1.85276	-1.11874	-3.73734
H	-3.32994	-2.01848	-4.04244
H	-1.82696	-2.88452	-3.73264
C	-3.54045	-3.25171	-1.58205
H	-2.97909	-4.16418	-1.79129
H	-4.46853	-3.30667	-2.15336
H	-3.81211	-3.25596	-0.52616
N	-1.48814	-0.02993	-0.14855
O	0.46619	-1.01443	-2.09934
H	1.29852	-0.75764	-1.33345
H	0.69684	-0.82624	-3.01491

TS3 (C_1): E_{tot} = -3208.20655532

C	1.43046	-0.35931	1.40143
C	1.54811	-1.73649	1.73056
H	1.63390	-2.55647	1.03254
C	1.50580	-1.84166	3.14022
H	1.56450	-2.75945	3.70536
C	1.37400	-0.53763	3.67622
H	1.31001	-0.28428	4.72355
C	1.32399	0.38559	2.60553
H	1.19749	1.45292	2.69614
C	2.71481	0.53159	-0.46495
C	3.37947	-0.39355	-1.28791
C	4.66226	-0.08137	-1.72303
H	5.19543	-0.78269	-2.35135
C	5.27677	1.10127	-1.36829
H	6.27676	1.32328	-1.71602
C	4.59903	1.99882	-0.57232
H	5.07609	2.93235	-0.30501
C	3.31297	1.74535	-0.10403
C	2.83267	-1.73929	-1.73537
H	1.77922	-1.85515	-1.45554
C	2.86964	-1.87958	-3.25744
H	3.89107	-1.88405	-3.63777
H	2.33392	-1.06681	-3.74471
H	2.41096	-2.82109	-3.56169
C	3.59914	-2.88447	-1.06951
H	3.14665	-3.84662	-1.31495
H	3.62549	-2.78301	0.01497
H	4.63355	-2.91167	-1.41417
C	2.68973	2.81987	0.76013
H	1.66086	2.55592	0.99500
C	2.61982	4.16065	0.02954
H	2.12511	4.07046	-0.93515
H	3.61318	4.58350	-0.13179
H	2.03963	4.86729	0.62061
C	3.44842	2.96240	2.08142
H	2.93350	3.66983	2.73308
H	4.45799	3.34445	1.92060
H	3.53913	2.01308	2.60998
Fe	-0.20441	-0.94379	2.41242
N	1.38706	0.16429	0.05359
Si	-0.24655	1.32805	-0.28816
C	-1.70357	-0.49886	1.13486
C	-1.89254	0.19897	2.36389
H	-1.91084	1.27263	2.48590
C	-1.99518	-0.75743	3.40171
H	-2.12838	-0.54052	4.45129
C	-1.89795	-2.04741	2.82399
H	-1.94661	-2.98570	3.35621
C	-1.71844	-1.89192	1.42943
H	-1.60603	-2.68758	0.70757
C	-2.55307	-0.04268	-1.05685
C	-3.63343	0.85009	-0.96647
C	-4.71158	0.68440	-1.82883
H	-5.55509	1.36118	-1.76401
C	-4.72701	-0.32433	-2.77290
H	-5.57490	-0.43826	-3.43561
C	-3.64561	-1.17849	-2.87279
H	-3.65327	-1.95416	-3.62904
C	-2.54968	-1.05443	-2.02592
C	-3.67816	1.99733	0.02034

H	-2.73739	2.02817	0.56892
C	-3.79758	3.34279	-0.69467
H	-4.72408	3.42365	-1.26429
H	-2.96527	3.49295	-1.38095
H	-3.78959	4.16156	0.02747
C	-4.79571	1.80622	1.04498
H	-4.78157	2.60841	1.78520
H	-4.68987	0.85868	1.57342
H	-5.77945	1.81488	0.57234
C	-1.37509	-1.98254	-2.22640
H	-0.67622	-1.82004	-1.40183
C	-0.65624	-1.63072	-3.52785
H	-0.29454	-0.60249	-3.50657
H	-1.32508	-1.73030	-4.38432
H	0.19095	-2.29583	-3.69798
C	-1.77658	-3.45633	-2.19757
H	-0.89274	-4.09478	-2.25040
H	-2.41435	-3.72262	-3.04154
H	-2.32192	-3.70909	-1.28733
N	-1.46177	0.05907	-0.13616
H	1.06587	-0.60781	-0.52975
O	-0.04564	1.64394	-1.86442
H	-0.38044	2.66703	0.58510
O	-0.57261	4.13307	0.63093
H	-1.46680	4.34378	0.91068
H	-0.12232	2.67319	-1.99200
H	-0.46025	4.23848	-0.66624
O	-0.28098	4.12708	-1.77088
H	-0.97185	4.57378	-2.26123

TS4 (C_1): E_{tot} = -3055.68762144

C	1.61427	-1.00867	0.81116
C	1.31217	-2.39365	0.72574
H	0.98365	-2.91068	-0.16446
C	1.49212	-2.94606	2.01659
H	1.32887	-3.97823	2.28798
C	1.92346	-1.91426	2.88537
H	2.14833	-2.01980	3.93595
C	2.00530	-0.71249	2.14088
H	2.31981	0.25032	2.51273
C	2.66157	0.83238	-0.32318
C	3.89010	0.35875	-0.81258
C	4.97485	1.22611	-0.81174
H	5.93223	0.88129	-1.18129
C	4.85871	2.52164	-0.34316
H	5.71579	3.18197	-0.35004
C	3.64307	2.96688	0.13465
H	3.55987	3.98295	0.49995
C	2.52421	2.13808	0.16032
C	4.10519	-1.06124	-1.30004
H	3.14754	-1.57407	-1.38903
C	4.73200	-1.10054	-2.69299
H	5.74324	-0.69165	-2.70571
H	4.13298	-0.54129	-3.41108
H	4.79605	-2.13108	-3.04396
C	4.93715	-1.85056	-0.28794
H	5.05984	-2.88328	-0.61760
H	4.45933	-1.86737	0.69187
H	5.93278	-1.41929	-0.16903
C	1.23366	2.71259	0.70388
H	0.45558	1.94864	0.66240
C	0.76802	3.89623	-0.14306
H	0.63434	3.61305	-1.18635
H	1.48555	4.71731	-0.11122
H	-0.18313	4.28042	0.22422
C	1.36051	3.12587	2.17064
H	0.40425	3.50014	2.54144
H	2.09467	3.92100	2.30699
H	1.65669	2.29139	2.80565
Fe	0.09233	-1.43618	2.05754
N	1.55756	-0.09597	-0.29226
Si	-0.14813	0.15785	-1.18881
C	-1.50420	-0.57134	1.18018
C	-1.17674	0.12477	2.37959
H	-0.88753	1.16338	2.44813
C	-1.26608	-0.78571	3.45868
H	-1.06239	-0.56231	4.49540
C	-1.67300	-2.04037	2.94095
H	-1.82870	-2.94232	3.51401
C	-1.82230	-1.91260	1.54152
H	-2.10144	-2.70092	0.85994
C	-2.79397	0.23035	-0.70954
C	-3.44194	1.45316	-0.45636
C	-4.68902	1.68128	-1.02484
H	-5.20617	2.61165	-0.82660
C	-5.28397	0.74287	-1.84717
H	-6.25221	0.94121	-2.28815
C	-4.63462	-0.44766	-2.10238
H	-5.10475	-1.17770	-2.74967
C	-3.39379	-0.73449	-1.53927
C	-2.85207	2.51707	0.44185
H	-1.80116	2.27263	0.60544
C	-2.89834	3.90556	-0.19451
H	-3.91967	4.25686	-0.34628
H	-2.39403	3.91952	-1.16070
H	-2.40807	4.63558	0.45188
C	-3.55752	2.52268	1.79945
H	-3.08918	3.23862	2.47839
H	-3.52729	1.54109	2.27126
H	-4.60615	2.80692	1.69305
C	-2.78127	-2.08658	-1.84507
H	-1.83059	-2.17365	-1.32117
C	-2.47277	-2.24271	-3.33385
H	-1.77570	-1.48067	-3.67509
H	-3.37727	-2.18577	-3.94243
H	-2.00868	-3.21193	-3.52127
C	-3.69254	-3.21835	-1.36304
H	-3.19316	-4.18215	-1.47712
H	-4.61909	-3.26562	-1.93766
H	-3.97055	-3.10149	-0.31487
N	-1.50861	-0.02406	-0.12045
O	0.38538	-0.99668	-2.13824
H	1.45077	-0.74219	-1.27218
H	-0.20885	1.53657	-1.72349

TS5 (C_1): E_{tot} = -3062.40989527

C	0.46163	4.06862	3.55398
C	0.30498	3.64852	2.20651
H	1.09279	3.60272	1.46717
C	-1.04734	3.26585	2.02298
H	-1.47936	2.88760	1.10851
C	-1.73550	3.47897	3.24296
H	-2.78334	3.29022	3.42176
C	-0.80744	3.97157	4.19151
H	-1.01634	4.22800	5.21957
C	1.66361	5.70751	4.85633
C	1.65673	5.74844	6.26087
C	1.71796	6.98617	6.89514
H	1.72488	7.03143	7.97764
C	1.75395	8.16077	6.17051
H	1.79469	9.11473	6.67999
C	1.72661	8.11119	4.78785
H	1.74163	9.03740	4.22803
C	1.68819	6.89947	4.10727
C	1.55429	4.50943	7.12514
H	1.46364	3.64222	6.46918
C	2.79866	4.31281	7.98905
H	3.69698	4.23796	7.37663
H	2.71775	3.39674	8.57709
H	2.93609	5.13869	8.68914
C	0.29515	4.53977	7.99311
H	0.32817	5.34419	8.72944
H	0.18567	3.60183	8.54030
H	-0.60401	4.68411	7.39391
C	1.62553	6.90146	2.59501
H	1.95860	5.92440	2.24144
C	2.54750	7.94029	1.95858
H	3.57159	7.84870	2.31882
H	2.21087	8.96214	2.14150
H	2.56952	7.80171	0.87716
C	0.18436	7.12248	2.12645
H	0.12143	7.06441	1.03843
H	-0.17089	8.10995	2.42846
H	-0.49913	6.38157	2.53820
Fe	-0.16101	2.15816	3.51515
N	1.66100	4.44652	4.18449
Si	3.14569	3.52045	4.25943
C	1.53110	1.19810	3.99546
C	0.89580	0.53402	2.90457
H	1.30256	0.40418	1.91494
C	-0.38224	0.10684	3.33171
H	-1.10793	-0.41598	2.72685
C	-0.55153	0.49910	4.68183
H	-1.42612	0.32347	5.29018
C	0.63433	1.14896	5.10060
H	0.81530	1.56775	6.08028
C	3.94406	0.97743	3.93001
C	4.51810	0.63441	2.69413
C	5.64146	-0.18626	2.68804
H	6.09734	-0.45819	1.74385
C	6.18693	-0.66653	3.86257
H	7.05758	-1.30878	3.83558
C	5.61551	-0.32125	5.07261
H	6.04843	-0.70337	5.98886
C	4.49979	0.50608	5.13216
C	3.95979	1.08795	1.36167
H	3.09095	1.72057	1.55292
C	4.96426	1.92509	0.57047
H	5.85173	1.34628	0.30801
H	5.28829	2.79911	1.13071
H	4.51586	2.27171	-0.36236
C	3.50898	-0.11287	0.52462
H	2.97912	0.21929	-0.37002
H	2.85347	-0.78247	1.08139
H	4.36297	-0.70567	0.19259
C	3.91818	0.83658	6.48987
H	3.17546	1.62648	6.36637
C	4.97555	1.37692	7.45084
H	5.47966	2.24743	7.03052
H	5.73663	0.63309	7.69021
H	4.51453	1.67923	8.39219
C	3.20306	-0.37911	7.08036
H	2.73641	-0.12784	8.03496
H	3.90072	-1.19923	7.25986
H	2.42499	-0.74550	6.41094
N	2.79418	1.83373	3.97297
B	5.02157	5.00155	2.79959
N	5.15286	5.48986	4.24089
H	4.05200	4.01541	2.86923
H	5.96784	4.36703	2.42381
H	4.52097	5.76399	2.02297
H	4.39825	4.55584	4.86335
H	6.08517	5.44400	4.62662
H	4.74104	6.39163	4.44530

TS6 (C_1): E_{tot} = -3061.25792805

C	-0.64767	4.00137	4.58065
C	-1.78456	4.24639	3.75946
H	-1.83950	4.96358	2.95386
C	-2.82189	3.38360	4.18447
H	-3.81054	3.32402	3.75485
C	-2.33522	2.60482	5.26324
H	-2.88824	1.84931	5.80108
C	-0.99890	2.99934	5.52395
H	-0.34489	2.59240	6.28269
C	0.63233	6.04120	4.57378
C	0.61491	6.60521	5.85721
C	0.61614	7.99013	5.96968
H	0.59550	8.44685	6.95135

C	0.65935	8.79891	4.84847
H	0.67066	9.87569	4.95611
C	0.69313	8.22827	3.59023
H	0.73512	8.87076	2.71932
C	0.66914	6.84695	3.42672
C	0.57623	5.75593	7.10836
H	0.79604	4.72579	6.82555
C	1.64624	6.16411	8.11912
H	1.64292	5.47601	8.96583
H	1.48006	7.16580	8.51874
H	2.63861	6.13692	7.67016
C	-0.81882	5.78601	7.73410
H	-1.08154	6.79374	8.06195
H	-0.86459	5.13217	8.60694
H	-1.58089	5.45573	7.02823
C	0.71073	6.27350	2.02572
H	0.49332	5.20492	2.08760
C	2.10437	6.43074	1.41585
H	2.87011	5.97217	2.04352
H	2.36640	7.48374	1.29868
H	2.15253	5.96470	0.42934
C	-0.35252	6.88545	1.11426
H	-0.16619	7.94189	0.91692
H	-1.35004	6.80582	1.54787
H	-0.36399	6.37565	0.14962
Fe	-1.13979	2.31407	3.61279
N	0.61475	4.61443	4.43712
Si	2.12681	3.84687	4.09697
C	0.66648	1.66763	3.04052
C	-0.03569	2.15261	1.90079
H	0.27120	2.97882	1.27479
C	-1.22380	1.39801	1.76053
H	-1.97686	1.53769	0.99952
C	-1.24410	0.41814	2.78299
H	-2.01784	-0.31824	2.94043
C	-0.08239	0.57870	3.57308
H	0.17424	-0.00507	4.44247
C	3.04678	1.42512	3.41534
C	3.82724	1.57350	2.25850
C	4.97176	0.79557	2.12259
H	5.58310	0.89615	1.23411
C	5.35408	-0.09392	3.10856
H	6.24861	-0.69060	2.98622
C	4.59602	-0.20625	4.25911
H	4.91291	-0.89061	5.03672
C	3.43845	0.54493	4.43784
C	3.51240	2.58052	1.17456
H	2.61169	3.12852	1.45518
C	4.63904	3.60830	1.06030
H	5.56169	3.15546	0.69304
H	4.85357	4.05882	2.03099
H	4.36550	4.40235	0.36363
C	3.21679	1.90138	-0.16108
H	4.07925	1.34566	-0.53290
H	2.95595	2.64329	-0.91787
H	2.38400	1.20336	-0.07167
C	2.67715	0.38764	5.73718
H	1.80607	1.04516	5.70442
C	3.52682	0.81260	6.93699
H	3.89941	1.82989	6.82658
H	4.38570	0.15248	7.07122
H	2.93759	0.76911	7.85459
C	2.18660	-1.04978	5.92677
H	1.53492	-1.12139	6.79926
H	3.01941	-1.73595	6.08908
H	1.63673	-1.41587	5.05980
N	1.85744	2.21284	3.57155
N	5.06372	3.73919	4.91659
H	5.24057	2.75756	4.80365
H	5.88394	4.26711	5.14386
B	3.75396	4.28877	5.20441
H	3.67506	5.44107	5.53285
H	2.96841	3.60811	5.94698

TS7 (C_1): E_{tot} = -3144.33378985

C	0.63507	3.99864	3.27035
C	0.31719	3.63918	1.92990
H	0.98718	3.71599	1.08611
C	-1.00442	3.13415	1.90321
H	-1.52660	2.76878	1.03142
C	-1.52423	3.19773	3.22011
H	-2.51145	2.88660	3.52823
C	-0.52201	3.72832	4.06299
H	-0.61431	3.88274	5.12686
C	1.94196	5.90683	3.94911
C	1.62003	6.43941	5.20946
C	1.66478	7.81857	5.38921
H	1.42154	8.23904	6.35775
C	2.00943	8.66548	4.35454
H	2.02776	9.73662	4.50860
C	2.34371	8.13237	3.12425
H	2.62918	8.79949	2.32008
C	2.33347	6.75990	2.90388
C	1.24845	5.58316	6.40215
H	1.18637	4.54485	6.07913
C	2.33165	5.64523	7.47966
H	3.30009	5.33247	7.08759
H	2.08157	4.98937	8.31586
H	2.44814	6.65597	7.87464
C	-0.11432	5.96746	6.97886
H	-0.10547	6.96705	7.41612
H	-0.40560	5.27248	7.76876
H	-0.88992	5.95475	6.21268
C	2.75879	6.24446	1.54802
H	2.82530	5.15712	1.61008
C	4.14456	6.76251	1.16277
H	4.89709	6.46604	1.89420
H	4.16727	7.85027	1.08058
H	4.44694	6.36170	0.19454
C	1.72557	6.59579	0.47724
H	2.00230	6.15759	-0.48425
H	1.65243	7.67582	0.33509
H	0.73320	6.23094	0.74274
Fe	0.17800	2.02745	3.17635
N	1.88055	4.49591	3.71875
Si	3.14673	3.44708	4.34273
C	1.97328	1.15599	3.48765
C	1.33386	0.55884	2.36404
H	1.70736	0.54711	1.35322
C	0.11121	-0.00684	2.79729
H	-0.60073	-0.52512	2.17254
C	-0.01940	0.23873	4.18438
H	-0.84755	-0.06161	4.80899
C	1.13513	0.93402	4.61934
H	1.32716	1.27068	5.62691
C	4.31781	1.26476	2.94575
C	4.68608	1.41223	1.59746
C	5.83262	0.76652	1.14187
H	6.13945	0.89135	0.11079
C	6.57729	-0.05022	1.97320
H	7.45343	-0.56041	1.59352
C	6.18334	-0.22611	3.29051
H	6.75482	-0.88936	3.92860
C	5.06501	0.42904	3.79794
C	3.85653	2.19713	0.60447
H	2.96537	2.54905	1.12644
C	4.59056	3.42030	0.06467
H	5.48428	3.13813	-0.49500
H	4.90821	4.08342	0.86360
H	3.94067	3.97953	-0.61198
C	3.41184	1.31082	-0.56309
H	2.68098	1.83643	-1.17973
H	2.96293	0.37363	-0.23364
H	4.25159	1.05193	-1.21031
C	4.64746	0.16928	5.22959
H	3.93405	0.94083	5.52377
C	5.81498	0.23304	6.21377
H	6.37274	1.16717	6.12446
H	6.52335	-0.58461	6.07305
H	5.44889	0.16136	7.23886
C	3.93366	-1.18021	5.33353
H	3.58126	-1.35451	6.35176
H	4.60531	-1.99899	5.06774
H	3.07113	-1.22506	4.67002
N	3.16152	1.92019	3.45162
B	4.86424	4.48646	4.61861
H	2.75757	3.02705	5.73764
H	4.93854	5.62989	4.29647
N	5.70827	4.01811	5.67211
H	6.34066	4.65295	6.13134
H	5.43471	3.25042	6.26267
B	6.96993	4.15653	2.73516
N	7.52791	2.91005	3.58719
H	7.20074	3.96624	1.56778
H	7.45949	5.16472	3.18331
H	5.74597	4.13029	2.93319
H	7.12517	3.00083	4.52475
H	7.22770	2.02113	3.19510
H	8.53832	2.90570	3.65358

TS8 (C_1): E_{tot} = -3144.33218205

C	0.66770	3.99990	3.23126
C	0.34545	3.61873	1.89774
H	1.01666	3.66798	1.05282
C	-0.98325	3.13164	1.87951
H	-1.50948	2.75578	1.01464
C	-1.50424	3.23209	3.19363
H	-2.49643	2.94244	3.50664
C	-0.49516	3.76513	4.02666
H	-0.58620	3.94503	5.08674
C	1.95544	5.90959	3.90710
C	1.64898	6.43573	5.17389
C	1.66343	7.81555	5.35252
H	1.43263	8.23044	6.32647
C	1.96218	8.67093	4.31027
H	1.95628	9.74227	4.46396
C	2.28097	8.14543	3.07280
H	2.52970	8.81864	2.26136
C	2.29971	6.77273	2.85381
C	1.33793	5.56962	6.37637
H	1.26651	4.53316	6.04896
C	2.47604	5.63499	7.39556
H	3.42517	5.34773	6.94175
H	2.28564	4.96213	8.23396
H	2.59611	6.64262	7.79751
C	0.00483	5.93869	7.02715
H	0.02896	6.93260	7.47653
H	-0.24079	5.23196	7.82201
H	-0.80971	5.92997	6.30246
C	2.70854	6.26617	1.48996
H	2.78876	5.17983	1.54944
C	4.08221	6.80352	1.08797
H	4.84321	6.53498	1.82189
H	4.08397	7.89033	0.99100
H	4.38763	6.39464	0.12382
C	1.65657	6.60470	0.43399
H	1.92742	6.17457	-0.53282
H	1.56301	7.68377	0.29676
H	0.67482	6.22108	0.71175
Fe	0.18168	2.03620	3.17859
N	1.91503	4.49853	3.67413
Si	3.21604	3.46256	4.26250
C	1.96849	1.15213	3.50215
C	1.31356	0.52560	2.40421
H	1.68253	0.47488	1.39197
C	0.08700	-0.00901	2.86661
H	-0.63655	-0.54016	2.26647
C	-0.02929	0.28617	4.24572

H	-0.85613	0.01723	4.88620
C	1.13762	0.98237	4.64695
H	1.34531	1.35415	5.63947
C	4.31306	1.24538	2.92398
C	4.68392	1.40915	1.57981
C	5.84641	0.79374	1.12437
H	6.15538	0.93240	0.09542
C	6.60818	-0.00472	1.95598
H	7.50218	-0.48596	1.58040
C	6.20905	-0.20295	3.26801
H	6.79445	-0.85537	3.90455
C	5.07112	0.41733	3.77354
C	3.84196	2.19283	0.59689
H	2.94126	2.51581	1.12051
C	4.55340	3.44196	0.08739
H	5.46435	3.19213	-0.45974
H	4.83556	4.10435	0.90177
H	3.90309	3.99879	-0.59067
C	3.41518	1.31942	-0.58551
H	2.69198	1.84951	-1.20753
H	2.95988	0.38200	-0.26530
H	4.26286	1.06288	-1.22322
C	4.64303	0.13537	5.19789
H	3.95176	0.92184	5.50768
C	5.80617	0.13361	6.18966
H	6.41367	1.03679	6.11408
H	6.47206	-0.71855	6.04538
H	5.42879	0.06794	7.21114
C	3.88808	-1.19454	5.26585
H	3.52755	-1.38446	6.27851
H	4.53854	-2.02402	4.98108
H	3.02684	-1.19945	4.59949
N	3.15871	1.90332	3.43256
B	5.01243	4.38706	4.34481
H	2.90660	3.10511	5.70015
H	4.92695	5.58242	4.32971
N	5.84418	3.83779	5.53948
H	6.22228	4.57553	6.12068
H	5.34016	3.19820	6.14180
B	6.99201	4.06597	2.95969
N	7.55108	2.99755	3.90171
H	6.86682	3.76164	1.81109
H	7.40895	5.16576	3.19250
H	5.63147	4.07061	3.22963
H	6.78435	3.26960	4.92668
H	7.42622	2.05140	3.55899
H	8.52513	3.14161	4.12988

TSS1 (C_1): E_{tot} = -2048.67712442

C	0.69019	3.71749	2.82358
C	0.35123	3.19370	1.54850
H	1.02752	3.10196	0.71094
C	-1.00571	2.78964	1.58455
H	-1.55665	2.34882	0.76698
C	-1.51710	3.07515	2.87510
H	-2.52550	2.89075	3.21438
C	-0.46997	3.64078	3.64457
H	-0.53254	3.95371	4.67729
Fe	0.08002	1.78069	3.00916
N	1.96169	4.16673	3.22012
Si	3.32120	3.12451	3.49721
C	1.71835	0.87633	3.81938
C	1.40637	0.28009	2.56951
H	2.05827	0.25781	1.70821
C	0.09242	-0.24288	2.64633
H	-0.43219	-0.75509	1.85345
C	-0.40892	0.01273	3.94707
H	-1.38179	-0.27045	4.32031
C	0.58987	0.71064	4.67085
H	0.51157	1.06513	5.68896
N	2.90310	1.56481	4.13261
H	3.30669	1.30250	5.01620
H	1.95079	5.04971	3.70247
H	3.40975	2.59260	1.88858
H	4.29880	3.07290	2.34127

TSS2 (C_1): E_{tot} = -2048.68023293

C	0.68020	3.72966	2.44194
C	0.10740	3.25248	1.22833
H	0.61523	3.19220	0.27629
C	-1.22014	2.84900	1.50026
H	-1.91355	2.42889	0.78709
C	-1.48331	3.09349	2.87206
H	-2.41233	2.89202	3.38416
C	-0.31582	3.64315	3.45481
H	-0.18550	3.92333	4.49024
Fe	0.12090	1.81243	2.68146
N	2.00992	4.18161	2.59788
Si	3.51413	3.31784	3.44468
C	1.77472	1.01241	3.50473
C	1.60409	0.49749	2.19035
H	2.30211	0.62028	1.37481
C	0.35115	-0.16093	2.13713
H	-0.07335	-0.64502	1.27012
C	-0.24484	-0.08091	3.42023
H	-1.20296	-0.49196	3.70131
C	0.63061	0.64156	4.26671
H	0.45905	0.89335	5.30344
N	2.84295	1.79860	3.96499
H	2.06522	5.18862	2.68168
H	3.38771	1.34336	4.68313
H	2.88073	3.55102	1.73971
H	3.67445	2.94700	1.66864

TSS3 (C_1): E_{tot} = -2048.67350879

C	1.82448	3.84351	1.32398
C	1.63024	3.23488	0.05839
H	2.39143	3.08838	-0.69423
C	0.26919	2.83803	-0.03837
H	-0.18681	2.35423	-0.88918
C	-0.38239	3.20610	1.16094
H	-1.42533	3.05287	1.39427
C	0.57885	3.81529	2.00932
H	0.39885	4.19179	3.00680
Fe	1.17106	1.91388	1.55554
N	3.03875	4.28090	1.85558
Si	4.54026	3.39292	1.73183
C	2.82872	1.00098	2.29427
C	2.29316	0.24685	1.20934
H	2.80425	0.01870	0.28497
C	0.97772	-0.14130	1.55904
H	0.30754	-0.72612	0.94681
C	0.69059	0.38055	2.84523
H	-0.23427	0.25830	3.38855
C	1.83815	1.07470	3.30504
H	1.94207	1.59021	4.24877
N	4.10331	1.61945	2.32000
H	4.76599	2.55222	3.09914
H	4.84051	3.27120	0.27119
H	4.86291	0.96484	2.17276
H	2.92114	4.82619	2.69448

TSS4 (C_1): E_{tot} = -3062.41379694

C	1.36463	1.51929	0.39800
C	1.03806	1.89265	1.73069
H	0.81263	1.20775	2.53638
C	1.01769	3.30773	1.79657
H	0.78670	3.89812	2.67072
C	1.36321	3.81272	0.51826
H	1.44018	4.85514	0.24787
C	1.58132	2.71381	-0.34589
H	1.85414	2.76605	-1.38946
C	2.69548	-0.15920	-0.66656
C	3.75817	-0.48947	0.18980
C	4.98491	-0.82227	-0.37038
H	5.81815	-1.07132	0.27503
C	5.16172	-0.85470	-1.74205
H	6.12198	-1.12706	-2.16038
C	4.10639	-0.53879	-2.57481
H	4.25192	-0.56944	-3.64778
C	2.86586	-0.17420	-2.05944
C	3.61327	-0.46945	1.69504
H	2.54950	-0.43240	1.93028
C	4.15856	-1.73956	2.34611
H	5.24331	-1.81999	2.25886
H	3.71533	-2.62586	1.89302
H	3.92406	-1.74226	3.41263
C	4.27975	0.77505	2.28369
H	4.12777	0.82189	3.36383
H	3.87547	1.68925	1.85015
H	5.35623	0.76574	2.10074
C	1.75765	0.18214	-3.02837
H	0.92079	0.58821	-2.45652
C	1.25782	-1.05847	-3.76805
H	0.90806	-1.82191	-3.07185
H	2.04886	-1.50367	-4.37414
H	0.43132	-0.80609	-4.43548
C	2.18350	1.26679	-4.01813
H	1.33481	1.57607	-4.63039
H	2.96168	0.91867	-4.69877
H	2.56860	2.15045	-3.50825
Fe	-0.31632	2.60398	0.39557
N	1.42616	0.20731	-0.11250
Si	0.18299	-1.02879	-0.16838
C	-1.65542	1.16782	0.02342
C	-1.52682	1.99979	-1.12605
H	-1.18706	1.68083	-2.10103
C	-1.87069	3.32263	-0.75707
H	-1.85508	4.18557	-1.40589
C	-2.24868	3.31240	0.60782
H	-2.56764	4.16774	1.18450
C	-2.12106	1.98964	1.09131
H	-2.30935	1.66906	2.10328
C	-2.50212	-1.07241	0.10709
C	-3.07412	-1.49097	-1.10577
C	-4.20912	-2.29383	-1.06775
H	-4.66569	-2.61644	-1.99555
C	-4.76608	-2.69309	0.13142
H	-5.64992	-3.31755	0.14101
C	-4.18105	-2.29623	1.31811
H	-4.61441	-2.62311	2.25570
C	-3.04950	-1.48779	1.33220
C	-2.51733	-1.11285	-2.46157
H	-1.57921	-0.57450	-2.31799
C	-2.18636	-2.35060	-3.29461
H	-3.07631	-2.93441	-3.53401
H	-1.49088	-3.00337	-2.76663
H	-1.72449	-2.06160	-4.23984
C	-3.47027	-0.17807	-3.20650
H	-3.03691	0.13559	-4.15826
H	-3.68601	0.71629	-2.62196
H	-4.42034	-0.66889	-3.42512
C	-2.46983	-1.10304	2.67692
H	-1.59432	-0.47382	2.50590
C	-1.99941	-2.33397	3.45337
H	-1.28542	-2.91103	2.86963
H	-2.83517	-2.98703	3.71163
H	-1.51808	-2.03378	4.38652
C	-3.47850	-0.31176	3.51357
H	-3.00990	0.06989	4.42267
H	-4.31732	-0.93830	3.82149
H	-3.89599	0.53229	2.96505
N	-1.34974	-0.21302	0.09007
B	0.73961	-2.96676	1.05112
N	1.32016	-4.33546	2.75845
H	2.15688	-3.99650	3.21047
H	1.51246	-5.24660	2.36894
H	0.59421	-4.43097	3.45370
H	-0.23665	-3.61470	0.82000
H	0.67032	-2.04893	1.85319

H	1.78192	-3.17271	0.50352

TSS5 (C_1): E_{tot} = -2980.51101763

C	1.47043	-0.63355	1.07425
C	1.22356	-2.03455	1.00454
H	0.94398	-2.58090	0.11554
C	1.37318	-2.57559	2.30347
H	1.23913	-3.61112	2.57856
C	1.73068	-1.52044	3.17944
H	1.91401	-1.61131	4.23977
C	1.79282	-0.32458	2.42850
H	2.02221	0.65487	2.81931
C	2.64884	0.62967	-0.61449
C	3.17805	-0.15785	-1.64992
C	4.41975	0.17863	-2.17817
H	4.84643	-0.42904	-2.96653
C	5.12254	1.27708	-1.72204
H	6.08609	1.52350	-2.14860
C	4.58093	2.06269	-0.72386
H	5.12796	2.93175	-0.37876
C	3.35000	1.75623	-0.15332
C	2.46327	-1.36380	-2.21725
H	1.45364	-1.38858	-1.80210
C	2.32631	-1.27268	-3.73707
H	3.29571	-1.28479	-4.23691
H	1.80935	-0.36127	-4.03714
H	1.75886	-2.12123	-4.12124
C	3.17554	-2.65674	-1.81624
H	2.61904	-3.53005	-2.16271
H	3.28927	-2.73411	-0.73533
H	4.17313	-2.70838	-2.25639
C	2.81982	2.67376	0.92749
H	1.88571	2.25989	1.31027
C	2.49452	4.05642	0.36065
H	1.80672	3.99219	-0.48384
H	3.39190	4.56625	0.00604
H	2.03482	4.68790	1.12270
C	3.78726	2.78424	2.10619
H	3.34243	3.37067	2.91211
H	4.71860	3.27760	1.82405
H	4.04675	1.80283	2.50385
Fe	-0.08109	-1.11720	2.27283
N	1.39709	0.27346	-0.01198
Si	-0.06310	1.07429	-0.51812
C	-1.63279	-0.24369	1.33698
C	-1.38251	0.42585	2.56609
H	-1.08812	1.46057	2.67011
C	-1.52660	-0.51688	3.61255
H	-1.38528	-0.31954	4.66485
C	-1.89333	-1.75984	3.04067
H	-2.07916	-2.67673	3.57981
C	-1.96036	-1.59549	1.63707
H	-2.20844	-2.36105	0.91712
C	-2.69759	0.29611	-0.75571
C	-3.65271	1.30266	-0.55236
C	-4.82423	1.26949	-1.29958
H	-5.57722	2.03347	-1.14858
C	-5.04229	0.28125	-2.24209
H	-5.95665	0.27415	-2.82094
C	-4.08476	-0.69472	-2.44558
H	-4.26002	-1.45956	-3.19241
C	-2.90682	-0.71268	-1.70575
C	-3.45247	2.41028	0.45880
H	-2.42918	2.35350	0.83294
C	-3.61466	3.79341	-0.17064
H	-4.63435	3.97432	-0.51425
H	-2.95066	3.92010	-1.02636
H	-3.37694	4.57099	0.55702
C	-4.38645	2.22929	1.65529
H	-4.20922	3.00324	2.40426
H	-4.23278	1.26091	2.13140
H	-5.43432	2.29303	1.35563
C	-1.88307	-1.79111	-1.97999
H	-1.11357	-1.73494	-1.20686
C	-1.20529	-1.54617	-3.32706
H	-0.71491	-0.57174	-3.35433
H	-1.92750	-1.57319	-4.14491
H	-0.44999	-2.30759	-3.52671
C	-2.48341	-3.19517	-1.92263
H	-1.69952	-3.94785	-2.02090
H	-3.19895	-3.36936	-2.72751
H	-3.00425	-3.37407	-0.98136
N	-1.50868	0.29304	0.03683
H	-0.07514	2.58301	-0.41106
H	-0.12505	2.35514	0.71243

TSS6 (C_1): E_{tot} = -2980.51348765

C	1.50360	3.02707	2.07646
C	1.16213	2.06350	1.08359
H	1.74552	1.82631	0.20546
C	-0.05240	1.44464	1.46547
H	-0.56085	0.66005	0.92517
C	-0.48625	2.04053	2.67561
H	-1.38172	1.78594	3.22276
C	0.46397	3.01727	3.05143
H	0.42412	3.63295	3.93713
C	2.37571	5.23734	1.81379
C	2.22715	6.13866	2.88041
C	1.94774	7.47133	2.59434
H	1.83692	8.18029	3.40576
C	1.80633	7.90946	1.29203
H	1.58001	8.94801	1.08860
C	1.96526	7.01566	0.24935
H	1.86263	7.36892	-0.76941
C	2.26233	5.67853	0.48490
C	2.38364	5.73022	4.33050
H	2.48246	4.64440	4.37518
C	3.66070	6.32565	4.92457
H	3.80117	5.99049	5.95363
H	3.62475	7.41658	4.93165
H	4.54085	6.02748	4.35466
C	1.16627	6.11733	5.17096
H	0.23888	5.73908	4.73943
H	1.06107	7.19919	5.26478
H	1.25821	5.71469	6.18101
C	2.44749	4.75722	-0.70028
H	2.81679	3.79928	-0.33178
C	3.49888	5.29086	-1.67262
H	4.45370	5.45123	-1.17188
H	3.19760	6.23492	-2.12877
H	3.66088	4.58022	-2.48513
C	1.11683	4.50531	-1.40961
H	1.24306	3.79379	-2.22812
H	0.71439	5.42672	-1.83472
H	0.37139	4.10198	-0.72464
Fe	1.39080	1.20775	2.91289
N	2.64857	3.85261	2.08398
Si	4.36806	3.52577	2.30863
C	3.32106	0.85991	3.34058
C	2.77107	-0.28060	2.69617
H	3.11611	-0.70800	1.76701
C	1.69905	-0.75449	3.49045
H	1.07509	-1.60576	3.26370
C	1.58528	0.08398	4.62548
H	0.85857	-0.01326	5.41811
C	2.59643	1.07357	4.54688
H	2.76603	1.86927	5.25848
C	5.53150	0.88064	2.41946
C	5.77083	0.70652	1.04807
C	6.87079	-0.05059	0.65940
H	7.07478	-0.19081	-0.39523
C	7.70369	-0.63958	1.59123
H	8.54941	-1.23206	1.26794
C	7.44928	-0.46721	2.93900
H	8.10400	-0.93401	3.66436
C	6.37455	0.29667	3.37834
C	4.88995	1.29479	-0.03365
H	4.05603	1.82221	0.43779
C	5.65926	2.30190	-0.88821
H	6.47436	1.82125	-1.43159
H	6.08432	3.09647	-0.27581
H	5.00079	2.76309	-1.62564
C	4.26534	0.21080	-0.91315
H	3.58932	0.65756	-1.64452
H	3.69310	-0.50866	-0.32784
H	5.02218	-0.34550	-1.46776
C	6.14042	0.43587	4.86849
H	5.38365	1.20265	5.04056
C	7.39444	0.89828	5.60919
H	7.78638	1.82377	5.18748
H	8.19151	0.15410	5.57862
H	7.16469	1.07934	6.66013
C	5.59911	-0.86970	5.45259
H	5.39303	-0.75859	6.51843
H	6.31908	-1.68205	5.33640
H	4.67287	-1.16929	4.96242
N	4.40559	1.64430	2.86955
H	4.58967	2.52809	3.84967
H	4.53233	3.51122	4.14922

TSS7 (C_1): E_{tot} = -2980.50682419

C	1.80279	3.82236	1.39144
C	1.67725	3.23907	0.10291
H	2.47701	3.10897	-0.61292
C	0.32814	2.82975	-0.06454
H	-0.08114	2.34749	-0.93974
C	-0.38645	3.18330	1.10433
H	-1.43817	3.01552	1.28157
C	0.52136	3.78758	2.00891
H	0.29045	4.16541	2.99420
C	2.90302	5.47960	2.73850
C	2.93221	5.48882	4.14147
C	2.83686	6.70926	4.80292
H	2.86432	6.73171	5.88594
C	2.69364	7.89371	4.10634
H	2.60820	8.83191	4.63894
C	2.66656	7.87346	2.72341
H	2.56205	8.80643	2.18328
C	2.78720	6.68204	2.01872
C	3.05869	4.22757	4.96450
H	3.08412	3.38722	4.27240
C	4.36194	4.20538	5.76123
H	4.46687	3.26275	6.30294
H	4.39896	5.01075	6.49690
H	5.22274	4.31005	5.10146
C	1.85019	4.02160	5.87712
H	0.91989	3.99543	5.30871
H	1.75988	4.81574	6.61975
H	1.93547	3.07842	6.42098
C	2.77717	6.71457	0.50475
H	3.09288	5.73631	0.13964
C	3.76902	7.73380	-0.05547
H	4.77417	7.56732	0.33271
H	3.48740	8.76080	0.18189
H	3.81297	7.65718	-1.14298
C	1.36670	6.97353	-0.02661
H	1.35697	6.96036	-1.11790
H	0.99747	7.94879	0.29706
H	0.66444	6.21649	0.32165
Fe	1.17066	1.90115	1.56337
N	2.98171	4.25470	2.01180
Si	4.53503	3.44533	1.85305
C	2.83579	0.96704	2.21548
C	2.21969	0.19537	1.18697
H	2.66434	-0.07040	0.24145
C	0.91915	-0.15226	1.61847
H	0.20113	-0.72960	1.05545
C	0.71997	0.39942	2.90658
H	-0.17395	0.31411	3.50586

C	1.90715	1.07276	3.28547
H	2.06357	1.59695	4.21632
C	5.21108	0.59670	2.01030
C	5.75846	0.29397	0.75463
C	6.82296	-0.60144	0.69460
H	7.26316	-0.84076	-0.26563
C	7.32565	-1.19983	1.83244
H	8.14970	-1.89761	1.76169
C	6.76723	-0.90539	3.06243
H	7.16136	-1.38361	3.95050
C	5.71519	-0.00619	3.17773
C	5.24012	0.86542	-0.54878
H	4.37525	1.49555	-0.33352
C	6.29618	1.73271	-1.23600
H	7.15559	1.13665	-1.54773
H	6.66210	2.52069	-0.57953
H	5.88392	2.20488	-2.12920
C	4.77795	-0.24055	-1.50141
H	4.29357	0.19110	-2.37865
H	4.07315	-0.92478	-1.02911
H	5.61597	-0.84136	-1.85741
C	5.13801	0.25483	4.55430
H	4.43757	1.08941	4.49492
C	6.20961	0.66271	5.56380
H	6.76690	1.53331	5.21805
H	6.92439	-0.13847	5.75628
H	5.74872	0.91849	6.51920
C	4.34287	-0.95855	5.03800
H	3.88474	-0.75705	6.00788
H	4.98517	-1.83392	5.15037
H	3.54791	-1.21362	4.33737
N	4.13566	1.53972	2.14584
H	4.68753	2.40341	3.05680
H	4.91267	3.51317	0.40765

TSS8 (C_1): E_{tot} = -3131.91949474

C	1.29747	3.27971	1.94334
C	0.74199	2.72850	0.75771
H	1.16060	2.80636	-0.23496
C	-0.45963	2.06854	1.11566
H	-1.11156	1.53891	0.43718
C	-0.65763	2.23137	2.50731
H	-1.48646	1.84742	3.08279
C	0.42516	2.98474	3.02226
H	0.55481	3.29590	4.04683
C	2.47128	5.06700	3.04944
C	3.10296	4.89348	4.29206
C	3.07930	5.95457	5.19605
H	3.57545	5.84155	6.15116
C	2.42882	7.13754	4.91968
H	2.43455	7.94697	5.63777
C	1.73737	7.25920	3.73166
H	1.17909	8.16582	3.53497
C	1.72451	6.23626	2.79115
C	3.73821	3.61136	4.79069
H	3.79682	2.89268	3.97554
C	5.15190	3.82641	5.33009
H	5.60773	2.86617	5.57020
H	5.14844	4.41404	6.24952
H	5.78964	4.32418	4.60622
C	2.87639	2.98357	5.89139
H	1.87543	2.73216	5.54413
H	2.77178	3.65285	6.74661
H	3.33888	2.06366	6.25621
C	0.82142	6.43931	1.58476
H	0.90685	5.60626	0.89328
C	1.19223	7.69396	0.79484
H	2.24411	7.69111	0.50808
H	1.01276	8.60880	1.36155
H	0.59839	7.75410	-0.11777
C	-0.64346	6.47778	2.02501
H	-1.29542	6.57740	1.15577
H	-0.85034	7.31640	2.69197
H	-0.91981	5.55931	2.54285
Fe	1.13282	1.27430	2.14146
N	2.50518	4.05695	1.99751
Si	4.18040	3.14768	1.30342
C	3.03613	0.62693	2.05450
C	2.22085	-0.14660	1.17563
H	2.33335	-0.21742	0.10531
C	1.21649	-0.77825	1.94257
H	0.43584	-1.41824	1.55887
C	1.40060	-0.40994	3.29793
H	0.78938	-0.72440	4.13084
C	2.53258	0.43453	3.37236
H	2.93292	0.87530	4.27169
C	5.28702	0.72090	1.20671
C	5.47547	0.49526	-0.16997
C	6.61789	-0.18590	-0.58321
H	6.78087	-0.35867	-1.64015
C	7.54200	-0.66040	0.32533
H	8.41583	-1.19944	-0.01706
C	7.34257	-0.44220	1.67582
H	8.06938	-0.81968	2.38444
C	6.23495	0.25848	2.13626
C	4.50173	0.93702	-1.24540
H	3.63690	1.40185	-0.76935
C	5.12195	1.98610	-2.16806
H	5.97477	1.58320	-2.71724
H	5.46384	2.85314	-1.60450
H	4.39030	2.33361	-2.89915
C	3.99636	-0.25351	-2.06465
H	3.20317	0.06206	-2.74457
H	3.60435	-1.04918	-1.43067
H	4.78888	-0.69064	-2.67389
C	6.07682	0.46963	3.62458
H	5.27179	1.18852	3.77475
C	7.33364	1.06885	4.25237
H	7.58636	2.01918	3.78331
H	8.19692	0.40726	4.16745
H	7.17787	1.25069	5.31712
C	5.68197	-0.83638	4.31513
H	5.50856	-0.67374	5.38102
H	6.46795	-1.58833	4.22079
H	4.77051	-1.25303	3.88646
N	4.13402	1.42841	1.68559
O	5.43950	3.74311	2.22424
H	5.88632	4.48694	1.81546
H	2.52573	4.62997	0.96331
H	3.70698	4.02993	-0.08767
O	2.78993	4.89221	-0.34496
H	3.19809	5.75204	-0.48187

TSS9 (C_1): E_{tot} = -3208.19847345

C	0.35775	4.52503	4.31802
C	0.01205	4.14005	2.99100
H	0.66161	4.19955	2.13052
C	-1.31344	3.64601	2.99907
H	-1.85538	3.26776	2.14479
C	-1.80344	3.72787	4.32711
H	-2.78358	3.42099	4.66138
C	-0.78136	4.26815	5.13923
H	-0.84593	4.43809	6.20307
C	1.61715	6.44464	5.01473
C	1.40874	6.93716	6.32041
C	1.47112	8.30907	6.55031
H	1.32941	8.68404	7.55681
C	1.68968	9.20699	5.52736
H	1.72799	10.26987	5.72717
C	1.84254	8.72879	4.24152
H	1.98871	9.43369	3.43288
C	1.81405	7.36668	3.96424
C	1.08212	6.07245	7.52211
H	1.00604	5.03396	7.20134
C	2.19215	6.13389	8.57108
H	3.15411	5.84953	8.14227
H	1.96713	5.46634	9.40617
H	2.30352	7.13862	8.98194
C	-0.26614	6.45463	8.13592
H	-0.24850	7.45146	8.57913
H	-0.53591	5.75251	8.92711
H	-1.06079	6.44568	7.38950
C	1.93536	6.94630	2.51826
H	2.17438	5.88337	2.50446
C	3.04518	7.68861	1.77144
H	3.98191	7.69775	2.32878
H	2.77817	8.72680	1.56836
H	3.23333	7.21808	0.80472
C	0.60445	7.15147	1.78971
H	0.65585	6.76637	0.76864
H	0.36025	8.21402	1.72971
H	-0.21871	6.65156	2.29674
Fe	-0.11301	2.55071	4.24950
N	1.60710	5.03485	4.73496
Si	2.74138	3.94290	5.64548
C	1.65042	1.56652	4.60789
C	1.03658	1.09419	3.41576
H	1.48046	1.11577	2.43096
C	-0.23767	0.56888	3.74977
H	-0.94187	0.13214	3.05725
C	-0.42630	0.73080	5.14223
H	-1.29973	0.43868	5.70611
C	0.73684	1.34149	5.67831
H	0.88749	1.60312	6.71513
C	3.85650	1.58929	3.72273
C	4.39830	2.34911	2.67483
C	5.35432	1.77815	1.83528
H	5.78390	2.37877	1.04336
C	5.75205	0.46788	1.96733
H	6.49781	0.04561	1.30709
C	5.15238	-0.30574	2.94090
H	5.42463	-1.35110	3.01935
C	4.20728	0.21589	3.81421
C	3.96615	3.75134	2.31879
H	3.23822	4.10191	3.04735
C	5.15168	4.71705	2.30879
H	5.82693	4.51850	1.47509
H	5.74029	4.63859	3.22237
H	4.81364	5.74666	2.19752
C	3.25714	3.75183	0.96180
H	2.89859	4.75187	0.70945
H	2.39892	3.07861	0.96428
H	3.92068	3.43273	0.15671
C	3.58680	-0.79222	4.77261
H	2.87986	-0.29704	5.43620
C	4.64567	-1.49937	5.62903
H	5.36568	-0.80795	6.07151
H	5.23079	-2.20523	5.03879
H	4.17450	-2.07696	6.42742
C	2.76005	-1.83465	4.01624
H	2.28827	-2.52636	4.71633
H	3.37977	-2.42212	3.33715
H	1.97118	-1.36392	3.43348
N	2.95095	2.14099	4.70919
O	4.05494	1.41219	6.92010
O	2.75030	2.69126	8.45174
H	3.48982	1.94175	7.75829
H	2.52705	3.28049	7.57325
H	3.27273	3.27134	9.01746
H	4.06899	0.45627	7.02565
H	3.57700	1.67294	5.95849
O	4.21172	4.71611	5.44805
H	4.30519	5.32279	4.70880

TSS10 (C_1): E_{tot} = -3055.63302411

C	1.17225	3.22262	2.13710
C	0.61369	2.72907	0.92415
H	1.02984	2.84958	-0.06594
C	-0.57607	2.03078	1.24947

H	-1.22551	1.53276	0.54522
C	-0.76696	2.11102	2.64888
H	-1.58798	1.68401	3.20475
C	0.30891	2.84948	3.20013
H	0.44965	3.09571	4.24157
C	2.36560	5.02501	3.17792
C	2.98125	4.93587	4.43546
C	2.88611	6.02283	5.29899
H	3.34944	5.96782	6.27660
C	2.20650	7.17062	4.94179
H	2.14137	8.00253	5.63060
C	1.61360	7.24976	3.69523
H	1.08507	8.15344	3.41813
C	1.68336	6.19536	2.79419
C	3.72344	3.70948	4.91823
H	3.77699	2.99355	4.09793
C	5.15249	4.05174	5.34257
H	5.72182	3.14301	5.54587
H	5.17008	4.64779	6.25611
H	5.67475	4.62476	4.57701
C	2.98540	3.01839	6.06510
H	1.98683	2.69937	5.76755
H	2.87885	3.67666	6.92869
H	3.53162	2.13203	6.39547
C	0.99722	6.34756	1.45152
H	1.28607	5.51790	0.80474
C	1.42781	7.62305	0.72788
H	2.51135	7.67171	0.62088
H	1.10311	8.52490	1.24869
H	0.99012	7.65376	-0.27093
C	-0.52280	6.28438	1.60808
H	-1.01256	6.35784	0.63573
H	-0.89027	7.10460	2.22773
H	-0.83600	5.34820	2.06999
Fe	1.04254	1.21048	2.21978
N	2.39378	3.93096	2.26416
Si	3.98257	3.19738	1.28057
C	2.96973	0.62836	2.04726
C	2.14722	-0.16709	1.19445
H	2.22694	-0.23194	0.12021
C	1.19604	-0.83869	1.99419
H	0.42708	-1.50709	1.63630
C	1.41693	-0.47086	3.34476
H	0.84954	-0.81243	4.19776
C	2.51712	0.41828	3.38155
H	2.92822	0.87973	4.26707
C	5.18984	0.77628	1.14166
C	5.36085	0.57885	-0.24026
C	6.50989	-0.07109	-0.68322
H	6.66115	-0.22017	-1.74541
C	7.45861	-0.54129	0.20232
H	8.33877	-1.05377	-0.16337
C	7.27702	-0.35126	1.55970
H	8.02412	-0.72481	2.24917
C	6.16107	0.31592	2.04881
C	4.36076	1.03286	-1.28544
H	3.48350	1.44008	-0.77903
C	4.93421	2.15188	-2.15464
H	5.81577	1.81981	-2.70580
H	5.22008	3.01448	-1.55261
H	4.19481	2.48839	-2.88339
C	3.88721	-0.13418	-2.15441
H	3.08139	0.18776	-2.81607
H	3.51997	-0.96569	-1.55213
H	4.68719	-0.52230	-2.78661
C	6.01998	0.49732	3.54423
H	5.20251	1.19892	3.72201
C	7.27843	1.09053	4.17789
H	7.56721	2.03156	3.70654
H	8.13316	0.41715	4.10498
H	7.11609	1.28324	5.23957
C	5.64608	-0.82430	4.21616
H	5.48477	-0.68421	5.28699
H	6.43992	-1.56382	4.09534
H	4.73422	-1.24143	3.79017
N	4.03726	1.45870	1.64865
O	5.32800	3.86119	1.98650
H	5.71936	3.37898	2.72078
H	2.77130	4.22364	0.99402

TSS11 (C_1): E_{tot} = -3055.62830834

C	1.51638	-1.09022	0.80689
C	1.06729	-2.41551	0.54964
H	0.65160	-2.77258	-0.38332
C	1.21828	-3.17148	1.73883
H	0.95301	-4.21033	1.86614
C	1.79996	-2.33293	2.71980
H	2.05279	-2.61843	3.72982
C	1.98145	-1.04899	2.15248
H	2.38667	-0.18536	2.65600
C	2.60075	0.70640	-0.41848
C	3.41951	0.20905	-1.44806
C	4.53819	0.94444	-1.82380
H	5.18293	0.57334	-2.61069
C	4.84148	2.14926	-1.21580
H	5.71023	2.71273	-1.53032
C	4.03639	2.62596	-0.19830
H	4.29045	3.56230	0.28397
C	2.91686	1.91618	0.22786
C	3.14323	-1.11691	-2.12269
H	2.12619	-1.42175	-1.87588
C	3.20954	-1.02893	-3.64548
H	4.21196	-0.79735	-4.00874
H	2.52835	-0.26744	-4.02595
H	2.92081	-1.98296	-4.08847
C	4.09015	-2.18899	-1.58141
H	3.86886	-3.15972	-2.02832
H	3.99547	-2.28984	-0.50020
H	5.13103	-1.94853	-1.80668
C	2.12710	2.44350	1.40953
H	1.31960	1.73784	1.60745
C	1.49567	3.81279	1.15336
H	0.76302	3.78640	0.34778
H	2.24639	4.56428	0.90233
H	0.97726	4.16192	2.04817
C	3.01448	2.51263	2.65572
H	2.41689	2.74321	3.53919
H	3.77322	3.29164	2.56385
H	3.54008	1.57536	2.83793
Fe	0.00476	-1.53376	2.05575
N	1.40872	0.01100	-0.06327
Si	-0.01664	0.27112	-1.07468
C	-1.49166	-0.46869	1.23655
C	-1.04245	0.18064	2.41870
H	-0.61528	1.17166	2.46940
C	-1.21119	-0.71318	3.50462
H	-0.94843	-0.51600	4.53325
C	-1.80045	-1.90223	3.00830
H	-2.06212	-2.77210	3.59194
C	-1.97363	-1.75729	1.61333
H	-2.38845	-2.49548	0.94384
C	-2.68883	0.24453	-0.73145
C	-3.37429	1.44334	-0.47678
C	-4.59956	1.65510	-1.09756
H	-5.14693	2.56903	-0.90168
C	-5.12847	0.72341	-1.97171
H	-6.07963	0.90820	-2.45389
C	-4.43517	-0.44370	-2.22979
H	-4.85410	-1.16473	-2.92134
C	-3.21687	-0.71211	-1.61333
C	-2.83371	2.49877	0.46282
H	-1.80638	2.23623	0.71210
C	-2.78313	3.87879	-0.19042
H	-3.77648	4.26263	-0.42857
H	-2.19947	3.85393	-1.10971
H	-2.31401	4.59670	0.48487
C	-3.63953	2.52757	1.76179
H	-3.21862	3.25209	2.46174
H	-3.64182	1.55267	2.24944
H	-4.67778	2.81139	1.57858
C	-2.52006	-2.01781	-1.93504
H	-1.64152	-2.10646	-1.29264
C	-2.03059	-2.04187	-3.38339
H	-1.35047	-1.21322	-3.57772
H	-2.86264	-1.97040	-4.08619
H	-1.49937	-2.97192	-3.59538
C	-3.41540	-3.22451	-1.64865
H	-2.85748	-4.15298	-1.78095
H	-4.27198	-3.26427	-2.32312
H	-3.80715	-3.20796	-0.63106
N	-1.43216	0.02007	-0.08597
O	0.12426	2.12103	-1.46912
H	1.03278	2.46458	-1.43985
H	0.03692	1.26003	-2.37954

TSS12 (C_1): E_{tot} = -3131.90660263

C	1.37864	-0.51548	1.48118
C	1.21917	-1.92521	1.55163
H	1.08314	-2.59275	0.71129
C	1.24789	-2.29575	2.91636
H	1.14469	-3.29957	3.29924
C	1.44595	-1.12327	3.68332
H	1.51491	-1.07042	4.75930
C	1.52116	-0.01783	2.80343
H	1.63969	1.00753	3.10571
C	2.67438	0.70926	-0.20257
C	3.33724	-0.02032	-1.20182
C	4.61216	0.38606	-1.57514
H	5.14648	-0.17091	-2.33380
C	5.20966	1.49118	-1.00496
H	6.20344	1.79238	-1.30798
C	4.51881	2.21981	-0.06058
H	4.97935	3.10305	0.36208
C	3.24228	1.85859	0.36146
C	2.76660	-1.22369	-1.93273
H	1.71986	-1.39829	-1.66279
C	2.74085	-0.97983	-3.44122
H	3.74348	-0.86419	-3.85311
H	2.17026	-0.08150	-3.67292
H	2.27397	-1.82084	-3.95439
C	3.52220	-2.50109	-1.56824
H	3.07631	-3.36529	-2.06324
H	3.50883	-2.68030	-0.49247
H	4.56657	-2.44768	-1.87820
C	2.59383	2.76132	1.39261
H	1.59030	2.39551	1.61530
C	2.41715	4.18482	0.85943
H	1.81333	4.21200	-0.04648
H	3.37981	4.65399	0.64901
H	1.91814	4.79801	1.61241
C	3.41064	2.78607	2.68931
H	2.86151	3.30858	3.47481
H	4.35103	3.32116	2.54990
H	3.66029	1.78837	3.05016
Fe	-0.27067	-0.94023	2.55519
N	1.36263	0.21752	0.23403
Si	-0.22880	1.38555	-0.28449
C	-1.75164	-0.20737	1.38823
C	-1.64907	0.56679	2.57791
H	-1.42195	1.62292	2.62461
C	-1.85852	-0.29453	3.68353
H	-1.83340	-0.00593	4.72391
C	-2.12449	-1.59275	3.18473
H	-2.33691	-2.46956	3.77794
C	-2.05497	-1.54384	1.77211
H	-2.20428	-2.37353	1.09777
C	-2.58631	0.04098	-0.84114
C	-3.65364	0.95159	-0.83427

C	-4.72370	0.73350	-1.69458
H	-5.55820	1.42443	-1.69577
C	-4.73966	-0.34776	-2.55532
H	-5.57897	-0.50174	-3.22089
C	-3.67344	-1.22735	-2.56657
H	-3.68737	-2.06517	-3.25330
C	-2.58707	-1.05334	-1.71614
C	-3.68375	2.16416	0.07138
H	-2.73379	2.22783	0.60286
C	-3.81849	3.45800	-0.73068
H	-4.76923	3.51706	-1.26228
H	-3.02119	3.54281	-1.46954
H	-3.75974	4.32391	-0.06930
C	-4.78210	2.03804	1.12616
H	-4.77099	2.89722	1.79914
H	-4.64700	1.13882	1.72759
H	-5.77275	1.98933	0.67037
C	-1.43866	-2.03282	-1.79524
H	-0.75098	-1.80284	-0.97689
C	-0.68559	-1.86004	-3.11341
H	-0.32020	-0.83962	-3.22227
H	-1.33240	-2.07657	-3.96545
H	0.16299	-2.54609	-3.16917
C	-1.89040	-3.48185	-1.61589
H	-1.02787	-4.15002	-1.58245
H	-2.52216	-3.81527	-2.44039
H	-2.45937	-3.62278	-0.69626
N	-1.50532	0.22336	0.07685
H	1.11075	-0.48491	-0.45958
O	0.02030	1.40873	-1.88776
H	-0.15857	2.98351	-0.15970
O	0.02908	3.77700	-1.40090
H	-0.77700	4.27121	-1.57059
H	0.05267	2.49200	-1.96322

TSS13 (C_1): E_{tot} = -3055.59499167

C	1.29194	-0.80846	1.22916
C	0.73734	-2.11463	1.19193
H	0.34433	-2.61665	0.31852
C	0.75473	-2.62920	2.51133
H	0.39273	-3.60050	2.81233
C	1.33690	-1.65323	3.35324
H	1.49781	-1.74298	4.41686
C	1.67099	-0.52610	2.56602
H	2.12799	0.37690	2.93059
C	2.73622	0.62672	-0.12914
C	3.61438	-0.06552	-0.97991
C	4.85387	0.49818	-1.24847
H	5.53946	-0.00886	-1.91377
C	5.23119	1.69926	-0.67890
H	6.20068	2.12590	-0.89918
C	4.36612	2.34684	0.17691
H	4.67404	3.28237	0.62605
C	3.10484	1.83529	0.47606
C	3.24826	-1.37051	-1.66539
H	2.46410	-1.87743	-1.09395
C	2.71797	-1.09204	-3.07444
H	3.49946	-0.64384	-3.69045
H	1.87201	-0.40329	-3.07002
H	2.40259	-2.01794	-3.55860
C	4.40300	-2.37016	-1.70045
H	4.04620	-3.32995	-2.07507
H	4.82473	-2.53208	-0.70870
H	5.20761	-2.05217	-2.36409
C	2.26089	2.63284	1.45346
H	1.32179	2.10419	1.63034
C	1.90553	4.01561	0.90667
H	1.34290	3.93781	-0.02008
H	2.79798	4.61528	0.72197
H	1.29019	4.55775	1.62709
C	2.97654	2.79656	2.80059
H	2.29155	3.20431	3.54526
H	3.81542	3.48934	2.72228
H	3.37907	1.86048	3.18709
Fe	-0.33891	-0.88358	2.41697
N	1.41176	0.05114	0.07959
Si	-0.18049	1.33357	-0.63543
C	-1.72948	0.13678	1.34704
C	-1.29967	0.92365	2.44959
H	-0.83625	1.89858	2.38602
C	-1.57825	0.20497	3.64058
H	-1.36413	0.53880	4.64492
C	-2.20966	-1.00930	3.28083
H	-2.55696	-1.77131	3.96233
C	-2.29842	-1.05793	1.86814
H	-2.72656	-1.85570	1.27943
C	-2.69221	0.20408	-0.84548
C	-3.79229	1.07104	-0.73075
C	-4.92913	0.80761	-1.48406
H	-5.79155	1.45708	-1.39683
C	-4.97591	-0.26519	-2.35604
H	-5.86818	-0.45444	-2.93852
C	-3.87250	-1.08544	-2.48983
H	-3.91067	-1.91272	-3.18832
C	-2.71664	-0.87035	-1.74515
C	-3.77886	2.27513	0.18715
H	-2.75640	2.43107	0.53452
C	-4.18593	3.55380	-0.54462
H	-5.22758	3.53293	-0.86859
H	-3.56601	3.71912	-1.42583
H	-4.07112	4.41658	0.11344
C	-4.65818	2.03986	1.41571
H	-4.61175	2.89451	2.09296
H	-4.34189	1.15689	1.97098
H	-5.70284	1.89920	1.13092
C	-1.54965	-1.81215	-1.94013
H	-0.73449	-1.46230	-1.30711
C	-1.04627	-1.78788	-3.38158
H	-0.76617	-0.77614	-3.66795
H	-1.80180	-2.15116	-4.08018
H	-0.16915	-2.42934	-3.49079
C	-1.89405	-3.23385	-1.49739
H	-1.03091	-3.89374	-1.61000
H	-2.70356	-3.65714	-2.09408
H	-2.20571	-3.26749	-0.45262
N	-1.55438	0.42320	-0.01141
O	0.06803	1.18034	-2.20495
H	1.24301	-0.54245	-0.72983
H	0.33843	2.46496	-1.52628

TSS14 (C_1): E_{tot} = -3035.84529028

C	-0.71615	1.26340	1.27934
C	0.44356	1.71577	1.97251
H	1.38085	2.00281	1.51853
C	0.17181	1.67369	3.36187
H	0.86038	1.94014	4.14980
C	-1.16162	1.22736	3.53535
H	-1.66692	1.09002	4.47966
C	-1.71276	0.97888	2.25724
H	-2.70434	0.60417	2.05543
C	-1.63225	2.17318	-0.71528
C	-1.00561	3.38693	-1.04888
C	-1.78223	4.43193	-1.53704
H	-1.31150	5.37584	-1.78291
C	-3.14382	4.29243	-1.71997
H	-3.73225	5.11812	-2.09819
C	-3.74365	3.08422	-1.42806
H	-4.80859	2.97190	-1.59186
C	-3.01144	2.01239	-0.92643
C	0.48232	3.62335	-0.90765
H	0.95808	2.68187	-0.62890
C	1.10206	4.05933	-2.23548
H	0.70316	5.01436	-2.57988
H	0.92154	3.32174	-3.01739
H	2.18107	4.18139	-2.13194
C	0.77675	4.64424	0.19217
H	1.85315	4.75396	0.33930
H	0.33388	4.34725	1.14232
H	0.38024	5.62829	-0.06475
C	-3.75284	0.71856	-0.66768
H	-3.05959	-0.00858	-0.24270
C	-4.27146	0.14204	-1.98628
H	-3.45208	-0.06674	-2.67502
H	-4.95124	0.83280	-2.48723
H	-4.83054	-0.78155	-1.81619
C	-4.90626	0.90789	0.31926
H	-5.34652	-0.05537	0.58310
H	-5.70347	1.52319	-0.10098
H	-4.57814	1.39248	1.23868
Fe	-0.06032	-0.18084	2.49400
N	-0.85972	1.11391	-0.12236
Si	-0.15772	-0.06446	-1.22592
C	0.62536	-1.48025	1.13837
C	-0.56742	-1.99023	1.72190
H	-1.47949	-2.19441	1.17810
C	-0.34799	-2.12147	3.11530
H	-1.06924	-2.46815	3.84050
C	0.98505	-1.72614	3.39241
H	1.45617	-1.71879	4.36391
C	1.59066	-1.33403	2.17442
H	2.60151	-0.97506	2.04823
C	1.92166	-1.79356	-0.84293
C	1.85902	-3.16494	-1.14656
C	2.96090	-3.77412	-1.73214
H	2.92714	-4.83187	-1.96281
C	4.10277	-3.05438	-2.03479
H	4.94822	-3.54329	-2.50097
C	4.15684	-1.70891	-1.73266
H	5.05597	-1.15119	-1.96523
C	3.08634	-1.06031	-1.12207
C	0.64156	-4.00265	-0.83012
H	-0.16677	-3.32594	-0.55964
C	0.17012	-4.81304	-2.03800
H	0.89130	-5.57693	-2.33166
H	-0.00186	-4.17605	-2.90681
H	-0.76225	-5.33380	-1.80911
C	0.90819	-4.91313	0.36919
H	0.01378	-5.48223	0.63035
H	1.20231	-4.33680	1.24592
H	1.70473	-5.62825	0.15382
C	3.24849	0.40437	-0.77538
H	2.36666	0.72535	-0.21762
C	3.34486	1.26062	-2.03637
H	2.45176	1.15915	-2.65421
H	4.20294	0.97517	-2.64724
H	3.45991	2.31491	-1.78116
C	4.45467	0.64253	0.13400
H	4.49386	1.68737	0.44697
H	5.39710	0.41932	-0.36817
H	4.40978	0.02628	1.03226
N	0.80857	-1.14630	-0.21825
N	-2.39758	-2.54473	-1.33291
H	-3.33744	-2.27784	-1.58685
H	-2.42796	-3.51348	-1.04632
H	-1.84628	-2.50990	-2.17934

TSS15 (C_1): E_{tot} = -3035.78641554

C	1.61144	-1.23436	0.50975
C	1.14861	-2.53714	0.17285
H	0.68437	-2.82082	-0.76258
C	1.37413	-3.38689	1.28539
H	1.12248	-4.43543	1.34099
C	2.01216	-2.62687	2.29413
H	2.32947	-2.99137	3.25953
C	2.15458	-1.29888	1.82267
H	2.59828	-0.47622	2.36150
C	2.56595	0.76046	-0.46374
C	3.58937	0.32151	-1.32426
C	4.71087	1.12422	-1.49416

H	5.51334	0.79189	-2.14110
C	4.81879	2.34939	-0.86105
H	5.69765	2.96328	-1.00921
C	3.79567	2.78675	-0.04191
H	3.88766	3.74758	0.45005
C	2.66098	2.00970	0.17640
C	3.51218	-1.00464	-2.05002
H	2.48533	-1.36720	-1.99059
C	3.83852	-0.87244	-3.53701
H	4.87882	-0.59473	-3.71372
H	3.20670	-0.12524	-4.01789
H	3.67114	-1.82461	-4.04239
C	4.41526	-2.04087	-1.37937
H	4.32681	-3.00845	-1.87632
H	4.15216	-2.18038	-0.33112
H	5.46329	-1.73754	-1.42554
C	1.60345	2.52335	1.13182
H	0.74338	1.85745	1.06284
C	1.12677	3.93369	0.78685
H	0.73135	3.99759	-0.22686
H	1.92454	4.67225	0.87755
H	0.32893	4.23805	1.46594
C	2.10629	2.49347	2.57595
H	1.32659	2.82655	3.26397
H	2.96712	3.15073	2.71096
H	2.40782	1.49268	2.87952
Fe	0.17290	-1.79804	1.81274
N	1.43151	-0.06596	-0.24953
Si	-0.05965	0.04959	-1.19756
C	-1.39119	-0.67369	1.19839
C	-0.86731	-0.14487	2.41123
H	-0.44227	0.83965	2.53841
C	-0.95584	-1.14493	3.40985
H	-0.62795	-1.04764	4.43412
C	-1.56322	-2.28766	2.83342
H	-1.77571	-3.21732	3.34000
C	-1.82899	-2.00519	1.47496
H	-2.27443	-2.68525	0.76582
C	-2.69107	0.31341	-0.59879
C	-3.25046	1.55971	-0.25824
C	-4.48505	1.91266	-0.79019
H	-4.93550	2.85975	-0.52004
C	-5.15077	1.07591	-1.66695
H	-6.10794	1.36836	-2.07880
C	-4.58545	-0.13502	-2.01447
H	-5.11107	-0.78303	-2.70547
C	-3.36301	-0.54472	-1.48868
C	-2.56343	2.51012	0.69777
H	-1.52732	2.18457	0.80146
C	-2.53399	3.94601	0.17662
H	-3.53080	4.38054	0.09173
H	-2.06332	4.00800	-0.80595
H	-1.96493	4.58381	0.85403
C	-3.21753	2.44668	2.07877
H	-2.68928	3.08629	2.78898
H	-3.21224	1.43127	2.47375
H	-4.25523	2.78364	2.03762
C	-2.83764	-1.90462	-1.90013
H	-1.89889	-2.08632	-1.37416
C	-2.52695	-1.96056	-3.39533
H	-1.77733	-1.21626	-3.66086
H	-3.41803	-1.78547	-4.00090
H	-2.13347	-2.94193	-3.66674
C	-3.81555	-3.01640	-1.51224
H	-3.37368	-3.99632	-1.70100
H	-4.73918	-2.96165	-2.09065
H	-4.09284	-2.96616	-0.45897
N	-1.41653	-0.03497	-0.06134
N	0.09195	1.78471	-1.90901
H	-0.77833	2.26863	-2.09995
H	0.08037	0.68583	-2.70735
H	0.86579	2.43029	-1.76350

TSS16 (C_1): E_{tot} = -3035.81253805

C	1.56385	-0.94473	0.84412
C	1.26558	-2.33054	0.71807
H	0.94240	-2.83280	-0.18266
C	1.43924	-2.93376	1.98873
H	1.27715	-3.97571	2.22090
C	1.86875	-1.93342	2.89341
H	2.09158	-2.07598	3.94009
C	1.95119	-0.70762	2.19039
H	2.25717	0.24136	2.60197
C	2.64488	0.88625	-0.23473
C	3.83211	0.40544	-0.81573
C	4.93798	1.24504	-0.87092
H	5.85942	0.88576	-1.31228
C	4.88734	2.53154	-0.36911
H	5.75549	3.17491	-0.42657
C	3.72288	2.98412	0.21793
H	3.69304	3.98813	0.62329
C	2.59132	2.17860	0.31124
C	3.98604	-1.01002	-1.33853
H	3.01334	-1.49984	-1.35948
C	4.51558	-1.04445	-2.77152
H	5.53238	-0.65725	-2.84794
H	3.88787	-0.45528	-3.44080
H	4.53364	-2.07004	-3.14340
C	4.86655	-1.83188	-0.39627
H	4.94938	-2.86207	-0.74695
H	4.44881	-1.85382	0.61034
H	5.87537	-1.42006	-0.33297
C	1.38455	2.75048	1.02653
H	0.60507	1.98715	1.06036
C	0.81741	3.96253	0.29010
H	0.52268	3.69957	-0.72453
H	1.54423	4.77509	0.23948
H	-0.06383	4.34494	0.80798
C	1.70657	3.11700	2.47716
H	0.80311	3.44799	2.99245
H	2.43076	3.93049	2.53901
H	2.11537	2.27488	3.03472
Fe	0.04068	-1.42881	2.06957
N	1.50282	0.00973	-0.20785
Si	-0.18811	0.72942	-1.11372
C	-1.52672	-0.58320	1.13537
C	-1.23059	0.14275	2.32670
H	-0.93884	1.18161	2.37978
C	-1.34112	-0.73804	3.42936
H	-1.15847	-0.48505	4.46325
C	-1.74191	-2.00474	2.93902
H	-1.91434	-2.88989	3.53315
C	-1.85867	-1.91330	1.53302
H	-2.12650	-2.72169	0.87037
C	-2.80148	0.14816	-0.74678
C	-3.45882	1.37019	-0.50750
C	-4.72210	1.57418	-1.05204
H	-5.23864	2.50745	-0.86349
C	-5.33555	0.61148	-1.82994
H	-6.31905	0.78982	-2.24486
C	-4.68101	-0.57964	-2.07504
H	-5.16319	-1.33054	-2.68972
C	-3.42013	-0.83192	-1.54478
C	-2.86319	2.47756	0.33428
H	-1.83351	2.21240	0.57544
C	-2.80417	3.79837	-0.43100
H	-3.79587	4.16555	-0.69952
H	-2.22337	3.68951	-1.34677
H	-2.32922	4.57001	0.17683
C	-3.62485	2.62960	1.65123
H	-3.15472	3.38586	2.28319
H	-3.64704	1.69212	2.20664
H	-4.65744	2.94040	1.48135
C	-2.77496	-2.16329	-1.86131
H	-1.83002	-2.21037	-1.31687
C	-2.47718	-2.28921	-3.35712
H	-1.89682	-1.44278	-3.72473
H	-3.39751	-2.32438	-3.94197
H	-1.92305	-3.20598	-3.57099
C	-3.63278	-3.33973	-1.39284
H	-3.10142	-4.28347	-1.52999
H	-4.56415	-3.40870	-1.95683
H	-3.90000	-3.25019	-0.34003
N	-1.50386	-0.09939	-0.18812
N	0.43032	-0.70887	-2.25283
H	1.32297	-0.59751	-1.31499
H	0.67463	-0.44289	-3.19754
H	-0.07625	-1.58492	-2.27280

TSS17 (C_1): E_{tot} = -3035.78585865

C	1.19668	3.27831	2.57638
C	0.41409	2.95343	1.43162
H	0.65438	3.19437	0.40604
C	-0.72509	2.23589	1.87478
H	-1.50671	1.84267	1.24206
C	-0.66341	2.13702	3.28462
H	-1.38905	1.65354	3.92117
C	0.52031	2.78185	3.72127
H	0.85173	2.88753	4.74300
C	2.63258	4.92370	3.57390
C	3.46700	4.68651	4.67785
C	3.57984	5.67574	5.64998
H	4.21042	5.50312	6.51369
C	2.89719	6.87194	5.54640
H	2.99549	7.62491	6.31733
C	2.08978	7.10015	4.44744
H	1.55871	8.04073	4.36848
C	1.94614	6.14754	3.44698
C	4.22586	3.39472	4.88533
H	4.12661	2.78975	3.98423
C	5.71548	3.64814	5.11433
H	6.26578	2.70622	5.12876
H	5.90320	4.14103	6.06946
H	6.13473	4.27855	4.33185
C	3.64889	2.58571	6.04788
H	2.60198	2.33181	5.88475
H	3.71285	3.13522	6.98857
H	4.19962	1.65110	6.17707
C	1.03076	6.45955	2.27998
H	1.17038	5.70474	1.50493
C	1.35935	7.80423	1.63204
H	2.40608	7.85549	1.33206
H	1.16234	8.64539	2.29815
H	0.74766	7.95069	0.74063
C	-0.43579	6.39701	2.70932
H	-1.09437	6.58965	1.86073
H	-0.65396	7.14321	3.47585
H	-0.68943	5.41699	3.11291
Fe	1.00631	1.27288	2.42982
N	2.44902	3.93750	2.56541
Si	3.82868	3.28408	1.21375
C	2.85751	0.68110	1.86807
C	1.87632	0.02158	1.06882
H	1.78048	0.09636	-0.00354
C	1.03873	-0.72990	1.92283
H	0.19500	-1.32953	1.61497
C	1.48898	-0.54718	3.25427
H	1.05188	-0.98578	4.13904
C	2.61550	0.30865	3.22127
H	3.18173	0.64486	4.07716
C	4.92572	0.84963	0.67442
C	4.88522	0.78451	-0.72981
C	5.91429	0.12795	-1.39945
H	5.89975	0.08015	-2.48161
C	6.94995	-0.47510	-0.71459
H	7.73362	-0.99101	-1.25392
C	6.97754	-0.41375	0.66617
H	7.79099	-0.89110	1.19870

C	5.98773	0.25317	1.37755
C	3.78049	1.39747	-1.56778
H	3.00634	1.78470	-0.90254
C	4.29953	2.58158	-2.38362
H	5.06911	2.27223	-3.09313
H	4.72872	3.34815	-1.73800
H	3.48958	3.04287	-2.95114
C	3.11970	0.36267	-2.48048
H	2.25442	0.79774	-2.98335
H	2.78332	-0.51196	-1.92292
H	3.79938	0.00907	-3.25722
C	6.07879	0.28726	2.88720
H	5.34357	1.00768	3.24975
C	7.45134	0.75100	3.37493
H	7.72408	1.72197	2.95827
H	8.24279	0.04953	3.10823
H	7.45935	0.84019	4.46244
C	5.73093	-1.07799	3.48206
H	5.72417	-1.03637	4.57334
H	6.46165	-1.83281	3.18501
H	4.74978	-1.41715	3.15217
N	3.89422	1.52089	1.40770
H	2.61401	4.34452	1.28762
N	5.38235	3.81834	1.69045
H	5.77691	4.67010	1.33307
H	6.03144	3.28362	2.24434

TSS18 (C_1): E_{tot} = -3035.72935412

C	1.44833	-0.80191	1.13738
C	0.80429	-2.06596	1.13227
H	0.42855	-2.58472	0.26183
C	0.74295	-2.53707	2.46967
H	0.31420	-3.47519	2.78929
C	1.35339	-1.56795	3.29819
H	1.46964	-1.62744	4.37005
C	1.77316	-0.48863	2.48116
H	2.27430	0.40427	2.82036
C	3.08958	0.42976	-0.12842
C	4.01917	-0.41258	-0.76336
C	5.32802	0.02753	-0.90539
H	6.05855	-0.60051	-1.39779
C	5.72304	1.25895	-0.41148
H	6.74942	1.58377	-0.52172
C	4.80180	2.07112	0.21797
H	5.11721	3.03755	0.59154
C	3.47267	1.68108	0.36546
C	3.59955	-1.74974	-1.34030
H	2.77539	-2.14077	-0.73930
C	3.10636	-1.56959	-2.77917
H	3.91664	-1.22021	-3.42097
H	2.30324	-0.83403	-2.85724
H	2.73657	-2.51131	-3.18958
C	4.69461	-2.81133	-1.28479
H	4.28998	-3.77836	-1.58565
H	5.09726	-2.91656	-0.27766
H	5.52281	-2.59082	-1.95973
C	2.49741	2.65171	0.99375
H	1.54172	2.14065	1.12317
C	2.26270	3.83719	0.05699
H	1.92616	3.50858	-0.92577
H	3.17577	4.42027	-0.07971
H	1.49826	4.50280	0.46147
C	2.95535	3.14196	2.36731
H	2.18175	3.76066	2.82474
H	3.85833	3.75135	2.30509
H	3.16862	2.31827	3.04931
Fe	-0.25925	-0.76016	2.31844
N	1.72835	0.00227	0.01518
Si	-0.68971	1.83565	-0.71060
C	-1.75205	0.30663	1.34831
C	-1.21361	1.06630	2.41877
H	-0.75149	2.04026	2.33656
C	-1.40209	0.33076	3.61776
H	-1.10399	0.64889	4.60569
C	-2.06270	-0.87646	3.29100
H	-2.35326	-1.65147	3.98451
C	-2.26538	-0.90233	1.88821
H	-2.74914	-1.68723	1.32537
C	-2.91691	0.22969	-0.75900
C	-4.15540	0.87183	-0.59176
C	-5.23736	0.43034	-1.34243
H	-6.20290	0.90585	-1.21971
C	-5.10257	-0.60048	-2.25663
H	-5.95440	-0.92347	-2.84070
C	-3.87565	-1.21360	-2.42127
H	-3.77738	-2.01794	-3.14047
C	-2.76852	-0.82178	-1.67308
C	-4.34416	2.01446	0.38238
H	-3.36541	2.31065	0.76093
C	-4.94811	3.24401	-0.29488
H	-5.96478	3.06475	-0.64811
H	-4.34973	3.55857	-1.15060
H	-4.99282	4.07834	0.40664
C	-5.17767	1.57208	1.58519
H	-5.28283	2.38889	2.30104
H	-4.70802	0.73494	2.10170
H	-6.18066	1.26349	1.28402
C	-1.45811	-1.55210	-1.86664
H	-0.75239	-1.15666	-1.13408
C	-0.87756	-1.31353	-3.26019
H	-0.68538	-0.25493	-3.43651
H	-1.55374	-1.66370	-4.04182
H	0.06642	-1.84792	-3.38343
C	-1.60509	-3.04732	-1.58536
H	-0.64170	-3.55310	-1.67873
H	-2.28836	-3.52995	-2.28555
H	-1.98186	-3.22703	-0.57797
N	-1.80100	0.68476	-0.00029
N	-0.99243	2.26542	-2.28133
H	1.39517	-0.43946	-0.82952
H	-1.00425	3.32568	-1.03268
H	-1.73614	1.91707	-2.88977

TSS19 (C_1): E_{tot} = -3035.81378984

C	1.52921	-0.85929	1.05195
C	1.24209	-2.25040	1.06492
H	0.96621	-2.83391	0.19874
C	1.34516	-2.69005	2.40617
H	1.17032	-3.69715	2.75404
C	1.71459	-1.58351	3.20947
H	1.87410	-1.59698	4.27712
C	1.83826	-0.44854	2.37238
H	2.12749	0.54341	2.68162
C	2.62257	0.91117	-0.16007
C	3.88008	0.44711	-0.58230
C	4.93834	1.34588	-0.60162
H	5.91197	1.01593	-0.93944
C	4.77503	2.65676	-0.19323
H	5.61356	3.34042	-0.20913
C	3.53620	3.08738	0.23349
H	3.41602	4.11526	0.55217
C	2.43643	2.23246	0.25761
C	4.12411	-0.97209	-1.06076
H	3.31444	-1.61936	-0.72146
C	4.13171	-1.01757	-2.58965
H	4.92585	-0.39007	-2.99836
H	3.17951	-0.67568	-2.99444
H	4.29583	-2.03763	-2.94073
C	5.40126	-1.57430	-0.47715
H	5.47047	-2.62768	-0.75061
H	5.40981	-1.51023	0.61091
H	6.30331	-1.08862	-0.85169
C	1.11159	2.80680	0.71073
H	0.35081	2.02576	0.67083
C	0.67128	3.93437	-0.22219
H	0.57954	3.58799	-1.25061
H	1.38069	4.76297	-0.21062
H	-0.29558	4.32999	0.08578
C	1.15782	3.30721	2.15492
H	0.17798	3.68508	2.45359
H	1.87092	4.12313	2.28016
H	1.43228	2.51869	2.85456
Fe	-0.06303	-1.20050	2.23046
N	1.53984	-0.04991	-0.13426
Si	-0.10260	0.12171	-1.16414
C	-1.60437	-0.41948	1.18869
C	-1.37258	0.35414	2.36353
H	-1.10694	1.40080	2.38861
C	-1.52548	-0.48945	3.48912
H	-1.39966	-0.19877	4.52158
C	-1.87486	-1.78165	3.02394
H	-2.05585	-2.64981	3.64027
C	-1.92598	-1.74208	1.61197
H	-2.13864	-2.57682	0.96251
C	-2.79283	0.26071	-0.79272
C	-3.46003	1.49024	-0.63715
C	-4.69769	1.66820	-1.24415
H	-5.22642	2.60441	-1.11569
C	-5.27044	0.67384	-2.01358
H	-6.23288	0.83232	-2.48245
C	-4.60271	-0.52212	-2.17994
H	-5.05350	-1.29800	-2.78677
C	-3.37011	-0.75669	-1.57659
C	-2.90355	2.62390	0.19400
H	-1.86303	2.39004	0.42410
C	-2.91542	3.95028	-0.56529
H	-3.92735	4.27759	-0.80703
H	-2.35537	3.88090	-1.49772
H	-2.46632	4.74018	0.03911
C	-3.66973	2.75261	1.51182
H	-3.22071	3.51740	2.14939
H	-3.67782	1.81411	2.06498
H	-4.70769	3.04095	1.33462
C	-2.73397	-2.11317	-1.80091
H	-1.78161	-2.15187	-1.27184
C	-2.42566	-2.34027	-3.28178
H	-1.81931	-1.53299	-3.69166
H	-3.33588	-2.40196	-3.88064
H	-1.87939	-3.27507	-3.41917
C	-3.62430	-3.23898	-1.26945
H	-3.10713	-4.19805	-1.33456
H	-4.54765	-3.32966	-1.84409
H	-3.90896	-3.07668	-0.22971
N	-1.53240	0.04741	-0.14036
N	0.54829	-1.14224	-2.00368
H	1.60339	-0.77177	-1.02331
H	-0.14242	1.49441	-1.73033
H	0.25092	-1.60151	-2.84523

TSS26 (C_1): E_{tot} = -3005.98119875

C	1.43016	-0.72275	-1.18326
C	1.03173	-0.12705	-2.41314
H	0.69348	0.89122	-2.54078
C	1.11251	-1.11021	-3.42802
H	0.85989	-0.96892	-4.46829
C	1.59409	-2.30706	-2.84244
H	1.76956	-3.23939	-3.35805
C	1.78995	-2.07468	-1.46238
H	2.13219	-2.80072	-0.74180
C	2.70871	0.20350	0.65757
C	3.32308	1.42684	0.34165
C	4.57330	1.70509	0.88031
H	5.06795	2.63640	0.63344
C	5.19744	0.81632	1.73597
H	6.16962	1.05097	2.14948
C	4.56904	-0.36779	2.06805
H	5.05801	-1.05180	2.75112
C	3.32442	-0.69827	1.54045
C	2.67790	2.44668	-0.57015

H	1.64041	2.14933	-0.73278
C	2.64724	3.83787	0.06118
H	3.64744	4.25043	0.20059
H	2.15643	3.82066	1.03472
H	2.10018	4.53316	-0.57708
C	3.37296	2.47559	-1.93170
H	2.87182	3.17104	-2.60806
H	3.37308	1.49127	-2.39947
H	4.41144	2.79852	-1.83694
C	2.69966	-2.01403	1.95402
H	1.74743	-2.12194	1.43115
C	2.40312	-2.04248	3.45417
H	1.76483	-1.21099	3.75026
H	3.31904	-1.98600	4.04481
H	1.89465	-2.96894	3.72683
C	3.58570	-3.20060	1.56840
H	3.07051	-4.14314	1.76171
H	4.51124	-3.21412	2.14611
H	3.86620	-3.17599	0.51535
Fe	-0.15958	-1.70797	-1.91808
N	1.43344	-0.10457	0.08663
Si	0.03307	0.33657	1.01604
C	-1.61615	-1.10050	-0.66978
C	-1.26743	-2.44569	-0.37241
H	-0.86885	-2.80157	0.56802
C	-1.49901	-3.22353	-1.53411
H	-1.32009	-4.28392	-1.63155
C	-2.02249	-2.37121	-2.53707
H	-2.30993	-2.66778	-3.53455
C	-2.09246	-1.05970	-2.00960
H	-2.44133	-0.17834	-2.52705
C	-2.59774	0.81843	0.41665
C	-3.57407	0.35931	1.31301
C	-4.70601	1.13954	1.51764
H	-5.47701	0.79627	2.19641
C	-4.86121	2.35656	0.87921
H	-5.74522	2.95479	1.05754
C	-3.88017	2.80800	0.01648
H	-4.00609	3.76592	-0.47375
C	-2.74189	2.05017	-0.23757
C	-3.43783	-0.95773	2.04510
H	-2.41533	-1.31491	1.91771
C	-3.66157	-0.80587	3.54896
H	-4.68734	-0.52294	3.79000
H	-2.99882	-0.05128	3.97320
H	-3.46148	-1.75067	4.05659
C	-4.37439	-2.00810	1.44726
H	-4.24538	-2.97006	1.94652
H	-4.18006	-2.15457	0.38479
H	-5.41984	-1.71403	1.55934
C	-1.70090	2.58995	-1.19306
H	-0.94775	1.81464	-1.34347
C	-0.99850	3.81361	-0.60567
H	-0.53523	3.58498	0.35492
H	-1.69701	4.63689	-0.44666
H	-0.21459	4.17010	-1.27649
C	-2.29172	2.91336	-2.56505
H	-1.50427	3.21075	-3.25969
H	-3.00837	3.73462	-2.51810
H	-2.80860	2.05437	-2.99372
N	-1.44323	0.01656	0.16800
B	0.07317	1.60404	2.45517
H	-0.95260	1.81967	3.02329
H	1.10844	1.86860	2.98167
H	0.06770	-0.42445	2.38650

TSS20 (C_1): E_{tot} = -3062.36583546

C	1.64453	-1.21379	-0.31170
C	1.29762	-2.47839	0.23935
H	0.84437	-2.64913	1.20643
C	1.63043	-3.47556	-0.71152
H	1.47951	-4.53787	-0.59145
C	2.22041	-2.83704	-1.82820
H	2.59444	-3.32622	-2.71488
C	2.22650	-1.44234	-1.58814
H	2.61294	-0.68103	-2.24821
C	-1.36028	-1.05334	-1.13646
C	-1.68017	-2.44439	-1.15777
H	-2.08149	-3.01605	-0.33548
C	-1.35739	-2.95104	-2.43622
H	-1.47536	-3.97452	-2.75933
C	-0.83749	-1.88740	-3.21457
H	-0.49278	-1.95708	-4.23545
C	-0.85777	-0.71348	-2.42412
H	-0.51301	0.26360	-2.73052
C	2.49450	0.95022	0.27628
C	3.55319	0.69412	1.15980
C	4.67970	1.50311	1.09443
H	5.51090	1.31500	1.76248
C	4.75571	2.55635	0.20174
H	5.64076	3.17798	0.16750
C	3.68933	2.82079	-0.63471
H	3.74938	3.65923	-1.31813
C	2.54478	2.03003	-0.61448
C	3.51099	-0.42547	2.17637
H	2.49688	-0.82549	2.20591
C	4.45214	-1.56281	1.77758
H	4.21014	-1.95830	0.79091
H	4.38718	-2.38469	2.49277
H	5.49031	-1.22536	1.75687
C	3.81600	0.08200	3.58535
H	4.84424	0.43370	3.68435
H	3.68058	-0.72231	4.31037
H	3.14825	0.89895	3.85609
C	1.40431	2.39548	-1.53981
H	0.56095	1.73664	-1.32070
C	0.95207	3.84046	-1.31499
H	0.79090	4.06510	-0.25875
H	0.02926	4.04741	-1.85858
H	1.69876	4.55271	-1.66819
C	1.76011	2.18115	-3.01055
H	2.61219	2.79442	-3.30752
H	0.92076	2.45069	-3.65513
H	2.01441	1.14200	-3.21647
C	-2.79023	0.18434	0.39109
C	-3.44992	-0.52298	1.41230
C	-4.71516	-0.09705	1.80876
H	-5.23136	-0.62728	2.59961
C	-5.33256	0.98136	1.20835
H	-6.31958	1.29038	1.52631
C	-4.68183	1.66423	0.19721
H	-5.17559	2.50555	-0.27241
C	-3.40933	1.29264	-0.21904
C	-2.87126	-1.74273	2.09962
H	-1.89800	-1.96228	1.65856
C	-3.76055	-2.96965	1.88369
H	-4.72211	-2.86434	2.38808
H	-3.28084	-3.86388	2.28497
H	-3.96798	-3.14023	0.82705
C	-2.65490	-1.49873	3.59381
H	-2.01648	-0.63450	3.77616
H	-2.18058	-2.36463	4.05825
H	-3.59905	-1.32670	4.11306
C	-2.75389	2.06951	-1.33977
H	-1.69735	1.79703	-1.36680
C	-3.36567	1.68018	-2.68658
H	-3.29530	0.60716	-2.86079
H	-2.85430	2.18879	-3.50614
H	-4.42050	1.95744	-2.73127
C	-2.82919	3.58148	-1.12990
H	-3.85305	3.95480	-1.16726
H	-2.27584	4.10024	-1.91361
H	-2.40734	3.88421	-0.17021
H	-0.31391	2.61492	0.73052
H	-1.35434	2.06854	1.84629
Fe	0.30109	-2.11986	-1.51034
N	1.38255	0.05171	0.25480
N	-1.47139	-0.18818	-0.02200
H	-0.03496	3.30984	3.36109
H	1.59754	2.91761	2.24539
H	0.82182	1.49366	3.43323
Si	-0.14828	0.24685	1.03453
H	-0.24345	-0.48386	2.31298
B	0.59836	2.47586	2.75579
N	-0.39960	1.96720	1.50839

TSS21 (C_1): E_{tot} = -3062.39957172

C	1.61151	-0.85278	1.10966
C	1.16033	-2.17883	1.34240
H	0.69610	-2.82149	0.60698
C	1.38065	-2.48361	2.70976
H	1.13253	-3.41431	3.19814
C	1.99385	-1.36112	3.31822
H	2.29503	-1.28472	4.35225
C	2.13205	-0.35069	2.33598
H	2.55863	0.63086	2.48135
C	2.67998	0.51726	-0.54659
C	3.69677	-0.27143	-1.11252
C	4.86779	0.34795	-1.53272
H	5.66541	-0.24883	-1.95789
C	5.03158	1.71783	-1.43055
H	5.94735	2.18397	-1.77045
C	4.01778	2.48671	-0.89166
H	4.15105	3.55947	-0.81360
C	2.83917	1.90701	-0.43252
C	3.55962	-1.77234	-1.25077
H	2.50840	-2.02917	-1.11224
C	3.95213	-2.26915	-2.64139
H	5.01618	-2.13868	-2.84567
H	3.39359	-1.75035	-3.42033
H	3.73740	-3.33505	-2.73198
C	4.37021	-2.48973	-0.16983
H	4.22829	-3.57014	-0.23368
H	4.07377	-2.17379	0.82983
H	5.43748	-2.28745	-0.28290
C	1.78117	2.80058	0.17455
H	0.96904	2.16433	0.52862
C	1.20303	3.75769	-0.86569
H	0.76892	3.20519	-1.69992
H	1.96676	4.42754	-1.26485
H	0.41764	4.37761	-0.42903
C	2.30922	3.56933	1.38561
H	1.50825	4.14461	1.85379
H	3.09717	4.27253	1.11124
H	2.71969	2.89672	2.13883
Fe	0.15608	-0.84244	2.51194
N	1.49263	-0.11318	-0.07769
Si	0.01760	0.00183	-1.02753
C	-1.42177	-0.17646	1.44991
C	-0.95586	0.86041	2.30958
H	-0.57378	1.81852	1.98769
C	-1.03349	0.40693	3.64800
H	-0.73781	0.96377	4.52466
C	-1.58221	-0.89833	3.63462
H	-1.77384	-1.51548	4.49982
C	-1.81788	-1.26381	2.28911
H	-2.20331	-2.21527	1.96176
C	-2.62259	-0.04505	-0.68945
C	-3.10859	1.22627	-1.06253
C	-4.23586	1.30720	-1.87293
H	-4.62559	2.27657	-2.15716
C	-4.88462	0.16661	-2.31472
H	-5.76152	0.24843	-2.94405
C	-4.41425	-1.07433	-1.93413
H	-4.93416	-1.96266	-2.27179
C	-3.28854	-1.20565	-1.12413
C	-2.47257	2.50173	-0.55540
H	-1.44892	2.27267	-0.25281

C	-2.38722	3.59526	-1.61704
H	-3.36827	3.96311	-1.92078
H	-1.86814	3.24612	-2.51132
H	-1.83350	4.45189	-1.23222
C	-3.22656	3.00440	0.67769
H	-2.74314	3.89063	1.09396
H	-3.26395	2.24542	1.45826
H	-4.25386	3.27339	0.42392
C	-2.87346	-2.59723	-0.69803
H	-1.92054	-2.51528	-0.17208
C	-2.66090	-3.55206	-1.87157
H	-1.92179	-3.16940	-2.57197
H	-3.58503	-3.73582	-2.42266
H	-2.30835	-4.51858	-1.50767
C	-3.92003	-3.17991	0.25762
H	-3.57322	-4.12313	0.68467
H	-4.85545	-3.38466	-0.26668
H	-4.15313	-2.49928	1.07591
N	-1.40351	-0.14454	0.04142
B	-0.03915	-0.86246	-2.98283
H	-0.01388	-1.51749	-1.64028
H	1.12782	-0.93659	-3.24916
N	-0.67632	0.41606	-3.86463
H	-0.71385	-1.77733	-3.38543
H	-0.70119	0.23105	-4.86756
H	-0.12914	1.24988	-3.66762
H	-1.62485	0.59901	-3.54699

TSS22 (C_1): E_{tot} = -3062.40989527

C	4.43601	17.88319	1.96556
C	3.56724	18.72950	1.21928
H	2.50028	18.82446	1.36140
C	4.35159	19.45008	0.28545
H	3.98306	20.18040	-0.41941
C	5.69602	19.03004	0.42418
H	6.53581	19.38701	-0.15297
C	5.75310	18.06834	1.46152
H	6.64179	17.57054	1.81637
C	4.48911	20.17444	4.13739
C	4.03962	21.24427	3.30452
H	3.01602	21.55608	3.16702
C	5.16520	21.81749	2.67190
H	5.14761	22.64223	1.97498
C	6.31821	21.11368	3.09993
H	7.33376	21.30962	2.78950
C	5.90600	20.11252	4.01111
H	6.54937	19.40088	4.50748
C	4.37475	15.71482	3.07034
C	3.50461	14.83131	2.40652
C	3.78719	13.47112	2.43164
H	3.12901	12.77753	1.92382
C	4.89557	12.98172	3.10035
H	5.09499	11.91801	3.11598
C	5.75206	13.85858	3.73717
H	6.62719	13.47023	4.24464
C	5.51841	15.23146	3.72463
C	2.30603	15.32721	1.62687
H	2.07805	16.34088	1.95905
C	2.64275	15.39218	0.13535
H	3.50887	16.02623	-0.05256
H	1.80309	15.79681	-0.43264
H	2.86361	14.39813	-0.25868
C	1.04907	14.49298	1.86427
H	1.13648	13.48324	1.45998
H	0.19446	14.95902	1.37200
H	0.81820	14.41263	2.92651
C	6.52684	16.14202	4.39055
H	6.19382	17.17109	4.24455
C	6.61749	15.88971	5.89379
H	5.65029	16.01237	6.37900
H	7.31839	16.58633	6.35793
H	6.97234	14.87991	6.10967
C	7.91056	15.99864	3.75330
H	8.34657	15.01903	3.95634
H	8.59587	16.74817	4.15295
H	7.87846	16.11521	2.67015
C	3.17260	19.83478	6.14700
C	1.91507	20.46276	6.18957
C	1.44359	20.94095	7.40859
H	0.47237	21.41882	7.45339
C	2.19092	20.82692	8.56338
H	1.81101	21.21355	9.50008
C	3.42920	20.21534	8.51291
H	4.01318	20.13411	9.42117
C	3.93379	19.70334	7.32307
C	1.03521	20.66115	4.97228
H	1.55544	20.26981	4.09870
C	0.76380	22.14544	4.71962
H	0.15604	22.58675	5.51099
H	0.22108	22.28056	3.78218
H	1.68878	22.71989	4.66283
C	-0.27613	19.88509	5.09425
H	-0.09697	18.82044	5.24215
H	-0.87500	20.00053	4.18913
H	-0.87751	20.23963	5.93316
C	5.30152	19.05826	7.33616
H	5.42578	18.51819	6.39556
C	6.39512	20.12496	7.42158
H	6.30343	20.85911	6.62208
H	7.38649	19.67175	7.35283
H	6.34309	20.66185	8.37082
C	5.45896	18.04389	8.46784
H	5.40783	18.51028	9.45244
H	6.42724	17.54672	8.40026
H	4.68880	17.27421	8.42299
Fe	4.99661	19.80917	2.20971
N	4.07985	17.11015	3.08696
N	3.68505	19.30971	4.91803
Si	3.00267	17.81940	4.27505
H	1.70926	18.12679	3.59495
B	2.05377	16.39879	5.60491
N	3.08784	15.67156	6.53733
H	1.57836	15.73462	4.72644
H	1.32236	17.14803	6.18627
H	3.37359	14.74713	6.26136
H	2.96288	15.78403	7.52849
H	3.37872	16.67183	5.67430

TSS23 (C_1): E_{tot} = -3062.36498066

C	1.76295	3.71870	1.35222
C	1.87662	3.13243	0.05940
H	2.79533	2.98659	-0.49253
C	0.58074	2.74455	-0.37085
H	0.34415	2.26670	-1.30991
C	-0.34239	3.11964	0.63240
H	-1.41180	2.97458	0.60012
C	0.38110	3.71456	1.69518
H	-0.03926	4.10680	2.60839
C	2.65095	5.37550	2.82271
C	2.39302	5.47486	4.20096
C	2.16732	6.73252	4.75067
H	1.95464	6.82122	5.80916
C	2.19815	7.87506	3.97320
H	2.00975	8.84316	4.41911
C	2.47890	7.77157	2.62403
H	2.51247	8.67019	2.01970
C	2.72115	6.53720	2.03158
C	2.32504	4.26733	5.11050
H	2.73006	3.41781	4.56018
C	3.16745	4.44372	6.37399
H	3.22633	3.50092	6.92111
H	2.73559	5.18198	7.05222
H	4.18270	4.75389	6.13656
C	0.88179	3.93772	5.49531
H	0.26995	3.70882	4.62408
H	0.41305	4.77227	6.02008
H	0.84645	3.07139	6.15994
C	3.02906	6.49163	0.54909
H	3.35602	5.48371	0.29189
C	4.18157	7.42406	0.17701
H	5.07210	7.20326	0.76611
H	3.93310	8.47563	0.32958
H	4.43883	7.30292	-0.87656
C	1.78112	6.79883	-0.27929
H	1.99886	6.72682	-1.34646
H	1.41412	7.80839	-0.08287
H	0.97565	6.09957	-0.05592
Fe	1.07623	1.81408	1.39609
N	2.80931	4.10357	2.20137
Si	4.42760	3.35034	1.91758
C	2.61566	0.87629	2.31288
C	2.12828	0.07140	1.23955
H	2.69268	-0.24335	0.37586
C	0.77116	-0.23419	1.49728
H	0.12588	-0.82546	0.86490
C	0.40655	0.38034	2.71974
H	-0.56468	0.33843	3.18957
C	1.54620	1.04940	3.23152
H	1.58777	1.62369	4.14473
C	5.01421	0.53008	2.36822
C	5.77025	0.34811	1.19579
C	6.94920	-0.39193	1.27043
H	7.54640	-0.53046	0.37729
C	7.36451	-0.97099	2.45345
H	8.28378	-1.54130	2.48823
C	6.57871	-0.84635	3.58737
H	6.88778	-1.33977	4.49998
C	5.39994	-0.10801	3.56817
C	5.32913	0.83798	-0.16809
H	4.40353	1.40333	-0.04792
C	6.35150	1.76212	-0.82627
H	7.30446	1.25832	-0.99969
H	6.52382	2.64634	-0.21435
H	5.98458	2.10270	-1.79585
C	5.03504	-0.35534	-1.08341
H	4.58489	-0.01584	-2.01759
H	4.35510	-1.07065	-0.62082
H	5.94621	-0.89895	-1.33876
C	4.52722	-0.07182	4.80573
H	3.87403	0.79846	4.73978
C	5.31212	0.06691	6.10754
H	5.96560	0.93995	6.09309
H	5.92027	-0.81167	6.32839
H	4.62215	0.19138	6.94285
C	3.63946	-1.31871	4.83929
H	2.96390	-1.28560	5.69575
H	4.24301	-2.22474	4.92338
H	3.03077	-1.40121	3.93959
N	3.91022	1.43319	2.41008
B	5.37036	3.40280	3.84864
H	5.36094	4.60093	3.99026
H	5.12306	2.86874	4.90600
H	4.33120	2.32005	3.33080
N	6.89714	2.95605	3.47689
H	7.00025	1.94304	3.49607
H	7.56698	3.35272	4.12745
H	7.13790	3.26294	2.53566

TSS24 (C_1): E_{tot} = -3062.38752135

C	1.60627	3.16498	1.92788
C	1.44808	2.41458	0.72581
H	2.18091	2.31389	-0.06125
C	0.17851	1.78955	0.75724
H	-0.22559	1.13803	-0.00312
C	-0.47124	2.17755	1.95488
H	-1.45597	1.86752	2.27144
C	0.39910	3.02957	2.67290
H	0.19861	3.47218	3.63639
C	2.55221	5.32156	2.29129

C	2.22810	6.00121	3.47740
C	2.01453	7.37547	3.42973
H	1.76897	7.90953	4.33978
C	2.10128	8.07384	2.24222
H	1.92281	9.14104	2.22159
C	2.42031	7.39820	1.07995
H	2.48780	7.95135	0.15136
C	2.65888	6.02864	1.08050
C	2.10115	5.31287	4.81963
H	2.22088	4.23861	4.67080
C	3.20508	5.77391	5.77105
H	3.13475	5.24254	6.72220
H	3.12709	6.84176	5.98279
H	4.19283	5.58874	5.34992
C	0.72785	5.54530	5.45283
H	-0.08374	5.28826	4.77207
H	0.58541	6.58767	5.74216
H	0.61638	4.94130	6.35496
C	2.99885	5.36331	-0.23458
H	3.32355	4.34294	-0.02820
C	4.15667	6.06257	-0.94578
H	5.04234	6.10527	-0.31212
H	3.90477	7.08232	-1.23988
H	4.42103	5.52597	-1.85862
C	1.76699	5.28981	-1.13802
H	1.99554	4.75288	-2.06095
H	1.42225	6.28802	-1.41485
H	0.94156	4.77667	-0.64571
Fe	1.32519	1.24600	2.38181
N	2.75089	3.89796	2.31475
Si	4.42614	3.39143	2.61833
C	3.15080	0.77589	3.10315
C	2.71752	-0.23297	2.19565
H	3.23168	-0.53066	1.29385
C	1.51154	-0.78624	2.68954
H	0.93910	-1.57252	2.22080
C	1.19086	-0.12469	3.89888
H	0.32691	-0.31364	4.51865
C	2.20379	0.83223	4.16257
H	2.23671	1.50362	5.00628
C	5.41918	0.77197	2.38650
C	5.86985	1.01049	1.07427
C	6.98224	0.31451	0.60375
H	7.34585	0.51329	-0.39674
C	7.61571	-0.63946	1.36841
H	8.48034	-1.16604	0.98644
C	7.10378	-0.93936	2.61567
H	7.56625	-1.72584	3.19923
C	6.00871	-0.26559	3.14067
C	5.18337	1.90949	0.06360
H	4.32589	2.40353	0.53043
C	6.10615	3.00229	-0.46826
H	6.96162	2.58270	-0.99886
H	6.48176	3.62639	0.34169
H	5.57344	3.64430	-1.17002
C	4.59386	1.08626	-1.08444
H	4.05409	1.73375	-1.77872
H	3.89667	0.33221	-0.71913
H	5.36919	0.57193	-1.65351
C	5.48663	-0.76177	4.47535
H	4.65063	-0.14703	4.78811
C	6.54067	-0.67023	5.57749
H	6.91186	0.34877	5.67870
H	7.39570	-1.32142	5.38782
H	6.11103	-0.97012	6.53497
C	4.95098	-2.18885	4.34206
H	4.52099	-2.52040	5.28879
H	5.73449	-2.89834	4.06968
H	4.16846	-2.24482	3.58554
N	4.33984	1.54810	2.95782
B	4.92805	2.37074	4.83114
H	4.30247	3.39864	4.60554
H	4.38213	1.60985	5.55933
H	6.08878	2.34600	4.57026
N	5.69148	3.53625	6.82988
H	5.00244	3.85188	7.49654
H	6.25183	4.33205	6.56150
H	6.29619	2.87446	7.29424

TSS25 (C_1): E_{tot} = -3062.41379694

C	1.36463	1.51929	0.39800
C	1.03806	1.89265	1.73069
H	0.81263	1.20775	2.53638
C	1.01769	3.30773	1.79657
H	0.78670	3.89812	2.67072
C	1.36321	3.81272	0.51826
H	1.44018	4.85514	0.24787
C	1.58132	2.71381	-0.34589
H	1.85414	2.76605	-1.38946
C	2.69548	-0.15920	-0.66656
C	3.75817	-0.48947	0.18980
C	4.98491	-0.82227	-0.37038
H	5.81815	-1.07132	0.27503
C	5.16172	-0.85470	-1.74205
H	6.12198	-1.12706	-2.16038
C	4.10639	-0.53879	-2.57481
H	4.25192	-0.56944	-3.64778
C	2.86586	-0.17420	-2.05944
C	3.61327	-0.46945	1.69504
H	2.54950	-0.43240	1.93028
C	4.15856	-1.73956	2.34611
H	5.24331	-1.81999	2.25886
H	3.71533	-2.62586	1.89302
H	3.92406	-1.74226	3.41263
C	4.27975	0.77505	2.28369
H	4.12777	0.82189	3.36383
H	3.87547	1.68925	1.85015
H	5.35623	0.76574	2.10074
C	1.75765	0.18214	-3.02837
H	0.92079	0.58821	-2.45652
C	1.25782	-1.05847	-3.76805
H	0.90806	-1.82191	-3.07185
H	2.04886	-1.50367	-4.37414
H	0.43132	-0.80609	-4.43548
C	2.18350	1.26679	-4.01813
H	1.33481	1.57607	-4.63039
H	2.96168	0.91867	-4.69877
H	2.56860	2.15045	-3.50825
Fe	-0.31632	2.60398	0.39557
N	1.42616	0.20731	-0.11250
Si	0.18299	-1.02879	-0.16838
C	-1.65542	1.16782	0.02342
C	-1.52682	1.99979	-1.12605
H	-1.18706	1.68083	-2.10103
C	-1.87069	3.32263	-0.75707
H	-1.85508	4.18557	-1.40589
C	-2.24868	3.31240	0.60782
H	-2.56764	4.16774	1.18450
C	-2.12106	1.98964	1.09131
H	-2.30935	1.66906	2.10328
C	-2.50212	-1.07241	0.10709
C	-3.07412	-1.49097	-1.10577
C	-4.20912	-2.29383	-1.06775
H	-4.66569	-2.61644	-1.99555
C	-4.76608	-2.69309	0.13142
H	-5.64992	-3.31755	0.14101
C	-4.18105	-2.29623	1.31811
H	-4.61441	-2.62311	2.25570
C	-3.04950	-1.48779	1.33220
C	-2.51733	-1.11285	-2.46157
H	-1.57921	-0.57450	-2.31799
C	-2.18636	-2.35060	-3.29461
H	-3.07631	-2.93441	-3.53401
H	-1.49088	-3.00337	-2.76663
H	-1.72449	-2.06160	-4.23984
C	-3.47027	-0.17807	-3.20650
H	-3.03691	0.13559	-4.15826
H	-3.68601	0.71629	-2.62196
H	-4.42034	-0.66889	-3.42512
C	-2.46983	-1.10304	2.67692
H	-1.59432	-0.47382	2.50590
C	-1.99941	-2.33397	3.45337
H	-1.28542	-2.91103	2.86963
H	-2.83517	-2.98703	3.71163
H	-1.51808	-2.03378	4.38652
C	-3.47850	-0.31176	3.51357
H	-3.00990	0.06989	4.42267
H	-4.31732	-0.93830	3.82149
H	-3.89599	0.53229	2.96505
N	-1.34974	-0.21302	0.09007
B	0.73961	-2.96676	1.05112
N	1.32016	-4.33546	2.75845
H	2.15688	-3.99650	3.21047
H	1.51246	-5.24660	2.36894
H	0.59421	-4.43097	3.45370
H	-0.23665	-3.61470	0.82000
H	0.67032	-2.04893	1.85319
H	1.78192	-3.17271	0.50352

1, triplet, (C_1): E_{tot} = -2979.39429474

C	4.71674	17.79127	1.89223
C	3.78349	18.62457	1.24324
H	2.75968	18.78437	1.54917
C	4.47476	19.32872	0.23510
H	4.04198	20.06874	-0.42186
C	5.79890	18.82186	0.14724
H	6.53730	19.10535	-0.58714
C	5.96424	17.88452	1.18535
H	6.84215	17.28801	1.38564
C	5.06111	20.01258	4.15622
C	4.71119	21.20726	3.49731
H	3.71841	21.62029	3.40219
C	5.90380	21.77417	2.96392
H	5.96078	22.70170	2.41381
C	6.99810	20.98814	3.36701
H	8.03486	21.16701	3.12604
C	6.48479	19.86319	4.04350
H	7.06419	19.06601	4.48730
C	4.71903	15.58561	2.83398
C	3.75974	14.79701	2.18116
C	4.02060	13.44263	2.01095
H	3.29608	12.81827	1.50266
C	5.18574	12.87201	2.49036
H	5.36710	11.81362	2.35579
C	6.11576	13.65681	3.14564
H	7.02174	13.19886	3.52302
C	5.90815	15.02117	3.32086
C	2.46473	15.37318	1.65341
H	2.35975	16.38662	2.04138
C	2.49611	15.46259	0.12751
H	3.33858	16.06263	-0.21620
H	1.58140	15.92193	-0.25143
H	2.58176	14.47257	-0.32470
C	1.24646	14.58993	2.14067
H	1.22088	13.57372	1.74431
H	0.32799	15.08338	1.81898
H	1.22824	14.52408	3.22867
C	6.95942	15.84048	4.04073
H	6.72676	16.89541	3.88711
C	6.92929	15.57145	5.54611
H	5.94391	15.77119	5.97101
H	7.65394	16.19914	6.06905
H	7.17158	14.53015	5.76579
C	8.36241	15.61269	3.47916
H	8.72459	14.60164	3.67005
H	9.07149	16.30028	3.94281
H	8.39621	15.77422	2.40112
C	3.49816	19.69722	5.93538

C	2.18201	20.15760	5.78265
C	1.53844	20.70443	6.88816
H	0.51873	21.05733	6.79520
C	2.17928	20.80912	8.10831
H	1.66558	21.24403	8.95572
C	3.47656	20.34954	8.24433
H	3.96811	20.42813	9.20640
C	4.15356	19.77955	7.17281
C	1.43162	20.05478	4.47073
H	2.14763	19.79523	3.68886
C	0.78668	21.37916	4.06339
H	-0.01880	21.67051	4.73885
H	0.35422	21.29868	3.06490
H	1.51190	22.19365	4.05091
C	0.38794	18.93827	4.52846
H	0.84423	17.97657	4.76927
H	-0.12792	18.83698	3.57156
H	-0.36496	19.14074	5.29216
C	5.56371	19.27234	7.37766
H	5.86570	18.73135	6.48061
C	6.53820	20.43485	7.56520
H	6.50758	21.11665	6.71524
H	7.56164	20.06876	7.66598
H	6.30308	21.00873	8.46361
C	5.64889	18.27704	8.53398
H	5.42335	18.73980	9.49595
H	6.65606	17.86247	8.60301
H	4.95473	17.44837	8.39200
Fe	5.55085	19.73256	2.12669
N	4.47402	16.98500	3.02082
N	4.17879	19.12093	4.81093
Si	3.84558	17.40896	4.60848

Aldridge B-Si-N, singlet, (C_1):

E_{tot} = -2363.36565325

Si	0.56861	-0.32634	0.87873
Si	0.10354	-1.88472	-1.88542
B	-0.71093	1.08744	0.11518
N	-0.21298	2.43529	0.08135
N	-2.13524	1.25813	-0.01148
N	0.89114	-1.54864	-0.31454
C	-1.27229	3.31789	-0.05621
C	-2.41809	2.61677	-0.11232
C	1.10444	2.88412	0.35794
C	1.50239	3.02374	1.69733
C	2.79221	3.47653	1.95189
C	3.65431	3.79779	0.91905
C	3.24066	3.66521	-0.39361
C	1.96669	3.19917	-0.69986
C	0.55485	2.76982	2.85076
C	1.19635	1.97622	3.98584
C	-0.01631	4.09467	3.36047
C	1.52599	3.09604	-2.14300
C	1.13413	4.47300	-2.68229
C	2.58348	2.45227	-3.03733
C	-3.18045	0.30175	0.00254
C	-3.43892	-0.41779	1.18374
C	-4.46472	-1.35497	1.16741
C	-5.23698	-1.55780	0.03617
C	-4.98843	-0.82222	-1.10434
C	-3.95516	0.10948	-1.14974
C	-2.68032	-0.14221	2.46825
C	-3.16541	1.15812	3.11351
C	-2.73857	-1.28305	3.47767
C	-3.72631	0.87916	-2.43501
C	-4.84192	1.90126	-2.66301
C	-3.60411	-0.04281	-3.64811
C	1.91004	-2.48290	0.07027
C	3.23887	-2.23727	-0.31992
C	4.22219	-3.14897	0.04157
C	3.91557	-4.28366	0.77114
C	2.61004	-4.50801	1.15950
C	1.59016	-3.62176	0.82331
C	3.63447	-0.97615	-1.05900
C	4.15453	0.06847	-0.06996
C	4.65464	-1.21703	-2.16978
C	0.19199	-3.91669	1.32293
C	-0.30345	-5.28576	0.85776
C	0.13125	-3.79824	2.84614
C	1.15594	-3.11964	-2.83746
C	-1.58777	-2.65762	-1.61444
C	-0.02450	-0.31756	-2.91311
H	-1.12889	4.38550	-0.10032
H	-3.42724	2.98275	-0.20721
H	3.12754	3.58974	2.97484
H	4.65197	4.15513	1.13871
H	3.92383	3.92608	-1.19218
H	-0.28606	2.17730	2.48619
H	0.44378	1.72897	4.73647
H	1.98121	2.53801	4.49536
H	1.62064	1.04190	3.61896
H	-0.73753	3.91897	4.16060
H	-0.52747	4.64071	2.56747
H	0.77125	4.73914	3.75553
H	0.63877	2.46232	-2.18032
H	0.78317	4.39994	-3.71308
H	0.33567	4.92229	-2.09192
H	1.98316	5.15894	-2.66608
H	2.17786	2.27750	-4.03483
H	2.91565	1.49281	-2.63880
H	3.46554	3.08338	-3.15726
H	-4.67990	-1.93222	2.05588
H	-6.03512	-2.28839	0.04955
H	-5.60058	-0.98415	-1.98282
H	-1.62120	-0.00729	2.23243
H	-2.59607	1.36838	4.02113
H	-4.21885	1.08139	3.38908
H	-3.05240	2.01385	2.44933
H	-2.04939	-1.08038	4.29752
H	-3.73184	-1.40088	3.91480
H	-2.44713	-2.23478	3.03291
H	-2.78602	1.42655	-2.35181
H	-4.65012	2.48789	-3.56284
H	-5.80796	1.40889	-2.78802
H	-4.93751	2.59534	-1.82835
H	-3.32798	0.53355	-4.53229
H	-4.54228	-0.55154	-3.87483
H	-2.84204	-0.80462	-3.49430
H	5.25038	-2.96988	-0.24624
H	4.69404	-4.98483	1.04198
H	2.37673	-5.39018	1.74361
H	2.74150	-0.56225	-1.53148
H	4.41272	0.99866	-0.57820
H	3.41408	0.30875	0.69606
H	5.04585	-0.29577	0.44445
H	4.80215	-0.30255	-2.74684
H	4.32900	-1.99760	-2.85832
H	5.63213	-1.50576	-1.78088
H	-0.49036	-3.17049	0.91464
H	-1.34620	-5.43275	1.14386
H	-0.23578	-5.38985	-0.22601
H	0.27252	-6.10039	1.29957
H	-0.87874	-3.99565	3.21042
H	0.80020	-4.51138	3.33088
H	0.41992	-2.79786	3.17082
H	2.14767	-2.73270	-3.07307
H	0.66679	-3.35986	-3.78437
H	1.29507	-4.05452	-2.29368
H	-2.23675	-2.05343	-0.98046
H	-2.10426	-2.79705	-2.56642
H	-1.50155	-3.64298	-1.15522
H	-0.70299	0.42069	-2.48850
H	-0.38526	-0.55873	-3.91539
H	0.95184	0.15510	-3.02597

Aldridge B-Si-N, triplet, (C_1):

E_{tot} = -2363.33183379

Si	0.45026	0.12365	-0.91276
Si	1.51941	-2.68633	-1.57898
B	-1.16455	0.61146	0.13923
N	-1.35685	1.95058	0.60584
N	-2.40712	-0.04798	0.41417
N	1.56332	-1.19450	-0.61924
C	-2.63231	2.05749	1.13282
C	-3.25769	0.86871	1.01789
C	-0.52902	3.08163	0.37807
C	0.50293	3.38223	1.27355
C	1.29218	4.49725	1.01325
C	1.07269	5.28487	-0.10221
C	0.05337	4.96642	-0.98084
C	-0.76429	3.86388	-0.76020
C	0.75920	2.55351	2.51056
C	2.23116	2.17764	2.66059
C	0.23896	3.27379	3.75491
C	-1.84600	3.53045	-1.76550
C	-2.82891	4.68602	-1.94706
C	-1.23426	3.10822	-3.10122
C	-2.89230	-1.29455	-0.06171
C	-3.01993	-2.37617	0.81839
C	-3.55413	-3.56385	0.32866
C	-3.93583	-3.68448	-0.99371
C	-3.79288	-2.60998	-1.85242
C	-3.27710	-1.39752	-1.40854
C	-2.62717	-2.27596	2.27624
C	-3.85213	-2.00741	3.15302
C	-1.89980	-3.52594	2.76672
C	-3.21179	-0.22391	-2.36281
C	-4.57457	0.46417	-2.45554
C	-2.69702	-0.60998	-3.74635
C	2.69740	-0.91465	0.20062
C	3.77921	-0.17419	-0.31208
C	4.87846	0.05962	0.50653
C	4.93143	-0.42694	1.79964
C	3.86006	-1.14345	2.29672
C	2.73109	-1.38918	1.52216
C	3.78842	0.40683	-1.71064
C	3.72939	1.93468	-1.66489
C	4.98924	-0.07417	-2.52493
C	1.57359	-2.13627	2.14968
C	1.97615	-3.54665	2.58022
C	0.99919	-1.35193	3.32925
C	3.12616	-3.61990	-1.30045
C	0.04641	-3.73622	-1.07322
C	1.37157	-2.26685	-3.40953
H	-3.00138	2.97820	1.55501
H	-4.25680	0.60231	1.32155
H	2.09549	4.75332	1.69238
H	1.70007	6.14669	-0.28901
H	-0.10424	5.58324	-1.85735
H	0.19608	1.62440	2.41237
H	2.36404	1.49458	3.50023
H	2.86140	3.04789	2.85183
H	2.61020	1.67688	1.76943
H	0.38364	2.65902	4.64496
H	-0.82567	3.49511	3.67189
H	0.76208	4.21854	3.91480
H	-2.42343	2.68370	-1.39260
H	-3.63443	4.39721	-2.62420
H	-3.27935	4.97593	-0.99755
H	-2.34915	5.57032	-2.36958
H	-2.01487	2.81028	-3.80426
H	-0.55523	2.26278	-2.97688
H	-0.66872	3.92072	-3.56085
H	-3.66908	-4.41364	0.98867
H	-4.34443	-4.61836	-1.35703
H	-4.09805	-2.71612	-2.88541
H	-1.94225	-1.43155	2.38476
H	-3.56332	-1.90917	4.20080

H	-4.57115	-2.82554	3.08012
H	-4.36639	-1.09089	2.86703
H	-1.52717	-3.37353	3.78047
H	-2.55365	-4.39843	2.80112
H	-1.05102	-3.77083	2.12851
H	-2.50577	0.50470	-1.96508
H	-4.51922	1.34016	-3.10518
H	-5.33076	-0.20818	-2.86527
H	-4.91921	0.79877	-1.47707
H	-2.54255	0.28514	-4.35015
H	-3.39476	-1.24734	-4.29186
H	-1.74313	-1.13381	-3.68035
H	5.71426	0.63300	0.12340
H	5.79975	-0.24137	2.41816
H	3.89817	-1.51251	3.31493
H	2.89495	0.06577	-2.23500
H	3.67873	2.34865	-2.67355
H	2.85168	2.28194	-1.11747
H	4.61221	2.35469	-1.17914
H	4.93304	0.30555	-3.54646
H	5.02710	-1.16299	-2.57585
H	5.93582	0.26772	-2.10353
H	0.78062	-2.22444	1.40507
H	1.12247	-4.08312	2.99752
H	2.35576	-4.12962	1.74089
H	2.75456	-3.53033	3.34483
H	0.16054	-1.88521	3.77797
H	1.74175	-1.19280	4.11315
H	0.63954	-0.37386	3.01041
H	3.99368	-3.03447	-1.60744
H	3.12882	-4.54232	-1.88474
H	3.28075	-3.89219	-0.25663
H	-0.89452	-3.19171	-1.17125
H	-0.03085	-4.62824	-1.69806
H	0.12196	-4.07102	-0.03721
H	0.47175	-1.69021	-3.62881
H	1.32833	-3.17700	-4.01143
H	2.22565	-1.68403	-3.75895

Aldridge N-Si-Si, singlet, (C_1):

E_{tot} = -2734.09531327

C	9.29868	-0.21524	18.24175
C	9.28195	-1.61852	18.21008
C	9.40394	-2.25696	16.97869
C	9.51269	-1.53977	15.80340
C	9.47959	-0.15652	15.84382
C	9.36444	0.52619	17.04797
C	9.05309	-2.46405	19.44576
C	7.66939	-3.11590	19.39772
C	10.12942	-3.52590	19.64384
C	9.32889	2.03992	17.05816
C	10.74115	2.61329	17.13585
C	8.57060	2.63887	15.87640
C	6.32159	0.61047	18.63084
C	7.47319	2.74482	20.51051
C	7.04824	-0.08767	21.54877
C	14.19331	3.07402	18.40825
C	12.77746	3.60424	21.06524
C	15.47223	2.13183	20.97641
C	12.98204	0.47071	23.40785
C	14.94492	-1.45424	21.99540
C	12.02567	-2.21944	22.37909
C	15.51777	-0.68038	18.31209
C	13.26213	-2.70549	18.01884
C	13.16518	-0.08824	16.44988
Si	10.43937	0.91273	20.59652
Si	12.63875	0.26337	19.92411
Si	13.80043	2.33235	20.10759
Si	13.18118	-0.76997	21.99170
Si	13.64096	-0.85736	18.10440
Si	7.52664	0.92460	20.03783
N	9.18407	0.47313	19.48249
H	9.39686	-3.33972	16.93778
H	9.60787	-2.05461	14.85650
H	9.54318	0.39776	14.91636
H	9.08292	-1.81312	20.32108
H	7.47095	-3.66945	20.31734
H	6.87550	-2.37926	19.27429
H	7.59220	-3.81907	18.56662
H	9.95493	-4.07471	20.57042
H	11.11710	-3.07476	19.70548
H	10.14504	-4.25483	18.83175
H	8.80646	2.35970	17.96072
H	10.71928	3.70031	17.23504
H	11.28849	2.20676	17.98748
H	11.31373	2.36656	16.24027
H	8.45321	3.71430	16.01565
H	7.57550	2.20475	15.77217
H	9.09552	2.49826	14.93048
H	6.34200	-0.41905	18.27381
H	5.30486	0.82141	18.96907
H	6.51612	1.25160	17.77041
H	8.12400	2.96172	21.35776
H	6.45680	3.02747	20.79424
H	7.77364	3.39679	19.68902
H	7.76204	0.06262	22.36041
H	6.06734	0.22030	21.91709
H	6.99705	-1.15709	21.34427
H	14.81693	2.41289	17.80524
H	14.74452	4.00860	18.53692
H	13.29973	3.30057	17.82794
H	12.49045	3.24607	22.05413
H	13.34624	4.52768	21.19729
H	11.85644	3.86126	20.53843
H	15.37834	1.80105	22.01045
H	15.99799	3.08961	20.98910
H	16.11322	1.41582	20.46066
H	11.96161	0.85470	23.45367
H	13.19785	-0.01022	24.36482
H	13.64791	1.32876	23.31698
H	15.06830	-2.23800	21.24605
H	15.17861	-1.89448	22.96745
H	15.68991	-0.68481	21.79395
H	12.18734	-3.06109	21.70587
H	12.20059	-2.57753	23.39625
H	10.97168	-1.94277	22.30970
H	15.84024	0.36122	18.28968
H	15.87300	-1.11261	19.24777
H	16.03250	-1.19507	17.49712
H	12.23095	-2.88567	17.71682
H	13.42995	-3.21610	18.96839
H	13.90999	-3.17734	17.27641
H	12.10018	-0.20599	16.25262
H	13.39666	0.97626	16.40983
H	13.71044	-0.57593	15.63845

Aldridge N-Si-Si, triplet, (C_1):

E_{tot} = -2734.05826754

C	9.23263	-0.25347	17.97497
C	9.22484	-1.66205	17.98929
C	9.28150	-2.34430	16.77840
C	9.34544	-1.66981	15.57328
C	9.31507	-0.28819	15.56748
C	9.24199	0.44085	16.74976
C	9.07968	-2.45664	19.27124
C	7.64323	-2.96505	19.42010
C	10.04268	-3.63621	19.37391
C	9.14969	1.95078	16.65484
C	10.43517	2.56863	16.10590
C	7.94678	2.38303	15.81389
C	6.29844	0.64299	18.99560
C	7.87039	2.71141	20.59578
C	7.66108	-0.10516	21.69969
C	14.29859	3.20158	18.66341
C	12.96740	3.61524	21.38919
C	15.64889	2.13050	21.22630
C	12.69371	0.42265	23.54511
C	14.70893	-1.57096	22.30818
C	11.69030	-2.16635	22.24820
C	15.75083	-0.55053	18.45369
C	13.49268	-2.58627	18.18079
C	13.31099	0.04474	16.64063
Si	10.76987	1.24648	19.63969
Si	12.86652	0.35107	20.09234
Si	13.99296	2.41148	20.35362
Si	12.98808	-0.80151	22.13555
Si	13.87521	-0.73873	18.25903
Si	7.79305	0.89241	20.10785
N	9.25689	0.46551	19.20565
H	9.27531	-3.42727	16.77521
H	9.40326	-2.21889	14.64265
H	9.34095	0.23681	14.62002
H	9.29730	-1.78964	20.10716
H	7.51437	-3.49139	20.36803
H	6.91283	-2.15819	19.38722
H	7.39280	-3.66218	18.61819
H	9.92822	-4.12695	20.34145
H	11.07892	-3.31854	19.28527
H	9.85761	-4.39162	18.60859
H	9.00012	2.35146	17.65860
H	10.35059	3.65594	16.06184
H	11.28967	2.32436	16.73718
H	10.65310	2.21255	15.09734
H	7.83176	3.46774	15.84543
H	7.02109	1.93576	16.17600
H	8.05797	2.10002	14.76608
H	6.29991	-0.31128	18.47008
H	5.37693	0.69832	19.57822
H	6.24875	1.42540	18.23713
H	8.67010	2.92318	21.30695
H	6.93173	3.01212	21.06738
H	8.02071	3.35860	19.72992
H	8.58479	-0.02826	22.27783
H	6.85590	0.27942	22.32904
H	7.46549	-1.16373	21.53138
H	14.94153	2.58849	18.03059
H	14.77888	4.17648	18.77060
H	13.35986	3.35343	18.12780
H	12.72327	3.20716	22.37014
H	13.51385	4.54824	21.54495
H	12.02568	3.86429	20.89729
H	15.50744	1.73970	22.23537
H	16.20218	3.06772	21.31637
H	16.28311	1.42490	20.68877
H	11.73763	0.93494	23.42562
H	12.67786	-0.08751	24.51041
H	13.47124	1.18642	23.59184
H	14.89961	-2.29904	21.51751
H	14.81601	-2.09089	23.26253
H	15.49596	-0.81742	22.25424
H	11.85483	-2.94176	21.50032
H	11.71372	-2.64452	23.22972
H	10.68358	-1.77370	22.09583
H	16.06757	0.49274	18.42532
H	16.10270	-0.97454	19.39579
H	16.27212	-1.07047	17.64681
H	12.47855	-2.75827	17.82058
H	13.59825	-3.08433	19.14591
H	14.17316	-3.07955	17.48315
H	12.24093	-0.10841	16.48837
H	13.49692	1.11856	16.61267
H	13.83304	-0.40410	15.79266

Power S-Si-S, singlet, (C_1):

E_{tot} = -2939.91879950

S	10.62351	3.07078	11.08831
Si	9.17352	2.59284	12.62938
C	11.94210	3.77123	12.04851
S	7.63575	2.53032	11.09788

C	11.73743	4.74980	13.03242
C	12.85085	5.32812	13.63668
H	12.69383	6.09620	14.38406
C	14.13672	4.94885	13.29434
H	14.98713	5.41685	13.77223
C	14.32549	3.96443	12.33910
H	15.32488	3.64589	12.06978
C	13.23979	3.36852	11.70979
C	10.38969	5.18994	13.48457
C	9.60399	6.02836	12.68526
C	8.35481	6.42793	13.15331
H	7.74179	7.06431	12.52481
C	7.88118	6.04532	14.39876
C	8.69302	5.24120	15.19178
H	8.33904	4.93015	16.16881
C	9.93670	4.80762	14.76061
C	10.07694	6.48949	11.34040
H	9.49191	7.33888	10.99037
H	9.97974	5.69227	10.59970
H	11.12595	6.78484	11.35913
C	6.53207	6.48038	14.89057
H	6.05879	7.17821	14.20145
H	6.60284	6.97037	15.86258
H	5.85941	5.62852	15.00667
C	10.74647	3.89182	15.63051
H	10.19578	3.62854	16.53277
H	11.69336	4.33699	15.93609
H	10.98316	2.96303	15.10550
C	13.47349	2.31076	10.69394
C	13.72090	2.66250	9.36496
C	13.95824	1.65713	8.43658
H	14.14464	1.92937	7.40325
C	13.95352	0.31537	8.79586
C	13.69221	-0.00895	10.12035
H	13.67258	-1.05213	10.41729
C	13.44879	0.96822	11.07747
C	13.69754	4.10166	8.93839
H	13.89523	4.19907	7.87165
H	14.43994	4.69792	9.47045
H	12.72487	4.55579	9.13802
C	14.24545	-0.75237	7.78213
H	13.80538	-1.70779	8.06651
H	15.32069	-0.90961	7.67449
H	13.85777	-0.48857	6.79825
C	13.13013	0.58234	12.49165
H	13.24862	-0.49039	12.64336
H	12.09752	0.83874	12.74307
H	13.77054	1.09438	13.21082
C	6.20064	1.74978	11.79350
C	6.23117	0.73677	12.76131
C	5.03728	0.12584	13.13303
H	5.06988	-0.66454	13.87301
C	3.82935	0.50571	12.57549
H	2.91223	0.01653	12.87556
C	3.80678	1.51852	11.63189
H	2.87020	1.83510	11.18984
C	4.97962	2.14566	11.23025
C	7.48372	0.30853	13.43156
C	7.72995	0.71839	14.75001
C	8.91556	0.34027	15.36164
H	9.11314	0.67262	16.37501
C	9.87663	-0.40836	14.69348
C	9.60334	-0.82136	13.39609
H	10.33282	-1.42313	12.86541
C	8.40939	-0.49712	12.75787
C	6.77717	1.63154	15.46475
H	7.16157	1.90214	16.44737
H	5.79306	1.18383	15.60289
H	6.62847	2.55581	14.90111
C	11.18217	-0.73746	15.35523
H	11.72543	-1.51067	14.81367
H	11.03624	-1.08640	16.37781
H	11.82787	0.14189	15.40198
C	8.13758	-0.99124	11.36875
H	8.87417	-1.73425	11.06665
H	8.17417	-0.17598	10.64256
H	7.14864	-1.44384	11.29276
C	4.91445	3.21778	10.20371
C	4.83288	4.55424	10.59794
C	4.73802	5.53877	9.62153
H	4.66940	6.57720	9.92752
C	4.73173	5.22760	8.26903
C	4.82413	3.89072	7.90174
H	4.82729	3.62813	6.84921
C	4.91879	2.87903	8.84767
C	4.88236	4.92862	12.04931
H	4.68052	5.99038	12.18904
H	5.87138	4.72302	12.46672
H	4.15859	4.36968	12.64343
C	4.65984	6.30473	7.22616
H	4.27171	7.23660	7.63611
H	4.01801	6.01595	6.39360
H	5.64795	6.51723	6.81311
C	5.05393	1.44691	8.41847
H	5.98623	1.01421	8.78684
H	5.05545	1.36024	7.33268
H	4.24348	0.82552	8.80155

Power S-Si-S, triplet, (C_1):

E_{tot} = -2939.85872870

S	10.54671	2.73265	10.90587
Si	9.32361	2.18591	12.56855
C	11.82419	3.82182	11.49343
S	7.36951	3.01347	12.82050
C	11.55020	5.02506	12.15241
C	12.61770	5.85070	12.49187
H	12.40886	6.78717	12.99413
C	13.92313	5.49777	12.19406
H	14.73827	6.15697	12.46201
C	14.18021	4.29449	11.55906
H	15.19768	3.99917	11.33485
C	13.14061	3.44204	11.20581
C	10.18012	5.44276	12.54201
C	9.29877	5.98838	11.60413
C	8.04825	6.42052	12.02909
H	7.35739	6.82398	11.29804
C	7.65117	6.32519	13.35420
C	8.54448	5.78595	14.27033
H	8.24910	5.69528	15.31004
C	9.80443	5.34415	13.88918
C	9.67237	6.10027	10.15556
H	8.99401	6.77048	9.62885
H	9.61858	5.12574	9.66545
H	10.68783	6.47372	10.02103
C	6.27304	6.72941	13.78159
H	5.83157	7.44955	13.09380
H	6.27145	7.16785	14.77981
H	5.61352	5.85917	13.80129
C	10.73677	4.77330	14.92063
H	10.20374	4.55630	15.84553
H	11.54650	5.46432	15.16189
H	11.20168	3.84646	14.58122
C	13.44954	2.14971	10.54131
C	13.63099	2.10739	9.15640
C	13.94210	0.89528	8.55469
H	14.07769	0.86195	7.47892
C	14.07615	-0.27359	9.29357
C	13.88029	-0.20782	10.66608
H	13.97196	-1.11172	11.25886
C	13.56632	0.98615	11.30476
C	13.45981	3.34585	8.32492
H	13.61384	3.13406	7.26761
H	14.15958	4.13158	8.61242
H	12.45679	3.76229	8.43702
C	14.44541	-1.56565	8.62482
H	14.15659	-2.42692	9.22647
H	15.52306	-1.63047	8.46126
H	13.96519	-1.66516	7.65131
C	13.32136	1.00609	12.78497
H	13.62609	0.06754	13.24737
H	12.25892	1.14761	13.00293
H	13.85799	1.81491	13.28115
C	6.31252	1.72470	12.18475
C	6.41041	0.38906	12.59856
C	5.48480	-0.52822	12.11351
H	5.55774	-1.55996	12.43440
C	4.47946	-0.14060	11.24326
H	3.76510	-0.86744	10.87999
C	4.39714	1.18027	10.83829
H	3.62233	1.49437	10.14986
C	5.30819	2.12548	11.29853
C	7.48903	-0.07949	13.50538
C	7.43542	0.19753	14.87439
C	8.48549	-0.21853	15.68714
H	8.44564	0.00453	16.74767
C	9.57806	-0.90646	15.17975
C	9.59434	-1.20326	13.82211
H	10.43623	-1.74587	13.40560
C	8.56706	-0.80787	12.97471
C	6.27873	0.94379	15.46949
H	6.27218	0.85487	16.55522
H	5.32329	0.57555	15.09591
H	6.33413	2.00645	15.22317
C	10.72198	-1.31473	16.06093
H	11.63148	-0.77054	15.79937
H	10.94322	-2.37813	15.96198
H	10.51241	-1.11663	17.11120
C	8.63179	-1.15785	11.51559
H	9.64316	-1.44252	11.22872
H	8.33095	-0.32344	10.88186
H	7.97172	-1.99435	11.27783
C	5.19097	3.53448	10.83937
C	4.30946	4.40217	11.48923
C	4.17503	5.70300	11.02074
H	3.48951	6.37538	11.52572
C	4.89735	6.16251	9.92717
C	5.77202	5.28382	9.30250
H	6.34747	5.62453	8.44824
C	5.93641	3.97622	9.74342
C	3.53027	3.94515	12.68886
H	2.93899	4.75907	13.10695
H	4.19075	3.57457	13.47558
H	2.84727	3.12996	12.44576
C	4.76760	7.58412	9.46308
H	3.75160	7.95828	9.58818
H	5.03360	7.69065	8.41185
H	5.42593	8.24584	10.03157
C	6.92269	3.07182	9.06745
H	7.79630	2.91054	9.70340
H	7.27776	3.50321	8.13225
H	6.49987	2.09155	8.84694

References

[S1] R. S. Ghadwal, H. W. Roesky, S. Merkel, J. Henn, D. Stalke, *Angew. Chem. Int. Ed.* **2009**, *48*, 5683.

[S2] J. Oetzel, N. Weyer, C. Bruhn, M. Leibold, B. Gerke, R. Pöttgen, M. Maier, R. F. Winter, M. C. Holthausen, U. Siemeling, *Chem. Eur. J.* **2017**, *23*, 1187.

[S3] B. A. Correia Bicho, C. Bruhn, R. Guthardt, N. Weyer, U. Siemeling, *Z. Anorg. Allg. Chem.* **2018**, *644*, 1329.

[S4] H. Arp, C. Marschner, J. Baumgartner, *Dalton Trans.* **2010**, *39*, 9270.

[S5] G. M. Sheldrick, *Acta Crystallogr., Sect. A: Found. Crystallogr.* **2008**, *64*, 112.

[S6] J. Oetzel, C. Bruhn, U. Siemeling, *Z. Anorg. Allg. Chem.* **2018**, *644*, 935.

[S7] F. Neese, *Wiley Interdiscip. Rev. Comput. Mol. Sci.* **2018**, *8*, e1327.

[S8] S. Grimme, J. G. Brandenburg, C. Bannwarth, A. Hansen, *J. Chem. Phys.* **2015**, *143*, 054107.

[S9] K. Fukui, *Acc. Chem. Res.* **1981**, *14*, 363.

[S10] S. Ten-no, *Chem. Phys. Lett.* **2004**, *398*, 56.

[S11] T. B. Adler, G. Knizia, H.-J. Werner, *J. Chem. Phys.* **2007**, *127*, 221106.

[S12] H.-J. Werner, P. J. Knowles, G. Knizia, F. R. Manby, M. Schütz, *Wiley Interdiscip. Rev. Comput. Mol. Sci.* **2012**, *2*, 242.

[S13] K. A. Peterson, T. B. Adler, H.-J. Werner, *J. Chem. Phys.* **2008**, *128*, 084102.

[S14] F. Weigend, *J. Comput. Chem.* **2008**, *29*, 167.

[S15] F. Weigend, A. Köhn, C. Hättig, *J. Chem. Phys.* **2002**, *116*, 3175.

[S16] K. E. Yousaf, K. A. Peterson, *Chem. Phys. Lett.* **2009**, *476*, 303.

[S17] C. Riplinger, F. Neese, *J. Chem. Phys.* **2013**, *138*, 034106.

[S18] M. J. Frisch, G. W. Trucks, H. B. Schlegel, G. E. Scuseria, M. A. Robb, J. R. Cheeseman, G. Scalmani, V. Barone, B. Mennucci, G. A. Petersson, H. Nakatsuji, M. Caricato, X. Li, H. P. Hratchian, A. F. Izmaylov, J. Bloino, G. Zheng, J. L. Sonnenberg, M. Hada, M. Ehara, K. Toyota, R. Fukuda, J. Hasegawa, M. Ishida, T. Nakajima, Y. Honda, O. Kitao, H. Nakai, T. Vreven, J. A. Montgomery, Jr., J. E. Peralta, F. Ogliaro, M. Bearpark, J. J. Heyd, E. Brothers, K. N. Kudin, V. N. Staroverov, R. Kobayashi, J. Normand, K. Raghavachari, A. Rendell, J. C. Burant, S. S. Iyengar, J. Tomasi, M. Cossi, N. Rega, J. M. Millam, M. Klene, J. E. Knox, J. B. Cross, V. Bakken, C. Adamo, J. Jaramillo, R. Gomperts, R. E. Stratmann, O. Yazyev, A. J. Austin, R. Cammi, C. Pomelli, J. W. Ochterski, R. L. Martin, K. Morokuma, V. G. Zakrzewski, G. A. Voth, P. Salvador, J. J. Dannenberg, S. Dapprich, A. D. Daniels, O. Farkas, J. B. Foresman, J. V. Ortiz, J. Cioslowski, D. J. Fox, Gaussian, Inc., Wallingford CT, **2009**.

[S19] Y. Zhao, D. G. Truhlar, *J. Chem. Phys.* **2006**, *125*, 194101.

[S20] W. J. Hehre, R. Ditchfield, J. A. Pople, *J. Chem. Phys.* **1972**, *56*, 2257.

[S21] M. M. Francl, W. J. Pietro, W. J. Hehre, J. S. Binkley, M. S. Gordon, D. J. DeFrees, J. A. Pople, *J. Chem. Phys.* **1982**, *77*, 3654.

[S22] G. te Velde, F. M. Bickelhaupt, E. J. Baerends, C. Fonseca Guerra, S. J. A. van Gisbergen, J. G. Snijders, T. Ziegler, *J. Comput. Chem.* **2001**, *22*, 931.

[S23] C. Fonseca Guerra, J. G. Snijders, G. te Velde, E. J. Baerends, *Theor. Chem. Acc.* **1998**, *99*, 391.

[S24] ADF2013, SCM, Theoretical Chemistry, Vrije Universiteit, Amsterdam, The Netherlands, http://www.scm.com.

[S25] A. D. Becke, *Phys. Rev. A* **1988**, *38*, 3098.

[S26] C. Lee, W. Yang, R. G. Parr, *Phys. Rev. B* **1988**, *37*, 785.

[S27] E. Van Lenthe, E. J. Baerends, *J. Comput. Chem.* **2003**, *24*, 1142.
[S28] C. Y. Legault, Université de Sherbrooke, http://www.cylview.org, **2009**.
[S29] G. A. Andrienko, **2015**.
[S30] A. V. Protchenko, K. H. Birjkumar, D. Dange, A. D. Schwarz, D. Vidovic, C. Jones, N. Kaltsoyannis, P. Mountford, S. Aldridge, *J. Am. Chem. Soc.* **2012**, *134*, 6500.
[S31] A. V. Protchenko, A. D. Schwarz, M. P. Blake, C. Jones, N. Kaltsoyannis, P. Mountford, S. Aldridge, *Angew. Chem. Int. Ed.* **2013**, *52*, 568.
[S32] B. D. Rekken, T. M. Brown, J. C. Fettinger, H. M. Tuononen, P. P. Power, *J. Am. Chem. Soc.* **2012**, *134*, 6504.
[S33] A. D. Becke, *J. Chem. Phys.* **1993**, *98*, 5648.
[S34] F. Weigend, R. Ahlrichs, *Phys. Chem. Chem. Phys.* **2005**, *7*, 3297.
[S35] J.-D. Chai, M. Head-Gordon, *Phys. Chem. Chem. Phys.* **2008**, *10*, 6615.
[S36] E. Wigner, *Phys. Rev.* **1932**, *40*, 749.
